# Integrated MicroRNA Expression Profile Reveals Dysregulated miR-20a-5p and miR-200a-3p in Liver Fibrosis

**DOI:** 10.1155/2021/9583932

**Published:** 2021-06-08

**Authors:** Mu Ye, Sheng Wang, Peilong Sun, Jingbo Qie

**Affiliations:** ^1^Department of General Surgery, Jinshan Hospital, Fudan University, Shanghai 201508, China; ^2^Institutes of Biomedical Sciences, Fudan University, Shanghai 200032, China

## Abstract

MicroRNAs (miRNAs) have been demonstrated to involve in liver fibrogenesis. However, the miRNA-gene regulation in liver fibrosis is still unclear. Herein, the miRNA expression profile GSE40744 was obtained to analyze the dysregulated miRNAs between liver fibrosis and normal samples. Then, we predicted the target genes of screened miRNAs by miRTarBase, followed by gene ontology (GO) and Kyoto Encyclopedia of Genes and Genomes (KEGG) analysis. Then, the protein-protein interaction (PPI) network was constructed to identify the functional miRNA-gene regulatory modules. Furthermore, we verified the hub gene expression using the gene expression profile GSE14323. Finally, 89 DEMs were identified in fibrotic liver samples compared to normal liver samples. The top 3 upregulated DEMs (miR-200b-3p, miR-200a-3p, and miR-182-5p) and downregulated DEMs (miR-20a-5p, miR-194-3p, and miR-148a-3p) were further studied. 516 and 1416 target genes were predicted, respectively. KEGG analysis demonstrated that the predicted genes were enriched in the p53 signaling pathway and hepatitis B, etc. Through constructing a PPI network, the genes with the highest connectivity were identified as hub genes. Of note, most of the hub genes were potentially targeted by miR-20a-5p and miR-200a-3p. Based on the data from GSE14323, the expression of EGFR, STAT3, CTNNB1, and TP53 targeted by miR-200a-3p was significantly downregulated in fibrotic liver samples. Oppositely, the expression of PTEN, MYC, MAPK1, UBC, and CCND1 potentially targeted by miR-20a-5p was significantly upregulated. In conclusion, it is demonstrated that miR-20a-5p and miR-200a-3p were identified as the novel liver fibrosis-associated miRNAs, which may play critical roles in liver fibrogenesis.

## 1. Introduction

Liver fibrosis results from chronic liver disease, which has the potential to progress into cirrhosis, complicating with the loss of architecture and attendant functional failure [1]. If advanced chronic fibrosis is not prevented, liver fibrosis may lead to liver cancer and liver failure, which are major causes of morbidity and mortality worldwide [2–5]. Increasing studies report that hepatocytes activated hepatic stellate cells and macrophages and cooperate in liver fibrosis. However, the underlying mechanism remains incompletely uncovered.

MicroRNA (miRNA) is a type of highly conserved tissue-specific nonsmall protein noncoding RNA. The dysregulation of miRNA expression is related to various cancers by acting as tumor suppressor and oncogene. miRNA actually participates at the posttranscriptional level and binds to the 3′UTR of its target messenger RNA (mRNA) to inhibit expression. The dysfunction of miRNA interferes with the expression of carcinogenic or tumor suppressor target genes, which is related to the pathogenesis of cancer [6]. Compelling evidence indicates that dysregulated expression of miRNAs is found in many types of diseases, including liver fibrosis [7–11]. It is well-studied that many miRNAs are aberrantly expressed in liver tissues from the patients with liver fibrosis. As we know, it is a complicated pathological process of liver fibrogenesis, involving large numbers of miRNAs and genes. To dig the miRNAs-to-gene regulatory network in complex cellular systems, increasing studies focus on the identification of novel liver fibrosis-associated miRNAs and their regulatory networks [12–16]. For example, miRNA-221 and miRNA-222 are screened out as the biomarkers in liver fibrosis [17], and miR-19b-3p [18], miRNA-181a [19, 20], miR-873-5p [21], and miR-34a-5p [22] are reported to regulate the progression of liver fibrosis.

This study analyzed the GSE40744 data set and identified DEMs between fibrotic liver samples and normal liver samples. Using bioinformatics tools to predict various key miRNA target genes. On this basis, in corresponding databases, such as Kyoto Encyclopedia of Genes and Genomes (KEGG) and Gene Ontology (GO) databases, the consensus targets are combined for further analysis. Then, the expression levels of hub target genes were explored in the GSE14323 database. This is of great value for improving the diagnosis and treatment of liver fibrosis.

## 2. Materials and Methods

### 2.1. Identification of DEMs Associated with Liver Fibrosis

GEO2R [23] was used to identify DEMs from the data of GSE40744 [24], containing the miRNA expression data from 18 fibrotic and 19 normal liver samples. The miRNAs with adjusted *P* value (adj. *P*) < 0.05 and ∣log2 (fold change)  | >1 were identified as DEMs. GSE14323 is a gene expression microarray, including 41 fibrosis and 19 normal liver samples, used to evaluate miRNA-regulated hub gene expression.

### 2.2. Prediction of Target Genes

We used miRTarBase (http://mirtarbase.mbc.nctu.edu.tw/php/index.php) to predict the target genes [25].

### 2.3. GO and KEGG Pathway Analysis

DAVID (https://david.ncifcrf.gov/) was introduced to perform GO and KEGG pathway enrichment analysis [26, 27]. FDR < 0.05 was statistically significant.

### 2.4. Construction of PPI and miRNA-Gene Network

STRING (http://string-db.org) was used to construct the PPI network and to identify the hub genes with the highest connectivity [28].

### 2.5. Statistical Analysis

The gene expression was shown as mean ± SD. Unpaired Student's *t*-test in Graphpad software was performed to estimate the differences. *P* < 0.05 was considered statistically significant.

## 3. Results

### 3.1. DEM Identification and Target Gene Prediction

**Figure 1:**
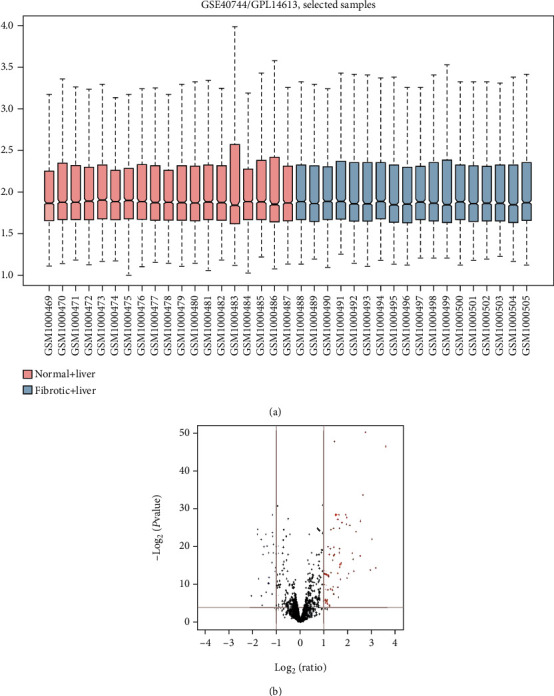
Identification of different expression miRNAs. (a) GSE40744 data after normalization. (b) Volcano plot of the DEMs. The black dots represent miRNAs that are not differentially expressed between liver fibrosis samples and normal liver samples, and the red dots and blue dots represent the upregulated and downregulated miRNAs in liver fibrosis samples, respectively.

In our study, we obtained the miRNA expression profile GSE40744 from GEO and checked the data consistency. The normalized data was visualized (Figure 1(a)) and then conducted a differential expression analysis using the GEO2R tool. Totally, 89 miRNAs were identified as DEMs between liver fibrosis samples and normal liver samples, including 62 upregulated miRNAs and 27 downregulated miRNAs (Figure 1(b)). As listed in Tables 1 and 2, miR-182-5p, miR-200a-3p, miR-200b-3p, miR-20a-5p, miR-194-3p, and miR-148a-3p were screened out as the top changed miRNAs, which was selected for further study. Furthermore, 516 and 1416 genes were predicted to be regulated by the top 3 upregulated and downregulated miRNAs through miRTarBase, respectively. (Table S1).

### 3.2. GO Functional Enrichment Analysis

**Figure 2:**
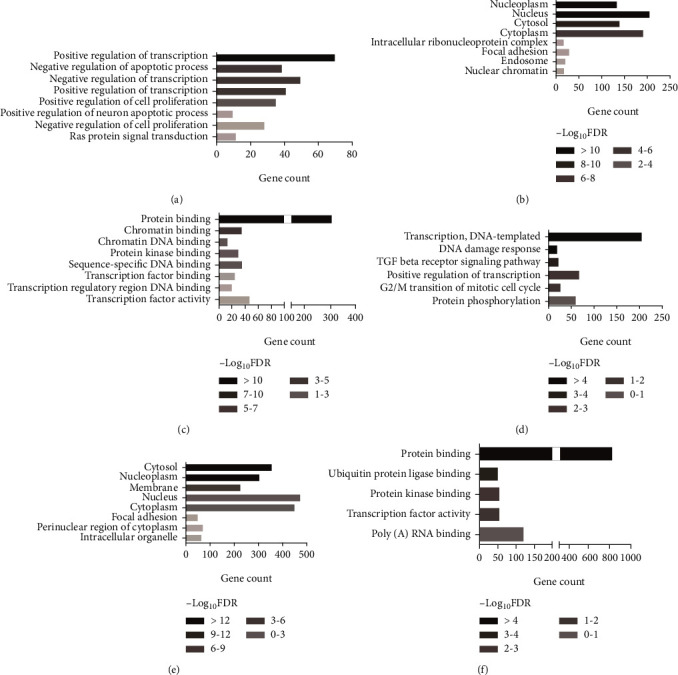
GO functions for the target genes of top three upregulated miRNAs and top three downregulated miRNAs. (a) Enriched biological process of the upregulated miRNAs. (b) Enriched cellular component of the upregulated miRNAs. (c) Enriched molecular function of the upregulated miRNAs. (d) Enriched biological process of the downregulated miRNAs. (e) Enriched cellular component of the downregulated miRNAs. (f) Enriched molecular function of the downregulated miRNAs.

DAVID and STRING databases were used to obtain the GO functional annotation of the target genes. For the genes targeted by the 3 upregulated miRNAs (Table S2), positive regulation of transcription from RNAP II promoter, negative regulation of apoptotic process, negative regulation of transcription from RNA RNAP II promoter, positive regulation of transcription, and DNA-templated and positive regulation of cell proliferation were enriched in the biological process (BP) category (Figure 2(a)); nucleoplasm, nucleus, cytosol, cytoplasm, intracellular ribonucleoprotein complex, and focal adhesion were enriched in the cellular component (CC) category (Figure 2(b)); protein binding, chromatin binding, chromatin DNA binding, protein kinase binding, and sequence-specific DNA binding were enriched in the molecular function (MF) category (Figure 2(c)). For the genes targeted by the 3 downregulated miRNAs (Table S3), regulation of transcription, DNA damage response, TGFBR signaling pathway, positive regulation of transcription, and cell cycle were enriched in the BP category; cytosol, nucleoplasm, membrane, nucleus, and cytoplasm were enriched in the CC category; protein binding, ubiquitin-protein ligase binding, protein kinase binding, protein kinase activity, and poly(A) RNA binding were enriched in the MF category.

### 3.3. KEGG Pathway Analysis

**Figure 3:**
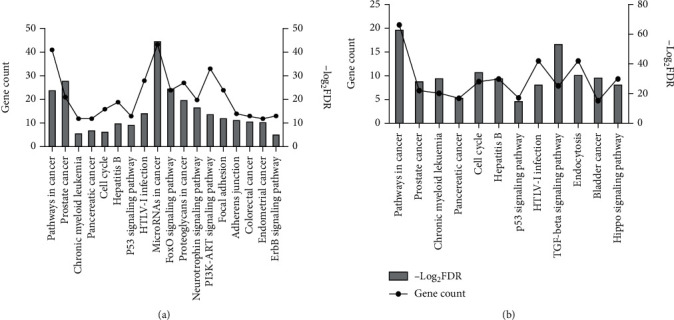
KEGG pathway enrichment analysis of target genes of six selected DEMs. (a) For upregulated miRNAs. (b) For downregulated miRNAs. The lines represent gene count and the columns represent—log_2_ FDR.

To demonstrate the biological function of the 6 selected DEMs, the target genes were subsequently used in KEGG pathway analysis. For 3 upregulated miRNAs (Figure 3(a), Table S4), 18 significantly enriched KEGG pathways were identified, containing microRNAs in cancer, prostate cancer, FoxO signaling pathway, pathways in cancer, and proteoglycans in cancer. And 12 significantly enriched KEGG pathways for downregulated miRNAs were identified (Figure 3(b), Table S4), including pathways in cancer, TGF-beta signaling pathway, cell cycle, endocytosis, and bladder cancer. Interestingly, both of the 2 groups of genes targeted by upregulated miRNAs or downregulated miRNAs were enriched in the process, prostate cancer, pathways in cancer, HTLV-I infection, hepatitis B, p53 signaling pathway, pancreatic cancer, cell cycle, and chronic myeloid leukemia. Excluding the process of cancer, it is evident that hepatitis B, p53 signaling pathway, and cell cycle play central roles in liver fibrosis. Therefore, the genes targeted by upregulated miRNAs or downregulated miRNA, which are included in these three pathways, may modulate liver fibrosis progress.

### 3.4. PPI and miRNA-Hub Gene Network

The results revealed that most of the target genes displayed broad external relationships. According to the node degree, the genes with the highest connectivity were identified as the hub genes (Table 3), including TP53, EGFR, PTEN, JUN, VEGFA, KRAS, STAT3, CTNNB1, NOTCH1, and EP300 for the 3 upregulated miRNAs, and TP53, UBC, RPS27A, MYC, HSP90AA1, MAPK1, PTEN, CCND1, HSPA8, and VEGFA for the downregulated miRNAs. Collectively, TP53 gained the highest connectivity in both groups (136 and 235). Meanwhile, TP53 and PTEN were the only two hub genes enriched in all three pathways.

**Figure 4:**
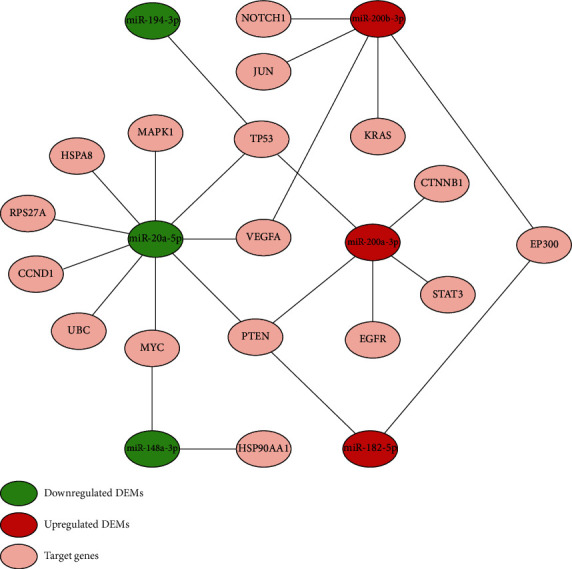
The regulatory network between dysregulated miRNAs and hub genes.

Subsequently, the miRNA-hub gene network was constructed (Figure 4). We found that nine in ten hub genes (TP53, PTEN, RPS27A, MYC, MAPK1, UBC, CCND1, HSPA8, and VEGFA) were potentially regulated by miR-20a-5p. miR-200a-3p could potentially target five (TP53, PTEN, EGFR, STAT3, and CTNNB1) in ten hub genes. In another way, we also found that TP53, as the highest-degree node, was potentially targeted by miR-20a-5p, miR-200a-3p, and miR-194-3p; PTEN was potentially regulated by miR-20a-5p, miR-200a-3p, and miR-182-5p. The results above prompted that miR-20a-5p and miR-200a-3p are two potential regulators in liver fibrogenesis.

### 3.5. Evaluation of the Hub Gene Expression

**Figure 5:**
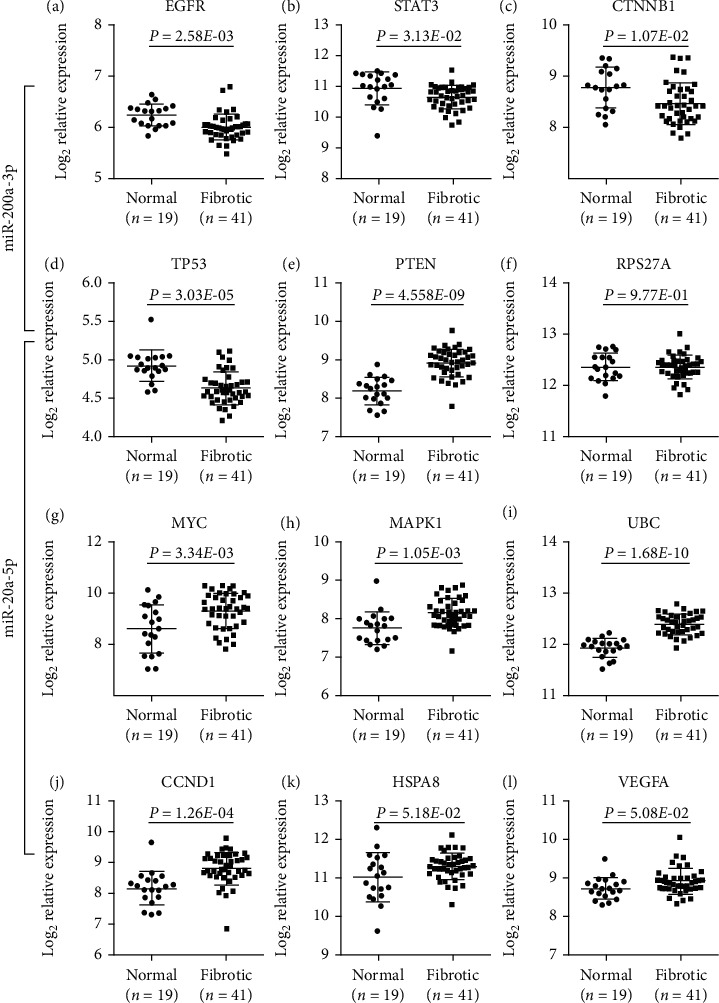
The mRNA expression of predicted targets of miR-200a-3p and miR-20a-5p from the GSE14323 dataset.

Because there was no survival outcome data of miRNA in fibrosis, we just evaluated the hub gene expression regulated by the two selected miRNAs using GSE14323. We found that the expression of four (EGFR, STAT3, CTNNB1, and TP53) in five targets of miR-200a-3p (upregulated) was significantly downregulated in fibrotic liver samples compared with normal liver samples (Figures 5(a)–5(d)). Similarly, the upregulated genes, PTEN, MYC, MAPK1, UBC, and CCND1, may be regulated by miR-20a-5p (Figures 5(e) and 5(g)–5(j)). Although HSPA8 and VEGFA in fibrotic liver samples were not significantly higher than that in normal liver samples, the *P* values were near 0.05 (Figures 5(k) and 5(l)). And the expression level of RPS27A in normal liver tissue and fibrotic liver tissue has no significant difference (Figure 5(f)). To verify the relationship between miRNAs and genes, we rechecked the data from miRTarBase [29]. The regulation of PTEN, MYC, CCND1, and VEGFA by hsa-miR-20a-5p was identified in at least one luciferase reporter assay, qRT-PCR, ELISA, and western blot, previously. The result was similar for hsa-miR-200a-3p. So, it was reasonable that the miRNAs identified in our study participated in the liver fibrosis progress via regulating their target genes.

## 4. Discussion

Although it is extensively reported that miRNAs participate in the whole process of liver fibrogenesis, including cell death, the proinflammatory factor secretion by macrophage, and hepatic stellate cell (HSC) activation [11, 30], the profiles of liver fibrosis available are limited. In our study, we reanalyzed the miRNA expression profile GSE40744 for the identification of DEMs and the gene expression profile GSE14323 for validation of target gene expression. Finally, 89 DEMs were identified, including 27 downregulated DEMs and 62 upregulated DEMs.

Pathway enrichment analysis found that the top 3 upregulated miRNAs and top 3 downregulated miRNAs are involved in the regulation of signal pathways. For example, the hallmark feature of cancer is abnormal cell proliferation. Cell cycle dysregulation is the main body of abnormal cell proliferation, which leads to tumor progression. The abnormal function of cell cycle regulators leads to uncontrolled cell proliferation, making it an attractive therapeutic target in cancer treatment. According to reports, in the rat liver fibrosis model, miR-34a regulates cell proliferation and apoptosis by inhibiting SIRT1 to activate p53. The miR-34a/SIRT1/p53 signaling pathway is activated in liver cells and is a therapeutic target for liver fibrosis. By a series of integrated analyses, miR-20a-5p and miR-200a-3p were screened out as the most potential modulator in liver fibrosis. miR-20a-5p was reported to inhibit proliferation and metastasis of HCC by targeting Runt-Related Transcription Factor 3 (RUNX3) and Hepatocyte Growth Factor (HGF). miR-200a-3p was reported to promote HCC proliferation and metastasis by targeting Zinc Finger E-Box Binding Homeobox 1 (ZEB1) and Cyclin Dependent Kinase 6 (CDK6) [31, 32]. Until now, there is no study showing the role of miR-200a-3 and miR-20a-5p in liver fibrosis, besides our previous study on miR-20a-5p/TGFBR2 axis [30]. In this study, we also identified many other potential target genes of miR-200a-3 and miR-20a-5p involved in liver fibrosis. For example, a case-control analysis showed that TP53 mutation reflects a moderate dietary exposure to aflatoxins leading to liver fibrosis [33], and p53 signal pathway is involved in liver fibrosis by inducing hepatocyte apoptosis [34]. It was reported EGFR played the key role in CCl_4_-induced liver fibrosis [35], if the expression of EGFR was inhibited, fibrosis and stellate cell activation were attenuated [36]. Another well-studied gene in liver fibrosis is PTEN, which was reported in the regulation of Kupffer cell activation [37]. The activation of the PTEN/p65 signaling pathway promoted liver fibrosis in nonalcoholic fatty liver disease [38].

This study has limitations. First, it is necessary to collect clinical samples to further study the expression levels of miR-20a-5p and miR-200a-3p. We plan to collect more samples in future studies to explore the correlation between the expression of miR-20a-5p, miR-200a-3p, and their target genes and the clinical parameters of patients with liver fibrosis. Second, it is necessary to further explore the regulation of miR-20a-5p and miR-200a-3p on the proliferation and cell cycle of liver fibrotic cells. Finally, we will further verify the regulation of miR-20a-5p and miR-200a-3p and their target genes on the progression of liver fibrosis.

In conclusion, this study identified 89 DEMs between fibrotic liver samples and normal liver samples by reanalyzing the GSE40744 data set. By constructing miRNA gene regulatory network and PPI network, further study miR-20a-5p and miR-200a-3p. Based on the GSE14323 database, our results showed that EGFR, STAT3, CTNNB1, and TP53 targeting by miR-200a-3p (upregulated) were significantly downregulated in fibrotic liver samples. The expression of PTEN, MYC, MAPK1, UBC, and CCND1 potentially regulated by miR-20a-5p (downregulated) was significantly overexpressed in fibrotic liver samples. In short, miR-200a-3 and miR-20a-5p were identified as the novel liver fibrosis-associated miRNAs, which may play key roles in liver fibrogenesis, suggesting the value of the further study.

## Figures and Tables

**Table 1:** Top ten upregulated differentially expressed miRNAs between liver fibrosis samples and normal liver samples.

miRNA name	logFC	*t*	*B*	*P* value	Adj. *P* value
hsa-mir-182-5p	3.58038	14.95512	29.6132	1.66*E*-17	1.23*E*-14
hsa-mir-200a-3p	3.166491	5.59633	4.6646	2.04*E*-06	5.46*E*-05
hsa-mir-200b-3p	2.934094	5.47836	4.3002	2.96*E*-06	7.56*E*-05
hsa-mir-155-5p	2.736842	16.72755	33.1346	4.06*E*-19	9.05*E*-16
hsa-mir-452-5p	2.634678	11.13642	20.7549	1.58*E*-13	8.77*E*-11
hsa-miR-29b-3p	2.524444	8.92977	14.7507	7.19*E*-11	1.00*E*-08
hsa-mir-31-5p	2.517047	6.38599	7.1117	1.68*E*-07	5.68*E*-06
hsa-mir-150-5p	2.345526	8.08317	12.2786	8.86*E*-10	7.04*E*-08
hsa-mir-708-5p	2.249942	5.40739	4.0813	3.70*E*-06	9.08*E*-05
hsa-mir-224-3p	2.218392	6.17261	6.4504	3.30*E*-07	1.10*E*-05

**Table 2:** Top ten downregulated differentially expressed miRNAs between liver fibrosis samples and normal liver samples.

miRNA name	logFC	*t*	*B*	*P* value	Adj. *P* value
hsa-mir-20a-5p	-2.064474	-3.68137	-1.0193	7.18*E*-04	8.15*E*-03
hsa-miR-194-3p	-1.806374	-7.87003	11.6433	1.69*E*-09	1.11*E*-07
hsa-mir-148a-3p	-1.792602	-8.298	12.9139	4.65*E*-10	4.50*E*-08
hsa-mir-1281	-1.75383	-4.87317	2.4477	1.97*E*-05	3.77*E*-04
hsa-mir-574-3p	-1.648655	-7.51855	10.5856	4.94*E*-09	2.89*E*-07
hsa-mir-1308	-1.64269	-3.68312	-1.0145	7.14*E*-04	8.15*E*-03
hsa-mir-572	-1.603509	-2.86486	-3.1265	6.76*E*-03	4.56*E*-02
hsa-mir-378-3p	-1.570848	-6.46778	7.3648	1.30*E*-07	4.60*E*-06
hsa-mir-130b-3p	-1.530614	-7.04856	9.1541	2.11*E*-08	1.07*E*-06
hsa-mir-193b-3p	-1.450292	-7.89223	11.7097	1.58*E*-09	1.10*E*-07

**Table 3:** Hub genes identified in the PPI interaction.

Gene symbol	Description	Degree	*P*1	*P*2	*P*3
Upregulated miRNA					
TP53	Tumor protein P53	136	+	+	+
EGFR	Epidermal growth factor receptor	106	—	—	—
PTEN	Phosphatase and tensin homolog	105	+	+	—
JUN	Jun proto-oncogene	93	+	—	—
VEGFA	Vascular endothelial growth factor A	89	—	—	—
KRAS	KRAS proto-oncogene, GTPase	87	+	—	—
STAT3	Signal transducer and activator of transcription 3	86	+	—	—
CTNNB1	Catenin beta 1	85	—	—	—
NOTCH1	Notch receptor 1	85	—	—	—
EP300	E1A binding protein p300	82	+	—	+
Downregulated miRNA					
TP53	Tumor protein P53	235	+	+	+
UBC	Ubiquitin C	191	—	—	—
RPS27A	Ribosomal protein S27a	184	—	—	—
MYC	MYC proto-oncogene, bHLH transcription factor	175	+	—	+
HSP90AA1	Heat shock protein 90 alpha family class A member 1	153	—	—	—
MAPK1	Mitogen-activated protein kinase 1	144	+	—	—
PTEN	Phosphatase and tensin homolog	134	+	+	—
CCND1	Cyclin D1	125	+	+	+
HSPA8	Heat shock protein family A (Hsp70) member 8	124	—	—	—
VEGFA	Vascular endothelial growth factor A	119	—	—	—

*P*1: hepatitis B; *P*2: p53 signaling pathway; *P*3: cell cycle. “+” and “-,” respectively, indicate genes can and cannot be found in corresponding KEGG pathways.

## Data Availability

The miRNA and mRNA expression data supporting this study are from previously reported studies and datasets, which have been cited. The processed data are available in the supplementary files or from the corresponding author upon request.

## Supplementary Materials

Supplementary 1 Table S1: list of predicted target genes.

Supplementary 2 Table S2: GO functions for target genes of the three upregulated miRNAs.

Supplementary 3 Table S3: GO functions for target genes of the three downregulated miRNAs.

Supplementary 4 Table S4: KEGG pathway enrichment analysis for selected DEMs.
