# Supplementary material for: Integrated MicroRNA Expression Profile Reveals Dysregulated miR-20a-5p and miR-200a-3p in Liver Fibrosis
Source: Biomed Res Int. 2021 Jun 8;2021:9583932. doi: 10.1155/2021/9583932 (PMC8218919; doi:10.1155/2021/9583932)
Supplement: Supplementary 1 — Table S1: list of predicted target genes. [file 9583932.f1.pdf]

miRTarBase ID	miRNA	Target Gene	Target Gene (Entrez Gene ID)	Experiments	Support Type	References (PMID)
MIRT000576	hsa-miR-182-5p	CDKN1A	1026	qRT-PCR//Luciferase reporter assay//Western blot	Non-Functional MTI	20190813
MIRT000671	hsa-miR-182-5p	FOXO3	2309	Luciferase reporter assay//Western blot	Functional MTI	19188590
MIRT001086	hsa-miR-182-5p	FOXO1	2308	qRT-PCR//Luciferase reporter assay//Western blot	Functional MTI	19574223
MIRT001222	hsa-miR-182-5p	RARG	5916	Luciferase reporter assay//Western blot	Functional MTI	19782699
MIRT001990	hsa-miR-182-5p	MITF	4286	Luciferase reporter assay	Functional MTI	17597072
MIRT001992	hsa-miR-182-5p	ADCY6	112	Luciferase reporter assay	Functional MTI	17597072
MIRT003601	hsa-miR-182-5p	EP300	2033	Microarray	Functional MTI (Weak)	19569050
MIRT005358	hsa-miR-182-5p	CLOCK	9575	Luciferase reporter assay//qRT-PCR	Functional MTI	20656788
MIRT005359	hsa-miR-182-5p	TSC22D3	1831	Luciferase reporter assay//qRT-PCR	Functional MTI	20656788
MIRT006273	hsa-miR-182-5p	CREB1	1385	GFP reporter assay	Functional MTI	22325466
MIRT006300	hsa-miR-182-5p	MTSS1	9788	Luciferase reporter assay	Functional MTI	22322863
MIRT006823	hsa-miR-182-5p	FGF9	2254	Luciferase reporter assay//Western blot	Functional MTI	22917588
MIRT006824	hsa-miR-182-5p	NTM	50863	Luciferase reporter assay//Western blot	Functional MTI	22917588
MIRT006978	hsa-miR-182-5p	CYLD	1540	Luciferase reporter assay//qRT-PCR//Western blot	Functional MTI	23006329
MIRT007063	hsa-miR-182-5p	BCL2	596	Flow//Luciferase reporter assay//qRT-PCR//Western blot	Functional MTI	22848417
MIRT007064	hsa-miR-182-5p	CCND2	894	Flow//Luciferase reporter assay//qRT-PCR//Western blot	Functional MTI	22848417
MIRT007155	hsa-miR-182-5p	PDCD4	27250	Luciferase reporter assay//qRT-PCR//Western blot	Functional MTI	23296900
MIRT007199	hsa-miR-182-5p	PFN1	5216	Luciferase reporter assay	Functional MTI	23430586
MIRT007226	hsa-miR-182-5p	SNAI2	6591	Luciferase reporter assay	Functional MTI	23354685
MIRT007293	hsa-miR-182-5p	RECK	8434	Luciferase reporter assay//qRT-PCR//Western blot	Functional MTI	23333633
MIRT007306	hsa-miR-182-5p	SMAD4	4089	Luciferase reporter assay	Functional MTI	23226455
MIRT007330	hsa-miR-182-5p	FOXF2	2295	Luciferase reporter assay	Functional MTI	23383207
MIRT025033	hsa-miR-182-5p	IGF1R	3480	PAR-CLIP	Functional MTI (Weak)	21572407
MIRT047175	hsa-miR-182-5p	CISD2	493856	CLASH	Functional MTI (Weak)	23622248
MIRT047176	hsa-miR-182-5p	BRIP1	83990	CLASH	Functional MTI (Weak)	23622248
MIRT047177	hsa-miR-182-5p	RBM12	10137	CLASH	Functional MTI (Weak)	23622248
MIRT047178	hsa-miR-182-5p	CBX6	23466	CLASH	Functional MTI (Weak)	23622248
MIRT047179	hsa-miR-182-5p	FMNL3	91010	CLASH	Functional MTI (Weak)	23622248
MIRT047180	hsa-miR-182-5p	RPL5	6125	CLASH	Functional MTI (Weak)	23622248
MIRT047181	hsa-miR-182-5p	RCC2	55920	CLASH	Functional MTI (Weak)	23622248
MIRT047182	hsa-miR-182-5p	GNL1	2794	CLASH	Functional MTI (Weak)	23622248
MIRT047183	hsa-miR-182-5p	SNRPA1	6627	CLASH	Functional MTI (Weak)	23622248
MIRT047184	hsa-miR-182-5p	C1orf174	339448	CLASH	Functional MTI (Weak)	23622248
MIRT047185	hsa-miR-182-5p	RLF	6018	CLASH	Functional MTI (Weak)	23622248
MIRT047186	hsa-miR-182-5p	CASC3	22794	CLASH	Functional MTI (Weak)	23622248
MIRT047187	hsa-miR-182-5p	FLOT1	10211	CLASH	Functional MTI (Weak)	23622248
MIRT047188	hsa-miR-182-5p	BAG1	573	CLASH	Functional MTI (Weak)	23622248
MIRT047189	hsa-miR-182-5p	RBM15B	29890	CLASH	Functional MTI (Weak)	23622248
MIRT047190	hsa-miR-182-5p	LSM14A	26065	CLASH	Functional MTI (Weak)	23622248
MIRT047191	hsa-miR-182-5p	RPS4X	6191	CLASH	Functional MTI (Weak)	23622248
MIRT047192	hsa-miR-182-5p	UBA1	7317	CLASH	Functional MTI (Weak)	23622248
MIRT047193	hsa-miR-182-5p	RPS3	6188	CLASH	Functional MTI (Weak)	23622248
MIRT047194	hsa-miR-182-5p	CENPS	378708	CLASH	Functional MTI (Weak)	23622248
MIRT047195	hsa-miR-182-5p	TMEM184C	55751	CLASH	Functional MTI (Weak)	23622248
MIRT047196	hsa-miR-182-5p	ETV3	2117	CLASH	Functional MTI (Weak)	23622248
MIRT047197	hsa-miR-182-5p	SEC61A2	55176	CLASH	Functional MTI (Weak)	23622248
MIRT047198	hsa-miR-182-5p	UBP1	7342	CLASH	Functional MTI (Weak)	23622248
MIRT047199	hsa-miR-182-5p	USP16	10600	CLASH	Functional MTI (Weak)	23622248
MIRT047200	hsa-miR-182-5p	ACVR1B	91	CLASH	Functional MTI (Weak)	23622248
MIRT047201	hsa-miR-182-5p	STAG2	10735	CLASH	Functional MTI (Weak)	23622248
MIRT047202	hsa-miR-182-5p	RAB3IP	117177	CLASH	Functional MTI (Weak)	23622248
MIRT047203	hsa-miR-182-5p	ATP5O	539	CLASH	Functional MTI (Weak)	23622248
MIRT047204	hsa-miR-182-5p	SLC25A14	9016	CLASH	Functional MTI (Weak)	23622248
MIRT047205	hsa-miR-182-5p	TTC28	23331	CLASH	Functional MTI (Weak)	23622248
MIRT047206	hsa-miR-182-5p	NUFIP2	57532	CLASH	Functional MTI (Weak)	23622248
MIRT047207	hsa-miR-182-5p	TNKS1BP1	85456	CLASH	Functional MTI (Weak)	23622248
MIRT047208	hsa-miR-182-5p	ACTN4	81	CLASH	Functional MTI (Weak)	23622248
MIRT047209	hsa-miR-182-5p	TUBA1B	10376	CLASH	Functional MTI (Weak)	23622248
MIRT047210	hsa-miR-182-5p	EVI5	7813	CLASH	Functional MTI (Weak)	23622248
MIRT047211	hsa-miR-182-5p	VPS51	738	CLASH	Functional MTI (Weak)	23622248
MIRT047212	hsa-miR-182-5p	ANO6	196527	CLASH	Functional MTI (Weak)	23622248
MIRT047213	hsa-miR-182-5p	PCOLCE2	26577	CLASH	Functional MTI (Weak)	23622248
MIRT047214	hsa-miR-182-5p	LBR	3930	CLASH	Functional MTI (Weak)	23622248
MIRT047215	hsa-miR-182-5p	PIK3C2A	5286	CLASH	Functional MTI (Weak)	23622248
MIRT047216	hsa-miR-182-5p	ERO1A	30001	CLASH	Functional MTI (Weak)	23622248
MIRT047217	hsa-miR-182-5p	PRKAA2	5563	CLASH	Functional MTI (Weak)	23622248
MIRT047218	hsa-miR-182-5p	ATP13A3	79572	CLASH	Functional MTI (Weak)	23622248
MIRT052907	hsa-miR-182-5p	PTEN	5728	Luciferase reporter assay//Microarray//qRT-PCR//Westerı	Functional MTI	24867318
MIRT053784	hsa-miR-182-5p	GSK3B	2932	Immunohistochemistry//Luciferase reporter assay//Microa	Functional MTI	24335145
MIRT054261	hsa-miR-182-5p	ZFAND4	93550	Immunofluorescence//Immunohistochemistry//Luciferase	Functional MTI	25682742
MIRT054501	hsa-miR-182-5p	BDNF	627	qRT-PCR//Western blotting	Functional MTI	23704927
MIRT054760	hsa-miR-182-5p	SATB2	23314	Luciferase reporter assay//QRTPCR//Western blot	Functional MTI	24884732
MIRT054831	hsa-miR-182-5p	CHL1	10752	Luciferase reporter assay/QRTPCR///Western blot	Functional MTI	24971532
MIRT054846	hsa-miR-182-5p	CADM1	23705	Luciferase reporter assay//qRT-PCR//Western blot	Functional MTI	24445397
MIRT054847	hsa-miR-182-5p	TP53INP1	94241	Luciferase reporter assay//qRT-PCR//Western blot	Functional MTI	24447717
MIRT180171	hsa-miR-182-5p	NOTCH2	4853	HITS-CLIP	Functional MTI (Weak)	23824327
MIRT203593	hsa-miR-182-5p	BRWD1	54014	HITS-CLIP	Functional MTI (Weak)	23313552
MIRT217011	hsa-miR-182-5p	ARRDC3	57561	Luciferase reporter assay	Functional MTI	27109471
MIRT265950	hsa-miR-182-5p	QSER1	79832	PAR-CLIP	Functional MTI (Weak)	21572407
MIRT353008	hsa-miR-182-5p	NUP50	10762	PAR-CLIP	Functional MTI (Weak)	20371350
MIRT437453	hsa-miR-182-5p	ATF1	466	Luciferase reporter assay	Functional MTI	23249749
MIRT437454	hsa-miR-182-5p	BARD1	580	Luciferase reporter assay	Functional MTI	23249749
MIRT437455	hsa-miR-182-5p	CREB5	9586	Luciferase reporter assay	Functional MTI	23249749
MIRT437456	hsa-miR-182-5p	RAD17	5884	Luciferase reporter assay	Functional MTI	23249749
MIRT437457	hsa-miR-182-5p	TP53BP1	7158	Luciferase reporter assay	Functional MTI	23249749
MIRT437458	hsa-miR-182-5p	CHEK2	11200	Luciferase reporter assay	Functional MTI	23249749
MIRT437459	hsa-miR-182-5p	CDKN1B	1027	Luciferase reporter assay	Functional MTI	23249749
MIRT437460	hsa-miR-182-5p	SMARCD3	6604	Luciferase reporter assay	Functional MTI	23249749
MIRT437477	hsa-miR-182-5p	TCEAL7	56849	Luciferase reporter assay//qRT-PCR//Western blot	Functional MTI	24021963
MIRT437908	hsa-miR-182-5p	FBXW7	55294	Luciferase reporter assay//Western blot	Functional MTI	25269767
MIRT437991	hsa-miR-182-5p	LRRC4	64101	qRT-PCR//Immunohistochemistry//Luciferase reporter as:	Functional MTI	24404152
MIRT438101	hsa-miR-182-5p	NDRG1	10397	Luciferase reporter assay	Functional MTI	23874837
MIRT438374	hsa-miR-182-5p	THBS1	7057	Luciferase reporter assay	Functional MTI	24053448
MIRT438756	hsa-miR-182-5p	ULBP2	80328	Luciferase reporter assay//qRT-PCR//Western blot	Functional MTI	23956116
MIRT442967	hsa-miR-182-5p	CTNNA3	29119	PAR-CLIP	Functional MTI (Weak)	22100165

MIRT447362	hsa-miR-182-5p	RASA2	5922	PAR-CLIP	Functional MTI (Weak)	22100165
MIRT452673	hsa-miR-182-5p	GPR156	165829	PAR-CLIP	Functional MTI (Weak)	23592263
MIRT453463	hsa-miR-182-5p	PITPNM3	83394	PAR-CLIP	Functional MTI (Weak)	23592263
MIRT454767	hsa-miR-182-5p	STOML3	161003	PAR-CLIP	Functional MTI (Weak)	23592263
MIRT468017	hsa-miR-182-5p	SIN3B	23309	PAR-CLIP	Functional MTI (Weak)	23592263
MIRT468529	hsa-miR-182-5p	SERPINH1	871	PAR-CLIP	Functional MTI (Weak)	23592263
MIRT470488	hsa-miR-182-5p	PPP1R11	6992	PAR-CLIP	Functional MTI (Weak)	23592263
MIRT474426	hsa-miR-182-5p	KLHL28	54813	PAR-CLIP	Functional MTI (Weak)	23592263
MIRT481285	hsa-miR-182-5p	ATXN1L	342371	PAR-CLIP	Functional MTI (Weak)	23592263
MIRT491596	hsa-miR-182-5p	CCL22	6367	PAR-CLIP	Functional MTI (Weak)	23592263
MIRT501916	hsa-miR-182-5p	MBD4	8930	PAR-CLIP	Functional MTI (Weak)	24398324
MIRT502391	hsa-miR-182-5p	CASTOR2	729438	PAR-CLIP	Functional MTI (Weak)	24398324
MIRT503220	hsa-miR-182-5p	ACER2	340485	PAR-CLIP	Functional MTI (Weak)	24398324
MIRT503542	hsa-miR-182-5p	RPL7L1	285855	PAR-CLIP	Functional MTI (Weak)	23446348
MIRT505720	hsa-miR-182-5p	SESN2	83667	PAR-CLIP	Functional MTI (Weak)	23446348
MIRT509890	hsa-miR-182-5p	RPS23	6228	PAR-CLIP	Functional MTI (Weak)	23446348
MIRT525375	hsa-miR-182-5p	SYNM	23336	PAR-CLIP	Functional MTI (Weak)	22012620
MIRT529422	hsa-miR-182-5p	MALT1	10892	PAR-CLIP	Functional MTI (Weak)	22012620
MIRT533379	hsa-miR-182-5p	UBE2D4	51619	PAR-CLIP	Functional MTI (Weak)	22012620
MIRT533467	hsa-miR-182-5p	TRIM71	131405	PAR-CLIP	Functional MTI (Weak)	22012620
MIRT533594	hsa-miR-182-5p	TNRC6A	27327	PAR-CLIP	Functional MTI (Weak)	22012620
MIRT535084	hsa-miR-182-5p	PODXL	5420	PAR-CLIP	Functional MTI (Weak)	22012620
MIRT541181	hsa-miR-182-5p	MORF4L1	10933	PAR-CLIP	Functional MTI (Weak)	21572407
MIRT549835	hsa-miR-182-5p	ECHDC1	55862	PAR-CLIP	Functional MTI (Weak)	21572407
MIRT550346	hsa-miR-182-5p	INCENP	3619	PAR-CLIP	Functional MTI (Weak)	21572407
MIRT553851	hsa-miR-182-5p	SYDE2	84144	PAR-CLIP	Functional MTI (Weak)	21572407
MIRT555374	hsa-miR-182-5p	PPP1R12A	4659	PAR-CLIP	Functional MTI (Weak)	21572407
MIRT555508	hsa-miR-182-5p	PNISR	25957	PAR-CLIP	Functional MTI (Weak)	21572407
MIRT555928	hsa-miR-182-5p	NUP43	348995	PAR-CLIP	Functional MTI (Weak)	21572407
MIRT556301	hsa-miR-182-5p	MAP3K3	4215	PAR-CLIP	Functional MTI (Weak)	21572407
MIRT557099	hsa-miR-182-5p	HOXA9	3205	PAR-CLIP	Functional MTI (Weak)	21572407
MIRT557242	hsa-miR-182-5p	CRAMP1	57585	PAR-CLIP	Functional MTI (Weak)	21572407
MIRT557937	hsa-miR-182-5p	MIGA1	374986	PAR-CLIP	Functional MTI (Weak)	21572407
MIRT558452	hsa-miR-182-5p	DDAH1	23576	PAR-CLIP	Functional MTI (Weak)	21572407
MIRT558720	hsa-miR-182-5p	CITED2	10370	PAR-CLIP	Functional MTI (Weak)	21572407
MIRT561179	hsa-miR-182-5p	TNFRSF10A	8797	PAR-CLIP	Functional MTI (Weak)	20371350
MIRT565334	hsa-miR-182-5p	TMEM170B	100113407	PAR-CLIP	Functional MTI (Weak)	20371350
MIRT571607	hsa-miR-182-5p	SYNGR2	9144	PAR-CLIP	Functional MTI (Weak)	20371350
MIRT573342	hsa-miR-182-5p	TUBD1	51174	PAR-CLIP	Functional MTI (Weak)	20371350
MIRT574601	hsa-miR-182-5p	LZIC	84328	PAR-CLIP	Functional MTI (Weak)	20371350
MIRT609631	hsa-miR-182-5p	PLEKHA8	84725	HITS-CLIP	Functional MTI (Weak)	23824327
MIRT612970	hsa-miR-182-5p	GID4	79018	HITS-CLIP	Functional MTI (Weak)	23824327
MIRT614906	hsa-miR-182-5p	NPTX1	4884	HITS-CLIP	Functional MTI (Weak)	23824327
MIRT614997	hsa-miR-182-5p	GABRB1	2560	HITS-CLIP	Functional MTI (Weak)	23824327
MIRT621334	hsa-miR-182-5p	SLC11A1	6556	HITS-CLIP	Functional MTI (Weak)	23824327
MIRT622018	hsa-miR-182-5p	STK17B	9262	HITS-CLIP	Functional MTI (Weak)	23824327
MIRT640564	hsa-miR-182-5p	CPE	1363	HITS-CLIP	Functional MTI (Weak)	23824327
MIRT644327	hsa-miR-182-5p	IPP	3652	HITS-CLIP	Functional MTI (Weak)	23824327
MIRT646586	hsa-miR-182-5p	ANKRD36	375248	HITS-CLIP	Functional MTI (Weak)	23824327
MIRT650974	hsa-miR-182-5p	ZNF831	128611	HITS-CLIP	Functional MTI (Weak)	23824327
MIRT652228	hsa-miR-182-5p	TRAPPC3L	100128327	HITS-CLIP	Functional MTI (Weak)	23824327
MIRT654840	hsa-miR-182-5p	PPM1L	151742	HITS-CLIP	Functional MTI (Weak)	23824327
MIRT656984	hsa-miR-182-5p	KDM5A	5927	HITS-CLIP	Functional MTI (Weak)	23824327
MIRT658776	hsa-miR-182-5p	EIF4EBP2	1979	HITS-CLIP	Functional MTI (Weak)	23824327
MIRT668093	hsa-miR-182-5p	GK5	256356	HITS-CLIP	Functional MTI (Weak)	23824327
MIRT668472	hsa-miR-182-5p	EXOSC2	23404	HITS-CLIP	Functional MTI (Weak)	23824327
MIRT687430	hsa-miR-182-5p	NR3C1	2908	HITS-CLIP	Functional MTI (Weak)	23313552
MIRT689956	hsa-miR-182-5p	ZNF185	7739	HITS-CLIP	Functional MTI (Weak)	23313552
MIRT690114	hsa-miR-182-5p	ZFAND1	79752	HITS-CLIP	Functional MTI (Weak)	23313552
MIRT697753	hsa-miR-182-5p	USP5	8078	HITS-CLIP	Functional MTI (Weak)	23313552
MIRT720750	hsa-miR-182-5p	FAM193A	8603	HITS-CLIP	Functional MTI (Weak)	19536157
MIRT732738	hsa-miR-182-5p	PDK4	5166	Luciferase reporter assay//qRT-PCR//Western blot	Functional MTI	27396327
MIRT733430	hsa-miR-182-5p	PLD1	5337	Immunofluorescence//Western blot	Functional MTI	26787840
MIRT734109	hsa-miR-182-5p	TRIM8	81603	Luciferase reporter assay//qRT-PCR//Western blot	Functional MTI	28280352
MIRT734439	hsa-miR-182-5p	TIAM1	7074	Luciferase reporter assay//qRT-PCR//Western blot	Functional MTI	25973950
MIRT734564	hsa-miR-182-5p	CUL5	8065	qRT-PCR	Functional MTI (Weak)	26847831
MIRT735083	hsa-miR-182-5p	PCDH8	5100	Luciferase reporter assay	Functional MTI	27246830
MIRT735126	hsa-miR-182-5p	NRN1	51299	Luciferase reporter assay	Functional MTI	26622652
MIRT735463	hsa-miR-182-5p	UQCRFS1	7386	Flow//Luciferase reporter assay//qRT-PCR//Western blot	Functional MTI	27476169
MIRT738068	hsa-miR-182-5p	AMMECR1L	83607	PAR-CLIP	Functional MTI (Weak)	26701625
MIRT738069	hsa-miR-182-5p	ATP8A2	51761	PAR-CLIP	Functional MTI (Weak)	26701625
MIRT738070	hsa-miR-182-5p	NFIC	4782	PAR-CLIP	Functional MTI (Weak)	26701625
MIRT738071	hsa-miR-182-5p	NPM1	4869	PAR-CLIP	Functional MTI (Weak)	26701625
MIRT738072	hsa-miR-182-5p	RAB5B	5869	PAR-CLIP	Functional MTI (Weak)	26701625
MIRT738073	hsa-miR-182-5p	S100A16	140576	PAR-CLIP	Functional MTI (Weak)	26701625
MIRT738074	hsa-miR-182-5p	SH3BGRL3	83442	PAR-CLIP	Functional MTI (Weak)	26701625
MIRT738075	hsa-miR-182-5p	ZFP36L1	677	PAR-CLIP	Functional MTI (Weak)	26701625
MIRT763002	hsa-miR-182-5p	IL31RA	133396	PAR-CLIP	Functional MTI (Weak)	27292025
MIRT763003	hsa-miR-182-5p	LOX	4015	PAR-CLIP	Functional MTI (Weak)	27292025
MIRT763004	hsa-miR-182-5p	PLAGL2	5326	PAR-CLIP	Functional MTI (Weak)	27292025
MIRT783958	hsa-miR-182-5p	LRTM1	57408	HITS-CLIP	Functional MTI (Weak)	27418678
MIRT783959	hsa-miR-182-5p	TMEM182	130827	HITS-CLIP	Functional MTI (Weak)	27418678
MIRT790132	hsa-miR-182-5p	PFKFB3	5209	HITS-CLIP	Functional MTI (Weak)	28735896
MIRT790133	hsa-miR-182-5p	RGS2	5997	HITS-CLIP	Functional MTI (Weak)	28735896
MIRT000204	hsa-miR-200a-3p	DLX5	1749	Luciferase reporter assay	Functional MTI	19454767
MIRT000730	hsa-miR-200a-3p	BAP1	8314	Microarray	Functional MTI (Weak)	17875710
MIRT000772	hsa-miR-200a-3p	G3BP2	9908	Microarray//Northern blot	Functional MTI (Weak)	16331254
MIRT000773	hsa-miR-200a-3p	PCDH8	5100	Microarray//Northern blot	Functional MTI (Weak)	16331254
MIRT000774	hsa-miR-200a-3p	RAB30	27314	Microarray//Northern blot	Functional MTI (Weak)	16331254
MIRT001035	hsa-miR-200a-3p	ZEB2	9839	Luciferase reporter assay//qRT-PCR	Functional MTI	18411277
MIRT001175	hsa-miR-200a-3p	CTNNB1	1499	qRT-PCR//Luciferase reporter assay//Western blot	Functional MTI	19703993
MIRT002480	hsa-miR-200a-3p	ZEB1	6935	Luciferase reporter assay//Microarray//Western blot	Functional MTI	18376396
MIRT002481	hsa-miR-200a-3p	GEMIN2	8487	Reporter assay	Functional MTI	18381893
MIRT004005	hsa-miR-200a-3p	PDCD4	27250	Microarray	Functional MTI (Weak)	17875710
MIRT004006	hsa-miR-200a-3p	GATA6	2627	Microarray	Functional MTI (Weak)	17875710
MIRT004007	hsa-miR-200a-3p	VCAM1	7412	Microarray	Functional MTI (Weak)	17875710

MIRT004400	hsa-miR-200a-3p	PSMD2	5708	Microarray//Northern blot	Functional MTI (Weak)	16331254
MIRT004524	hsa-miR-200a-3p	WASF3	10810	Luciferase reporter assay	Functional MTI	19801681
MIRT004549	hsa-miR-200a-3p	TFRC	7037	Northern blot	Functional MTI (Weak)	19135902
MIRT004622	hsa-miR-200a-3p	ZFPM2	23414	Luciferase reporter assay//Western blot//Reporter assay	Functional MTI	20005803
MIRT004867	hsa-miR-200a-3p	TRAPPC2B	10597	Luciferase reporter assay	Functional MTI	18835392
MIRT005752	hsa-miR-200a-3p	GDAP1	54332	Immunofluorescence//In situ hybridization//Luciferase rep	Functional MTI	20827281
MIRT006423	hsa-miR-200a-3p	MAPK14	1432	Luciferase reporter assay//Microarray//Western blot	Functional MTI	22101765
MIRT006665	hsa-miR-200a-3p	CCNE2	9134	Luciferase reporter assay	Functional MTI	22183793
MIRT006704	hsa-miR-200a-3p	KEAP1	9817	Luciferase reporter assay//qRT-PCR//Western blot	Functional MTI	21914820
MIRT006834	hsa-miR-200a-3p	SRF	6722	Immunoblot//Luciferase reporter assay//qRT-PCR	Functional MTI	22907787
MIRT007290	hsa-miR-200a-3p	SMAD2	4087	Luciferase reporter assay	Functional MTI	22020340
MIRT007291	hsa-miR-200a-3p	SMAD3	4088	Luciferase reporter assay	Functional MTI	22020340
MIRT007299	hsa-miR-200a-3p	YAP1	10413	Western blot	Functional MTI	23340296
MIRT020343	hsa-miR-200a-3p	KLHL20	27252	Reporter assay	Functional MTI	20005803
MIRT020345	hsa-miR-200a-3p	PTPRD	5789	Reporter assay	Functional MTI	20005803
MIRT020346	hsa-miR-200a-3p	ELMO2	63916	Reporter assay	Functional MTI	20005803
MIRT020347	hsa-miR-200a-3p	ERBIN	55914	Reporter assay	Functional MTI	20005803
MIRT020348	hsa-miR-200a-3p	WDR37	22884	Reporter assay	Functional MTI	20005803
MIRT020349	hsa-miR-200a-3p	VAC14	55697	Reporter assay	Functional MTI	20005803
MIRT020350	hsa-miR-200a-3p	SHC1	6464	Reporter assay	Functional MTI	20005803
MIRT020351	hsa-miR-200a-3p	TCF7L1	83439	Reporter assay	Functional MTI	20005803
MIRT020352	hsa-miR-200a-3p	RASSF2	9770	Reporter assay	Functional MTI	20005803
MIRT020353	hsa-miR-200a-3p	RIN2	54453	Reporter assay	Functional MTI	20005803
MIRT020354	hsa-miR-200a-3p	HOXB5	3215	Reporter assay	Functional MTI	20005803
MIRT020355	hsa-miR-200a-3p	SEPT7	989	Reporter assay	Functional MTI	20005803
MIRT020356	hsa-miR-200a-3p	KLF11	8462	Reporter assay	Functional MTI	20005803
MIRT052922	hsa-miR-200a-3p	MYB	4602	Flow//Luciferase reporter assay//qRT-PCR//Western blot	Functional MTI	22101269
MIRT053166	hsa-miR-200a-3p	TFAM	7019	Luciferase reporter assay//Western blot	Functional MTI	24684598
MIRT053353	hsa-miR-200a-3p	UBASH3B	84959	Immunohistochemistry//Luciferase reporter assay//Microa	Functional MTI	23784775
MIRT053604	hsa-miR-200a-3p	TP53	7157	Luciferase reporter assay//Western blot	Functional MTI	23144891
MIRT054264	hsa-miR-200a-3p	DNMT1	1786	ChIP-seq//qRT-PCR//Western blot	Functional MTI	25595591
MIRT054265	hsa-miR-200a-3p	EZH2	2146	ChIP-seq//qRT-PCR//Western blot	Functional MTI	25595591
MIRT054371	hsa-miR-200a-3p	PTEN	5728	qRT-PCR//Western blot	Functional MTI	24413994
MIRT054542	hsa-miR-200a-3p	HFE	3077	Western blot//qRT-PCR	Functional MTI	23708087
MIRT054545	hsa-miR-200a-3p	DLC1	10395	Western blot//qRT-PCR	Functional MTI	23708087
MIRT054553	hsa-miR-200a-3p	ATRX	546	Western blot//qRT-PCR	Functional MTI	23708087
MIRT061758	hsa-miR-200a-3p	PPP1R15B	84919	PAR-CLIP	Functional MTI (Weak)	23592263
MIRT072481	hsa-miR-200a-3p	RAB8B	51762	PAR-CLIP	Functional MTI (Weak)	20371350
MIRT078341	hsa-miR-200a-3p	MED13	9969	PAR-CLIP	Functional MTI (Weak)	24398324
MIRT099340	hsa-miR-200a-3p	QKI	9444	PAR-CLIP	Functional MTI (Weak)	20371350
MIRT117486	hsa-miR-200a-3p	ADNP2	22850	HITS-CLIP	Functional MTI (Weak)	23313552
MIRT126445	hsa-miR-200a-3p	ARL5B	221079	PAR-CLIP	Functional MTI (Weak)	23592263
MIRT127537	hsa-miR-200a-3p	TNKS2	80351	PAR-CLIP	Functional MTI (Weak)	21572407
MIRT135564	hsa-miR-200a-3p	SPRYD4	283377	PAR-CLIP	Functional MTI (Weak)	21572407
MIRT145857	hsa-miR-200a-3p	STAT3	6774	qRT-PCR	Functional MTI (Weak)	26535690
MIRT176071	hsa-miR-200a-3p	OGT	8473	PAR-CLIP	Functional MTI (Weak)	23446348
MIRT176153	hsa-miR-200a-3p	PGK1	5230	PAR-CLIP	Functional MTI (Weak)	22012620
MIRT227601	hsa-miR-200a-3p	ZBTB34	403341	PAR-CLIP	Functional MTI (Weak)	23592263
MIRT313184	hsa-miR-200a-3p	HNRNPAB	3182	PAR-CLIP	Functional MTI (Weak)	20371350
MIRT368862	hsa-miR-200a-3p	IPO5	3843	PAR-CLIP	Functional MTI (Weak)	23446348
MIRT437873	hsa-miR-200a-3p	AREG	374	ELISA//Immunohistochemistry//Immunoprecipitaion//Luc	Functional MTI	24762440
MIRT438100	hsa-miR-200a-3p	GRB2	2885	Luciferase reporter assay	Functional MTI	23874841
MIRT438675	hsa-miR-200a-3p	CDK6	1021	Luciferase reporter assay//qRT-PCR//Western blot	Functional MTI	24009066
MIRT441429	hsa-miR-200a-3p	STXBP2	6813	PAR-CLIP	Functional MTI (Weak)	22100165
MIRT441732	hsa-miR-200a-3p	SEPT8	23176	PAR-CLIP	Functional MTI (Weak)	22100165
MIRT442158	hsa-miR-200a-3p	DPY19L1	23333	PAR-CLIP	Functional MTI (Weak)	22100165
MIRT443389	hsa-miR-200a-3p	CHML	1122	PAR-CLIP	Functional MTI (Weak)	22100165
MIRT447629	hsa-miR-200a-3p	NDST4	64579	PAR-CLIP	Functional MTI (Weak)	22100165
MIRT452254	hsa-miR-200a-3p	TRAM1	23471	PAR-CLIP	Functional MTI (Weak)	23592263
MIRT455748	hsa-miR-200a-3p	YRDC	79693	PAR-CLIP	Functional MTI (Weak)	23592263
MIRT470195	hsa-miR-200a-3p	PSMD11	5717	PAR-CLIP	Functional MTI (Weak)	23592263
MIRT473463	hsa-miR-200a-3p	MCL1	4170	PAR-CLIP	Functional MTI (Weak)	23592263
MIRT478508	hsa-miR-200a-3p	CYP1B1	1545	PAR-CLIP	Functional MTI (Weak)	23592263
MIRT488715	hsa-miR-200a-3p	BICD2	23299	PAR-CLIP	Functional MTI (Weak)	23592263
MIRT498345	hsa-miR-200a-3p	PRELID2	153768	PAR-CLIP	Functional MTI (Weak)	22291592
MIRT500583	hsa-miR-200a-3p	USP53	54532	PAR-CLIP	Functional MTI (Weak)	24398324
MIRT502259	hsa-miR-200a-3p	HNRNPF	3185	PAR-CLIP	Functional MTI (Weak)	24398324
MIRT502310	hsa-miR-200a-3p	GNA13	10672	PAR-CLIP	Functional MTI (Weak)	24398324
MIRT502449	hsa-miR-200a-3p	FUT11	170384	PAR-CLIP	Functional MTI (Weak)	24398324
MIRT502530	hsa-miR-200a-3p	EPHA2	1969	PAR-CLIP	Functional MTI (Weak)	24398324
MIRT503111	hsa-miR-200a-3p	BRD3	8019	PAR-CLIP	Functional MTI (Weak)	24398324
MIRT505323	hsa-miR-200a-3p	TMOD3	29766	PAR-CLIP	Functional MTI (Weak)	23446348
MIRT506628	hsa-miR-200a-3p	MARCH6	10299	PAR-CLIP	Functional MTI (Weak)	23446348
MIRT506861	hsa-miR-200a-3p	KIAA1147	57189	PAR-CLIP	Functional MTI (Weak)	23446348
MIRT519589	hsa-miR-200a-3p	ZNF805	390980	PAR-CLIP	Functional MTI (Weak)	23446348
MIRT519813	hsa-miR-200a-3p	ZMAT3	64393	PAR-CLIP	Functional MTI (Weak)	23446348
MIRT529418	hsa-miR-200a-3p	MALT1	10892	PAR-CLIP	Functional MTI (Weak)	22012620
MIRT536478	hsa-miR-200a-3p	KIAA1549	57670	PAR-CLIP	Functional MTI (Weak)	22012620
MIRT537735	hsa-miR-200a-3p	ELAVL2	1993	PAR-CLIP	Functional MTI (Weak)	22012620
MIRT539039	hsa-miR-200a-3p	ATXN7L1	222255	PAR-CLIP	Functional MTI (Weak)	22012620
MIRT547112	hsa-miR-200a-3p	PIGW	284098	PAR-CLIP	Functional MTI (Weak)	21572407
MIRT548308	hsa-miR-200a-3p	EPHA7	2045	PAR-CLIP	Functional MTI (Weak)	21572407
MIRT549102	hsa-miR-200a-3p	C18orf25	147339	PAR-CLIP	Functional MTI (Weak)	21572407
MIRT554194	hsa-miR-200a-3p	SLC35D1	23169	PAR-CLIP	Functional MTI (Weak)	21572407
MIRT555677	hsa-miR-200a-3p	PGAM4	441531	PAR-CLIP	Functional MTI (Weak)	21572407
MIRT557413	hsa-miR-200a-3p	H2AFZ	3015	PAR-CLIP	Functional MTI (Weak)	21572407
MIRT559958	hsa-miR-200a-3p	TRMT112	51504	PAR-CLIP	Functional MTI (Weak)	20371350
MIRT564938	hsa-miR-200a-3p	XPOT	11260	PAR-CLIP	Functional MTI (Weak)	20371350
MIRT568097	hsa-miR-200a-3p	CDV3	55573	PAR-CLIP	Functional MTI (Weak)	20371350
MIRT573383	hsa-miR-200a-3p	KIAA1549L	25758	PAR-CLIP	Functional MTI (Weak)	20371350
MIRT614582	hsa-miR-200a-3p	LHX1	3975	HITS-CLIP	Functional MTI (Weak)	23824327
MIRT632224	hsa-miR-200a-3p	YME1L1	10730	HITS-CLIP	Functional MTI (Weak)	23824327
MIRT634842	hsa-miR-200a-3p	APOOL	139322	HITS-CLIP	Functional MTI (Weak)	23824327
MIRT639525	hsa-miR-200a-3p	ZNF621	285268	HITS-CLIP	Functional MTI (Weak)	23824327
MIRT645300	hsa-miR-200a-3p	ANGPTL7	10218	HITS-CLIP	Functional MTI (Weak)	23824327
MIRT654775	hsa-miR-200a-3p	PRKAA2	5563	HITS-CLIP	Functional MTI (Weak)	23824327

MIRT656541	hsa-miR-200a-3p	MACC1	346389	HITS-CLIP	Functional MTI (Weak)	23824327
MIRT663684	hsa-miR-200a-3p	CCDC18	343099	HITS-CLIP	Functional MTI (Weak)	23824327
MIRT682853	hsa-miR-200a-3p	DNAJC28	54943	HITS-CLIP	Functional MTI (Weak)	23706177
MIRT682940	hsa-miR-200a-3p	ZNF292	23036	HITS-CLIP	Functional MTI (Weak)	23706177
MIRT682959	hsa-miR-200a-3p	RPL12	6136	HITS-CLIP	Functional MTI (Weak)	23706177
MIRT683087	hsa-miR-200a-3p	A1BG	1	HITS-CLIP	Functional MTI (Weak)	23706177
MIRT692251	hsa-miR-200a-3p	POLR3F	10621	HITS-CLIP	Functional MTI (Weak)	23313552
MIRT696502	hsa-miR-200a-3p	COX6B1	1340	HITS-CLIP	Functional MTI (Weak)	23313552
MIRT696748	hsa-miR-200a-3p	RBM28	55131	HITS-CLIP	Functional MTI (Weak)	23313552
MIRT702209	hsa-miR-200a-3p	LPP	4026	HITS-CLIP	Functional MTI (Weak)	23313552
MIRT708614	hsa-miR-200a-3p	CCDC71L	168455	HITS-CLIP	Functional MTI (Weak)	19536157
MIRT709036	hsa-miR-200a-3p	TROVE2	6738	HITS-CLIP	Functional MTI (Weak)	19536157
MIRT710918	hsa-miR-200a-3p	BICRAL	23506	HITS-CLIP	Functional MTI (Weak)	19536157
MIRT713191	hsa-miR-200a-3p	ERO1A	30001	HITS-CLIP	Functional MTI (Weak)	19536157
MIRT714497	hsa-miR-200a-3p	HSPA4	3308	HITS-CLIP	Functional MTI (Weak)	19536157
MIRT718596	hsa-miR-200a-3p	SCD5	79966	HITS-CLIP	Functional MTI (Weak)	19536157
MIRT723110	hsa-miR-200a-3p	CLDND1	56650	HITS-CLIP	Functional MTI (Weak)	19536157
MIRT723914	hsa-miR-200a-3p	PEX11A	8800	HITS-CLIP	Functional MTI (Weak)	19536157
MIRT725579	hsa-miR-200a-3p	CELF1	10658	HITS-CLIP	Functional MTI (Weak)	19536157
MIRT731049	hsa-miR-200a-3p	MYH10	4628	Luciferase reporter assay//Western blot	Functional MTI	24858044
MIRT731785	hsa-miR-200a-3p	SPAG9	9043	Luciferase reporter assay//qRT-PCR//Western blot	Functional MTI	26797273
MIRT732234	hsa-miR-200a-3p	TGFB2	7042	Luciferase reporter assay//qRT-PCR//Western blot	Functional MTI	27267902
MIRT732658	hsa-miR-200a-3p	foxg1a	30274	Western blot	Functional MTI	25937343
MIRT732985	hsa-miR-200a-3p	DEK	7913	Immunohistochemistry//Luciferase reporter assay//qRT-P(	Functional MTI	26947878
MIRT733513	hsa-miR-200a-3p	THRB	7068	Luciferase reporter assay//Next Generation Sequencing (N	Functional MTI	27734462
MIRT733772	hsa-miR-200a-3p	MFF	56947	Immunofluorescence//Western blot	Functional MTI	28148392
MIRT734407	hsa-miR-200a-3p	GJA1	2697	Luciferase reporter assay//qRT-PCR//Western blot	Functional MTI	26283635
MIRT734700	hsa-miR-200a-3p	HGF	3082	qRT-PCR//Western blot	Functional MTI	27374174
MIRT734965	hsa-miR-200a-3p	DICER1	23405	qRT-PCR	Functional MTI (Weak)	25768283
MIRT735206	hsa-miR-200a-3p	FOXA2	3170	Luciferase reporter assay//qRT-PCR//Western blot	Functional MTI	28367241
MIRT735283	hsa-miR-200a-3p	HMGB1	3146	ELISA//Luciferase reporter assay//qRT-PCR//Western blc	Functional MTI	28403886
MIRT735284	hsa-miR-200a-3p	TP73-AS1	57212	ELISA//Luciferase reporter assay//qRT-PCR//Western blc	Functional MTI	28403886
MIRT735329	hsa-miR-200a-3p	MALAT1	378938	Luciferase reporter assay//qRT-PCR	Functional MTI	26461224
MIRT735389	hsa-miR-200a-3p	MNX1	3110	Luciferase reporter assay//qRT-PCR//Western blot	Functional MTI	26801823
MIRT735444	hsa-miR-200a-3p	EGFR	1956	Western blot	Functional MTI	28057023
MIRT738760	hsa-miR-200a-3p	C16orf58	64755	PAR-CLIP	Functional MTI (Weak)	26701625
MIRT738761	hsa-miR-200a-3p	IRF2BPL	64207	PAR-CLIP	Functional MTI (Weak)	26701625
MIRT763653	hsa-miR-200a-3p	KLF12	11278	PAR-CLIP	Functional MTI (Weak)	27292025
MIRT763654	hsa-miR-200a-3p	MDM4	4194	PAR-CLIP	Functional MTI (Weak)	27292025
MIRT763655	hsa-miR-200a-3p	PHB2	11331	PAR-CLIP	Functional MTI (Weak)	27292025
MIRT763656	hsa-miR-200a-3p	RAP2C	57826	PAR-CLIP	Functional MTI (Weak)	27292025
MIRT763657	hsa-miR-200a-3p	TNFRSF10B	8795	PAR-CLIP	Functional MTI (Weak)	27292025
MIRT790156	hsa-miR-200a-3p	UQCRFS1	7386	HITS-CLIP	Functional MTI (Weak)	28735896
MIRT000198	hsa-miR-200b-3p	PTPN12	5782	Northern blot//qRT-PCR//Western blot	Functional MTI	16762633
MIRT000723	hsa-miR-200b-3p	ZEB2	9839	Luciferase reporter assay//qRT-PCR	Functional MTI	18411277
MIRT000724	hsa-miR-200b-3p	BAP1	8314	Microarray	Functional MTI (Weak)	17875710
MIRT002483	hsa-miR-200b-3p	ZEB1	6935	Luciferase reporter assay//qRT-PCR//Reporter assay	Functional MTI	18411277
MIRT002987	hsa-miR-200b-3p	RERE	473	Luciferase reporter assay	Functional MTI	17923093
MIRT003599	hsa-miR-200b-3p	EP300	2033	Microarray	Functional MTI (Weak)	19569050
MIRT003783	hsa-miR-200b-3p	ETS1	2113	Immunocytochemistry//Luciferase reporter assay//qRT-P(	Functional MTI	21081489
MIRT004008	hsa-miR-200b-3p	GATA4	2626	Microarray	Functional MTI (Weak)	17875710
MIRT004009	hsa-miR-200b-3p	FN1	2335	Microarray	Functional MTI (Weak)	17875710
MIRT004296	hsa-miR-200b-3p	WASF3	10810	Luciferase reporter assay//qRT-PCR	Functional MTI	19801681
MIRT004623	hsa-miR-200b-3p	ZFPM2	23414	Luciferase reporter assay//Western blot//Reporter assay	Functional MTI	20005803
MIRT005035	hsa-miR-200b-3p	MATR3	9782	Luciferase reporter assay//Microarray//qRT-PCR	Functional MTI	19849700
MIRT006163	hsa-miR-200b-3p	RNF2	6045	Luciferase reporter assay//Microarray//qRT-PCR//Westerı	Functional MTI	21840484
MIRT006165	hsa-miR-200b-3p	BMI1	648	Luciferase reporter assay//Microarray//qRT-PCR//Westerı	Functional MTI	21840484
MIRT006298	hsa-miR-200b-3p	E2F3	1871	Flow//Luciferase reporter assay//Western blot	Functional MTI	22139708
MIRT006440	hsa-miR-200b-3p	VEGFA	7422	Luciferase reporter assay//Western blot	Functional MTI	21544626
MIRT006441	hsa-miR-200b-3p	FLT1	2321	Luciferase reporter assay//Western blot	Functional MTI	21544626
MIRT006442	hsa-miR-200b-3p	KDR	3791	Luciferase reporter assay//Western blot	Functional MTI	21544626
MIRT006511	hsa-miR-200b-3p	RND3	390	Luciferase reporter assay	Functional MTI	20683643
MIRT006666	hsa-miR-200b-3p	CCNE2	9134	Luciferase reporter assay	Functional MTI	22183793
MIRT006670	hsa-miR-200b-3p	BCL2	596	Luciferase reporter assay//qRT-PCR//Western blot	Functional MTI	21993663
MIRT006671	hsa-miR-200b-3p	XIAP	331	Luciferase reporter assay//qRT-PCR//Western blot	Functional MTI	21993663
MIRT007322	hsa-miR-200b-3p	SMAD2	4087	Luciferase reporter assay	Functional MTI	23492772
MIRT007342	hsa-miR-200b-3p	CREB1	1385	Luciferase reporter assay//qRT-PCR//Western blot	Functional MTI	23543137
MIRT024121	hsa-miR-200b-3p	KLHL20	27252	Reporter assay	Functional MTI	20005803
MIRT024122	hsa-miR-200b-3p	ELMO2	63916	Reporter assay	Functional MTI	20005803
MIRT024123	hsa-miR-200b-3p	PTPRD	5789	Reporter assay	Functional MTI	20005803
MIRT024124	hsa-miR-200b-3p	ERRFI1	54206	PAR-CLIP	Functional MTI (Weak)	26701625
MIRT024125	hsa-miR-200b-3p	ERBIN	55914	Reporter assay	Functional MTI	20005803
MIRT024126	hsa-miR-200b-3p	WDR37	22884	Reporter assay	Functional MTI	20005803
MIRT024127	hsa-miR-200b-3p	TCF7L1	83439	Reporter assay	Functional MTI	20005803
MIRT024128	hsa-miR-200b-3p	VAC14	55697	Reporter assay	Functional MTI	20005803
MIRT024129	hsa-miR-200b-3p	HOXB5	3215	Reporter assay	Functional MTI	20005803
MIRT024130	hsa-miR-200b-3p	RIN2	54453	Reporter assay	Functional MTI	20005803
MIRT024131	hsa-miR-200b-3p	RASSF2	9770	Reporter assay	Functional MTI	20005803
MIRT024132	hsa-miR-200b-3p	KLF11	8462	Reporter assay	Functional MTI	20005803
MIRT024133	hsa-miR-200b-3p	SEPT7	989	Reporter assay	Functional MTI	20005803
MIRT024134	hsa-miR-200b-3p	SHC1	6464	Reporter assay	Functional MTI	20005803
MIRT046569	hsa-miR-200b-3p	SUPV3L1	6832	CLASH	Functional MTI (Weak)	23622248
MIRT052923	hsa-miR-200b-3p	MYB	4602	Flow//Luciferase reporter assay//qRT-PCR//Western blot	Functional MTI	22101269
MIRT053342	hsa-miR-200b-3p	QRSL1	55278	Luciferase reporter assay//qRT-PCR//Western blot	Functional MTI	22499991
MIRT053529	hsa-miR-200b-3p	SUZ12	23512	ChIP-seq//Flow//Luciferase reporter assay//Western blot	Functional MTI	23735840
MIRT053535	hsa-miR-200b-3p	WNT1	7471	Immunohistochemistry//Luciferase reporter assay//Microa	Functional MTI	23851184
MIRT053568	hsa-miR-200b-3p	DNMT3A	1788	In situ hybridization//Luciferase reporter assay//qRT-PCR	Functional MTI	23995857
MIRT053569	hsa-miR-200b-3p	DNMT3B	1789	In situ hybridization//Luciferase reporter assay//qRT-PCR	Functional MTI	23995857
MIRT053570	hsa-miR-200b-3p	SP1	6667	In situ hybridization//Luciferase reporter assay//qRT-PCR	Functional MTI	23995857
MIRT053625	hsa-miR-200b-3p	MSN	4478	Immunofluorescence//Immunohistochemistry//Luciferase	Functional MTI	24037528
MIRT054266	hsa-miR-200b-3p	DNMT1	1786	ChIP-seq//qRT-PCR//Western blot	Functional MTI	25595591
MIRT054267	hsa-miR-200b-3p	EZH2	2146	ChIP-seq//qRT-PCR//Western blot	Functional MTI	25595591
MIRT054541	hsa-miR-200b-3p	HFE	3077	Western blot//qRT-PCR	Functional MTI	23708087
MIRT054548	hsa-miR-200b-3p	DLC1	10395	Western blot//qRT-PCR	Functional MTI	23708087
MIRT054550	hsa-miR-200b-3p	HNRNPA3	220988	Western blot//qRT-PCR	Functional MTI	23708087
MIRT054683	hsa-miR-200b-3p	FERMT2	10979	Immunofluorescence//Immunohistochemistry//Luciferase	Functional MTI	24064224

MIRT054844	hsa-miR-200b-3p	RAB21	23011	Luciferase reporter assay//qRT-PCR//Western blot	Functional MTI	24447584
MIRT054845	hsa-miR-200b-3p	RAB3B	5865	Luciferase reporter assay//qRT-PCR//Western blot	Functional MTI	24447584
MIRT054885	hsa-miR-200b-3p	RAB18	22931	Luciferase reporter assay//qRT-PCR//Western blot	Functional MTI	24477653
MIRT054886	hsa-miR-200b-3p	RAB23	51715	Luciferase reporter assay//qRT-PCR//Western blot	Functional MTI	24477653
MIRT054917	hsa-miR-200b-3p	OXR1	55074	Luciferase reporter assay//qRT-PCR//Western blot	Functional MTI	23404117
MIRT063868	hsa-miR-200b-3p	RASSF8	11228	PAR-CLIP	Functional MTI (Weak)	23592263
MIRT080631	hsa-miR-200b-3p	PMAIP1	5366	PAR-CLIP	Functional MTI (Weak)	21572407
MIRT100486	hsa-miR-200b-3p	UHRF1BP1	54887	PAR-CLIP	Functional MTI (Weak)	21572407
MIRT106295	hsa-miR-200b-3p	ZFHX4	79776	PAR-CLIP	Functional MTI (Weak)	23446348
MIRT108446	hsa-miR-200b-3p	ACSL4	2182	PAR-CLIP	Functional MTI (Weak)	22100165
MIRT143030	hsa-miR-200b-3p	SHCBP1	79801	PAR-CLIP	Functional MTI (Weak)	22012620
MIRT157710	hsa-miR-200b-3p	CAB39	51719	PAR-CLIP	Functional MTI (Weak)	24398324
MIRT159114	hsa-miR-200b-3p	NRBP1	29959	PAR-CLIP	Functional MTI (Weak)	24398324
MIRT204208	hsa-miR-200b-3p	ATP5G3	518	PAR-CLIP	Functional MTI (Weak)	21572407
MIRT209178	hsa-miR-200b-3p	ZMAT3	64393	PAR-CLIP	Functional MTI (Weak)	24398324
MIRT212861	hsa-miR-200b-3p	N4BP2	55728	PAR-CLIP	Functional MTI (Weak)	22100165
MIRT218291	hsa-miR-200b-3p	QKI	9444	PAR-CLIP	Functional MTI (Weak)	24398324
MIRT243811	hsa-miR-200b-3p	ANKRD33B	651746	PAR-CLIP	Functional MTI (Weak)	23592263
MIRT244404	hsa-miR-200b-3p	DNAJB6	10049	PAR-CLIP	Functional MTI (Weak)	24398324
MIRT245588	hsa-miR-200b-3p	DDIT4	54541	PAR-CLIP	Functional MTI (Weak)	23446348
MIRT271934	hsa-miR-200b-3p	KLHL42	57542	PAR-CLIP	Functional MTI (Weak)	23446348
MIRT278144	hsa-miR-200b-3p	FOXG1	2290	Luciferase reporter assay	Functional MTI	27044840
MIRT296344	hsa-miR-200b-3p	PARD6B	84612	PAR-CLIP	Functional MTI (Weak)	22012620
MIRT319179	hsa-miR-200b-3p	DNAJB9	4189	PAR-CLIP	Functional MTI (Weak)	24398324
MIRT329852	hsa-miR-200b-3p	SHOC2	8036	PAR-CLIP	Functional MTI (Weak)	20371350
MIRT344584	hsa-miR-200b-3p	NUFIP2	57532	PAR-CLIP	Functional MTI (Weak)	20371350
MIRT437862	hsa-miR-200b-3p	NOTCH1	4851	Luciferase reporter assay//qRT-PCR//Western blot	Functional MTI	23955600
MIRT437874	hsa-miR-200b-3p	BTC	685	ELISA//Immunohistochemistry//Immunoprecipitaion//Luc	Functional MTI	24762440
MIRT437988	hsa-miR-200b-3p	CDKN1B	1027	PAR-CLIP	Functional MTI (Weak)	20371350
MIRT438051	hsa-miR-200b-3p	LOX	4015	Luciferase reporter assay//qRT-PCR//Western blot	Functional MTI	23975428
MIRT438165	hsa-miR-200b-3p	PHLPP1	23239	Luciferase reporter assay//Northern blot//qRT-PCR//West	Functional MTI	24337069
MIRT438166	hsa-miR-200b-3p	SEC23A	10484	PAR-CLIP	Functional MTI (Weak)	23592263
MIRT438204	hsa-miR-200b-3p	ROCK2	9475	Luciferase reporter assay	Functional MTI	24169343
MIRT438291	hsa-miR-200b-3p	PIN1	5300	Luciferase reporter assay	Functional MTI	24072701
MIRT438476	hsa-miR-200b-3p	DDX53	168400	Luciferase reporter assay	Functional MTI	24174534
MIRT444900	hsa-miR-200b-3p	PHF14	9678	PAR-CLIP	Functional MTI (Weak)	22100165
MIRT447579	hsa-miR-200b-3p	CDC14B	8555	PAR-CLIP	Functional MTI (Weak)	22100165
MIRT449890	hsa-miR-200b-3p	ZNF621	285268	PAR-CLIP	Functional MTI (Weak)	22100165
MIRT450413	hsa-miR-200b-3p	CACNG8	59283	PAR-CLIP	Functional MTI (Weak)	22100165
MIRT451990	hsa-miR-200b-3p	FKBP5	2289	PAR-CLIP	Functional MTI (Weak)	23592263
MIRT473906	hsa-miR-200b-3p	M6PR	4074	PAR-CLIP	Functional MTI (Weak)	23592263
MIRT474749	hsa-miR-200b-3p	KIF13A	63971	PAR-CLIP	Functional MTI (Weak)	23592263
MIRT478184	hsa-miR-200b-3p	DENND5B	160518	PAR-CLIP	Functional MTI (Weak)	23592263
MIRT488806	hsa-miR-200b-3p	MALT1	10892	PAR-CLIP	Functional MTI (Weak)	23592263
MIRT497728	hsa-miR-200b-3p	CYP1A1	1543	PAR-CLIP	Functional MTI (Weak)	22291592
MIRT498688	hsa-miR-200b-3p	SERPINH1	871	PAR-CLIP	Functional MTI (Weak)	24398324
MIRT499209	hsa-miR-200b-3p	TJAP1	93643	PAR-CLIP	Functional MTI (Weak)	24398324
MIRT499298	hsa-miR-200b-3p	TPD52L1	7164	PAR-CLIP	Functional MTI (Weak)	24398324
MIRT499786	hsa-miR-200b-3p	CASP2	835	PAR-CLIP	Functional MTI (Weak)	24398324
MIRT499851	hsa-miR-200b-3p	AVPR1A	552	PAR-CLIP	Functional MTI (Weak)	24398324
MIRT499929	hsa-miR-200b-3p	GPX8	493869	PAR-CLIP	Functional MTI (Weak)	24398324
MIRT499960	hsa-miR-200b-3p	ABI2	10152	PAR-CLIP	Functional MTI (Weak)	24398324
MIRT500165	hsa-miR-200b-3p	CLEC2D	29121	PAR-CLIP	Functional MTI (Weak)	24398324
MIRT500609	hsa-miR-200b-3p	UBE2D1	7321	PAR-CLIP	Functional MTI (Weak)	24398324
MIRT500743	hsa-miR-200b-3p	TP53INP1	94241	PAR-CLIP	Functional MTI (Weak)	24398324
MIRT500827	hsa-miR-200b-3p	ELOC	6921	PAR-CLIP	Functional MTI (Weak)	24398324
MIRT500837	hsa-miR-200b-3p	TBK1	29110	PAR-CLIP	Functional MTI (Weak)	24398324
MIRT501488	hsa-miR-200b-3p	PRRG4	79056	PAR-CLIP	Functional MTI (Weak)	24398324
MIRT501594	hsa-miR-200b-3p	PLCG1	5335	PAR-CLIP	Functional MTI (Weak)	24398324
MIRT501842	hsa-miR-200b-3p	NCOA2	10499	PAR-CLIP	Functional MTI (Weak)	24398324
MIRT502084	hsa-miR-200b-3p	KRAS	3845	PAR-CLIP	Functional MTI (Weak)	24398324
MIRT502178	hsa-miR-200b-3p	JUN	3725	PAR-CLIP	Functional MTI (Weak)	24398324
MIRT502837	hsa-miR-200b-3p	CELF1	10658	PAR-CLIP	Functional MTI (Weak)	24398324
MIRT502959	hsa-miR-200b-3p	CCNT2	905	PAR-CLIP	Functional MTI (Weak)	24398324
MIRT503131	hsa-miR-200b-3p	BAG4	9530	PAR-CLIP	Functional MTI (Weak)	24398324
MIRT505280	hsa-miR-200b-3p	TUBB2A	7280	PAR-CLIP	Functional MTI (Weak)	23446348
MIRT505430	hsa-miR-200b-3p	TCF7L2	6934	PAR-CLIP	Functional MTI (Weak)	23446348
MIRT511849	hsa-miR-200b-3p	GPATCH8	23131	PAR-CLIP	Functional MTI (Weak)	23446348
MIRT513408	hsa-miR-200b-3p	LMNB1	4001	PAR-CLIP	Functional MTI (Weak)	23446348
MIRT528183	hsa-miR-200b-3p	C6orf47	57827	PAR-CLIP	Functional MTI (Weak)	22012620
MIRT530669	hsa-miR-200b-3p	TRIM56	81844	PAR-CLIP	Functional MTI (Weak)	22012620
MIRT530836	hsa-miR-200b-3p	MAPK7	5598	PAR-CLIP	Functional MTI (Weak)	22012620
MIRT531134	hsa-miR-200b-3p	CLPB	81570	PAR-CLIP	Functional MTI (Weak)	22012620
MIRT531393	hsa-miR-200b-3p	TXNDC16	57544	PAR-CLIP	Functional MTI (Weak)	22012620
MIRT533348	hsa-miR-200b-3p	UNC119B	84747	PAR-CLIP	Functional MTI (Weak)	22012620
MIRT533791	hsa-miR-200b-3p	TMEM119	338773	PAR-CLIP	Functional MTI (Weak)	22012620
MIRT533975	hsa-miR-200b-3p	TAF1D	79101	PAR-CLIP	Functional MTI (Weak)	22012620
MIRT535530	hsa-miR-200b-3p	PAK2	5062	PAR-CLIP	Functional MTI (Weak)	22012620
MIRT535670	hsa-miR-200b-3p	NLGN4X	57502	PAR-CLIP	Functional MTI (Weak)	22012620
MIRT537615	hsa-miR-200b-3p	ERMP1	79956	PAR-CLIP	Functional MTI (Weak)	22012620
MIRT540709	hsa-miR-200b-3p	PDPK1	5170	PAR-CLIP	Functional MTI (Weak)	21572407
MIRT549052	hsa-miR-200b-3p	CAMSAP2	23271	PAR-CLIP	Functional MTI (Weak)	21572407
MIRT557648	hsa-miR-200b-3p	GATA6	2627	PAR-CLIP	Functional MTI (Weak)	21572407
MIRT557786	hsa-miR-200b-3p	FRS2	10818	PAR-CLIP	Functional MTI (Weak)	21572407
MIRT558564	hsa-miR-200b-3p	CRLF3	51379	PAR-CLIP	Functional MTI (Weak)	21572407
MIRT558829	hsa-miR-200b-3p	CDCA4	55038	PAR-CLIP	Functional MTI (Weak)	21572407
MIRT564438	hsa-miR-200b-3p	YEATS4	8089	PAR-CLIP	Functional MTI (Weak)	20371350
MIRT566768	hsa-miR-200b-3p	MLLT10	8028	PAR-CLIP	Functional MTI (Weak)	20371350
MIRT568295	hsa-miR-200b-3p	BBX	56987	PAR-CLIP	Functional MTI (Weak)	20371350
MIRT570299	hsa-miR-200b-3p	ARPC3	10094	PAR-CLIP	Functional MTI (Weak)	20371350
MIRT571758	hsa-miR-200b-3p	PSD3	23362	PAR-CLIP	Functional MTI (Weak)	20371350
MIRT573139	hsa-miR-200b-3p	ABT1	29777	PAR-CLIP	Functional MTI (Weak)	20371350
MIRT616923	hsa-miR-200b-3p	PPM1H	57460	HITS-CLIP	Functional MTI (Weak)	23824327
MIRT640091	hsa-miR-200b-3p	INO80D	54891	HITS-CLIP	Functional MTI (Weak)	23824327
MIRT640649	hsa-miR-200b-3p	IGF2	3481	HITS-CLIP	Functional MTI (Weak)	23824327
MIRT661419	hsa-miR-200b-3p	FAM216B	144809	HITS-CLIP	Functional MTI (Weak)	23824327

MIRT664728	hsa-miR-200b-3p	PTPN4	5775	HITS-CLIP	Functional MTI (Weak)	23824327
MIRT686617	hsa-miR-200b-3p	TMEM70	54968	HITS-CLIP	Functional MTI (Weak)	23313552
MIRT686764	hsa-miR-200b-3p	STX16	8675	HITS-CLIP	Functional MTI (Weak)	23313552
MIRT699807	hsa-miR-200b-3p	SEC24A	10802	HITS-CLIP	Functional MTI (Weak)	23313552
MIRT701748	hsa-miR-200b-3p	MTDH	92140	HITS-CLIP	Functional MTI (Weak)	23313552
MIRT711054	hsa-miR-200b-3p	IRS1	3667	HITS-CLIP	Functional MTI (Weak)	19536157
MIRT721943	hsa-miR-200b-3p	NGRN	51335	HITS-CLIP	Functional MTI (Weak)	19536157
MIRT723590	hsa-miR-200b-3p	PABPC1	26986	HITS-CLIP	Functional MTI (Weak)	19536157
MIRT731596	hsa-miR-200b-3p	RHOA	387	Luciferase reporter assay	Functional MTI	26903801
MIRT733087	hsa-miR-200b-3p	ADAM12	8038	Luciferase reporter assay//qRT-PCR//Western blot	Functional MTI	25886595
MIRT733564	hsa-miR-200b-3p	ETV5	2119	qRT-PCR//Western blot	Functional MTI	27276064
MIRT733587	hsa-miR-200b-3p	ATG12	9140	//qRT-PCR//Western blot//Luciferase reporter assay	Functional MTI	26416454
MIRT733739	hsa-miR-200b-3p	CD274	29126	Flow//Immunofluorescence//Luciferase reporter assay//qR	Functional MTI	28056089
MIRT733756	hsa-miR-200b-3p	IKBKB	3551	Luciferase reporter assay//qRT-PCR//Western blot	Functional MTI	26433127
MIRT734229	hsa-miR-200b-3p	LDHA	3939	Luciferase reporter assay//qRT-PCR//Western blot	Functional MTI	27374173
MIRT734346	hsa-miR-200b-3p	CRKL	1399	Luciferase reporter assay//qRT-PCR//Western blot	Functional MTI	26079153
MIRT735330	hsa-miR-200b-3p	MALAT1	378938	Luciferase reporter assay//qRT-PCR	Functional MTI	26461224
MIRT735429	hsa-miR-200b-3p	FSCN1	6624	Luciferase reporter assay//qRT-PCR//Western blot	Functional MTI	27356635
MIRT738763	hsa-miR-200b-3p	ARHGDIA	396	PAR-CLIP	Functional MTI (Weak)	26701625
MIRT738764	hsa-miR-200b-3p	DCBLD2	131566	PAR-CLIP	Functional MTI (Weak)	26701625
MIRT738765	hsa-miR-200b-3p	GOT1	2805	PAR-CLIP	Functional MTI (Weak)	26701625
MIRT738766	hsa-miR-200b-3p	MKNK2	2872	PAR-CLIP	Functional MTI (Weak)	26701625
MIRT738767	hsa-miR-200b-3p	PKD1	5310	PAR-CLIP	Functional MTI (Weak)	26701625
MIRT738768	hsa-miR-200b-3p	PPP1R18	170954	PAR-CLIP	Functional MTI (Weak)	26701625
MIRT738769	hsa-miR-200b-3p	RTL8C	8933	PAR-CLIP	Functional MTI (Weak)	26701625
MIRT738770	hsa-miR-200b-3p	SESN1	27244	PAR-CLIP	Functional MTI (Weak)	26701625
MIRT738771	hsa-miR-200b-3p	SHOX2	6474	PAR-CLIP	Functional MTI (Weak)	26701625
MIRT738772	hsa-miR-200b-3p	TUBB	203068	PAR-CLIP	Functional MTI (Weak)	26701625
MIRT738773	hsa-miR-200b-3p	WNT16	51384	PAR-CLIP	Functional MTI (Weak)	26701625
MIRT763660	hsa-miR-200b-3p	ATP6V1E1	529	PAR-CLIP	Functional MTI (Weak)	27292025
MIRT763661	hsa-miR-200b-3p	BCL2L11	10018	PAR-CLIP	Functional MTI (Weak)	27292025
MIRT763662	hsa-miR-200b-3p	MAMLD1	10046	PAR-CLIP	Functional MTI (Weak)	27292025
MIRT763663	hsa-miR-200b-3p	MBD5	55777	PAR-CLIP	Functional MTI (Weak)	27292025
MIRT763664	hsa-miR-200b-3p	OSTM1	28962	PAR-CLIP	Functional MTI (Weak)	27292025
MIRT763665	hsa-miR-200b-3p	RAP2C	57826	PAR-CLIP	Functional MTI (Weak)	27292025
MIRT763666	hsa-miR-200b-3p	SECISBP2L	9728	PAR-CLIP	Functional MTI (Weak)	27292025
MIRT784124	hsa-miR-200b-3p	C21orf91	54149	HITS-CLIP	Functional MTI (Weak)	27418678

miRTarBase ID	miRNA	Target Gene	Target Gene (Entrez Gene ID)	Experiments	Support Type	References (PMID)
MIRT000002	hsa-miR-20a-5p	HIF1A	3091	Luciferase reporter assay//Western blot//Northern blot//qRT-PCR	Functional MTI	18632605
MIRT000178	hsa-miR-20a-5p	TCEAL1	9338	Luciferase reporter assay//Microarray//Northern blot//qRT-PCR//Western bl	Functional MTI	23059786
MIRT000179	hsa-miR-20a-5p	CCND1	595	Luciferase reporter assay//qRT-PCR//Western blot	Functional MTI	18695042
MIRT000180	hsa-miR-20a-5p	E2F1	1869	Western blot	Functional MTI	19110058
MIRT000181	hsa-miR-20a-5p	BMPR2	659	Luciferase reporter assay//qRT-PCR//Western blot	Functional MTI	19390056
MIRT000597	hsa-miR-20a-5p	CDKN1A	1026	qRT-PCR//Luciferase reporter assay//Western blot	Functional MTI	20190813
MIRT001785	hsa-miR-20a-5p	TGFBR2	7048	Immunoblot//Luciferase reporter assay//Microarray//qRT-PCR//Western blot	Functional MTI	20940405
MIRT003010	hsa-miR-20a-5p	MAP3K12	7786	Luciferase reporter assay	Functional MTI	19666108
MIRT003011	hsa-miR-20a-5p	BCL2	596	Luciferase reporter assay	Functional MTI	19666108
MIRT003012	hsa-miR-20a-5p	MEF2D	4209	Luciferase reporter assay	Functional MTI	19666108
MIRT003369	hsa-miR-20a-5p	PTEN	5728	Luciferase reporter assay//qRT-PCR//Western blot	Non-Functional MTI	20008935
MIRT003382	hsa-miR-20a-5p	APP	351	Luciferase reporter assay//Western blot	Functional MTI	19110058
MIRT003742	hsa-miR-20a-5p	RUNX1	861	Luciferase reporter assay//qRT-PCR//Western blot	Functional MTI	17589498
MIRT003903	hsa-miR-20a-5p	NRAS	4893	Western blot	Non-Functional MTI	19110058
MIRT004450	hsa-miR-20a-5p	VEGFA	7422	ELISA//Luciferase reporter assay	Functional MTI	18320040
MIRT004570	hsa-miR-20a-5p	BCL2L11	10018	PAR-CLIP	Functional MTI (Weak)	23592263
MIRT004711	hsa-miR-20a-5p	MUC17	140453	Immunohistochemistry//Microarray//qRT-PCR	Functional MTI (Weak)	20926598
MIRT005289	hsa-miR-20a-5p	MYC	4609	Western blot	Functional MTI	18695042
MIRT005481	hsa-miR-20a-5p	BNIP2	663	qRT-PCR//Western blot	Functional MTI	21242194
MIRT005627	hsa-miR-20a-5p	THBS1	7057	Microarray//qRT-PCR	Non-Functional MTI (Weak)	20940405
MIRT005631	hsa-miR-20a-5p	SMAD4	4089	Microarray	Non-Functional MTI (Weak)	20940405
MIRT005854	hsa-miR-20a-5p	CCND2	894	Luciferase reporter assay	Functional MTI	21283765
MIRT005855	hsa-miR-20a-5p	E2F3	1871	Luciferase reporter assay	Non-Functional MTI	21283765
MIRT005856	hsa-miR-20a-5p	MAPK9	5601	Luciferase reporter assay	Non-Functional MTI	21283765
MIRT005857	hsa-miR-20a-5p	RB1	5925	Luciferase reporter assay	Functional MTI	21283765
MIRT005858	hsa-miR-20a-5p	RBL1	5933	Luciferase reporter assay	Functional MTI	21283765
MIRT005859	hsa-miR-20a-5p	RBL2	5934	Luciferase reporter assay	Functional MTI	21283765
MIRT005860	hsa-miR-20a-5p	WEE1	7465	Luciferase reporter assay	Functional MTI	21283765
MIRT006178	hsa-miR-20a-5p	IRF2	3660	Luciferase reporter assay//Microarray//Northern blot//qRT-PCR//Western bl	Functional MTI	21880628
MIRT006180	hsa-miR-20a-5p	KIT	3815	Luciferase reporter assay//Microarray//Northern blot//qRT-PCR//Western bl	Functional MTI	21880628
MIRT006289	hsa-miR-20a-5p	EGLN3	112399	Luciferase reporter assay//Western blot	Functional MTI	22182733
MIRT006754	hsa-miR-20a-5p	PPARG	5468	Luciferase reporter assay	Functional MTI	21743293
MIRT006755	hsa-miR-20a-5p	BAMBI	25805	Luciferase reporter assay	Functional MTI	21743293
MIRT006756	hsa-miR-20a-5p	CRIM1	51232	Luciferase reporter assay	Functional MTI	21743293
MIRT006772	hsa-miR-20a-5p	MAP2K3	5606	Luciferase reporter assay	Functional MTI	22696064
MIRT007002	hsa-miR-20a-5p	PURA	5813	Luciferase reporter assay	Functional MTI	22835829
MIRT031081	hsa-miR-20a-5p	JAK1	3716	HITS-CLIP	Functional MTI (Weak)	22473208
MIRT031082	hsa-miR-20a-5p	ARHGAP12	94134	Reporter assay	Functional MTI	21751348
MIRT031083	hsa-miR-20a-5p	TSG101	7251	Reporter assay	Functional MTI	21751348
MIRT035531	hsa-miR-20a-5p	SIRPA	140885	Luciferase reporter assay	Functional MTI	23562609
MIRT050476	hsa-miR-20a-5p	PHF8	23133	CLASH	Functional MTI (Weak)	23622248
MIRT050477	hsa-miR-20a-5p	GPATCH11	253635	CLASH	Functional MTI (Weak)	23622248
MIRT050478	hsa-miR-20a-5p	RPRD1A	55197	CLASH	Functional MTI (Weak)	23622248
MIRT050479	hsa-miR-20a-5p	ATP8B2	57198	CLASH	Functional MTI (Weak)	23622248
MIRT050480	hsa-miR-20a-5p	PSMD2	5708	CLASH	Functional MTI (Weak)	23622248
MIRT050481	hsa-miR-20a-5p	INSIG1	3638	CLASH	Functional MTI (Weak)	23622248
MIRT050482	hsa-miR-20a-5p	RTN2	6253	CLASH	Functional MTI (Weak)	23622248
MIRT050483	hsa-miR-20a-5p	TCEA1	6917	CLASH	Functional MTI (Weak)	23622248
MIRT050484	hsa-miR-20a-5p	PLEKHM3	389072	CLASH	Functional MTI (Weak)	23622248
MIRT050485	hsa-miR-20a-5p	RPS10	6204	CLASH	Functional MTI (Weak)	23622248
MIRT050486	hsa-miR-20a-5p	ALDH18A1	5832	CLASH	Functional MTI (Weak)	23622248
MIRT050487	hsa-miR-20a-5p	UEVLD	55293	CLASH	Functional MTI (Weak)	23622248
MIRT050488	hsa-miR-20a-5p	FGF7	2252	CLASH	Functional MTI (Weak)	23622248
MIRT050489	hsa-miR-20a-5p	SSRP1	6749	CLASH	Functional MTI (Weak)	23622248
MIRT050490	hsa-miR-20a-5p	COX5A	9377	CLASH	Functional MTI (Weak)	23622248
MIRT050491	hsa-miR-20a-5p	AGO1	26523	CLASH	Functional MTI (Weak)	23622248
MIRT050492	hsa-miR-20a-5p	KIAA0100	9703	CLASH	Functional MTI (Weak)	23622248
MIRT050493	hsa-miR-20a-5p	FHL3	2275	CLASH	Functional MTI (Weak)	23622248
MIRT050494	hsa-miR-20a-5p	GDI2	2665	CLASH	Functional MTI (Weak)	23622248
MIRT050495	hsa-miR-20a-5p	INTS3	65123	CLASH	Functional MTI (Weak)	23622248
MIRT050496	hsa-miR-20a-5p	HMG20A	10363	CLASH	Functional MTI (Weak)	23622248
MIRT050497	hsa-miR-20a-5p	NAXE	128240	CLASH	Functional MTI (Weak)	23622248
MIRT050498	hsa-miR-20a-5p	PRKD3	23683	CLASH	Functional MTI (Weak)	23622248
MIRT050499	hsa-miR-20a-5p	AP3S2	10239	CLASH	Functional MTI (Weak)	23622248
MIRT050500	hsa-miR-20a-5p	PCNX4	64430	CLASH	Functional MTI (Weak)	23622248
MIRT050501	hsa-miR-20a-5p	IKZF5	64376	CLASH	Functional MTI (Weak)	23622248
MIRT050502	hsa-miR-20a-5p	TMEM97	27346	CLASH	Functional MTI (Weak)	23622248
MIRT050503	hsa-miR-20a-5p	ATP6	4508	CLASH	Functional MTI (Weak)	23622248
MIRT050504	hsa-miR-20a-5p	RPA2	6118	CLASH	Functional MTI (Weak)	23622248
MIRT050505	hsa-miR-20a-5p	PHYH	5264	CLASH	Functional MTI (Weak)	23622248
MIRT050506	hsa-miR-20a-5p	KIF2C	11004	CLASH	Functional MTI (Weak)	23622248
MIRT050507	hsa-miR-20a-5p	DDX5	1655	CLASH	Functional MTI (Weak)	23622248
MIRT050508	hsa-miR-20a-5p	DTX2	113878	CLASH	Functional MTI (Weak)	23622248
MIRT050509	hsa-miR-20a-5p	MPHOSPH8	54737	CLASH	Functional MTI (Weak)	23622248
MIRT050510	hsa-miR-20a-5p	HAUS2	55142	CLASH	Functional MTI (Weak)	23622248
MIRT050511	hsa-miR-20a-5p	ZNF331	55422	CLASH	Functional MTI (Weak)	23622248
MIRT050512	hsa-miR-20a-5p	PPP6R3	55291	CLASH	Functional MTI (Weak)	23622248
MIRT050513	hsa-miR-20a-5p	METTL22	79091	CLASH	Functional MTI (Weak)	23622248
MIRT050514	hsa-miR-20a-5p	PPAN	56342	CLASH	Functional MTI (Weak)	23622248
MIRT050515	hsa-miR-20a-5p	FBL	2091	CLASH	Functional MTI (Weak)	23622248
MIRT050516	hsa-miR-20a-5p	RPL31	6160	CLASH	Functional MTI (Weak)	23622248
MIRT050517	hsa-miR-20a-5p	SEPT2	4735	CLASH	Functional MTI (Weak)	23622248
MIRT050518	hsa-miR-20a-5p	TOMM20	9804	CLASH	Functional MTI (Weak)	23622248
MIRT050519	hsa-miR-20a-5p	NCOR2	9612	CLASH	Functional MTI (Weak)	23622248
MIRT050520	hsa-miR-20a-5p	PSD3	23362	CLASH	Functional MTI (Weak)	23622248
MIRT050521	hsa-miR-20a-5p	AP3D1	8943	CLASH	Functional MTI (Weak)	23622248
MIRT050522	hsa-miR-20a-5p	TBC1D15	64786	CLASH	Functional MTI (Weak)	23622248
MIRT050523	hsa-miR-20a-5p	DPY19L4	286148	CLASH	Functional MTI (Weak)	23622248
MIRT050524	hsa-miR-20a-5p	PTPN23	25930	CLASH	Functional MTI (Weak)	23622248
MIRT050525	hsa-miR-20a-5p	C11orf68	83638	CLASH	Functional MTI (Weak)	23622248
MIRT050526	hsa-miR-20a-5p	CTR9	9646	CLASH	Functional MTI (Weak)	23622248
MIRT050527	hsa-miR-20a-5p	PAIP1	10605	CLASH	Functional MTI (Weak)	23622248
MIRT050528	hsa-miR-20a-5p	L2HGDH	79944	CLASH	Functional MTI (Weak)	23622248
MIRT050529	hsa-miR-20a-5p	CDT1	81620	CLASH	Functional MTI (Weak)	23622248
MIRT050530	hsa-miR-20a-5p	RPL30	6156	CLASH	Functional MTI (Weak)	23622248
MIRT050531	hsa-miR-20a-5p	MRS2	57380	CLASH	Functional MTI (Weak)	23622248
MIRT050532	hsa-miR-20a-5p	TMX4	56255	CLASH	Functional MTI (Weak)	23622248
MIRT050533	hsa-miR-20a-5p	LAMTOR1	55004	CLASH	Functional MTI (Weak)	23622248
MIRT050534	hsa-miR-20a-5p	ADGRL3	23284	CLASH	Functional MTI (Weak)	23622248
MIRT050535	hsa-miR-20a-5p	RBM10	8241	CLASH	Functional MTI (Weak)	23622248
MIRT050536	hsa-miR-20a-5p	ADGRE2	30817	CLASH	Functional MTI (Weak)	23622248
MIRT050537	hsa-miR-20a-5p	FBXO3	26273	CLASH	Functional MTI (Weak)	23622248
MIRT050538	hsa-miR-20a-5p	MLXIP	22877	CLASH	Functional MTI (Weak)	23622248

MIRT050539	hsa-miR-20a-5p	RNGTT	8732	CLASH	Functional MTI (Weak)	23622248
MIRT050540	hsa-miR-20a-5p	MAD1L1	8379	CLASH	Functional MTI (Weak)	23622248
MIRT050541	hsa-miR-20a-5p	DLC1	10395	CLASH	Functional MTI (Weak)	23622248
MIRT050542	hsa-miR-20a-5p	NUP214	8021	CLASH	Functional MTI (Weak)	23622248
MIRT050543	hsa-miR-20a-5p	PAQR5	54852	CLASH	Functional MTI (Weak)	23622248
MIRT050544	hsa-miR-20a-5p	BTBD2	55643	CLASH	Functional MTI (Weak)	23622248
MIRT050545	hsa-miR-20a-5p	XYLT2	64132	CLASH	Functional MTI (Weak)	23622248
MIRT050546	hsa-miR-20a-5p	ZNF398	57541	CLASH	Functional MTI (Weak)	23622248
MIRT050547	hsa-miR-20a-5p	CEP120	153241	CLASH	Functional MTI (Weak)	23622248
MIRT050548	hsa-miR-20a-5p	IL17RC	84818	CLASH	Functional MTI (Weak)	23622248
MIRT050549	hsa-miR-20a-5p	UBE2C	11065	CLASH	Functional MTI (Weak)	23622248
MIRT050550	hsa-miR-20a-5p	PGK1	5230	CLASH	Functional MTI (Weak)	23622248
MIRT050551	hsa-miR-20a-5p	ORMDL3	94103	CLASH	Functional MTI (Weak)	23622248
MIRT050552	hsa-miR-20a-5p	TUBB	203068	CLASH	Functional MTI (Weak)	23622248
MIRT050553	hsa-miR-20a-5p	TDRD3	81550	CLASH	Functional MTI (Weak)	23622248
MIRT050554	hsa-miR-20a-5p	DLG5	9231	CLASH	Functional MTI (Weak)	23622248
MIRT050555	hsa-miR-20a-5p	VEZF1	7716	CLASH	Functional MTI (Weak)	23622248
MIRT050556	hsa-miR-20a-5p	CCDC88C	440193	CLASH	Functional MTI (Weak)	23622248
MIRT050557	hsa-miR-20a-5p	USP10	9100	CLASH	Functional MTI (Weak)	23622248
MIRT050558	hsa-miR-20a-5p	KIAA1191	57179	CLASH	Functional MTI (Weak)	23622248
MIRT050559	hsa-miR-20a-5p	STAT3	6774	CLASH	Functional MTI (Weak)	23622248
MIRT050560	hsa-miR-20a-5p	GATA6	2627	CLASH	Functional MTI (Weak)	23622248
MIRT050561	hsa-miR-20a-5p	RPL18A	6142	CLASH	Functional MTI (Weak)	23622248
MIRT050562	hsa-miR-20a-5p	SARAF	51669	CLASH	Functional MTI (Weak)	23622248
MIRT050563	hsa-miR-20a-5p	ARL9	132946	CLASH	Functional MTI (Weak)	23622248
MIRT050564	hsa-miR-20a-5p	CTSA	5476	CLASH	Functional MTI (Weak)	23622248
MIRT050565	hsa-miR-20a-5p	ABCA3	21	CLASH	Functional MTI (Weak)	23622248
MIRT050566	hsa-miR-20a-5p	MRPL13	28998	CLASH	Functional MTI (Weak)	23622248
MIRT050567	hsa-miR-20a-5p	MAN1C1	57134	CLASH	Functional MTI (Weak)	23622248
MIRT050568	hsa-miR-20a-5p	AGO4	192670	CLASH	Functional MTI (Weak)	23622248
MIRT050569	hsa-miR-20a-5p	BACH1	571	CLASH	Functional MTI (Weak)	23622248
MIRT050570	hsa-miR-20a-5p	RFC3	5983	CLASH	Functional MTI (Weak)	23622248
MIRT050571	hsa-miR-20a-5p	ARHGEF7	8874	CLASH	Functional MTI (Weak)	23622248
MIRT050572	hsa-miR-20a-5p	GPN2	54707	CLASH	Functional MTI (Weak)	23622248
MIRT050573	hsa-miR-20a-5p	LDHB	3945	CLASH	Functional MTI (Weak)	23622248
MIRT050574	hsa-miR-20a-5p	PTPRS	5802	CLASH	Functional MTI (Weak)	23622248
MIRT050575	hsa-miR-20a-5p	PPP2R1A	5518	CLASH	Functional MTI (Weak)	23622248
MIRT050576	hsa-miR-20a-5p	CDK16	5127	CLASH	Functional MTI (Weak)	23622248
MIRT050577	hsa-miR-20a-5p	WBP4	11193	CLASH	Functional MTI (Weak)	23622248
MIRT050578	hsa-miR-20a-5p	CCNB1	891	CLASH	Functional MTI (Weak)	23622248
MIRT050579	hsa-miR-20a-5p	POGZ	23126	CLASH	Functional MTI (Weak)	23622248
MIRT050580	hsa-miR-20a-5p	KLHL15	80311	CLASH	Functional MTI (Weak)	23622248
MIRT050581	hsa-miR-20a-5p	RTFDC1	51507	CLASH	Functional MTI (Weak)	23622248
MIRT050582	hsa-miR-20a-5p	FLNA	2316	CLASH	Functional MTI (Weak)	23622248
MIRT050583	hsa-miR-20a-5p	PLXNA1	5361	CLASH	Functional MTI (Weak)	23622248
MIRT050584	hsa-miR-20a-5p	ADSS	159	CLASH	Functional MTI (Weak)	23622248
MIRT050585	hsa-miR-20a-5p	MANEAL	149175	CLASH	Functional MTI (Weak)	23622248
MIRT050586	hsa-miR-20a-5p	NUP188	23511	CLASH	Functional MTI (Weak)	23622248
MIRT050587	hsa-miR-20a-5p	ECI1	1632	CLASH	Functional MTI (Weak)	23622248
MIRT050588	hsa-miR-20a-5p	NCOA3	8202	CLASH	Functional MTI (Weak)	23622248
MIRT050589	hsa-miR-20a-5p	MORF4L2	9643	CLASH	Functional MTI (Weak)	23622248
MIRT050590	hsa-miR-20a-5p	ATL3	25923	CLASH	Functional MTI (Weak)	23622248
MIRT050591	hsa-miR-20a-5p	FOXJ3	22887	CLASH	Functional MTI (Weak)	23622248
MIRT050592	hsa-miR-20a-5p	PRRC2C	23215	CLASH	Functional MTI (Weak)	23622248
MIRT050593	hsa-miR-20a-5p	RPL21	6144	CLASH	Functional MTI (Weak)	23622248
MIRT050594	hsa-miR-20a-5p	SELENBP1	8991	CLASH	Functional MTI (Weak)	23622248
MIRT050595	hsa-miR-20a-5p	YBX1	4904	CLASH	Functional MTI (Weak)	23622248
MIRT050596	hsa-miR-20a-5p	B4GALT2	8704	CLASH	Functional MTI (Weak)	23622248
MIRT050597	hsa-miR-20a-5p	PYGB	5834	CLASH	Functional MTI (Weak)	23622248
MIRT050598	hsa-miR-20a-5p	AKR7A2	8574	CLASH	Functional MTI (Weak)	23622248
MIRT050599	hsa-miR-20a-5p	C9orf78	51759	CLASH	Functional MTI (Weak)	23622248
MIRT050600	hsa-miR-20a-5p	STIL	6491	CLASH	Functional MTI (Weak)	23622248
MIRT050601	hsa-miR-20a-5p	CDK19	23097	CLASH	Functional MTI (Weak)	23622248
MIRT050602	hsa-miR-20a-5p	KDM4D	55693	CLASH	Functional MTI (Weak)	23622248
MIRT050603	hsa-miR-20a-5p	UQCRC1	7384	CLASH	Functional MTI (Weak)	23622248
MIRT050604	hsa-miR-20a-5p	RUFY2	55680	CLASH	Functional MTI (Weak)	23622248
MIRT050605	hsa-miR-20a-5p	RPS27	6232	CLASH	Functional MTI (Weak)	23622248
MIRT050606	hsa-miR-20a-5p	BTN3A1	11119	CLASH	Functional MTI (Weak)	23622248
MIRT050607	hsa-miR-20a-5p	PBXIP1	57326	CLASH	Functional MTI (Weak)	23622248
MIRT050608	hsa-miR-20a-5p	ARFGEF2	10564	CLASH	Functional MTI (Weak)	23622248
MIRT050609	hsa-miR-20a-5p	NUDT21	11051	CLASH	Functional MTI (Weak)	23622248
MIRT050610	hsa-miR-20a-5p	NETO2	81831	CLASH	Functional MTI (Weak)	23622248
MIRT050611	hsa-miR-20a-5p	SLC25A28	81894	CLASH	Functional MTI (Weak)	23622248
MIRT050612	hsa-miR-20a-5p	NAP1L1	4673	CLASH	Functional MTI (Weak)	23622248
MIRT050613	hsa-miR-20a-5p	PHC1	1911	CLASH	Functional MTI (Weak)	23622248
MIRT050614	hsa-miR-20a-5p	ZNF706	51123	CLASH	Functional MTI (Weak)	23622248
MIRT050615	hsa-miR-20a-5p	CCDC47	57003	CLASH	Functional MTI (Weak)	23622248
MIRT050616	hsa-miR-20a-5p	ARPC2	10109	CLASH	Functional MTI (Weak)	23622248
MIRT050617	hsa-miR-20a-5p	EIF4G2	1982	CLASH	Functional MTI (Weak)	23622248
MIRT050618	hsa-miR-20a-5p	MAGOHB	55110	CLASH	Functional MTI (Weak)	23622248
MIRT050619	hsa-miR-20a-5p	ZNF598	90850	CLASH	Functional MTI (Weak)	23622248
MIRT050620	hsa-miR-20a-5p	P3H4	10609	CLASH	Functional MTI (Weak)	23622248
MIRT050621	hsa-miR-20a-5p	CERS2	29956	CLASH	Functional MTI (Weak)	23622248
MIRT050622	hsa-miR-20a-5p	LYPD6	130574	CLASH	Functional MTI (Weak)	23622248
MIRT050623	hsa-miR-20a-5p	HEXIM1	10614	CLASH	Functional MTI (Weak)	23622248
MIRT050624	hsa-miR-20a-5p	WAC	51322	CLASH	Functional MTI (Weak)	23622248
MIRT050625	hsa-miR-20a-5p	ZNFX1	57169	CLASH	Functional MTI (Weak)	23622248
MIRT050626	hsa-miR-20a-5p	RBM12B	389677	CLASH	Functional MTI (Weak)	23622248
MIRT052914	hsa-miR-20a-5p	LIMK1	3984	Luciferase reporter assay//Microarray//qRT-PCR//Western blot	Functional MTI	24858712
MIRT052971	hsa-miR-20a-5p	PHLPP2	23035	Immunoblot//Luciferase reporter assay	Functional MTI	22116552
MIRT053007	hsa-miR-20a-5p	GJA1	2697	Luciferase reporter assay//qRT-PCR//Western blot	Functional MTI	22785209
MIRT053023	hsa-miR-20a-5p	DUSP2	1844	qRT-PCR//Western blot//Reporter assay	Functional MTI	22648654
MIRT053109	hsa-miR-20a-5p	ITGB8	3696	Luciferase reporter assay//qRT-PCR	Functional MTI	23602254
MIRT053159	hsa-miR-20a-5p	SMAD7	4092	Luciferase reporter assay//qRT-PCR//Western blot	Functional MTI	23665284
MIRT053208	hsa-miR-20a-5p	MAP3K5	4217	Luciferase reporter assay//qRT-PCR//Western blot	Functional MTI	23087182
MIRT053332	hsa-miR-20a-5p	MCL1	4170	Immunohistochemistry//Luciferase reporter assay//qRT-PCR//Western blot	Functional MTI	23594563
MIRT053505	hsa-miR-20a-5p	TP53INP1	94241	Luciferase reporter assay//qRT-PCR//Western blot	Functional MTI	23333058
MIRT053563	hsa-miR-20a-5p	EGR2	1959	Flow//Luciferase reporter assay//qRT-PCR//Western blot	Functional MTI	23924943
MIRT054860	hsa-miR-20a-5p	ABL2	27	Luciferase reporter assay//qRT-PCR//Western blot	Functional MTI	24464651
MIRT055020	hsa-miR-20a-5p	TPRG1L	127262	HITS-CLIP	Functional MTI (Weak)	22473208
MIRT055382	hsa-miR-20a-5p	SHOC2	8036	PAR-CLIP	Functional MTI (Weak)	23592263
MIRT055649	hsa-miR-20a-5p	WDR37	22884	HITS-CLIP	Functional MTI (Weak)	22473208
MIRT056476	hsa-miR-20a-5p	PFKP	5214	HITS-CLIP	Functional MTI (Weak)	22473208
MIRT056811	hsa-miR-20a-5p	REEP3	221035	HITS-CLIP	Functional MTI (Weak)	22473208
MIRT057384	hsa-miR-20a-5p	TNKS2	80351	HITS-CLIP	Functional MTI (Weak)	22473208

MIRT057822	hsa-miR-20a-5p	SLC30A7	148867	PAR-CLIP	Functional MTI (Weak)	23592263
MIRT058912	hsa-miR-20a-5p	FAM46C	54855	HITS-CLIP	Functional MTI (Weak)	22473208
MIRT059186	hsa-miR-20a-5p	CRY2	1408	HITS-CLIP	Functional MTI (Weak)	22473208
MIRT060075	hsa-miR-20a-5p	TMEM138	51524	HITS-CLIP	Functional MTI (Weak)	22473208
MIRT060678	hsa-miR-20a-5p	KLHL20	27252	HITS-CLIP	Functional MTI (Weak)	22473208
MIRT061181	hsa-miR-20a-5p	MED17	9440	HITS-CLIP	Functional MTI (Weak)	22473208
MIRT061789	hsa-miR-20a-5p	PPP1R15B	84919	PAR-CLIP	Functional MTI (Weak)	24398324
MIRT063054	hsa-miR-20a-5p	ULK1	8408	PAR-CLIP//HITS-CLIP	Functional MTI (Weak)	21572407
MIRT063434	hsa-miR-20a-5p	SKI	6497	HITS-CLIP	Functional MTI (Weak)	22473208
MIRT064435	hsa-miR-20a-5p	GPR137B	7107	PAR-CLIP	Functional MTI (Weak)	20371350
MIRT064797	hsa-miR-20a-5p	ZBTB18	10472	HITS-CLIP	Functional MTI (Weak)	22473208
MIRT065364	hsa-miR-20a-5p	TMBIM6	7009	HITS-CLIP	Functional MTI (Weak)	22473208
MIRT065670	hsa-miR-20a-5p	ACVR1B	91	PAR-CLIP	Functional MTI (Weak)	23446348
MIRT065858	hsa-miR-20a-5p	GDF11	10220	PAR-CLIP	Functional MTI (Weak)	21572407
MIRT065886	hsa-miR-20a-5p	RAB5B	5869	HITS-CLIP	Functional MTI (Weak)	22473208
MIRT067229	hsa-miR-20a-5p	FOXJ2	55810	PAR-CLIP	Functional MTI (Weak)	21572407
MIRT068492	hsa-miR-20a-5p	NHLRC3	387921	HITS-CLIP	Functional MTI (Weak)	22473208
MIRT070839	hsa-miR-20a-5p	EIF2S1	1965	PAR-CLIP	Functional MTI (Weak)	21572407
MIRT070995	hsa-miR-20a-5p	SMOC1	64093	PAR-CLIP	Functional MTI (Weak)	21572407
MIRT071324	hsa-miR-20a-5p	CMPK1	51727	HITS-CLIP	Functional MTI (Weak)	22473208
MIRT071903	hsa-miR-20a-5p	ZFYVE9	9372	PAR-CLIP	Functional MTI (Weak)	23592263
MIRT072247	hsa-miR-20a-5p	B2M	567	PAR-CLIP	Functional MTI (Weak)	23592263
MIRT072567	hsa-miR-20a-5p	USP3	9960	HITS-CLIP	Functional MTI (Weak)	22473208
MIRT073117	hsa-miR-20a-5p	UBE2Q2	92912	HITS-CLIP	Functional MTI (Weak)	22473208
MIRT073374	hsa-miR-20a-5p	ABHD2	11057	HITS-CLIP	Functional MTI (Weak)	22473208
MIRT073407	hsa-miR-20a-5p	SEMA4B	10509	HITS-CLIP	Functional MTI (Weak)	22473208
MIRT074789	hsa-miR-20a-5p	CYLD	1540	HITS-CLIP	Functional MTI (Weak)	22473208
MIRT074891	hsa-miR-20a-5p	CHD9	80205	HITS-CLIP	Functional MTI (Weak)	22473208
MIRT075775	hsa-miR-20a-5p	KIAA0513	9764	PAR-CLIP	Functional MTI (Weak)	23592263
MIRT076177	hsa-miR-20a-5p	GID4	79018	HITS-CLIP	Functional MTI (Weak)	22473208
MIRT077064	hsa-miR-20a-5p	KRT10	3858	PAR-CLIP	Functional MTI (Weak)	23592263
MIRT077831	hsa-miR-20a-5p	MINK1	50488	HITS-CLIP	Functional MTI (Weak)	22473208
MIRT078811	hsa-miR-20a-5p	UNK	85451	PAR-CLIP	Functional MTI (Weak)	22012620
MIRT079344	hsa-miR-20a-5p	CCDC137	339230	HITS-CLIP	Functional MTI (Weak)	22473208
MIRT079411	hsa-miR-20a-5p	FOXK2	3607	HITS-CLIP	Functional MTI (Weak)	22473208
MIRT079772	hsa-miR-20a-5p	CABLES1	91768	PAR-CLIP	Functional MTI (Weak)	23592263
MIRT080178	hsa-miR-20a-5p	PRKACB	5567	PAR-CLIP	Functional MTI (Weak)	21572407
MIRT080848	hsa-miR-20a-5p	RAB12	201475	HITS-CLIP	Functional MTI (Weak)	22473208
MIRT081117	hsa-miR-20a-5p	LDLR	3949	HITS-CLIP	Functional MTI (Weak)	22473208
MIRT081198	hsa-miR-20a-5p	MIDN	90007	HITS-CLIP	Functional MTI (Weak)	22473208
MIRT081981	hsa-miR-20a-5p	GRAMD1A	57655	HITS-CLIP	Functional MTI (Weak)	22473208
MIRT082290	hsa-miR-20a-5p	FNBP1L	54874	PAR-CLIP	Functional MTI (Weak)	23446348
MIRT083739	hsa-miR-20a-5p	PARD6B	84612	HITS-CLIP	Functional MTI (Weak)	22473208
MIRT083960	hsa-miR-20a-5p	RAB22A	57403	HITS-CLIP	Functional MTI (Weak)	22473208
MIRT084344	hsa-miR-20a-5p	RRM2	6241	HITS-CLIP	Functional MTI (Weak)	22473208
MIRT085173	hsa-miR-20a-5p	SLC5A3	6526	PAR-CLIP	Functional MTI (Weak)	21572407
MIRT085375	hsa-miR-20a-5p	SPOPL	339745	HITS-CLIP	Functional MTI (Weak)	22473208
MIRT085867	hsa-miR-20a-5p	TANC1	85461	HITS-CLIP	Functional MTI (Weak)	22473208
MIRT086425	hsa-miR-20a-5p	NABP1	64859	HITS-CLIP	Functional MTI (Weak)	22473208
MIRT087604	hsa-miR-20a-5p	ATG16L1	55054	HITS-CLIP	Functional MTI (Weak)	22473208
MIRT088033	hsa-miR-20a-5p	UBXN2A	165324	PAR-CLIP	Functional MTI (Weak)	21572407
MIRT088337	hsa-miR-20a-5p	MAPRE3	22924	PAR-CLIP	Functional MTI (Weak)	24398324
MIRT090633	hsa-miR-20a-5p	U2SURP	23350	HITS-CLIP	Functional MTI (Weak)	22473208
MIRT092685	hsa-miR-20a-5p	C3orf38	285237	PAR-CLIP	Functional MTI (Weak)	24398324
MIRT093800	hsa-miR-20a-5p	KLF3	51274	PAR-CLIP	Functional MTI (Weak)	22012620
MIRT093941	hsa-miR-20a-5p	SLAIN2	57606	HITS-CLIP	Functional MTI (Weak)	22473208
MIRT095200	hsa-miR-20a-5p	SMAD5	4090	HITS-CLIP	Functional MTI (Weak)	22473208
MIRT095719	hsa-miR-20a-5p	ANKH	56172	PAR-CLIP	Functional MTI (Weak)	23592263
MIRT095997	hsa-miR-20a-5p	ATP6V0E1	8992	HITS-CLIP	Functional MTI (Weak)	22473208
MIRT096308	hsa-miR-20a-5p	SQSTM1	8878	HITS-CLIP	Functional MTI (Weak)	22473208
MIRT097128	hsa-miR-20a-5p	FCHO2	115548	HITS-CLIP	Functional MTI (Weak)	22473208
MIRT097603	hsa-miR-20a-5p	POLR3G	10622	HITS-CLIP	Functional MTI (Weak)	22473208
MIRT097649	hsa-miR-20a-5p	LYSMD3	116068	HITS-CLIP	Functional MTI (Weak)	22473208
MIRT099308	hsa-miR-20a-5p	QKI	9444	PAR-CLIP	Functional MTI (Weak)	23446348
MIRT099355	hsa-miR-20a-5p	C6orf120	387263	HITS-CLIP	Functional MTI (Weak)	22473208
MIRT099824	hsa-miR-20a-5p	SOX4	6659	PAR-CLIP	Functional MTI (Weak)	23592263
MIRT100277	hsa-miR-20a-5p	MICB	4277	HITS-CLIP	Functional MTI (Weak)	22473208
MIRT100454	hsa-miR-20a-5p	ZBTB9	221504	HITS-CLIP	Functional MTI (Weak)	22473208
MIRT100945	hsa-miR-20a-5p	CENPQ	55166	PAR-CLIP	Functional MTI (Weak)	23446348
MIRT102221	hsa-miR-20a-5p	HBP1	26959	HITS-CLIP	Functional MTI (Weak)	22473208
MIRT102294	hsa-miR-20a-5p	DNAJB9	4189	PAR-CLIP	Functional MTI (Weak)	24398324
MIRT103189	hsa-miR-20a-5p	SP4	6671	PAR-CLIP	Functional MTI (Weak)	23446348
MIRT104161	hsa-miR-20a-5p	PHTF2	57157	PAR-CLIP	Functional MTI (Weak)	23446348
MIRT104394	hsa-miR-20a-5p	ANKIB1	54467	HITS-CLIP	Functional MTI (Weak)	22473208
MIRT108651	hsa-miR-20a-5p	ZBTB33	10009	HITS-CLIP	Functional MTI (Weak)	22473208
MIRT108719	hsa-miR-20a-5p	XIAP	331	PAR-CLIP	Functional MTI (Weak)	21572407
MIRT110266	hsa-miR-20a-5p	GBF1	8729	HITS-CLIP	Functional MTI (Weak)	22473208
MIRT112089	hsa-miR-20a-5p	TIMM17A	10440	PAR-CLIP	Functional MTI (Weak)	22012620
MIRT115779	hsa-miR-20a-5p	CAPN15	6650	PAR-CLIP	Functional MTI (Weak)	23592263
MIRT121800	hsa-miR-20a-5p	GRPEL2	134266	HITS-CLIP	Functional MTI (Weak)	22473208
MIRT122363	hsa-miR-20a-5p	RGMB	285704	PAR-CLIP	Functional MTI (Weak)	21572407
MIRT124125	hsa-miR-20a-5p	GINS4	84296	PAR-CLIP	Functional MTI (Weak)	23446348
MIRT125725	hsa-miR-20a-5p	TRIM8	81603	HITS-CLIP	Functional MTI (Weak)	22473208
MIRT126308	hsa-miR-20a-5p	ACADSB	36	HITS-CLIP	Functional MTI (Weak)	22473208
MIRT126344	hsa-miR-20a-5p	ZRANB1	54764	HITS-CLIP	Functional MTI (Weak)	22473208
MIRT126550	hsa-miR-20a-5p	MASTL	84930	HITS-CLIP	Functional MTI (Weak)	22473208
MIRT127161	hsa-miR-20a-5p	VPS26A	9559	HITS-CLIP	Functional MTI (Weak)	22473208
MIRT129131	hsa-miR-20a-5p	ARCN1	372	HITS-CLIP	Functional MTI (Weak)	22473208
MIRT130070	hsa-miR-20a-5p	TXNIP	10628	HITS-CLIP	Functional MTI (Weak)	22473208
MIRT132394	hsa-miR-20a-5p	PPP1R12B	4660	HITS-CLIP	Functional MTI (Weak)	22473208
MIRT133313	hsa-miR-20a-5p	ORAI1	84876	HITS-CLIP	Functional MTI (Weak)	22473208
MIRT134275	hsa-miR-20a-5p	DNM1L	10059	HITS-CLIP	Functional MTI (Weak)	22473208
MIRT135706	hsa-miR-20a-5p	PIP4K2C	79837	HITS-CLIP	Functional MTI (Weak)	22473208
MIRT135793	hsa-miR-20a-5p	GNS	2799	HITS-CLIP	Functional MTI (Weak)	22473208
MIRT136562	hsa-miR-20a-5p	TXLNA	200081	HITS-CLIP	Functional MTI (Weak)	22473208
MIRT138110	hsa-miR-20a-5p	BRMS1L	84312	HITS-CLIP	Functional MTI (Weak)	22473208
MIRT138348	hsa-miR-20a-5p	FRMD6	122786	HITS-CLIP	Functional MTI (Weak)	22473208
MIRT138792	hsa-miR-20a-5p	SUSD6	9766	HITS-CLIP	Functional MTI (Weak)	22473208
MIRT140626	hsa-miR-20a-5p	PLEKHO2	80301	HITS-CLIP	Functional MTI (Weak)	22473208
MIRT140794	hsa-miR-20a-5p	SMAD6	4091	HITS-CLIP	Functional MTI (Weak)	22473208
MIRT141126	hsa-miR-20a-5p	SCAMP5	192683	HITS-CLIP	Functional MTI (Weak)	22473208
MIRT141698	hsa-miR-20a-5p	RCCD1	91433	HITS-CLIP	Functional MTI (Weak)	22473208
MIRT142095	hsa-miR-20a-5p	CCP110	9738	HITS-CLIP	Functional MTI (Weak)	22473208
MIRT142381	hsa-miR-20a-5p	TNRC6A	27327	HITS-CLIP	Functional MTI (Weak)	22473208
MIRT144207	hsa-miR-20a-5p	SNTB2	6645	HITS-CLIP	Functional MTI (Weak)	22473208

MIRT144294	hsa-miR-20a-5p	NFAT5	10725	HITS-CLIP	Functional MTI (Weak)	22473208
MIRT144978	hsa-miR-20a-5p	PAFAH1B1	5048	HITS-CLIP	Functional MTI (Weak)	22473208
MIRT145019	hsa-miR-20a-5p	TNFAIP1	7126	HITS-CLIP	Functional MTI (Weak)	22473208
MIRT145597	hsa-miR-20a-5p	LASP1	3927	HITS-CLIP	Functional MTI (Weak)	22473208
MIRT146071	hsa-miR-20a-5p	RUNDC1	146923	HITS-CLIP	Functional MTI (Weak)	22473208
MIRT147118	hsa-miR-20a-5p	MAP3K3	4215	HITS-CLIP	Functional MTI (Weak)	22473208
MIRT147273	hsa-miR-20a-5p	KPNA2	3838	HITS-CLIP	Functional MTI (Weak)	22473208
MIRT147921	hsa-miR-20a-5p	CAMTA1	23261	HITS-CLIP	Functional MTI (Weak)	22473208
MIRT148868	hsa-miR-20a-5p	ANKRD12	23253	HITS-CLIP	Functional MTI (Weak)	22473208
MIRT151690	hsa-miR-20a-5p	CHAF1A	10036	HITS-CLIP	Functional MTI (Weak)	22473208
MIRT151799	hsa-miR-20a-5p	BLOC1S3	388552	HITS-CLIP	Functional MTI (Weak)	22473208
MIRT151853	hsa-miR-20a-5p	ARHGAP35	2909	HITS-CLIP	Functional MTI (Weak)	22473208
MIRT151932	hsa-miR-20a-5p	TBC1D17	79735	HITS-CLIP	Functional MTI (Weak)	22473208
MIRT152367	hsa-miR-20a-5p	ARHGEF18	23370	HITS-CLIP	Functional MTI (Weak)	22473208
MIRT152673	hsa-miR-20a-5p	POFUT1	23509	HITS-CLIP	Functional MTI (Weak)	22473208
MIRT153327	hsa-miR-20a-5p	MAVS	57506	PAR-CLIP	Functional MTI (Weak)	21572407
MIRT153454	hsa-miR-20a-5p	TTPAL	79183	HITS-CLIP	Functional MTI (Weak)	22473208
MIRT153969	hsa-miR-20a-5p	PRNP	5621	HITS-CLIP	Functional MTI (Weak)	22473208
MIRT155231	hsa-miR-20a-5p	IFNAR2	3455	HITS-CLIP	Functional MTI (Weak)	22473208
MIRT155333	hsa-miR-20a-5p	IFNAR1	3454	HITS-CLIP	Functional MTI (Weak)	22473208
MIRT155886	hsa-miR-20a-5p	SIK1	150094	PAR-CLIP	Functional MTI (Weak)	21572407
MIRT156404	hsa-miR-20a-5p	RAPGEF4	11069	HITS-CLIP	Functional MTI (Weak)	22473208
MIRT156640	hsa-miR-20a-5p	C2orf69	205327	HITS-CLIP	Functional MTI (Weak)	22473208
MIRT157145	hsa-miR-20a-5p	FAM117B	150864	HITS-CLIP	Functional MTI (Weak)	22473208
MIRT157585	hsa-miR-20a-5p	MTMR3	8897	HITS-CLIP	Functional MTI (Weak)	22473208
MIRT158288	hsa-miR-20a-5p	ASB1	51665	HITS-CLIP	Functional MTI (Weak)	22473208
MIRT158573	hsa-miR-20a-5p	TNRC6B	23112	PAR-CLIP	Functional MTI (Weak)	23446348
MIRT159105	hsa-miR-20a-5p	NRBP1	29959	HITS-CLIP	Functional MTI (Weak)	22473208
MIRT159399	hsa-miR-20a-5p	FEZ2	9637	HITS-CLIP	Functional MTI (Weak)	22473208
MIRT160004	hsa-miR-20a-5p	TET3	200424	HITS-CLIP	Functional MTI (Weak)	22473208
MIRT164198	hsa-miR-20a-5p	GAB1	2549	HITS-CLIP	Functional MTI (Weak)	22473208
MIRT164524	hsa-miR-20a-5p	MSMO1	6307	HITS-CLIP	Functional MTI (Weak)	22473208
MIRT164659	hsa-miR-20a-5p	NSD2	7468	HITS-CLIP	Functional MTI (Weak)	22473208
MIRT164720	hsa-miR-20a-5p	ADD1	118	HITS-CLIP	Functional MTI (Weak)	22473208
MIRT166062	hsa-miR-20a-5p	FAF2	23197	HITS-CLIP	Functional MTI (Weak)	22473208
MIRT167840	hsa-miR-20a-5p	HECA	51696	HITS-CLIP	Functional MTI (Weak)	22473208
MIRT168216	hsa-miR-20a-5p	BTN3A2	11118	HITS-CLIP	Functional MTI (Weak)	22473208
MIRT169663	hsa-miR-20a-5p	AGFG2	3268	HITS-CLIP	Functional MTI (Weak)	22473208
MIRT170564	hsa-miR-20a-5p	CASP2	835	HITS-CLIP	Functional MTI (Weak)	22473208
MIRT170849	hsa-miR-20a-5p	TAX1BP1	8887	HITS-CLIP	Functional MTI (Weak)	22473208
MIRT172197	hsa-miR-20a-5p	OXR1	55074	HITS-CLIP	Functional MTI (Weak)	22473208
MIRT173112	hsa-miR-20a-5p	E2F5	1875	HITS-CLIP	Functional MTI (Weak)	22473208
MIRT173688	hsa-miR-20a-5p	PRPF4	9128	HITS-CLIP	Functional MTI (Weak)	22473208
MIRT175379	hsa-miR-20a-5p	ACSL4	2182	PAR-CLIP//HITS-CLIP	Functional MTI (Weak)	21572407
MIRT175594	hsa-miR-20a-5p	OCRL	4952	HITS-CLIP	Functional MTI (Weak)	22473208
MIRT175644	hsa-miR-20a-5p	PHF6	84295	HITS-CLIP	Functional MTI (Weak)	22473208
MIRT176079	hsa-miR-20a-5p	CHIC1	53344	HITS-CLIP	Functional MTI (Weak)	22473208
MIRT178062	hsa-miR-20a-5p	SAMD8	142891	PAR-CLIP	Functional MTI (Weak)	21572407
MIRT182517	hsa-miR-20a-5p	ZBTB37	84614	PAR-CLIP	Functional MTI (Weak)	20371350
MIRT187472	hsa-miR-20a-5p	PCBP2	5094	PAR-CLIP	Functional MTI (Weak)	23446348
MIRT188183	hsa-miR-20a-5p	DYRK2	8445	PAR-CLIP	Functional MTI (Weak)	21572407
MIRT194849	hsa-miR-20a-5p	UBFD1	56061	HITS-CLIP	Functional MTI (Weak)	22473208
MIRT199106	hsa-miR-20a-5p	ZNF532	55205	PAR-CLIP	Functional MTI (Weak)	22012620
MIRT199283	hsa-miR-20a-5p	SH3GLB1	51100	PAR-CLIP	Functional MTI (Weak)	21572407
MIRT200924	hsa-miR-20a-5p	ZNF264	9422	PAR-CLIP	Functional MTI (Weak)	22012620
MIRT201019	hsa-miR-20a-5p	ZNF805	390980	PAR-CLIP	Functional MTI (Weak)	21572407
MIRT205046	hsa-miR-20a-5p	CREB1	1385	PAR-CLIP	Functional MTI (Weak)	23446348
MIRT205281	hsa-miR-20a-5p	STK11IP	114790	PAR-CLIP	Functional MTI (Weak)	21572407
MIRT206192	hsa-miR-20a-5p	RAB10	10890	PAR-CLIP	Functional MTI (Weak)	21572407
MIRT208975	hsa-miR-20a-5p	SKIL	6498	PAR-CLIP	Functional MTI (Weak)	24398324
MIRT213203	hsa-miR-20a-5p	REST	5978	PAR-CLIP	Functional MTI (Weak)	23446348
MIRT213321	hsa-miR-20a-5p	KIAA0232	9778	HITS-CLIP	Functional MTI (Weak)	23824327
MIRT216423	hsa-miR-20a-5p	SERF1A	8293	PAR-CLIP	Functional MTI (Weak)	22012620
MIRT216448	hsa-miR-20a-5p	SERF1B	728492	PAR-CLIP	Functional MTI (Weak)	22012620
MIRT216661	hsa-miR-20a-5p	F2R	2149	PAR-CLIP	Functional MTI (Weak)	20371350
MIRT220118	hsa-miR-20a-5p	CAV1	857	PAR-CLIP	Functional MTI (Weak)	20371350
MIRT222673	hsa-miR-20a-5p	EIF4H	7458	PAR-CLIP	Functional MTI (Weak)	22012620
MIRT222908	hsa-miR-20a-5p	CROT	54677	HITS-CLIP	Functional MTI (Weak)	22473208
MIRT224747	hsa-miR-20a-5p	DPYSL2	1808	PAR-CLIP	Functional MTI (Weak)	22012620
MIRT224885	hsa-miR-20a-5p	MAK16	84549	HITS-CLIP	Functional MTI (Weak)	22473208
MIRT227326	hsa-miR-20a-5p	TRIM32	22954	HITS-CLIP	Functional MTI (Weak)	22473208
MIRT230965	hsa-miR-20a-5p	PRRG4	79056	PAR-CLIP	Functional MTI (Weak)	20371350
MIRT238172	hsa-miR-20a-5p	ANKRD33B	651746	PAR-CLIP	Functional MTI (Weak)	23592263
MIRT241294	hsa-miR-20a-5p	ZC3H12C	85463	PAR-CLIP	Functional MTI (Weak)	23446348
MIRT242196	hsa-miR-20a-5p	TTC9	23508	PAR-CLIP	Functional MTI (Weak)	23446348
MIRT242657	hsa-miR-20a-5p	SALL3	27164	PAR-CLIP	Functional MTI (Weak)	22012620
MIRT243778	hsa-miR-20a-5p	AFF1	4299	HITS-CLIP	Functional MTI (Weak)	22473208
MIRT244588	hsa-miR-20a-5p	HOOK3	84376	HITS-CLIP	Functional MTI (Weak)	23313552
MIRT244945	hsa-miR-20a-5p	PRRG1	5638	In situ hybridization//Next Generation Sequencing (NGS)//qRT-PCR	Functional MTI (Weak)	26233958
MIRT246959	hsa-miR-20a-5p	TSKU	25987	PAR-CLIP	Functional MTI (Weak)	20371350
MIRT247079	hsa-miR-20a-5p	CEP57	9702	HITS-CLIP	Functional MTI (Weak)	22473208
MIRT248850	hsa-miR-20a-5p	SESN2	83667	HITS-CLIP	Functional MTI (Weak)	22473208
MIRT250444	hsa-miR-20a-5p	NFATC2IP	84901	HITS-CLIP	Functional MTI (Weak)	22473208
MIRT254248	hsa-miR-20a-5p	TRAPPC10	7109	HITS-CLIP	Functional MTI (Weak)	22473208
MIRT257279	hsa-miR-20a-5p	FOXC1	2296	PAR-CLIP	Functional MTI (Weak)	21572407
MIRT266048	hsa-miR-20a-5p	FJX1	24147	PAR-CLIP	Functional MTI (Weak)	21572407
MIRT266853	hsa-miR-20a-5p	SLC25A44	9673	PAR-CLIP	Functional MTI (Weak)	21572407
MIRT280207	hsa-miR-20a-5p	EIF2B2	8892	HITS-CLIP	Functional MTI (Weak)	23313552
MIRT280991	hsa-miR-20a-5p	SPRED1	161742	PAR-CLIP	Functional MTI (Weak)	23592263
MIRT283172	hsa-miR-20a-5p	C16orf52	730094	PAR-CLIP	Functional MTI (Weak)	21572407
MIRT286945	hsa-miR-20a-5p	SOCS7	30837	PAR-CLIP	Functional MTI (Weak)	21572407
MIRT289571	hsa-miR-20a-5p	KDM6B	23135	PAR-CLIP	Functional MTI (Weak)	20371350
MIRT291931	hsa-miR-20a-5p	TPM4	7171	PAR-CLIP	Functional MTI (Weak)	23592263
MIRT293645	hsa-miR-20a-5p	PVR	5817	PAR-CLIP	Functional MTI (Weak)	21572407
MIRT296868	hsa-miR-20a-5p	REV1	51455	HITS-CLIP	Functional MTI (Weak)	22473208
MIRT299337	hsa-miR-20a-5p	CYBRD1	79901	HITS-CLIP	Functional MTI (Weak)	22473208
MIRT302434	hsa-miR-20a-5p	CLIP4	79745	PAR-CLIP	Functional MTI (Weak)	20371350
MIRT303488	hsa-miR-20a-5p	NAGK	55577	PAR-CLIP	Functional MTI (Weak)	23446348
MIRT322520	hsa-miR-20a-5p	HMBOX1	79618	PAR-CLIP	Functional MTI (Weak)	22012620
MIRT325510	hsa-miR-20a-5p	PTPDC1	138639	PAR-CLIP	Functional MTI (Weak)	21572407
MIRT363922	hsa-miR-20a-5p	UBE2V2	7336	HITS-CLIP	Functional MTI (Weak)	23313552
MIRT368882	hsa-miR-20a-5p	BCL2L2	599	PAR-CLIP	Functional MTI (Weak)	23446348
MIRT397684	hsa-miR-20a-5p	ATXN7L3B	552889	PAR-CLIP	Functional MTI (Weak)	23592263
MIRT400046	hsa-miR-20a-5p	GRK3	157	HITS-CLIP	Functional MTI (Weak)	23824327
MIRT437765	hsa-miR-20a-5p	PRKG1	5592	Immunocytochemistry//Luciferase reporter assay//qRT-PCR//Western blot	Functional MTI	25447536

MIRT437944	hsa-miR-20a-5p	RGS5	8490	ChIP-seq//qRT-PCR//Luciferase reporter assay	Functional MTI	23308108
MIRT438054	hsa-miR-20a-5p	ETV1	2115	Luciferase reporter assay//qRT-PCR//microarray//Western blotting	Functional MTI	23969726
MIRT438160	hsa-miR-20a-5p	EPAS1	2034	Luciferase reporter assay	Functional MTI	24194900
MIRT438351	hsa-miR-20a-5p	FBXO31	79791	HITS-CLIP	Functional MTI (Weak)	22473208
MIRT438791	hsa-miR-20a-5p	TP53	7157	qRT-PCR	Functional MTI (Weak)	24955218
MIRT438806	hsa-miR-20a-5p	DNMT1	1786	Luciferase reporter assay	Functional MTI	23306545
MIRT438812	hsa-miR-20a-5p	PKD1	5310	Luciferase reporter assay	Functional MTI	23344677
MIRT439181	hsa-miR-20a-5p	ZNF800	168850	HITS-CLIP	Functional MTI (Weak)	22473208
MIRT439185	hsa-miR-20a-5p	ZNF770	54989	HITS-CLIP	Functional MTI (Weak)	22473208
MIRT439192	hsa-miR-20a-5p	ZNF597	146434	HITS-CLIP	Functional MTI (Weak)	22473208
MIRT439211	hsa-miR-20a-5p	ZNF280C	55609	HITS-CLIP	Functional MTI (Weak)	22473208
MIRT439214	hsa-miR-20a-5p	ZNF280B	140883	HITS-CLIP	Functional MTI (Weak)	22473208
MIRT439225	hsa-miR-20a-5p	ZNF12	7559	HITS-CLIP	Functional MTI (Weak)	22473208
MIRT439256	hsa-miR-20a-5p	ZBTB7A	51341	HITS-CLIP	Functional MTI (Weak)	22473208
MIRT439257	hsa-miR-20a-5p	ZBTB6	10773	HITS-CLIP	Functional MTI (Weak)	22473208
MIRT439259	hsa-miR-20a-5p	ZBTB4	57659	HITS-CLIP	Functional MTI (Weak)	22473208
MIRT439269	hsa-miR-20a-5p	YOD1	55432	HITS-CLIP	Functional MTI (Weak)	22473208
MIRT439286	hsa-miR-20a-5p	WDR89	112840	HITS-CLIP	Functional MTI (Weak)	22473208
MIRT439291	hsa-miR-20a-5p	WDR1	9948	HITS-CLIP	Functional MTI (Weak)	22473208
MIRT439295	hsa-miR-20a-5p	VTI1A	143187	HITS-CLIP	Functional MTI (Weak)	22473208
MIRT439300	hsa-miR-20a-5p	VPS13C	54832	HITS-CLIP	Functional MTI (Weak)	22473208
MIRT439306	hsa-miR-20a-5p	VDAC1	7416	HITS-CLIP	Functional MTI (Weak)	22473208
MIRT439319	hsa-miR-20a-5p	UXS1	80146	HITS-CLIP	Functional MTI (Weak)	22473208
MIRT439327	hsa-miR-20a-5p	USP32	84669	HITS-CLIP	Functional MTI (Weak)	22473208
MIRT439332	hsa-miR-20a-5p	USP28	57646	HITS-CLIP	Functional MTI (Weak)	22473208
MIRT439337	hsa-miR-20a-5p	USP16	10600	HITS-CLIP	Functional MTI (Weak)	22473208
MIRT439349	hsa-miR-20a-5p	UBR5	51366	HITS-CLIP	Functional MTI (Weak)	22473208
MIRT439366	hsa-miR-20a-5p	UBC	7316	HITS-CLIP	Functional MTI (Weak)	22473208
MIRT439372	hsa-miR-20a-5p	TWF1	5756	HITS-CLIP	Functional MTI (Weak)	22473208
MIRT439399	hsa-miR-20a-5p	TOPORS	10210	HITS-CLIP	Functional MTI (Weak)	22473208
MIRT439410	hsa-miR-20a-5p	TNFRSF21	27242	HITS-CLIP	Functional MTI (Weak)	22473208
MIRT439416	hsa-miR-20a-5p	TMX3	54495	HITS-CLIP	Functional MTI (Weak)	22473208
MIRT439423	hsa-miR-20a-5p	TMEM67	91147	HITS-CLIP	Functional MTI (Weak)	22473208
MIRT439427	hsa-miR-20a-5p	TMEM64	169200	HITS-CLIP	Functional MTI (Weak)	22473208
MIRT439435	hsa-miR-20a-5p	TMEM167A	153339	HITS-CLIP	Functional MTI (Weak)	22473208
MIRT439439	hsa-miR-20a-5p	TMEM127	55654	HITS-CLIP	Functional MTI (Weak)	22473208
MIRT439440	hsa-miR-20a-5p	TMEM123	114908	HITS-CLIP	Functional MTI (Weak)	22473208
MIRT439460	hsa-miR-20a-5p	TGOLN2	10618	HITS-CLIP	Functional MTI (Weak)	22473208
MIRT439478	hsa-miR-20a-5p	TCF4	6925	HITS-CLIP	Functional MTI (Weak)	22473208
MIRT439489	hsa-miR-20a-5p	TADA2B	93624	HITS-CLIP	Functional MTI (Weak)	22473208
MIRT439512	hsa-miR-20a-5p	STX6	10228	HITS-CLIP	Functional MTI (Weak)	22473208
MIRT439521	hsa-miR-20a-5p	STK17B	9262	HITS-CLIP	Functional MTI (Weak)	22473208
MIRT439535	hsa-miR-20a-5p	SSX2IP	117178	HITS-CLIP	Functional MTI (Weak)	22473208
MIRT439537	hsa-miR-20a-5p	SSH2	85464	HITS-CLIP	Functional MTI (Weak)	22473208
MIRT439566	hsa-miR-20a-5p	SOD2	6648	HITS-CLIP	Functional MTI (Weak)	22473208
MIRT439590	hsa-miR-20a-5p	SLK	9748	HITS-CLIP	Functional MTI (Weak)	22473208
MIRT439594	hsa-miR-20a-5p	SLC4A7	9497	HITS-CLIP	Functional MTI (Weak)	22473208
MIRT439606	hsa-miR-20a-5p	SLC35F5	80255	HITS-CLIP	Functional MTI (Weak)	22473208
MIRT439620	hsa-miR-20a-5p	SLC16A9	220963	HITS-CLIP	Functional MTI (Weak)	22473208
MIRT439633	hsa-miR-20a-5p	SIKE1	80143	HITS-CLIP	Functional MTI (Weak)	22473208
MIRT439644	hsa-miR-20a-5p	SGTB	54557	HITS-CLIP	Functional MTI (Weak)	22473208
MIRT439648	hsa-miR-20a-5p	PEAK1	79834	HITS-CLIP	Functional MTI (Weak)	22473208
MIRT439654	hsa-miR-20a-5p	SRSF2	6427	HITS-CLIP	Functional MTI (Weak)	22473208
MIRT439680	hsa-miR-20a-5p	SENP1	29843	HITS-CLIP	Functional MTI (Weak)	22473208
MIRT439688	hsa-miR-20a-5p	SEC23A	10484	HITS-CLIP	Functional MTI (Weak)	22473208
MIRT439692	hsa-miR-20a-5p	SEC16A	9919	HITS-CLIP	Functional MTI (Weak)	22473208
MIRT439703	hsa-miR-20a-5p	SCAMP2	10066	HITS-CLIP	Functional MTI (Weak)	22473208
MIRT439711	hsa-miR-20a-5p	SAMD9L	219285	HITS-CLIP	Functional MTI (Weak)	22473208
MIRT439715	hsa-miR-20a-5p	SACS	26278	HITS-CLIP	Functional MTI (Weak)	22473208
MIRT439742	hsa-miR-20a-5p	RPL17	6139	HITS-CLIP	Functional MTI (Weak)	22473208
MIRT439758	hsa-miR-20a-5p	RNF216	54476	HITS-CLIP	Functional MTI (Weak)	22473208
MIRT439776	hsa-miR-20a-5p	RFXANK	8625	HITS-CLIP	Functional MTI (Weak)	22473208
MIRT439786	hsa-miR-20a-5p	REEP5	7905	HITS-CLIP	Functional MTI (Weak)	22473208
MIRT439809	hsa-miR-20a-5p	RBBP7	5931	HITS-CLIP	Functional MTI (Weak)	22473208
MIRT439821	hsa-miR-20a-5p	RAN	5901	HITS-CLIP	Functional MTI (Weak)	22473208
MIRT439832	hsa-miR-20a-5p	RABEP1	9135	HITS-CLIP	Functional MTI (Weak)	22473208
MIRT439837	hsa-miR-20a-5p	RAB30	27314	HITS-CLIP	Functional MTI (Weak)	22473208
MIRT439848	hsa-miR-20a-5p	RAB11FIP1	80223	HITS-CLIP	Functional MTI (Weak)	22473208
MIRT439853	hsa-miR-20a-5p	PURB	5814	HITS-CLIP	Functional MTI (Weak)	22473208
MIRT439863	hsa-miR-20a-5p	PTPN4	5775	HITS-CLIP	Functional MTI (Weak)	22473208
MIRT439874	hsa-miR-20a-5p	PTGES3	10728	HITS-CLIP	Functional MTI (Weak)	22473208
MIRT439875	hsa-miR-20a-5p	PTGER4	5734	HITS-CLIP	Functional MTI (Weak)	22473208
MIRT439905	hsa-miR-20a-5p	PPP6C	5537	HITS-CLIP	Functional MTI (Weak)	22473208
MIRT439910	hsa-miR-20a-5p	PPP3R1	5534	HITS-CLIP	Functional MTI (Weak)	22473208
MIRT439915	hsa-miR-20a-5p	PPP1R3B	79660	HITS-CLIP	Functional MTI (Weak)	22473208
MIRT439933	hsa-miR-20a-5p	POLQ	10721	HITS-CLIP	Functional MTI (Weak)	22473208
MIRT439940	hsa-miR-20a-5p	PNPLA4	8228	HITS-CLIP	Functional MTI (Weak)	22473208
MIRT439954	hsa-miR-20a-5p	PLAGL2	5326	HITS-CLIP	Functional MTI (Weak)	22473208
MIRT439959	hsa-miR-20a-5p	PKMYT1	9088	HITS-CLIP	Functional MTI (Weak)	22473208
MIRT439971	hsa-miR-20a-5p	PIP4K2A	5305	HITS-CLIP	Functional MTI (Weak)	22473208
MIRT439977	hsa-miR-20a-5p	PIGO	84720	HITS-CLIP	Functional MTI (Weak)	22473208
MIRT439991	hsa-miR-20a-5p	PGM2L1	283209	HITS-CLIP	Functional MTI (Weak)	22473208
MIRT440006	hsa-miR-20a-5p	PDZD11	51248	HITS-CLIP	Functional MTI (Weak)	22473208
MIRT440025	hsa-miR-20a-5p	PCMTD1	115294	HITS-CLIP	Functional MTI (Weak)	22473208
MIRT440046	hsa-miR-20a-5p	PANK3	79646	HITS-CLIP	Functional MTI (Weak)	22473208
MIRT440068	hsa-miR-20a-5p	NUP98	4928	HITS-CLIP	Functional MTI (Weak)	22473208
MIRT440072	hsa-miR-20a-5p	NUP35	129401	HITS-CLIP	Functional MTI (Weak)	22473208
MIRT440093	hsa-miR-20a-5p	NR2C2	7182	HITS-CLIP	Functional MTI (Weak)	22473208
MIRT440099	hsa-miR-20a-5p	NPAT	4863	HITS-CLIP	Functional MTI (Weak)	22473208
MIRT440112	hsa-miR-20a-5p	NIPA1	123606	HITS-CLIP	Functional MTI (Weak)	22473208
MIRT440138	hsa-miR-20a-5p	NCAPD2	9918	HITS-CLIP	Functional MTI (Weak)	22473208
MIRT440145	hsa-miR-20a-5p	NAA50	80218	HITS-CLIP	Functional MTI (Weak)	22473208
MIRT440158	hsa-miR-20a-5p	N4BP1	9683	HITS-CLIP	Functional MTI (Weak)	22473208
MIRT440170	hsa-miR-20a-5p	MXI1	4601	HITS-CLIP	Functional MTI (Weak)	22473208
MIRT440208	hsa-miR-20a-5p	MTF1	4520	HITS-CLIP	Functional MTI (Weak)	22473208
MIRT440233	hsa-miR-20a-5p	MKRN1	23608	HITS-CLIP	Functional MTI (Weak)	22473208
MIRT440235	hsa-miR-20a-5p	MKNK2	2872	HITS-CLIP	Functional MTI (Weak)	22473208
MIRT440260	hsa-miR-20a-5p	MECP2	4204	HITS-CLIP	Functional MTI (Weak)	22473208
MIRT440278	hsa-miR-20a-5p	MAPK1	5594	HITS-CLIP	Functional MTI (Weak)	22473208
MIRT440284	hsa-miR-20a-5p	MAP3K2	10746	HITS-CLIP	Functional MTI (Weak)	22473208
MIRT440286	hsa-miR-20a-5p	MAP3K14	9020	HITS-CLIP	Functional MTI (Weak)	22473208
MIRT440296	hsa-miR-20a-5p	M6PR	4074	HITS-CLIP	Functional MTI (Weak)	22473208
MIRT440318	hsa-miR-20a-5p	LPGAT1	9926	HITS-CLIP	Functional MTI (Weak)	22473208
MIRT440325	hsa-miR-20a-5p	LIMA1	51474	HITS-CLIP	Functional MTI (Weak)	22473208
MIRT440340	hsa-miR-20a-5p	LAPTM4A	9741	HITS-CLIP	Functional MTI (Weak)	22473208

MIRT440346	hsa-miR-20a-5p	LAMC1	3915	HITS-CLIP	Functional MTI (Weak)	22473208
MIRT440357	hsa-miR-20a-5p	KLHL28	54813	HITS-CLIP	Functional MTI (Weak)	22473208
MIRT440369	hsa-miR-20a-5p	KIF23	9493	HITS-CLIP	Functional MTI (Weak)	22473208
MIRT440386	hsa-miR-20a-5p	CCSER2	54462	HITS-CLIP	Functional MTI (Weak)	22473208
MIRT440388	hsa-miR-20a-5p	ATG14	22863	HITS-CLIP	Functional MTI (Weak)	22473208
MIRT440391	hsa-miR-20a-5p	EFCAB14	9813	HITS-CLIP	Functional MTI (Weak)	22473208
MIRT440410	hsa-miR-20a-5p	KATNAL1	84056	HITS-CLIP	Functional MTI (Weak)	22473208
MIRT440417	hsa-miR-20a-5p	ITPKB	3707	HITS-CLIP	Functional MTI (Weak)	22473208
MIRT440427	hsa-miR-20a-5p	ITCH	83737	HITS-CLIP	Functional MTI (Weak)	22473208
MIRT440430	hsa-miR-20a-5p	IQSEC1	9922	HITS-CLIP	Functional MTI (Weak)	22473208
MIRT440451	hsa-miR-20a-5p	INPP5F	22876	HITS-CLIP	Functional MTI (Weak)	22473208
MIRT440469	hsa-miR-20a-5p	IER3	8870	HITS-CLIP	Functional MTI (Weak)	22473208
MIRT440506	hsa-miR-20a-5p	HIF1AN	55662	HITS-CLIP	Functional MTI (Weak)	22473208
MIRT440509	hsa-miR-20a-5p	HAUS8	93323	HITS-CLIP	Functional MTI (Weak)	22473208
MIRT440520	hsa-miR-20a-5p	HCP5	10866	HITS-CLIP	Functional MTI (Weak)	22473208
MIRT440537	hsa-miR-20a-5p	GPAM	57678	HITS-CLIP	Functional MTI (Weak)	22473208
MIRT440543	hsa-miR-20a-5p	GOLGA1	2800	HITS-CLIP	Functional MTI (Weak)	22473208
MIRT440557	hsa-miR-20a-5p	GNAS	2778	HITS-CLIP	Functional MTI (Weak)	22473208
MIRT440561	hsa-miR-20a-5p	GLO1	2739	HITS-CLIP	Functional MTI (Weak)	22473208
MIRT440573	hsa-miR-20a-5p	GIGYF1	64599	HITS-CLIP	Functional MTI (Weak)	22473208
MIRT440583	hsa-miR-20a-5p	GBP3	2635	HITS-CLIP	Functional MTI (Weak)	22473208
MIRT440590	hsa-miR-20a-5p	GAK	2580	HITS-CLIP	Functional MTI (Weak)	22473208
MIRT440593	hsa-miR-20a-5p	GABPB1	2553	HITS-CLIP	Functional MTI (Weak)	22473208
MIRT440595	hsa-miR-20a-5p	GABBR1	2550	HITS-CLIP	Functional MTI (Weak)	22473208
MIRT440601	hsa-miR-20a-5p	FYCO1	79443	HITS-CLIP	Functional MTI (Weak)	22473208
MIRT440609	hsa-miR-20a-5p	CMTR2	55783	HITS-CLIP	Functional MTI (Weak)	22473208
MIRT440633	hsa-miR-20a-5p	FMNL3	91010	HITS-CLIP	Functional MTI (Weak)	22473208
MIRT440646	hsa-miR-20a-5p	FEM1C	56929	HITS-CLIP	Functional MTI (Weak)	22473208
MIRT440653	hsa-miR-20a-5p	FBXO48	554251	HITS-CLIP	Functional MTI (Weak)	22473208
MIRT440657	hsa-miR-20a-5p	FBXO21	23014	HITS-CLIP	Functional MTI (Weak)	22473208
MIRT440660	hsa-miR-20a-5p	FBXO10	26267	HITS-CLIP	Functional MTI (Weak)	22473208
MIRT440662	hsa-miR-20a-5p	FBXL5	26234	HITS-CLIP	Functional MTI (Weak)	22473208
MIRT440674	hsa-miR-20a-5p	FAM83D	81610	HITS-CLIP	Functional MTI (Weak)	22473208
MIRT440677	hsa-miR-20a-5p	FAM57A	79850	HITS-CLIP	Functional MTI (Weak)	22473208
MIRT440685	hsa-miR-20a-5p	FAM129A	116496	HITS-CLIP	Functional MTI (Weak)	22473208
MIRT440686	hsa-miR-20a-5p	FAM126B	285172	HITS-CLIP	Functional MTI (Weak)	22473208
MIRT440693	hsa-miR-20a-5p	FAM102A	399665	HITS-CLIP	Functional MTI (Weak)	22473208
MIRT440699	hsa-miR-20a-5p	EZH1	2145	HITS-CLIP	Functional MTI (Weak)	22473208
MIRT440703	hsa-miR-20a-5p	ETF1	2107	HITS-CLIP	Functional MTI (Weak)	22473208
MIRT440712	hsa-miR-20a-5p	ERAP1	51752	HITS-CLIP	Functional MTI (Weak)	22473208
MIRT440720	hsa-miR-20a-5p	ENTPD7	57089	HITS-CLIP	Functional MTI (Weak)	22473208
MIRT440729	hsa-miR-20a-5p	EIF5A2	56648	HITS-CLIP	Functional MTI (Weak)	22473208
MIRT440752	hsa-miR-20a-5p	EEA1	8411	HITS-CLIP	Functional MTI (Weak)	22473208
MIRT440755	hsa-miR-20a-5p	E2F2	1870	HITS-CLIP	Functional MTI (Weak)	22473208
MIRT440759	hsa-miR-20a-5p	DYNC1LI2	1783	HITS-CLIP	Functional MTI (Weak)	22473208
MIRT440767	hsa-miR-20a-5p	DUSP18	150290	HITS-CLIP	Functional MTI (Weak)	22473208
MIRT440797	hsa-miR-20a-5p	DNAJC27	51277	HITS-CLIP	Functional MTI (Weak)	22473208
MIRT440826	hsa-miR-20a-5p	DENND5B	160518	HITS-CLIP	Functional MTI (Weak)	22473208
MIRT440840	hsa-miR-20a-5p	DDHD1	80821	HITS-CLIP	Functional MTI (Weak)	22473208
MIRT440857	hsa-miR-20a-5p	CTSS	1520	HITS-CLIP	Functional MTI (Weak)	22473208
MIRT440873	hsa-miR-20a-5p	CRTC3	64784	HITS-CLIP	Functional MTI (Weak)	22473208
MIRT440876	hsa-miR-20a-5p	CRK	1398	HITS-CLIP	Functional MTI (Weak)	22473208
MIRT440886	hsa-miR-20a-5p	CPOX	1371	HITS-CLIP	Functional MTI (Weak)	22473208
MIRT440918	hsa-miR-20a-5p	CNOT7	29883	HITS-CLIP	Functional MTI (Weak)	22473208
MIRT440933	hsa-miR-20a-5p	CLOCK	9575	HITS-CLIP	Functional MTI (Weak)	22473208
MIRT440941	hsa-miR-20a-5p	CIT	11113	HITS-CLIP	Functional MTI (Weak)	22473208
MIRT440943	hsa-miR-20a-5p	CHURC1	91612	HITS-CLIP	Functional MTI (Weak)	22473208
MIRT440953	hsa-miR-20a-5p	CFL2	1073	HITS-CLIP	Functional MTI (Weak)	22473208
MIRT440954	hsa-miR-20a-5p	CEP97	79598	HITS-CLIP	Functional MTI (Weak)	22473208
MIRT440976	hsa-miR-20a-5p	CD47	961	HITS-CLIP	Functional MTI (Weak)	22473208
MIRT440986	hsa-miR-20a-5p	CCL1	6346	HITS-CLIP	Functional MTI (Weak)	22473208
MIRT441010	hsa-miR-20a-5p	CAPRIN2	65981	HITS-CLIP	Functional MTI (Weak)	22473208
MIRT441022	hsa-miR-20a-5p	CAMK2N2	94032	HITS-CLIP	Functional MTI (Weak)	22473208
MIRT441030	hsa-miR-20a-5p	TMEM245	23731	HITS-CLIP	Functional MTI (Weak)	22473208
MIRT441031	hsa-miR-20a-5p	C9orf40	55071	HITS-CLIP	Functional MTI (Weak)	22473208
MIRT441033	hsa-miR-20a-5p	BMT2	154743	HITS-CLIP	Functional MTI (Weak)	22473208
MIRT441036	hsa-miR-20a-5p	C7orf43	55262	HITS-CLIP	Functional MTI (Weak)	22473208
MIRT441043	hsa-miR-20a-5p	TMEM267	64417	HITS-CLIP	Functional MTI (Weak)	22473208
MIRT441050	hsa-miR-20a-5p	PRR14L	253143	HITS-CLIP	Functional MTI (Weak)	22473208
MIRT441055	hsa-miR-20a-5p	SUCO	51430	HITS-CLIP	Functional MTI (Weak)	22473208
MIRT441057	hsa-miR-20a-5p	RSRP1	57035	HITS-CLIP	Functional MTI (Weak)	22473208
MIRT441073	hsa-miR-20a-5p	FAM210A	125228	HITS-CLIP	Functional MTI (Weak)	22473208
MIRT441080	hsa-miR-20a-5p	ELMSAN1	91748	HITS-CLIP	Functional MTI (Weak)	22473208
MIRT441082	hsa-miR-20a-5p	C14orf28	122525	HITS-CLIP	Functional MTI (Weak)	22473208
MIRT441084	hsa-miR-20a-5p	VCPKMT	79609	HITS-CLIP	Functional MTI (Weak)	22473208
MIRT441088	hsa-miR-20a-5p	EMSY	56946	HITS-CLIP	Functional MTI (Weak)	22473208
MIRT441094	hsa-miR-20a-5p	BTN3A3	10384	HITS-CLIP	Functional MTI (Weak)	22473208
MIRT441097	hsa-miR-20a-5p	BTBD7	55727	HITS-CLIP	Functional MTI (Weak)	22473208
MIRT441126	hsa-miR-20a-5p	BAGE5	85316	HITS-CLIP	Functional MTI (Weak)	22473208
MIRT441138	hsa-miR-20a-5p	ATXN1	6310	HITS-CLIP	Functional MTI (Weak)	22473208
MIRT441150	hsa-miR-20a-5p	ATP2B1	490	HITS-CLIP	Functional MTI (Weak)	22473208
MIRT441164	hsa-miR-20a-5p	ATG2B	55102	HITS-CLIP	Functional MTI (Weak)	22473208
MIRT441167	hsa-miR-20a-5p	ATG2A	23130	HITS-CLIP	Functional MTI (Weak)	22473208
MIRT441187	hsa-miR-20a-5p	ARL1	400	HITS-CLIP	Functional MTI (Weak)	22473208
MIRT441189	hsa-miR-20a-5p	ARID4B	51742	HITS-CLIP	Functional MTI (Weak)	22473208
MIRT441201	hsa-miR-20a-5p	ARHGAP1	392	HITS-CLIP	Functional MTI (Weak)	22473208
MIRT441213	hsa-miR-20a-5p	ARAP2	116984	HITS-CLIP	Functional MTI (Weak)	22473208
MIRT441217	hsa-miR-20a-5p	AP1G1	164	HITS-CLIP	Functional MTI (Weak)	22473208
MIRT441223	hsa-miR-20a-5p	ANKRD52	283373	HITS-CLIP	Functional MTI (Weak)	22473208
MIRT441227	hsa-miR-20a-5p	ANKRD13C	81573	HITS-CLIP	Functional MTI (Weak)	22473208
MIRT441232	hsa-miR-20a-5p	ANKFY1	51479	HITS-CLIP	Functional MTI (Weak)	22473208
MIRT441235	hsa-miR-20a-5p	ALDH9A1	223	HITS-CLIP	Functional MTI (Weak)	22473208
MIRT441239	hsa-miR-20a-5p	AKTIP	64400	HITS-CLIP	Functional MTI (Weak)	22473208
MIRT441293	hsa-miR-20a-5p	ACBD5	91452	HITS-CLIP	Functional MTI (Weak)	22473208
MIRT441296	hsa-miR-20a-5p	ACAP2	23527	HITS-CLIP	Functional MTI (Weak)	22473208
MIRT441312	hsa-miR-20a-5p	ABCA1	19	HITS-CLIP	Functional MTI (Weak)	22473208
MIRT441316	hsa-miR-20a-5p	AAK1	22848	HITS-CLIP	Functional MTI (Weak)	22473208
MIRT441880	hsa-miR-20a-5p	PFKFB2	5208	PAR-CLIP	Functional MTI (Weak)	22100165
MIRT442202	hsa-miR-20a-5p	VPS50	55610	PAR-CLIP	Functional MTI (Weak)	22100165
MIRT442551	hsa-miR-20a-5p	SLCO5A1	81796	PAR-CLIP	Functional MTI (Weak)	22100165
MIRT442768	hsa-miR-20a-5p	NRIP3	56675	PAR-CLIP	Functional MTI (Weak)	22100165
MIRT442802	hsa-miR-20a-5p	CEP170	9859	PAR-CLIP	Functional MTI (Weak)	22100165
MIRT443258	hsa-miR-20a-5p	A1CF	29974	PAR-CLIP	Functional MTI (Weak)	22100165
MIRT443712	hsa-miR-20a-5p	LLPH	84298	PAR-CLIP	Functional MTI (Weak)	22100165
MIRT444311	hsa-miR-20a-5p	SREK1IP1	285672	PAR-CLIP	Functional MTI (Weak)	22100165

MIRT444437	hsa-miR-20a-5p	EMC1	23065	PAR-CLIP	Functional MTI (Weak)	22100165
MIRT448315	hsa-miR-20a-5p	WNK3	65267	PAR-CLIP	Functional MTI (Weak)	22100165
MIRT448365	hsa-miR-20a-5p	TSR1	55720	PAR-CLIP	Functional MTI (Weak)	22100165
MIRT448645	hsa-miR-20a-5p	NPNT	255743	PAR-CLIP	Functional MTI (Weak)	22100165
MIRT448728	hsa-miR-20a-5p	ITGA2	3673	PAR-CLIP	Functional MTI (Weak)	22100165
MIRT449173	hsa-miR-20a-5p	SORCS2	57537	PAR-CLIP	Functional MTI (Weak)	22100165
MIRT450189	hsa-miR-20a-5p	TMEM9B	56674	PAR-CLIP	Functional MTI (Weak)	22100165
MIRT450915	hsa-miR-20a-5p	CADM2	253559	PAR-CLIP	Functional MTI (Weak)	22100165
MIRT450954	hsa-miR-20a-5p	ATAD2	29028	PAR-CLIP	Functional MTI (Weak)	22100165
MIRT458293	hsa-miR-20a-5p	FUT10	84750	PAR-CLIP	Functional MTI (Weak)	23592263
MIRT463554	hsa-miR-20a-5p	ZBTB5	9925	PAR-CLIP	Functional MTI (Weak)	23592263
MIRT464847	hsa-miR-20a-5p	RPS27A	6233	PAR-CLIP	Functional MTI (Weak)	23592263
MIRT465236	hsa-miR-20a-5p	TRIP10	9322	PAR-CLIP	Functional MTI (Weak)	23592263
MIRT465539	hsa-miR-20a-5p	PRICKLE4	29964	PAR-CLIP	Functional MTI (Weak)	23592263
MIRT466455	hsa-miR-20a-5p	TFAM	7019	PAR-CLIP	Functional MTI (Weak)	23592263
MIRT467495	hsa-miR-20a-5p	SMIM13	221710	PAR-CLIP	Functional MTI (Weak)	23592263
MIRT467895	hsa-miR-20a-5p	SLC22A23	63027	PAR-CLIP	Functional MTI (Weak)	23592263
MIRT468161	hsa-miR-20a-5p	SGPL1	8879	PAR-CLIP	Functional MTI (Weak)	23592263
MIRT468185	hsa-miR-20a-5p	SGMS1	259230	PAR-CLIP	Functional MTI (Weak)	23592263
MIRT469861	hsa-miR-20a-5p	PXK	54899	PAR-CLIP	Functional MTI (Weak)	23592263
MIRT470052	hsa-miR-20a-5p	PTGFRN	5738	PAR-CLIP	Functional MTI (Weak)	23592263
MIRT471004	hsa-miR-20a-5p	PITPNA	5306	PAR-CLIP	Functional MTI (Weak)	23592263
MIRT472168	hsa-miR-20a-5p	NIN	51199	PAR-CLIP	Functional MTI (Weak)	23592263
MIRT472288	hsa-miR-20a-5p	NFIB	4781	PAR-CLIP	Functional MTI (Weak)	23592263
MIRT473167	hsa-miR-20a-5p	MLLT1	4298	PAR-CLIP	Functional MTI (Weak)	23592263
MIRT474598	hsa-miR-20a-5p	KLF6	1316	PAR-CLIP	Functional MTI (Weak)	23592263
MIRT475410	hsa-miR-20a-5p	ICMT	23463	PAR-CLIP	Functional MTI (Weak)	23592263
MIRT475477	hsa-miR-20a-5p	HSPA8	3312	PAR-CLIP	Functional MTI (Weak)	23592263
MIRT476130	hsa-miR-20a-5p	GPR157	80045	PAR-CLIP	Functional MTI (Weak)	23592263
MIRT477116	hsa-miR-20a-5p	FAM160B1	57700	PAR-CLIP	Functional MTI (Weak)	23592263
MIRT477193	hsa-miR-20a-5p	F3	2152	PAR-CLIP	Functional MTI (Weak)	23592263
MIRT477286	hsa-miR-20a-5p	ERGIC2	51290	PAR-CLIP	Functional MTI (Weak)	23592263
MIRT478722	hsa-miR-20a-5p	CSNK1A1	1452	PAR-CLIP	Functional MTI (Weak)	23592263
MIRT479040	hsa-miR-20a-5p	COIL	8161	PAR-CLIP	Functional MTI (Weak)	23592263
MIRT479064	hsa-miR-20a-5p	CNOT6L	246175	PAR-CLIP	Functional MTI (Weak)	23592263
MIRT479268	hsa-miR-20a-5p	CHSY1	22856	PAR-CLIP	Functional MTI (Weak)	23592263
MIRT480567	hsa-miR-20a-5p	BZW1	9689	PAR-CLIP	Functional MTI (Weak)	23592263
MIRT480676	hsa-miR-20a-5p	BSCL2	26580	PAR-CLIP	Functional MTI (Weak)	23592263
MIRT480786	hsa-miR-20a-5p	BMP2	650	PAR-CLIP	Functional MTI (Weak)	23592263
MIRT480953	hsa-miR-20a-5p	BBX	56987	PAR-CLIP	Functional MTI (Weak)	23592263
MIRT481882	hsa-miR-20a-5p	ANKRD50	57182	PAR-CLIP	Functional MTI (Weak)	23592263
MIRT482138	hsa-miR-20a-5p	AKAP11	11215	PAR-CLIP	Functional MTI (Weak)	23592263
MIRT482477	hsa-miR-20a-5p	ADAR	103	PAR-CLIP	Functional MTI (Weak)	23592263
MIRT484868	hsa-miR-20a-5p	ZNF70	7621	PAR-CLIP	Functional MTI (Weak)	23592263
MIRT484885	hsa-miR-20a-5p	ZNF652	22834	PAR-CLIP	Functional MTI (Weak)	23592263
MIRT484922	hsa-miR-20a-5p	ZFYVE26	23503	PAR-CLIP	Functional MTI (Weak)	23592263
MIRT485095	hsa-miR-20a-5p	SLC30A1	7779	PAR-CLIP	Functional MTI (Weak)	23592263
MIRT485193	hsa-miR-20a-5p	PTP4A1	7803	PAR-CLIP	Functional MTI (Weak)	23592263
MIRT485331	hsa-miR-20a-5p	MYO1D	4642	PAR-CLIP	Functional MTI (Weak)	23592263
MIRT485368	hsa-miR-20a-5p	MYLIP	29116	PAR-CLIP	Functional MTI (Weak)	23592263
MIRT485588	hsa-miR-20a-5p	FOXQ1	94234	PAR-CLIP	Functional MTI (Weak)	23592263
MIRT486029	hsa-miR-20a-5p	LPAR2	9170	PAR-CLIP	Functional MTI (Weak)	23592263
MIRT486757	hsa-miR-20a-5p	CNOT4	4850	PAR-CLIP	Functional MTI (Weak)	23592263
MIRT489607	hsa-miR-20a-5p	ZDHHC20	253832	PAR-CLIP	Functional MTI (Weak)	23592263
MIRT491660	hsa-miR-20a-5p	PDRG1	81572	PAR-CLIP	Functional MTI (Weak)	23592263
MIRT491807	hsa-miR-20a-5p	ZFYVE21	79038	PAR-CLIP	Functional MTI (Weak)	23592263
MIRT492011	hsa-miR-20a-5p	UGCG	7357	PAR-CLIP	Functional MTI (Weak)	23592263
MIRT492377	hsa-miR-20a-5p	SEMA7A	8482	PAR-CLIP	Functional MTI (Weak)	23592263
MIRT492786	hsa-miR-20a-5p	PDGFB	5155	PAR-CLIP	Functional MTI (Weak)	23592263
MIRT493619	hsa-miR-20a-5p	HMGB3	3149	PAR-CLIP	Functional MTI (Weak)	23592263
MIRT494427	hsa-miR-20a-5p	BTG2	7832	PAR-CLIP	Functional MTI (Weak)	23592263
MIRT496064	hsa-miR-20a-5p	MORC1	27136	PAR-CLIP	Functional MTI (Weak)	22291592
MIRT500724	hsa-miR-20a-5p	TRIM37	4591	PAR-CLIP	Functional MTI (Weak)	24398324
MIRT502008	hsa-miR-20a-5p	MAP7	9053	PAR-CLIP	Functional MTI (Weak)	24398324
MIRT503213	hsa-miR-20a-5p	ACER2	340485	PAR-CLIP	Functional MTI (Weak)	24398324
MIRT503562	hsa-miR-20a-5p	MDM2	4193	PAR-CLIP	Functional MTI (Weak)	23446348
MIRT503610	hsa-miR-20a-5p	ZNF780A	284323	PAR-CLIP	Functional MTI (Weak)	23446348
MIRT503829	hsa-miR-20a-5p	TMEM242	729515	PAR-CLIP	Functional MTI (Weak)	23446348
MIRT503967	hsa-miR-20a-5p	ZNF180	7733	PAR-CLIP	Functional MTI (Weak)	23446348
MIRT504566	hsa-miR-20a-5p	ZNF417	147687	PAR-CLIP	Functional MTI (Weak)	23446348
MIRT504641	hsa-miR-20a-5p	MFSD8	256471	PAR-CLIP	Functional MTI (Weak)	23446348
MIRT505060	hsa-miR-20a-5p	ZNF202	7753	PAR-CLIP	Functional MTI (Weak)	23446348
MIRT505866	hsa-miR-20a-5p	POLR1B	84172	PAR-CLIP	Functional MTI (Weak)	23446348
MIRT506282	hsa-miR-20a-5p	PDPK1	5170	PAR-CLIP	Functional MTI (Weak)	23446348
MIRT506378	hsa-miR-20a-5p	NUFIP2	57532	PAR-CLIP	Functional MTI (Weak)	23446348
MIRT506458	hsa-miR-20a-5p	NACC2	138151	PAR-CLIP	Functional MTI (Weak)	23446348
MIRT506547	hsa-miR-20a-5p	MORF4L1	10933	PAR-CLIP	Functional MTI (Weak)	23446348
MIRT506622	hsa-miR-20a-5p	MARCH6	10299	PAR-CLIP	Functional MTI (Weak)	23446348
MIRT506685	hsa-miR-20a-5p	LZIC	84328	PAR-CLIP	Functional MTI (Weak)	23446348
MIRT506873	hsa-miR-20a-5p	KIAA1147	57189	PAR-CLIP	Functional MTI (Weak)	23446348
MIRT506887	hsa-miR-20a-5p	PCLAF	9768	PAR-CLIP	Functional MTI (Weak)	23446348
MIRT506991	hsa-miR-20a-5p	HNRNPR	10236	PAR-CLIP	Functional MTI (Weak)	23446348
MIRT507203	hsa-miR-20a-5p	FZD9	8326	PAR-CLIP	Functional MTI (Weak)	23446348
MIRT507440	hsa-miR-20a-5p	ELK4	2005	PAR-CLIP	Functional MTI (Weak)	23446348
MIRT507947	hsa-miR-20a-5p	BTF3L4	91408	PAR-CLIP	Functional MTI (Weak)	23446348
MIRT508576	hsa-miR-20a-5p	CEP72	55722	PAR-CLIP	Functional MTI (Weak)	23446348
MIRT508745	hsa-miR-20a-5p	ZNF682	91120	PAR-CLIP	Functional MTI (Weak)	23446348
MIRT508837	hsa-miR-20a-5p	GPR155	151556	PAR-CLIP	Functional MTI (Weak)	23446348
MIRT509130	hsa-miR-20a-5p	BMP8B	656	PAR-CLIP	Functional MTI (Weak)	23446348
MIRT509745	hsa-miR-20a-5p	EFCAB11	90141	PAR-CLIP	Functional MTI (Weak)	23446348
MIRT510029	hsa-miR-20a-5p	CRISPLD2	83716	PAR-CLIP	Functional MTI (Weak)	23446348
MIRT511267	hsa-miR-20a-5p	KLHL36	79786	PAR-CLIP	Functional MTI (Weak)	23446348
MIRT511320	hsa-miR-20a-5p	KIAA1551	55196	PAR-CLIP	Functional MTI (Weak)	23446348
MIRT511556	hsa-miR-20a-5p	HMGB1	3146	PAR-CLIP	Functional MTI (Weak)	23446348
MIRT512322	hsa-miR-20a-5p	ACTR2	10097	PAR-CLIP	Functional MTI (Weak)	23446348
MIRT513467	hsa-miR-20a-5p	NARS	4677	PAR-CLIP	Functional MTI (Weak)	23446348
MIRT513709	hsa-miR-20a-5p	RBM20	282996	PAR-CLIP	Functional MTI (Weak)	23446348
MIRT513751	hsa-miR-20a-5p	PKNOX1	5316	PAR-CLIP	Functional MTI (Weak)	23446348
MIRT514101	hsa-miR-20a-5p	EPS15L1	58513	PAR-CLIP	Functional MTI (Weak)	23446348
MIRT514137	hsa-miR-20a-5p	SERF2	10169	PAR-CLIP	Functional MTI (Weak)	23446348
MIRT514320	hsa-miR-20a-5p	FXYD5	53827	PAR-CLIP	Functional MTI (Weak)	23446348
MIRT514998	hsa-miR-20a-5p	DNTTIP2	30836	PAR-CLIP	Functional MTI (Weak)	23446348
MIRT515205	hsa-miR-20a-5p	CRCP	27297	PAR-CLIP	Functional MTI (Weak)	23446348
MIRT515575	hsa-miR-20a-5p	TMEM134	80194	PAR-CLIP	Functional MTI (Weak)	23446348
MIRT516060	hsa-miR-20a-5p	MED18	54797	PAR-CLIP	Functional MTI (Weak)	23446348

MIRT516559	hsa-miR-20a-5p	MIXL1	83881	PAR-CLIP	Functional MTI (Weak)	23446348
MIRT516799	hsa-miR-20a-5p	CAVIN1	284119	PAR-CLIP	Functional MTI (Weak)	23446348
MIRT517021	hsa-miR-20a-5p	COX19	90639	PAR-CLIP	Functional MTI (Weak)	23446348
MIRT517187	hsa-miR-20a-5p	SLC28A1	9154	PAR-CLIP	Functional MTI (Weak)	23446348
MIRT517260	hsa-miR-20a-5p	PRIM1	5557	PAR-CLIP	Functional MTI (Weak)	23446348
MIRT518317	hsa-miR-20a-5p	ZNF514	84874	PAR-CLIP	Functional MTI (Weak)	23446348
MIRT518468	hsa-miR-20a-5p	KIF6	221458	PAR-CLIP	Functional MTI (Weak)	23446348
MIRT518699	hsa-miR-20a-5p	KCNMB1	3779	PAR-CLIP	Functional MTI (Weak)	23446348
MIRT518804	hsa-miR-20a-5p	MED16	10025	PAR-CLIP	Functional MTI (Weak)	23446348
MIRT518863	hsa-miR-20a-5p	NEK8	284086	PAR-CLIP	Functional MTI (Weak)	23446348
MIRT518972	hsa-miR-20a-5p	GRK7	131890	PAR-CLIP	Functional MTI (Weak)	23446348
MIRT519432	hsa-miR-20a-5p	KCNA7	3743	PAR-CLIP	Functional MTI (Weak)	23446348
MIRT519548	hsa-miR-20a-5p	TMEM38A	79041	PAR-CLIP	Functional MTI (Weak)	23446348
MIRT520082	hsa-miR-20a-5p	YIPF4	84272	PAR-CLIP	Functional MTI (Weak)	23446348
MIRT520156	hsa-miR-20a-5p	WSB1	26118	PAR-CLIP	Functional MTI (Weak)	23446348
MIRT521010	hsa-miR-20a-5p	SOCS5	9655	PAR-CLIP	Functional MTI (Weak)	23446348
MIRT521304	hsa-miR-20a-5p	RRAGD	58528	PAR-CLIP	Functional MTI (Weak)	23446348
MIRT521549	hsa-miR-20a-5p	QSOX1	5768	PAR-CLIP	Functional MTI (Weak)	23446348
MIRT522172	hsa-miR-20a-5p	NR2F6	2063	PAR-CLIP	Functional MTI (Weak)	23446348
MIRT522505	hsa-miR-20a-5p	MFN1	55669	PAR-CLIP	Functional MTI (Weak)	23446348
MIRT523083	hsa-miR-20a-5p	HYPK	25764	PAR-CLIP	Functional MTI (Weak)	23446348
MIRT523654	hsa-miR-20a-5p	FOXK1	221937	PAR-CLIP	Functional MTI (Weak)	23446348
MIRT524083	hsa-miR-20a-5p	DNAJC10	54431	PAR-CLIP	Functional MTI (Weak)	23446348
MIRT524251	hsa-miR-20a-5p	DCTN6	10671	PAR-CLIP	Functional MTI (Weak)	23446348
MIRT524295	hsa-miR-20a-5p	CYCS	54205	PAR-CLIP	Functional MTI (Weak)	23446348
MIRT524457	hsa-miR-20a-5p	CNKSR3	154043	PAR-CLIP	Functional MTI (Weak)	23446348
MIRT524706	hsa-miR-20a-5p	BTG3	10950	PAR-CLIP	Functional MTI (Weak)	23446348
MIRT524984	hsa-miR-20a-5p	AGO3	192669	PAR-CLIP	Functional MTI (Weak)	23446348
MIRT525204	hsa-miR-20a-5p	ZNF93	81931	PAR-CLIP	Functional MTI (Weak)	22012620
MIRT525488	hsa-miR-20a-5p	TPK1	27010	PAR-CLIP	Functional MTI (Weak)	22012620
MIRT526639	hsa-miR-20a-5p	NME6	10201	PAR-CLIP	Functional MTI (Weak)	22012620
MIRT527206	hsa-miR-20a-5p	XIRP2	129446	PAR-CLIP	Functional MTI (Weak)	22012620
MIRT527259	hsa-miR-20a-5p	TMEM196	256130	PAR-CLIP	Functional MTI (Weak)	22012620
MIRT527474	hsa-miR-20a-5p	CLEC12B	387837	PAR-CLIP	Functional MTI (Weak)	22012620
MIRT528374	hsa-miR-20a-5p	ZMYM1	79830	PAR-CLIP	Functional MTI (Weak)	22012620
MIRT530003	hsa-miR-20a-5p	TNFAIP8L1	126282	PAR-CLIP	Functional MTI (Weak)	22012620
MIRT530591	hsa-miR-20a-5p	ABHD15	116236	PAR-CLIP	Functional MTI (Weak)	22012620
MIRT530989	hsa-miR-20a-5p	EXO5	64789	PAR-CLIP	Functional MTI (Weak)	22012620
MIRT531480	hsa-miR-20a-5p	TNFRSF10B	8795	PAR-CLIP	Functional MTI (Weak)	22012620
MIRT531779	hsa-miR-20a-5p	TXK	7294	PAR-CLIP	Functional MTI (Weak)	22012620
MIRT531839	hsa-miR-20a-5p	MTPAP	55149	PAR-CLIP	Functional MTI (Weak)	22012620
MIRT532053	hsa-miR-20a-5p	FHDC1	85462	PAR-CLIP	Functional MTI (Weak)	22012620
MIRT532618	hsa-miR-20a-5p	SPTLC2	9517	PAR-CLIP	Functional MTI (Weak)	22012620
MIRT532922	hsa-miR-20a-5p	ZNF385A	25946	PAR-CLIP	Functional MTI (Weak)	22012620
MIRT533881	hsa-miR-20a-5p	TBL1XR1	79718	PAR-CLIP	Functional MTI (Weak)	22012620
MIRT534008	hsa-miR-20a-5p	KMT5B	51111	PAR-CLIP	Functional MTI (Weak)	22012620
MIRT534573	hsa-miR-20a-5p	RPS6KA5	9252	PAR-CLIP	Functional MTI (Weak)	22012620
MIRT534631	hsa-miR-20a-5p	RNASEH1	246243	PAR-CLIP	Functional MTI (Weak)	22012620
MIRT535033	hsa-miR-20a-5p	PRKAR1A	5573	PAR-CLIP	Functional MTI (Weak)	22012620
MIRT536446	hsa-miR-20a-5p	KMT2B	9757	PAR-CLIP	Functional MTI (Weak)	22012620
MIRT536503	hsa-miR-20a-5p	TMEM131L	23240	PAR-CLIP	Functional MTI (Weak)	22012620
MIRT536542	hsa-miR-20a-5p	KCNJ8	3764	PAR-CLIP	Functional MTI (Weak)	22012620
MIRT537450	hsa-miR-20a-5p	FBXL7	23194	PAR-CLIP	Functional MTI (Weak)	22012620
MIRT537742	hsa-miR-20a-5p	ELAVL2	1993	PAR-CLIP	Functional MTI (Weak)	22012620
MIRT538040	hsa-miR-20a-5p	DNAJB6	10049	PAR-CLIP	Functional MTI (Weak)	22012620
MIRT538070	hsa-miR-20a-5p	DIAPH2	1730	PAR-CLIP	Functional MTI (Weak)	22012620
MIRT539427	hsa-miR-20a-5p	ADAT2	134637	PAR-CLIP	Functional MTI (Weak)	22012620
MIRT540278	hsa-miR-20a-5p	FAM89A	375061	PAR-CLIP	Functional MTI (Weak)	21572407
MIRT541093	hsa-miR-20a-5p	RLIM	51132	PAR-CLIP	Functional MTI (Weak)	21572407
MIRT542063	hsa-miR-20a-5p	SLC25A46	91137	PAR-CLIP	Functional MTI (Weak)	21572407
MIRT542145	hsa-miR-20a-5p	DIS3L	115752	PAR-CLIP	Functional MTI (Weak)	21572407
MIRT542271	hsa-miR-20a-5p	HSPA4L	22824	PAR-CLIP	Functional MTI (Weak)	21572407
MIRT543188	hsa-miR-20a-5p	FICD	11153	PAR-CLIP	Functional MTI (Weak)	21572407
MIRT543511	hsa-miR-20a-5p	PLS1	5357	PAR-CLIP	Functional MTI (Weak)	21572407
MIRT543583	hsa-miR-20a-5p	RPF2	84154	PAR-CLIP	Functional MTI (Weak)	21572407
MIRT544632	hsa-miR-20a-5p	CSDE1	7812	PAR-CLIP	Functional MTI (Weak)	21572407
MIRT545206	hsa-miR-20a-5p	HIST1H2BD	3017	PAR-CLIP	Functional MTI (Weak)	21572407
MIRT546299	hsa-miR-20a-5p	TMEM200C	645369	PAR-CLIP	Functional MTI (Weak)	21572407
MIRT546694	hsa-miR-20a-5p	RORA	6095	PAR-CLIP	Functional MTI (Weak)	21572407
MIRT546827	hsa-miR-20a-5p	RAP2C	57826	PAR-CLIP	Functional MTI (Weak)	21572407
MIRT547085	hsa-miR-20a-5p	PLRG1	5356	PAR-CLIP	Functional MTI (Weak)	21572407
MIRT548324	hsa-miR-20a-5p	EPHA4	2043	PAR-CLIP	Functional MTI (Weak)	21572407
MIRT548396	hsa-miR-20a-5p	ENPP5	59084	PAR-CLIP	Functional MTI (Weak)	21572407
MIRT548822	hsa-miR-20a-5p	CLIC4	25932	PAR-CLIP	Functional MTI (Weak)	21572407
MIRT548867	hsa-miR-20a-5p	CERCAM	51148	PAR-CLIP//HITS-CLIP	Functional MTI (Weak)	21572407
MIRT549113	hsa-miR-20a-5p	C16orf70	80262	PAR-CLIP	Functional MTI (Weak)	21572407
MIRT549826	hsa-miR-20a-5p	LUZP2	338645	PAR-CLIP	Functional MTI (Weak)	21572407
MIRT550097	hsa-miR-20a-5p	TRAPPC2	6399	PAR-CLIP	Functional MTI (Weak)	21572407
MIRT550308	hsa-miR-20a-5p	ZNF681	148213	PAR-CLIP	Functional MTI (Weak)	21572407
MIRT551127	hsa-miR-20a-5p	ZNF107	51427	PAR-CLIP	Functional MTI (Weak)	21572407
MIRT551748	hsa-miR-20a-5p	FMNL2	114793	PAR-CLIP	Functional MTI (Weak)	21572407
MIRT552208	hsa-miR-20a-5p	F2RL3	9002	PAR-CLIP	Functional MTI (Weak)	21572407
MIRT552689	hsa-miR-20a-5p	YWHAZ	7534	PAR-CLIP	Functional MTI (Weak)	21572407
MIRT552869	hsa-miR-20a-5p	WIPF2	147179	PAR-CLIP	Functional MTI (Weak)	21572407
MIRT552899	hsa-miR-20a-5p	WASL	8976	PAR-CLIP	Functional MTI (Weak)	21572407
MIRT553034	hsa-miR-20a-5p	USP48	84196	PAR-CLIP//HITS-CLIP	Functional MTI (Weak)	21572407
MIRT553579	hsa-miR-20a-5p	TMEM100	55273	PAR-CLIP	Functional MTI (Weak)	21572407
MIRT553705	hsa-miR-20a-5p	TCF7L2	6934	PAR-CLIP	Functional MTI (Weak)	21572407
MIRT554416	hsa-miR-20a-5p	SCD	6319	PAR-CLIP	Functional MTI (Weak)	21572407
MIRT554485	hsa-miR-20a-5p	SAMD12	401474	PAR-CLIP	Functional MTI (Weak)	21572407
MIRT554558	hsa-miR-20a-5p	RRN3	54700	PAR-CLIP	Functional MTI (Weak)	21572407
MIRT554751	hsa-miR-20a-5p	RHOC	389	PAR-CLIP	Functional MTI (Weak)	21572407
MIRT555468	hsa-miR-20a-5p	POLR3A	11128	PAR-CLIP	Functional MTI (Weak)	21572407
MIRT556188	hsa-miR-20a-5p	MCC	4163	PAR-CLIP	Functional MTI (Weak)	21572407
MIRT556596	hsa-miR-20a-5p	LEPROT	54741	PAR-CLIP	Functional MTI (Weak)	21572407
MIRT556908	hsa-miR-20a-5p	ISOC1	51015	PAR-CLIP	Functional MTI (Weak)	21572407
MIRT557037	hsa-miR-20a-5p	HOXD11	3237	PAR-CLIP	Functional MTI (Weak)	21572407
MIRT557596	hsa-miR-20a-5p	GNPTAB	79158	PAR-CLIP	Functional MTI (Weak)	21572407
MIRT557768	hsa-miR-20a-5p	FRS2	10818	PAR-CLIP	Functional MTI (Weak)	21572407
MIRT557892	hsa-miR-20a-5p	FEM1B	10116	PAR-CLIP	Functional MTI (Weak)	21572407
MIRT558341	hsa-miR-20a-5p	DNAJC28	54943	PAR-CLIP	Functional MTI (Weak)	21572407
MIRT558939	hsa-miR-20a-5p	CBX1	10951	PAR-CLIP	Functional MTI (Weak)	21572407
MIRT559442	hsa-miR-20a-5p	ARSJ	79642	PAR-CLIP	Functional MTI (Weak)	21572407
MIRT561247	hsa-miR-20a-5p	ZNF354B	117608	PAR-CLIP	Functional MTI (Weak)	20371350
MIRT561650	hsa-miR-20a-5p	RUNX3	864	PAR-CLIP	Functional MTI (Weak)	20371350

MIRT562219	hsa-miR-20a-5p	HMGB2	3148	PAR-CLIP	Functional MTI (Weak)	20371350
MIRT562567	hsa-miR-20a-5p	CCDC71L	168455	PAR-CLIP	Functional MTI (Weak)	20371350
MIRT562969	hsa-miR-20a-5p	LRPAP1	4043	PAR-CLIP	Functional MTI (Weak)	20371350
MIRT563383	hsa-miR-20a-5p	DSPP	1834	PAR-CLIP	Functional MTI (Weak)	20371350
MIRT564687	hsa-miR-20a-5p	ZNF35	7584	PAR-CLIP	Functional MTI (Weak)	20371350
MIRT565704	hsa-miR-20a-5p	SESN3	143686	PAR-CLIP	Functional MTI (Weak)	20371350
MIRT566145	hsa-miR-20a-5p	RACGAP1	29127	PAR-CLIP	Functional MTI (Weak)	20371350
MIRT566573	hsa-miR-20a-5p	OTUD4	54726	PAR-CLIP	Functional MTI (Weak)	20371350
MIRT566882	hsa-miR-20a-5p	LRP12	29967	PAR-CLIP	Functional MTI (Weak)	20371350
MIRT567059	hsa-miR-20a-5p	KCNB1	3745	PAR-CLIP	Functional MTI (Weak)	20371350
MIRT567117	hsa-miR-20a-5p	ITGB1	3688	PAR-CLIP	Functional MTI (Weak)	20371350
MIRT567547	hsa-miR-20a-5p	FGFR1OP	11116	PAR-CLIP	Functional MTI (Weak)	20371350
MIRT567689	hsa-miR-20a-5p	EIF4A2	1974	PAR-CLIP	Functional MTI (Weak)	20371350
MIRT567850	hsa-miR-20a-5p	DCAF8	50717	PAR-CLIP	Functional MTI (Weak)	20371350
MIRT567996	hsa-miR-20a-5p	COX6B1	1340	PAR-CLIP	Functional MTI (Weak)	20371350
MIRT568184	hsa-miR-20a-5p	CCDC6	8030	PAR-CLIP	Functional MTI (Weak)	20371350
MIRT568274	hsa-miR-20a-5p	BICD2	23299	PAR-CLIP	Functional MTI (Weak)	20371350
MIRT571019	hsa-miR-20a-5p	CKAP2	26586	PAR-CLIP	Functional MTI (Weak)	20371350
MIRT571682	hsa-miR-20a-5p	RRAS2	22800	PAR-CLIP	Functional MTI (Weak)	20371350
MIRT571698	hsa-miR-20a-5p	RPRD2	23248	PAR-CLIP	Functional MTI (Weak)	20371350
MIRT571728	hsa-miR-20a-5p	RPL17-C18orf32	100526842	PAR-CLIP	Functional MTI (Weak)	20371350
MIRT571863	hsa-miR-20a-5p	NKIRAS1	28512	PAR-CLIP	Functional MTI (Weak)	20371350
MIRT572213	hsa-miR-20a-5p	C18orf32	497661	PAR-CLIP	Functional MTI (Weak)	20371350
MIRT572680	hsa-miR-20a-5p	AGMAT	79814	PAR-CLIP	Functional MTI (Weak)	20371350
MIRT573920	hsa-miR-20a-5p	SNAP47	116841	PAR-CLIP	Functional MTI (Weak)	20371350
MIRT608372	hsa-miR-20a-5p	PIWIL2	55124	HITS-CLIP	Functional MTI (Weak)	24906430
MIRT608752	hsa-miR-20a-5p	MYH9	4627	HITS-CLIP	Functional MTI (Weak)	24906430
MIRT608978	hsa-miR-20a-5p	PRKCB	5579	HITS-CLIP	Functional MTI (Weak)	24906430
MIRT611003	hsa-miR-20a-5p	BRI3BP	140707	HITS-CLIP	Functional MTI (Weak)	23824327
MIRT611840	hsa-miR-20a-5p	FEM1A	55527	HITS-CLIP	Functional MTI (Weak)	23824327
MIRT612615	hsa-miR-20a-5p	RANGAP1	5905	HITS-CLIP	Functional MTI (Weak)	23824327
MIRT614267	hsa-miR-20a-5p	WDR53	348793	HITS-CLIP	Functional MTI (Weak)	23824327
MIRT615180	hsa-miR-20a-5p	SPIB	6689	HITS-CLIP	Functional MTI (Weak)	23824327
MIRT615451	hsa-miR-20a-5p	FAXC	84553	HITS-CLIP	Functional MTI (Weak)	23824327
MIRT616176	hsa-miR-20a-5p	ELOC	6921	HITS-CLIP	Functional MTI (Weak)	23824327
MIRT619845	hsa-miR-20a-5p	POLM	27434	HITS-CLIP	Functional MTI (Weak)	23824327
MIRT620981	hsa-miR-20a-5p	TM4SF5	9032	HITS-CLIP	Functional MTI (Weak)	23824327
MIRT624428	hsa-miR-20a-5p	CBX8	57332	HITS-CLIP	Functional MTI (Weak)	23824327
MIRT625084	hsa-miR-20a-5p	C15orf41	84529	HITS-CLIP	Functional MTI (Weak)	23824327
MIRT626042	hsa-miR-20a-5p	ATAT1	79969	HITS-CLIP	Functional MTI (Weak)	23824327
MIRT626225	hsa-miR-20a-5p	PNRC1	10957	HITS-CLIP	Functional MTI (Weak)	23824327
MIRT626934	hsa-miR-20a-5p	HIST1H2BG	8339	HITS-CLIP	Functional MTI (Weak)	23824327
MIRT628569	hsa-miR-20a-5p	MELK	9833	HITS-CLIP	Functional MTI (Weak)	23824327
MIRT633126	hsa-miR-20a-5p	CBX5	23468	HITS-CLIP	Functional MTI (Weak)	23824327
MIRT634102	hsa-miR-20a-5p	APOH	350	HITS-CLIP	Functional MTI (Weak)	23824327
MIRT634459	hsa-miR-20a-5p	PAK6	56924	HITS-CLIP	Functional MTI (Weak)	23824327
MIRT634650	hsa-miR-20a-5p	HIP1	3092	HITS-CLIP	Functional MTI (Weak)	23824327
MIRT634959	hsa-miR-20a-5p	GTF2H2C	728340	HITS-CLIP	Functional MTI (Weak)	23824327
MIRT640014	hsa-miR-20a-5p	OSTM1	28962	HITS-CLIP	Functional MTI (Weak)	23824327
MIRT641710	hsa-miR-20a-5p	SPCS1	28972	HITS-CLIP	Functional MTI (Weak)	23824327
MIRT645219	hsa-miR-20a-5p	POLR3F	10621	HITS-CLIP	Functional MTI (Weak)	23824327
MIRT662411	hsa-miR-20a-5p	ICA1L	130026	HITS-CLIP	Functional MTI (Weak)	23824327
MIRT664232	hsa-miR-20a-5p	LSM3	27258	HITS-CLIP	Functional MTI (Weak)	23824327
MIRT664398	hsa-miR-20a-5p	CYB5A	1528	HITS-CLIP	Functional MTI (Weak)	23824327
MIRT664798	hsa-miR-20a-5p	LIAS	11019	HITS-CLIP	Functional MTI (Weak)	23824327
MIRT673138	hsa-miR-20a-5p	MFSD2A	84879	HITS-CLIP	Functional MTI (Weak)	23824327
MIRT675843	hsa-miR-20a-5p	DHODH	1723	HITS-CLIP	Functional MTI (Weak)	23824327
MIRT677159	hsa-miR-20a-5p	DEGS1	8560	HITS-CLIP	Functional MTI (Weak)	23824327
MIRT677265	hsa-miR-20a-5p	C15orf40	123207	HITS-CLIP	Functional MTI (Weak)	23824327
MIRT678752	hsa-miR-20a-5p	SRCAP	10847	HITS-CLIP	Functional MTI (Weak)	23824327
MIRT680378	hsa-miR-20a-5p	GATAD1	57798	HITS-CLIP	Functional MTI (Weak)	23824327
MIRT680705	hsa-miR-20a-5p	ZNF785	146540	HITS-CLIP	Functional MTI (Weak)	23706177
MIRT680783	hsa-miR-20a-5p	WDR73	84942	HITS-CLIP	Functional MTI (Weak)	23706177
MIRT681415	hsa-miR-20a-5p	RMND1	55005	HITS-CLIP	Functional MTI (Weak)	23706177
MIRT681709	hsa-miR-20a-5p	ABI2	10152	HITS-CLIP	Functional MTI (Weak)	23706177
MIRT681845	hsa-miR-20a-5p	N4BP2L2	10443	HITS-CLIP	Functional MTI (Weak)	23706177
MIRT682336	hsa-miR-20a-5p	RAB42	115273	HITS-CLIP	Functional MTI (Weak)	23706177
MIRT683334	hsa-miR-20a-5p	FAAP24	91442	HITS-CLIP	Functional MTI (Weak)	23313552
MIRT683404	hsa-miR-20a-5p	ESR2	2100	HITS-CLIP	Functional MTI (Weak)	23313552
MIRT683508	hsa-miR-20a-5p	ZNF7	7553	HITS-CLIP	Functional MTI (Weak)	23313552
MIRT683540	hsa-miR-20a-5p	C11orf54	28970	HITS-CLIP	Functional MTI (Weak)	23313552
MIRT683891	hsa-miR-20a-5p	OCIAD1	54940	HITS-CLIP	Functional MTI (Weak)	23313552
MIRT683964	hsa-miR-20a-5p	MYLK3	91807	HITS-CLIP	Functional MTI (Weak)	23313552
MIRT683993	hsa-miR-20a-5p	QRFPR	84109	HITS-CLIP	Functional MTI (Weak)	23313552
MIRT684096	hsa-miR-20a-5p	TLR7	51284	HITS-CLIP	Functional MTI (Weak)	23313552
MIRT684149	hsa-miR-20a-5p	CEP104	9731	HITS-CLIP	Functional MTI (Weak)	23313552
MIRT684374	hsa-miR-20a-5p	BCAS4	55653	HITS-CLIP	Functional MTI (Weak)	23313552
MIRT684590	hsa-miR-20a-5p	ORAI2	80228	HITS-CLIP	Functional MTI (Weak)	23313552
MIRT684633	hsa-miR-20a-5p	GTF2IRD2B	389524	HITS-CLIP	Functional MTI (Weak)	23313552
MIRT684664	hsa-miR-20a-5p	PDE4C	5143	HITS-CLIP	Functional MTI (Weak)	23313552
MIRT684729	hsa-miR-20a-5p	LRRD1	401387	HITS-CLIP	Functional MTI (Weak)	23313552
MIRT684758	hsa-miR-20a-5p	DNAJB13	374407	HITS-CLIP	Functional MTI (Weak)	23313552
MIRT684803	hsa-miR-20a-5p	MYO1F	4542	HITS-CLIP	Functional MTI (Weak)	23313552
MIRT684935	hsa-miR-20a-5p	CD28	940	HITS-CLIP	Functional MTI (Weak)	23313552
MIRT685211	hsa-miR-20a-5p	DCTN5	84516	HITS-CLIP	Functional MTI (Weak)	23313552
MIRT685264	hsa-miR-20a-5p	F2RL1	2150	HITS-CLIP	Functional MTI (Weak)	23313552
MIRT685332	hsa-miR-20a-5p	ASB16	92591	HITS-CLIP	Functional MTI (Weak)	23313552
MIRT685367	hsa-miR-20a-5p	CCL5	6352	HITS-CLIP	Functional MTI (Weak)	23313552
MIRT685535	hsa-miR-20a-5p	MSH3	4437	HITS-CLIP	Functional MTI (Weak)	23313552
MIRT685594	hsa-miR-20a-5p	KCNK6	9424	HITS-CLIP	Functional MTI (Weak)	23313552
MIRT685725	hsa-miR-20a-5p	BHMT2	23743	HITS-CLIP	Functional MTI (Weak)	23313552
MIRT685758	hsa-miR-20a-5p	C12orf65	91574	HITS-CLIP	Functional MTI (Weak)	23313552
MIRT685799	hsa-miR-20a-5p	ZNF426	79088	HITS-CLIP	Functional MTI (Weak)	23313552
MIRT685971	hsa-miR-20a-5p	PTGIS	5740	HITS-CLIP	Functional MTI (Weak)	23313552
MIRT686122	hsa-miR-20a-5p	TNIP3	79931	HITS-CLIP	Functional MTI (Weak)	23313552
MIRT686172	hsa-miR-20a-5p	HS3ST1	9957	HITS-CLIP	Functional MTI (Weak)	23313552
MIRT686299	hsa-miR-20a-5p	WWC1	23286	HITS-CLIP	Functional MTI (Weak)	23313552
MIRT686337	hsa-miR-20a-5p	VPS53	55275	HITS-CLIP	Functional MTI (Weak)	23313552
MIRT686460	hsa-miR-20a-5p	LINC00598	646982	HITS-CLIP	Functional MTI (Weak)	23313552
MIRT686504	hsa-miR-20a-5p	TRIOBP	11078	HITS-CLIP	Functional MTI (Weak)	23313552
MIRT686543	hsa-miR-20a-5p	TRAF3IP2	10758	HITS-CLIP	Functional MTI (Weak)	23313552
MIRT686598	hsa-miR-20a-5p	TMOD3	29766	HITS-CLIP	Functional MTI (Weak)	23313552
MIRT686843	hsa-miR-20a-5p	SLC7A11	23657	HITS-CLIP	Functional MTI (Weak)	23313552
MIRT686896	hsa-miR-20a-5p	SLC1A5	6510	HITS-CLIP	Functional MTI (Weak)	23313552
MIRT686996	hsa-miR-20a-5p	SERINC1	57515	HITS-CLIP	Functional MTI (Weak)	23313552

MIRT687060	hsa-miR-20a-5p	RNF115	27246	HITS-CLIP	Functional MTI (Weak)	23313552
MIRT687095	hsa-miR-20a-5p	RABGAP1L	9910	HITS-CLIP	Functional MTI (Weak)	23313552
MIRT687270	hsa-miR-20a-5p	PDHB	5162	HITS-CLIP	Functional MTI (Weak)	23313552
MIRT687666	hsa-miR-20a-5p	LRIF1	55791	HITS-CLIP	Functional MTI (Weak)	23313552
MIRT687874	hsa-miR-20a-5p	ISCA2	122961	HITS-CLIP	Functional MTI (Weak)	23313552
MIRT687999	hsa-miR-20a-5p	GTF2IRD2	84163	HITS-CLIP	Functional MTI (Weak)	23313552
MIRT688139	hsa-miR-20a-5p	GEMIN8	54960	HITS-CLIP	Functional MTI (Weak)	23313552
MIRT688233	hsa-miR-20a-5p	FKBP14	55033	HITS-CLIP	Functional MTI (Weak)	23313552
MIRT688291	hsa-miR-20a-5p	FAM213A	84293	HITS-CLIP	Functional MTI (Weak)	23313552
MIRT688473	hsa-miR-20a-5p	DNAJB4	11080	HITS-CLIP	Functional MTI (Weak)	23313552
MIRT688522	hsa-miR-20a-5p	DDI2	84301	HITS-CLIP	Functional MTI (Weak)	23313552
MIRT688682	hsa-miR-20a-5p	CPT1A	1374	HITS-CLIP	Functional MTI (Weak)	23313552
MIRT688716	hsa-miR-20a-5p	CPS1	1373	HITS-CLIP	Functional MTI (Weak)	23313552
MIRT688846	hsa-miR-20a-5p	CAPZA2	830	HITS-CLIP	Functional MTI (Weak)	23313552
MIRT689128	hsa-miR-20a-5p	ZBTB25	7597	HITS-CLIP	Functional MTI (Weak)	23313552
MIRT689190	hsa-miR-20a-5p	ZNF665	79788	HITS-CLIP	Functional MTI (Weak)	23313552
MIRT689815	hsa-miR-20a-5p	GTF2H3	2967	HITS-CLIP	Functional MTI (Weak)	23313552
MIRT689860	hsa-miR-20a-5p	HIST1H2BJ	8970	HITS-CLIP	Functional MTI (Weak)	23313552
MIRT690755	hsa-miR-20a-5p	IRAK4	51135	HITS-CLIP	Functional MTI (Weak)	23313552
MIRT691000	hsa-miR-20a-5p	ZNF578	147660	HITS-CLIP	Functional MTI (Weak)	23313552
MIRT691092	hsa-miR-20a-5p	NUGGC	389643	HITS-CLIP	Functional MTI (Weak)	23313552
MIRT691349	hsa-miR-20a-5p	KIAA1841	84542	HITS-CLIP	Functional MTI (Weak)	23313552
MIRT691512	hsa-miR-20a-5p	FOXRED2	80020	HITS-CLIP	Functional MTI (Weak)	23313552
MIRT691594	hsa-miR-20a-5p	CCDC125	202243	HITS-CLIP	Functional MTI (Weak)	23313552
MIRT691630	hsa-miR-20a-5p	IPP	3652	HITS-CLIP	Functional MTI (Weak)	23313552
MIRT692090	hsa-miR-20a-5p	ACOT9	23597	HITS-CLIP	Functional MTI (Weak)	23313552
MIRT692127	hsa-miR-20a-5p	CXorf38	159013	HITS-CLIP	Functional MTI (Weak)	23313552
MIRT692336	hsa-miR-20a-5p	RFK	55312	HITS-CLIP	Functional MTI (Weak)	23313552
MIRT692398	hsa-miR-20a-5p	LY6G5B	58496	HITS-CLIP	Functional MTI (Weak)	23313552
MIRT692459	hsa-miR-20a-5p	METTL8	79828	HITS-CLIP	Functional MTI (Weak)	23313552
MIRT692562	hsa-miR-20a-5p	PARD3	56288	HITS-CLIP	Functional MTI (Weak)	23313552
MIRT692623	hsa-miR-20a-5p	GDF5OS	554250	HITS-CLIP	Functional MTI (Weak)	23313552
MIRT692804	hsa-miR-20a-5p	SYNPO2L	79933	HITS-CLIP	Functional MTI (Weak)	23313552
MIRT692835	hsa-miR-20a-5p	C1orf50	79078	HITS-CLIP	Functional MTI (Weak)	23313552
MIRT692897	hsa-miR-20a-5p	RBM41	55285	HITS-CLIP	Functional MTI (Weak)	23313552
MIRT693007	hsa-miR-20a-5p	LGSN	51557	HITS-CLIP	Functional MTI (Weak)	23313552
MIRT693157	hsa-miR-20a-5p	THEM4	117145	HITS-CLIP	Functional MTI (Weak)	23313552
MIRT693368	hsa-miR-20a-5p	RNF34	80196	HITS-CLIP	Functional MTI (Weak)	23313552
MIRT694129	hsa-miR-20a-5p	ZNF446	55663	HITS-CLIP	Functional MTI (Weak)	23313552
MIRT694213	hsa-miR-20a-5p	ZNF347	84671	HITS-CLIP	Functional MTI (Weak)	23313552
MIRT694679	hsa-miR-20a-5p	C14orf119	55017	HITS-CLIP	Functional MTI (Weak)	23313552
MIRT694833	hsa-miR-20a-5p	STX4	6810	HITS-CLIP	Functional MTI (Weak)	23313552
MIRT694955	hsa-miR-20a-5p	ANKS4B	257629	HITS-CLIP	Functional MTI (Weak)	23313552
MIRT695199	hsa-miR-20a-5p	SLC25A33	84275	HITS-CLIP	Functional MTI (Weak)	23313552
MIRT695681	hsa-miR-20a-5p	MAN2B2	23324	HITS-CLIP	Functional MTI (Weak)	23313552
MIRT695852	hsa-miR-20a-5p	ABCG8	64241	HITS-CLIP	Functional MTI (Weak)	23313552
MIRT695944	hsa-miR-20a-5p	ZNF174	7727	HITS-CLIP	Functional MTI (Weak)	23313552
MIRT696202	hsa-miR-20a-5p	GNB5	10681	HITS-CLIP	Functional MTI (Weak)	23313552
MIRT696463	hsa-miR-20a-5p	SUGP1	57794	HITS-CLIP	Functional MTI (Weak)	23313552
MIRT696879	hsa-miR-20a-5p	UBOX5	22888	HITS-CLIP	Functional MTI (Weak)	23313552
MIRT696926	hsa-miR-20a-5p	CCDC198	55195	HITS-CLIP	Functional MTI (Weak)	23313552
MIRT697265	hsa-miR-20a-5p	ZYG11A	440590	HITS-CLIP	Functional MTI (Weak)	23313552
MIRT697410	hsa-miR-20a-5p	ZMAT3	64393	HITS-CLIP	Functional MTI (Weak)	23313552
MIRT698004	hsa-miR-20a-5p	TSPAN6	7105	HITS-CLIP	Functional MTI (Weak)	23313552
MIRT699290	hsa-miR-20a-5p	SLC6A4	6532	HITS-CLIP	Functional MTI (Weak)	23313552
MIRT699653	hsa-miR-20a-5p	SH3BP5	9467	HITS-CLIP	Functional MTI (Weak)	23313552
MIRT699715	hsa-miR-20a-5p	SF3B3	23450	HITS-CLIP	Functional MTI (Weak)	23313552
MIRT700064	hsa-miR-20a-5p	RPL14	9045	HITS-CLIP	Functional MTI (Weak)	23313552
MIRT700122	hsa-miR-20a-5p	RNF19B	127544	HITS-CLIP	Functional MTI (Weak)	23313552
MIRT701130	hsa-miR-20a-5p	PAPD5	64282	HITS-CLIP	Functional MTI (Weak)	23313552
MIRT701314	hsa-miR-20a-5p	NUDT3	11165	HITS-CLIP	Functional MTI (Weak)	23313552
MIRT701596	hsa-miR-20a-5p	MYPN	84665	HITS-CLIP	Functional MTI (Weak)	23313552
MIRT702396	hsa-miR-20a-5p	KLF10	7071	HITS-CLIP	Functional MTI (Weak)	23313552
MIRT702545	hsa-miR-20a-5p	KCND3	3752	HITS-CLIP	Functional MTI (Weak)	23313552
MIRT703111	hsa-miR-20a-5p	GPRIN3	285513	HITS-CLIP	Functional MTI (Weak)	23313552
MIRT704130	hsa-miR-20a-5p	DRAXIN	374946	HITS-CLIP	Functional MTI (Weak)	23313552
MIRT704161	hsa-miR-20a-5p	DNAL1	83544	HITS-CLIP	Functional MTI (Weak)	23313552
MIRT704217	hsa-miR-20a-5p	LDHD	197257	HITS-CLIP	Functional MTI (Weak)	23313552
MIRT704783	hsa-miR-20a-5p	CDKN2AIPNL	91368	HITS-CLIP	Functional MTI (Weak)	23313552
MIRT705104	hsa-miR-20a-5p	ABHD18	80167	HITS-CLIP	Functional MTI (Weak)	23313552
MIRT705369	hsa-miR-20a-5p	ATP1B3	483	HITS-CLIP	Functional MTI (Weak)	23313552
MIRT706125	hsa-miR-20a-5p	ENTPD4	9583	HITS-CLIP	Functional MTI (Weak)	22927820
MIRT706295	hsa-miR-20a-5p	SLC35F6	54978	HITS-CLIP	Functional MTI (Weak)	22927820
MIRT706331	hsa-miR-20a-5p	CCDC30	728621	HITS-CLIP	Functional MTI (Weak)	22927820
MIRT706371	hsa-miR-20a-5p	STAC2	342667	HITS-CLIP	Functional MTI (Weak)	22927820
MIRT706423	hsa-miR-20a-5p	HAS2	3037	HITS-CLIP	Functional MTI (Weak)	22927820
MIRT706534	hsa-miR-20a-5p	MTMR9	66036	HITS-CLIP	Functional MTI (Weak)	22927820
MIRT707608	hsa-miR-20a-5p	PCNX2	80003	HITS-CLIP	Functional MTI (Weak)	21572407
MIRT707836	hsa-miR-20a-5p	TMEM133	83935	HITS-CLIP	Functional MTI (Weak)	21572407
MIRT708390	hsa-miR-20a-5p	CDIPT	10423	HITS-CLIP	Functional MTI (Weak)	19536157
MIRT708469	hsa-miR-20a-5p	MAPKAPK5	8550	HITS-CLIP	Functional MTI (Weak)	19536157
MIRT709092	hsa-miR-20a-5p	FAHD1	81889	HITS-CLIP	Functional MTI (Weak)	19536157
MIRT709557	hsa-miR-20a-5p	ZBED1	9189	HITS-CLIP	Functional MTI (Weak)	19536157
MIRT710425	hsa-miR-20a-5p	YTHDC1	91746	HITS-CLIP	Functional MTI (Weak)	19536157
MIRT711789	hsa-miR-20a-5p	RFXAP	5994	HITS-CLIP	Functional MTI (Weak)	19536157
MIRT713354	hsa-miR-20a-5p	KLRD1	3824	HITS-CLIP	Functional MTI (Weak)	19536157
MIRT714326	hsa-miR-20a-5p	ZNF454	285676	HITS-CLIP	Functional MTI (Weak)	19536157
MIRT716560	hsa-miR-20a-5p	GOLGA2	2801	HITS-CLIP	Functional MTI (Weak)	19536157
MIRT719091	hsa-miR-20a-5p	ACOX1	51	HITS-CLIP	Functional MTI (Weak)	19536157
MIRT725227	hsa-miR-20a-5p	PEA15	8682	HITS-CLIP	Functional MTI (Weak)	19536157
MIRT731268	hsa-miR-20a-5p	RB1CC1	9821	Luciferase reporter assay	Functional MTI	26829385
MIRT731837	hsa-miR-20a-5p	NFKBIB	4793	Immunohistochemistry//Luciferase reporter assay//qRT-PCR//Western blot	Functional MTI	26286834
MIRT732249	hsa-miR-20a-5p	KIF26B	55083	//Luciferase reporter assay//qRT-PCR//Western blot	Functional MTI	27499703
MIRT732435	hsa-miR-20a-5p	TIMP2	7077	Luciferase reporter assay	Functional MTI	24704830
MIRT732531	hsa-miR-20a-5p	PTENP1	11191	qRT-PCR	Functional MTI (Weak)	25617127
MIRT732671	hsa-miR-20a-5p	NTN4	59277	In situ hybridization//Next Generation Sequencing (NGS)//qRT-PCR	Functional MTI (Weak)	26233958
MIRT734034	hsa-miR-20a-5p	TGFBR1	7046	Immunoblot//Immunofluorescence//Luciferase reporter assay//qRT-PCR	Functional MTI	26729221
MIRT734853	hsa-miR-20a-5p	PTPRO	5800	Luciferase reporter assay	Functional MTI	27720936
MIRT734854	hsa-miR-20a-5p	PPP2R2A	5520	Luciferase reporter assay	Functional MTI	27720936
MIRT735333	hsa-miR-20a-5p	DAPK3	1613	GFP reporter assay//qRT-PCR//Western blot	Functional MTI	26117336
MIRT738870	hsa-miR-20a-5p	DCBLD2	131566	PAR-CLIP	Functional MTI (Weak)	26701625
MIRT738871	hsa-miR-20a-5p	EREG	2069	PAR-CLIP	Functional MTI (Weak)	26701625
MIRT738872	hsa-miR-20a-5p	GPR183	1880	PAR-CLIP	Functional MTI (Weak)	26701625
MIRT738873	hsa-miR-20a-5p	MINOS1-NBL1	100532736	PAR-CLIP	Functional MTI (Weak)	26701625
MIRT738874	hsa-miR-20a-5p	NBL1	4681	PAR-CLIP	Functional MTI (Weak)	26701625

MIRT738875	hsa-miR-20a-5p	PLEKHM1	9842	PAR-CLIP	Functional MTI (Weak)	26701625
MIRT738876	hsa-miR-20a-5p	RAB3IP	117177	PAR-CLIP	Functional MTI (Weak)	26701625
MIRT738877	hsa-miR-20a-5p	SYNJ2BP	55333	PAR-CLIP	Functional MTI (Weak)	26701625
MIRT763757	hsa-miR-20a-5p	DPP9	91039	PAR-CLIP	Functional MTI (Weak)	27292025
MIRT763758	hsa-miR-20a-5p	DSTYK	25778	PAR-CLIP	Functional MTI (Weak)	27292025
MIRT763759	hsa-miR-20a-5p	ISY1	57461	PAR-CLIP	Functional MTI (Weak)	27292025
MIRT763760	hsa-miR-20a-5p	KPNA6	23633	PAR-CLIP	Functional MTI (Weak)	27292025
MIRT763761	hsa-miR-20a-5p	NR3C1	2908	PAR-CLIP	Functional MTI (Weak)	27292025
MIRT763762	hsa-miR-20a-5p	PMAIP1	5366	PAR-CLIP	Functional MTI (Weak)	27292025
MIRT763763	hsa-miR-20a-5p	SESN1	27244	PAR-CLIP	Functional MTI (Weak)	27292025
MIRT763764	hsa-miR-20a-5p	SLC12A6	9990	PAR-CLIP	Functional MTI (Weak)	27292025
MIRT763765	hsa-miR-20a-5p	TRIM65	201292	PAR-CLIP	Functional MTI (Weak)	27292025
MIRT784171	hsa-miR-20a-5p	ARMT1	79624	HITS-CLIP	Functional MTI (Weak)	27418678
MIRT784172	hsa-miR-20a-5p	C17orf75	64149	HITS-CLIP	Functional MTI (Weak)	27418678
MIRT784173	hsa-miR-20a-5p	FAM241A	132720	HITS-CLIP	Functional MTI (Weak)	27418678
MIRT784174	hsa-miR-20a-5p	LRRC58	116064	HITS-CLIP	Functional MTI (Weak)	27418678
MIRT784175	hsa-miR-20a-5p	MPPE1	65258	HITS-CLIP	Functional MTI (Weak)	27418678
MIRT784176	hsa-miR-20a-5p	MRPS10	55173	HITS-CLIP	Functional MTI (Weak)	27418678
MIRT784177	hsa-miR-20a-5p	OLAH	55301	HITS-CLIP	Functional MTI (Weak)	27418678
MIRT784178	hsa-miR-20a-5p	SON	6651	HITS-CLIP	Functional MTI (Weak)	27418678
MIRT784179	hsa-miR-20a-5p	SP2	6668	HITS-CLIP	Functional MTI (Weak)	27418678
MIRT784180	hsa-miR-20a-5p	WDR92	116143	HITS-CLIP	Functional MTI (Weak)	27418678
MIRT784181	hsa-miR-20a-5p	ZNF786	136051	HITS-CLIP	Functional MTI (Weak)	27418678
MIRT061357	hsa-miR-194-3p	WEE1	7465	PAR-CLIP	Functional MTI (Weak)	23446348
MIRT144690	hsa-miR-194-3p	SLC7A5	8140	PAR-CLIP	Functional MTI (Weak)	23592263
MIRT180544	hsa-miR-194-3p	TXNIP	10628	PAR-CLIP	Functional MTI (Weak)	23446348
MIRT185104	hsa-miR-194-3p	LYPLAL1	127018	PAR-CLIP	Functional MTI (Weak)	23592263
MIRT221017	hsa-miR-194-3p	UBN2	254048	HITS-CLIP	Functional MTI (Weak)	24906430
MIRT336220	hsa-miR-194-3p	SKI	6497	PAR-CLIP	Functional MTI (Weak)	20371350
MIRT356733	hsa-miR-194-3p	C5orf30	90355	PAR-CLIP	Functional MTI (Weak)	22100165
MIRT361010	hsa-miR-194-3p	CDC5L	988	PAR-CLIP	Functional MTI (Weak)	23592263
MIRT378601	hsa-miR-194-3p	KCNQ5	56479	HITS-CLIP	Functional MTI (Weak)	24906430
MIRT442819	hsa-miR-194-3p	CDH6	1004	PAR-CLIP	Functional MTI (Weak)	22100165
MIRT450939	hsa-miR-194-3p	BDP1	55814	PAR-CLIP	Functional MTI (Weak)	22100165
MIRT453122	hsa-miR-194-3p	HOXC4	3221	PAR-CLIP	Functional MTI (Weak)	23592263
MIRT453144	hsa-miR-194-3p	PLA2G2C	391013	PAR-CLIP	Functional MTI (Weak)	23592263
MIRT453556	hsa-miR-194-3p	PRR12	57479	PAR-CLIP	Functional MTI (Weak)	23592263
MIRT453645	hsa-miR-194-3p	SLC4A2	6522	PAR-CLIP	Functional MTI (Weak)	23592263
MIRT453728	hsa-miR-194-3p	RAP1GDS1	5910	PAR-CLIP	Functional MTI (Weak)	23592263
MIRT453746	hsa-miR-194-3p	CSNK1E	1454	PAR-CLIP	Functional MTI (Weak)	23592263
MIRT453811	hsa-miR-194-3p	KBTBD12	166348	PAR-CLIP	Functional MTI (Weak)	23592263
MIRT454397	hsa-miR-194-3p	NRG4	145957	PAR-CLIP	Functional MTI (Weak)	23592263
MIRT454450	hsa-miR-194-3p	QRFPR	84109	PAR-CLIP	Functional MTI (Weak)	23592263
MIRT455169	hsa-miR-194-3p	SUV39H1	6839	PAR-CLIP	Functional MTI (Weak)	23592263
MIRT455791	hsa-miR-194-3p	TAF8	129685	PAR-CLIP	Functional MTI (Weak)	23592263
MIRT457702	hsa-miR-194-3p	ZNF587	84914	PAR-CLIP	Functional MTI (Weak)	23592263
MIRT457893	hsa-miR-194-3p	THEM6	51337	PAR-CLIP	Functional MTI (Weak)	23592263
MIRT458690	hsa-miR-194-3p	MRI1	84245	PAR-CLIP	Functional MTI (Weak)	23592263
MIRT458897	hsa-miR-194-3p	PFAS	5198	PAR-CLIP	Functional MTI (Weak)	23592263
MIRT461741	hsa-miR-194-3p	NDUFA2	4695	PAR-CLIP	Functional MTI (Weak)	23592263
MIRT461896	hsa-miR-194-3p	NECAB3	63941	PAR-CLIP	Functional MTI (Weak)	23592263
MIRT463513	hsa-miR-194-3p	ZBTB8B	728116	PAR-CLIP	Functional MTI (Weak)	23592263
MIRT463723	hsa-miR-194-3p	YWHAE	7531	PAR-CLIP	Functional MTI (Weak)	23592263
MIRT464202	hsa-miR-194-3p	VGLL4	9686	PAR-CLIP	Functional MTI (Weak)	23592263
MIRT464331	hsa-miR-194-3p	UST	10090	PAR-CLIP	Functional MTI (Weak)	23592263
MIRT465076	hsa-miR-194-3p	TSR1	55720	PAR-CLIP	Functional MTI (Weak)	23592263
MIRT465446	hsa-miR-194-3p	TP53	7157	PAR-CLIP	Functional MTI (Weak)	23592263
MIRT466583	hsa-miR-194-3p	TBC1D2B	23102	PAR-CLIP	Functional MTI (Weak)	23592263
MIRT467140	hsa-miR-194-3p	SREK1IP1	285672	PAR-CLIP	Functional MTI (Weak)	23592263
MIRT467684	hsa-miR-194-3p	SLC38A2	54407	PAR-CLIP	Functional MTI (Weak)	23592263
MIRT468107	hsa-miR-194-3p	SH3TC2	79628	PAR-CLIP	Functional MTI (Weak)	23592263
MIRT468323	hsa-miR-194-3p	SF3B3	23450	PAR-CLIP	Functional MTI (Weak)	23592263
MIRT468621	hsa-miR-194-3p	SUMO1	7341	PAR-CLIP	Functional MTI (Weak)	23592263
MIRT469332	hsa-miR-194-3p	RGP1	9827	PAR-CLIP	Functional MTI (Weak)	23592263
MIRT469574	hsa-miR-194-3p	RARA	5914	PAR-CLIP	Functional MTI (Weak)	23592263
MIRT469797	hsa-miR-194-3p	RAB15	376267	PAR-CLIP	Functional MTI (Weak)	23592263
MIRT470444	hsa-miR-194-3p	PPP1R15B	84919	PAR-CLIP	Functional MTI (Weak)	23592263
MIRT470693	hsa-miR-194-3p	POLR2D	5433	PAR-CLIP	Functional MTI (Weak)	23592263
MIRT472614	hsa-miR-194-3p	NAA50	80218	PAR-CLIP	Functional MTI (Weak)	23592263
MIRT474393	hsa-miR-194-3p	KLK10	5655	PAR-CLIP	Functional MTI (Weak)	23592263
MIRT475364	hsa-miR-194-3p	ICOSLG	23308	PAR-CLIP	Functional MTI (Weak)	23592263
MIRT477889	hsa-miR-194-3p	DYNLL2	140735	PAR-CLIP	Functional MTI (Weak)	23592263
MIRT478304	hsa-miR-194-3p	DDX19A	55308	PAR-CLIP	Functional MTI (Weak)	23592263
MIRT478360	hsa-miR-194-3p	DDI2	84301	PAR-CLIP	Functional MTI (Weak)	23592263
MIRT479609	hsa-miR-194-3p	CDC25A	993	PAR-CLIP	Functional MTI (Weak)	23592263
MIRT480634	hsa-miR-194-3p	BTBD3	22903	PAR-CLIP	Functional MTI (Weak)	23592263
MIRT480763	hsa-miR-194-3p	BMP3	651	PAR-CLIP	Functional MTI (Weak)	23592263
MIRT480868	hsa-miR-194-3p	BHLHB9	80823	PAR-CLIP	Functional MTI (Weak)	23592263
MIRT481325	hsa-miR-194-3p	ATP5A1	498	PAR-CLIP	Functional MTI (Weak)	23592263
MIRT481330	hsa-miR-194-3p	ATL3	25923	PAR-CLIP	Functional MTI (Weak)	23592263
MIRT481840	hsa-miR-194-3p	AP1S1	1174	PAR-CLIP	Functional MTI (Weak)	23592263
MIRT484003	hsa-miR-194-3p	ATAD5	79915	PAR-CLIP	Functional MTI (Weak)	23592263
MIRT485075	hsa-miR-194-3p	SPATA13	221178	PAR-CLIP	Functional MTI (Weak)	23592263
MIRT487291	hsa-miR-194-3p	GPAT4	137964	PAR-CLIP	Functional MTI (Weak)	23592263
MIRT487653	hsa-miR-194-3p	METTL22	79091	PAR-CLIP	Functional MTI (Weak)	23592263
MIRT490705	hsa-miR-194-3p	FSTL4	23105	PAR-CLIP	Functional MTI (Weak)	23592263
MIRT492290	hsa-miR-194-3p	SHISA6	388336	PAR-CLIP	Functional MTI (Weak)	23592263
MIRT493159	hsa-miR-194-3p	MKNK2	2872	PAR-CLIP	Functional MTI (Weak)	23592263
MIRT495715	hsa-miR-194-3p	PADI1	29943	PAR-CLIP	Functional MTI (Weak)	22291592
MIRT502209	hsa-miR-194-3p	HSPB8	26353	PAR-CLIP	Functional MTI (Weak)	24398324
MIRT502502	hsa-miR-194-3p	EVI5	7813	PAR-CLIP	Functional MTI (Weak)	24398324
MIRT513113	hsa-miR-194-3p	IP6K1	9807	PAR-CLIP	Functional MTI (Weak)	23446348
MIRT513614	hsa-miR-194-3p	VPS37B	79720	PAR-CLIP	Functional MTI (Weak)	23446348
MIRT514362	hsa-miR-194-3p	UBBP4	23666	PAR-CLIP	Functional MTI (Weak)	23446348
MIRT528132	hsa-miR-194-3p	PPP1R10	5514	PAR-CLIP	Functional MTI (Weak)	22012620
MIRT530501	hsa-miR-194-3p	FADS6	283985	PAR-CLIP	Functional MTI (Weak)	22012620
MIRT533066	hsa-miR-194-3p	ZBTB34	403341	PAR-CLIP	Functional MTI (Weak)	22012620
MIRT538400	hsa-miR-194-3p	CPT1A	1374	PAR-CLIP	Functional MTI (Weak)	22012620
MIRT540488	hsa-miR-194-3p	BLOC1S4	55330	PAR-CLIP	Functional MTI (Weak)	21572407
MIRT540981	hsa-miR-194-3p	NCBP3	55421	PAR-CLIP	Functional MTI (Weak)	21572407
MIRT559903	hsa-miR-194-3p	CNNM4	26504	PAR-CLIP	Functional MTI (Weak)	20371350
MIRT561819	hsa-miR-194-3p	NUFIP2	57532	PAR-CLIP	Functional MTI (Weak)	20371350
MIRT562375	hsa-miR-194-3p	ERI2	112479	PAR-CLIP	Functional MTI (Weak)	20371350
MIRT567681	hsa-miR-194-3p	EIF4EBP1	1978	PAR-CLIP	Functional MTI (Weak)	20371350
MIRT567741	hsa-miR-194-3p	DLX2	1746	PAR-CLIP	Functional MTI (Weak)	20371350

MIRT568810	hsa-miR-194-3p	VPS37D	155382	PAR-CLIP	Functional MTI (Weak)	20371350
MIRT571055	hsa-miR-194-3p	POLQ	10721	PAR-CLIP	Functional MTI (Weak)	20371350
MIRT572696	hsa-miR-194-3p	NCMAP	400746	PAR-CLIP	Functional MTI (Weak)	20371350
MIRT624884	hsa-miR-194-3p	AASDHPPT	60496	HITS-CLIP	Functional MTI (Weak)	23824327
MIRT642127	hsa-miR-194-3p	DDA1	79016	HITS-CLIP	Functional MTI (Weak)	23824327
MIRT644192	hsa-miR-194-3p	LAT2	7462	HITS-CLIP	Functional MTI (Weak)	23824327
MIRT665131	hsa-miR-194-3p	EMC2	9694	HITS-CLIP	Functional MTI (Weak)	23824327
MIRT667938	hsa-miR-194-3p	HYPK	25764	HITS-CLIP	Functional MTI (Weak)	23824327
MIRT668481	hsa-miR-194-3p	EXOSC2	23404	HITS-CLIP	Functional MTI (Weak)	23824327
MIRT700629	hsa-miR-194-3p	PRDM10	56980	HITS-CLIP	Functional MTI (Weak)	23313552
MIRT709486	hsa-miR-194-3p	LOXL2	4017	HITS-CLIP	Functional MTI (Weak)	19536157
MIRT711951	hsa-miR-194-3p	SLC7A14	57709	HITS-CLIP	Functional MTI (Weak)	19536157
MIRT721123	hsa-miR-194-3p	PSMC2	5701	HITS-CLIP	Functional MTI (Weak)	19536157
MIRT724619	hsa-miR-194-3p	SNAP25	6616	HITS-CLIP	Functional MTI (Weak)	19536157
MIRT735492	hsa-miR-194-3p	ARHGEF2	9181	Luciferase reporter assay//qRT-PCR//Western blot	Functional MTI	27573550
MIRT738581	hsa-miR-194-3p	ADRM1	11047	PAR-CLIP	Functional MTI (Weak)	26701625
MIRT738582	hsa-miR-194-3p	ALDH9A1	223	PAR-CLIP	Functional MTI (Weak)	26701625
MIRT738583	hsa-miR-194-3p	ARPIN	348110	PAR-CLIP	Functional MTI (Weak)	26701625
MIRT738584	hsa-miR-194-3p	ARRB2	409	PAR-CLIP	Functional MTI (Weak)	26701625
MIRT738585	hsa-miR-194-3p	CARM1	10498	PAR-CLIP	Functional MTI (Weak)	26701625
MIRT738586	hsa-miR-194-3p	CDCP1	64866	PAR-CLIP	Functional MTI (Weak)	26701625
MIRT738587	hsa-miR-194-3p	ECE1	1889	PAR-CLIP	Functional MTI (Weak)	26701625
MIRT738588	hsa-miR-194-3p	ESYT1	23344	PAR-CLIP	Functional MTI (Weak)	26701625
MIRT738589	hsa-miR-194-3p	GLG1	2734	PAR-CLIP	Functional MTI (Weak)	26701625
MIRT738590	hsa-miR-194-3p	GPRC5A	9052	PAR-CLIP	Functional MTI (Weak)	26701625
MIRT738591	hsa-miR-194-3p	HDGF	3068	PAR-CLIP	Functional MTI (Weak)	26701625
MIRT738592	hsa-miR-194-3p	HSP90AB1	3326	PAR-CLIP	Functional MTI (Weak)	26701625
MIRT738593	hsa-miR-194-3p	MAPK8IP3	23162	PAR-CLIP	Functional MTI (Weak)	26701625
MIRT738594	hsa-miR-194-3p	NACC1	112939	PAR-CLIP	Functional MTI (Weak)	26701625
MIRT738595	hsa-miR-194-3p	NPLOC4	55666	PAR-CLIP	Functional MTI (Weak)	26701625
MIRT738596	hsa-miR-194-3p	NR1H2	7376	PAR-CLIP	Functional MTI (Weak)	26701625
MIRT738597	hsa-miR-194-3p	PHLDA3	23612	PAR-CLIP	Functional MTI (Weak)	26701625
MIRT738598	hsa-miR-194-3p	PTPN1	5770	PAR-CLIP	Functional MTI (Weak)	26701625
MIRT738599	hsa-miR-194-3p	RAB11FIP4	84440	PAR-CLIP	Functional MTI (Weak)	26701625
MIRT738600	hsa-miR-194-3p	SEC61A1	29927	PAR-CLIP	Functional MTI (Weak)	26701625
MIRT738601	hsa-miR-194-3p	TK1	7083	PAR-CLIP	Functional MTI (Weak)	26701625
MIRT738602	hsa-miR-194-3p	TRMU	55687	PAR-CLIP	Functional MTI (Weak)	26701625
MIRT738603	hsa-miR-194-3p	YARS	8565	PAR-CLIP	Functional MTI (Weak)	26701625
MIRT738604	hsa-miR-194-3p	ZBTB7A	51341	PAR-CLIP	Functional MTI (Weak)	26701625
MIRT763455	hsa-miR-194-3p	DUSP2	1844	PAR-CLIP	Functional MTI (Weak)	27292025
MIRT763456	hsa-miR-194-3p	FBXO48	554251	PAR-CLIP	Functional MTI (Weak)	27292025
MIRT763457	hsa-miR-194-3p	GRK2	156	PAR-CLIP	Functional MTI (Weak)	27292025
MIRT763458	hsa-miR-194-3p	ISCU	23479	PAR-CLIP	Functional MTI (Weak)	27292025
MIRT763459	hsa-miR-194-3p	LIPG	9388	PAR-CLIP	Functional MTI (Weak)	27292025
MIRT763460	hsa-miR-194-3p	MINK1	50488	PAR-CLIP	Functional MTI (Weak)	27292025
MIRT763461	hsa-miR-194-3p	MRPS23	51649	PAR-CLIP	Functional MTI (Weak)	27292025
MIRT763462	hsa-miR-194-3p	ORC6	23594	PAR-CLIP	Functional MTI (Weak)	27292025
MIRT763463	hsa-miR-194-3p	TTC22	55001	PAR-CLIP	Functional MTI (Weak)	27292025
MIRT790147	hsa-miR-194-3p	ITPA	3704	HITS-CLIP	Functional MTI (Weak)	28735896
MIRT790148	hsa-miR-194-3p	PQLC1	80148	HITS-CLIP	Functional MTI (Weak)	28735896
MIRT000020	hsa-miR-148a-3p	DNMT1	1786	Luciferase reporter assay	Functional MTI	20146264
MIRT000297	hsa-miR-148a-3p	HLA-G	3135	Luciferase reporter assay	Functional MTI	17847008
MIRT000298	hsa-miR-148a-3p	TGIF2	60436	Luciferase reporter assay//Western blot	Functional MTI	18768788
MIRT000955	hsa-miR-148a-3p	DNMT3B	1789	GFP reporter assay//Western blot//qRT-PCR	Functional MTI	18367714
MIRT003998	hsa-miR-148a-3p	NR1I2	8856	Luciferase reporter assay//qRT-PCR	Functional MTI	18268015
MIRT004504	hsa-miR-148a-3p	RPS6KA5	9252	qRT-PCR//Luciferase reporter assay//Western blot//Reporter assay	Functional MTI	20406806
MIRT005898	hsa-miR-148a-3p	CCKBR	887	Luciferase reporter assay//Microarray	Functional MTI	21168126
MIRT006859	hsa-miR-148a-3p	IRS1	3667	Luciferase reporter assay//Western blot	Functional MTI	22935141
MIRT006946	hsa-miR-148a-3p	ACVR1	90	Luciferase reporter assay	Functional MTI	22408438
MIRT006975	hsa-miR-148a-3p	BCL2	596	Luciferase reporter assay	Functional MTI	21455217
MIRT007017	hsa-miR-148a-3p	TMED7	51014	Luciferase reporter assay	Functional MTI	21552422
MIRT025970	hsa-miR-148a-3p	GPATCH8	23131	Sequencing	Functional MTI (Weak)	20371350
MIRT025971	hsa-miR-148a-3p	TMEM14A	28978	Sequencing	Functional MTI (Weak)	20371350
MIRT025972	hsa-miR-148a-3p	ANP32A	8125	Sequencing	Functional MTI (Weak)	20371350
MIRT025973	hsa-miR-148a-3p	RAB1B	81876	Sequencing	Functional MTI (Weak)	20371350
MIRT025974	hsa-miR-148a-3p	HSP90B1	7184	Sequencing	Functional MTI (Weak)	20371350
MIRT025975	hsa-miR-148a-3p	POFUT1	23509	Sequencing	Functional MTI (Weak)	20371350
MIRT025976	hsa-miR-148a-3p	CYCS	54205	Sequencing	Functional MTI (Weak)	20371350
MIRT025977	hsa-miR-148a-3p	ADARB1	104	Sequencing	Functional MTI (Weak)	20371350
MIRT025978	hsa-miR-148a-3p	CBX3	11335	Sequencing	Functional MTI (Weak)	20371350
MIRT025979	hsa-miR-148a-3p	UQCRQ	27089	Sequencing	Functional MTI (Weak)	20371350
MIRT025980	hsa-miR-148a-3p	SPRY2	10253	Sequencing	Functional MTI (Weak)	20371350
MIRT025981	hsa-miR-148a-3p	PAN3	255967	Sequencing	Functional MTI (Weak)	20371350
MIRT025982	hsa-miR-148a-3p	KANSL1	284058	Sequencing	Functional MTI (Weak)	20371350
MIRT025983	hsa-miR-148a-3p	GAS1	2619	Sequencing//PAR-CLIP	Functional MTI (Weak)	20371350
MIRT025984	hsa-miR-148a-3p	PTPN4	5775	Sequencing	Functional MTI (Weak)	20371350
MIRT025985	hsa-miR-148a-3p	ZNF92	168374	Sequencing	Functional MTI (Weak)	20371350
MIRT025986	hsa-miR-148a-3p	RAB10	10890	Sequencing	Functional MTI (Weak)	20371350
MIRT025987	hsa-miR-148a-3p	PAPD4	167153	Sequencing	Functional MTI (Weak)	20371350
MIRT025988	hsa-miR-148a-3p	HCCS	3052	Sequencing	Functional MTI (Weak)	20371350
MIRT025989	hsa-miR-148a-3p	WAPL	23063	Sequencing	Functional MTI (Weak)	20371350
MIRT025990	hsa-miR-148a-3p	MPP5	64398	Sequencing	Functional MTI (Weak)	20371350
MIRT025991	hsa-miR-148a-3p	ZNF490	57474	Sequencing	Functional MTI (Weak)	20371350
MIRT025992	hsa-miR-148a-3p	RAB12	201475	Sequencing	Functional MTI (Weak)	20371350
MIRT025993	hsa-miR-148a-3p	GNB5	10681	Sequencing	Functional MTI (Weak)	20371350
MIRT025994	hsa-miR-148a-3p	SNAPIN	23557	Sequencing	Functional MTI (Weak)	20371350
MIRT025995	hsa-miR-148a-3p	PSMD9	5715	Sequencing	Functional MTI (Weak)	20371350
MIRT025996	hsa-miR-148a-3p	TRIM59	286827	Sequencing	Functional MTI (Weak)	20371350
MIRT025997	hsa-miR-148a-3p	DYNLL2	140735	Sequencing	Functional MTI (Weak)	20371350
MIRT025998	hsa-miR-148a-3p	SECISBP2L	9728	Sequencing//PAR-CLIP	Functional MTI (Weak)	20371350
MIRT025999	hsa-miR-148a-3p	LYSMD1	388695	Sequencing	Functional MTI (Weak)	20371350
MIRT026000	hsa-miR-148a-3p	PBXIP1	57326	Sequencing	Functional MTI (Weak)	20371350
MIRT026001	hsa-miR-148a-3p	MTMR9	66036	Sequencing	Functional MTI (Weak)	20371350
MIRT026002	hsa-miR-148a-3p	DNAJB4	11080	Sequencing	Functional MTI (Weak)	20371350
MIRT026003	hsa-miR-148a-3p	DSTYK	25778	Sequencing	Functional MTI (Weak)	20371350
MIRT026004	hsa-miR-148a-3p	LBR	3930	Sequencing	Functional MTI (Weak)	20371350
MIRT026005	hsa-miR-148a-3p	KIAA1549	57670	Sequencing	Functional MTI (Weak)	20371350
MIRT026006	hsa-miR-148a-3p	DYRK1A	1859	Sequencing	Functional MTI (Weak)	20371350
MIRT026007	hsa-miR-148a-3p	CDK19	23097	Sequencing	Functional MTI (Weak)	20371350
MIRT026008	hsa-miR-148a-3p	RAB34	83871	Sequencing	Functional MTI (Weak)	20371350
MIRT026009	hsa-miR-148a-3p	ARRDC3	57561	Sequencing	Functional MTI (Weak)	20371350
MIRT026010	hsa-miR-148a-3p	PRNP	5621	Sequencing	Functional MTI (Weak)	20371350
MIRT026011	hsa-miR-148a-3p	HOXC8	3224	Sequencing	Functional MTI (Weak)	20371350
MIRT026012	hsa-miR-148a-3p	TMEM9B	56674	Sequencing	Functional MTI (Weak)	20371350
MIRT026013	hsa-miR-148a-3p	RASSF8	11228	Sequencing	Functional MTI (Weak)	20371350

MIRT026014	hsa-miR-148a-3p	BTBD3	22903	Sequencing//PAR-CLIP	Functional MTI (Weak)	20371350
MIRT026015	hsa-miR-148a-3p	TNRC6A	27327	Sequencing	Functional MTI (Weak)	20371350
MIRT026016	hsa-miR-148a-3p	SESTD1	91404	Sequencing	Functional MTI (Weak)	20371350
MIRT026017	hsa-miR-148a-3p	CDC25B	994	Reporter assay;Western blot;Other	Functional MTI	21709669
MIRT048023	hsa-miR-148a-3p	MRPL45	84311	CLASH	Functional MTI (Weak)	23622248
MIRT048024	hsa-miR-148a-3p	DENR	8562	CLASH	Functional MTI (Weak)	23622248
MIRT048025	hsa-miR-148a-3p	APPBP2	10513	CLASH	Functional MTI (Weak)	23622248
MIRT048026	hsa-miR-148a-3p	SLC2A3	6515	CLASH	Functional MTI (Weak)	23622248
MIRT048027	hsa-miR-148a-3p	PTPN23	25930	CLASH	Functional MTI (Weak)	23622248
MIRT048028	hsa-miR-148a-3p	VPS41	27072	CLASH	Functional MTI (Weak)	23622248
MIRT048029	hsa-miR-148a-3p	MSL3	10943	CLASH	Functional MTI (Weak)	23622248
MIRT048030	hsa-miR-148a-3p	AMELX	265	CLASH	Functional MTI (Weak)	23622248
MIRT048031	hsa-miR-148a-3p	OR2C3	81472	CLASH	Functional MTI (Weak)	23622248
MIRT048032	hsa-miR-148a-3p	SLC25A3	5250	CLASH	Functional MTI (Weak)	23622248
MIRT048033	hsa-miR-148a-3p	APC	324	CLASH	Functional MTI (Weak)	23622248
MIRT048034	hsa-miR-148a-3p	GOLIM4	27333	CLASH	Functional MTI (Weak)	23622248
MIRT048035	hsa-miR-148a-3p	MYCBP2	23077	CLASH	Functional MTI (Weak)	23622248
MIRT048036	hsa-miR-148a-3p	RPS17	6218	CLASH	Functional MTI (Weak)	23622248
MIRT048037	hsa-miR-148a-3p	HSPA4	3308	CLASH	Functional MTI (Weak)	23622248
MIRT048038	hsa-miR-148a-3p	WDTC1	23038	CLASH	Functional MTI (Weak)	23622248
MIRT048039	hsa-miR-148a-3p	HMGB1	3146	CLASH	Functional MTI (Weak)	23622248
MIRT048040	hsa-miR-148a-3p	MAP3K4	4216	CLASH	Functional MTI (Weak)	23622248
MIRT048041	hsa-miR-148a-3p	USP38	84640	CLASH	Functional MTI (Weak)	23622248
MIRT048042	hsa-miR-148a-3p	NONO	4841	CLASH	Functional MTI (Weak)	23622248
MIRT048043	hsa-miR-148a-3p	CCNI	10983	CLASH	Functional MTI (Weak)	23622248
MIRT048044	hsa-miR-148a-3p	AURKB	9212	CLASH	Functional MTI (Weak)	23622248
MIRT052917	hsa-miR-148a-3p	MMP7	4316	In situ hybridization//Microarray//qRT-PCR//Western blot//Luciferase report	Functional MTI	24283384
MIRT053185	hsa-miR-148a-3p	WNT10B	7480	Luciferase reporter assay//Microarray//qRT-PCR//Western blot	Functional MTI	22890324
MIRT053199	hsa-miR-148a-3p	MYC	4609	Immunoblot//Microarray//qRT-PCR	Functional MTI (Weak)	23389829
MIRT053475	hsa-miR-148a-3p	CDKN1B	1027	In situ hybridization//Luciferase reporter assay//Northern blot//Western blot	Functional MTI	21552422
MIRT053477	hsa-miR-148a-3p	SERPINE1	5054	Luciferase reporter assay//Microarray//qRT-PCR	Functional MTI	21703006
MIRT053478	hsa-miR-148a-3p	ITGB8	3696	Luciferase reporter assay//Microarray//qRT-PCR	Functional MTI	21703006
MIRT053479	hsa-miR-148a-3p	VAV2	7410	Luciferase reporter assay//Microarray//qRT-PCR	Functional MTI	21703006
MIRT053480	hsa-miR-148a-3p	ITGA5	3678	Luciferase reporter assay//Microarray//qRT-PCR	Functional MTI	21703006
MIRT053483	hsa-miR-148a-3p	ROCK1	6093	Immunohistochemistry//Luciferase reporter assay//qRT-PCR//Western blot	Functional MTI	21994419
MIRT053518	hsa-miR-148a-3p	RUNX3	864	qRT-PCR//Western blot	Functional MTI	23549984
MIRT053560	hsa-miR-148a-3p	SMAD2	4087	Luciferase reporter assay//qRT-PCR//Western blot	Functional MTI	23873106
MIRT054115	hsa-miR-148a-3p	UNKL	64718	Microarray	Functional MTI (Weak)	22995316
MIRT054388	hsa-miR-148a-3p	MET	4233	Immunoblot//Immunofluorescence//Immunohistochemistry//Luciferase repor	Functional MTI	24013226
MIRT057492	hsa-miR-148a-3p	CEP55	55165	PAR-CLIP	Functional MTI (Weak)	21572407
MIRT062708	hsa-miR-148a-3p	MLEC	9761	PAR-CLIP	Functional MTI (Weak)	21572407
MIRT084562	hsa-miR-148a-3p	BCL2L11	10018	Immunoblot//Luciferase reporter assay//qRT-PCR	Functional MTI	24425048
MIRT105304	hsa-miR-148a-3p	VPS37A	137492	PAR-CLIP	Functional MTI (Weak)	21572407
MIRT130076	hsa-miR-148a-3p	TXNIP	10628	PAR-CLIP	Functional MTI (Weak)	24398324
MIRT138426	hsa-miR-148a-3p	KIF2C	11004	PAR-CLIP	Functional MTI (Weak)	23592263
MIRT152411	hsa-miR-148a-3p	ARID3A	1820	PAR-CLIP	Functional MTI (Weak)	23592263
MIRT155749	hsa-miR-148a-3p	SIK1	150094	PAR-CLIP	Functional MTI (Weak)	23592263
MIRT210293	hsa-miR-148a-3p	ARL8B	55207	PAR-CLIP	Functional MTI (Weak)	23592263
MIRT218522	hsa-miR-148a-3p	HLA-A	3105	PAR-CLIP	Functional MTI (Weak)	23592263
MIRT222270	hsa-miR-148a-3p	CCT6A	908	PAR-CLIP	Functional MTI (Weak)	21572407
MIRT270936	hsa-miR-148a-3p	GPRC5A	9052	PAR-CLIP	Functional MTI (Weak)	23592263
MIRT280554	hsa-miR-148a-3p	GLRX5	51218	PAR-CLIP	Functional MTI (Weak)	21572407
MIRT281136	hsa-miR-148a-3p	PDIA3	2923	Luciferase reporter assay//qRT-PCR//Western blot	Functional MTI	26004124
MIRT296526	hsa-miR-148a-3p	STX16	8675	PAR-CLIP	Functional MTI (Weak)	23592263
MIRT301572	hsa-miR-148a-3p	TNRC6B	23112	PAR-CLIP	Functional MTI (Weak)	23592263
MIRT347400	hsa-miR-148a-3p	CEBPG	1054	PAR-CLIP	Functional MTI (Weak)	20371350
MIRT382244	hsa-miR-148a-3p	SH3PXD2A	9644	PAR-CLIP	Functional MTI (Weak)	23592263
MIRT399584	hsa-miR-148a-3p	RBM38	55544	PAR-CLIP	Functional MTI (Weak)	23592263
MIRT437409	hsa-miR-148a-3p	MAFB	9935	Luciferase reporter assay	Functional MTI	23225151
MIRT438088	hsa-miR-148a-3p	ERRFI1	54206	Immunoblot//Luciferase reporter assay//qRT-PCR	Functional MTI	24425048
MIRT438751	hsa-miR-148a-3p	S1PR1	1901	Immunohistochemistry//Luciferase reporter assay//qRT-PCR//Western blot	Functional MTI	24798342
MIRT438752	hsa-miR-148a-3p	USP4	7375	Immunohistochemistry//Luciferase reporter assay//qRT-PCR//Western blot	Functional MTI	24798342
MIRT453164	hsa-miR-148a-3p	CNOT4	4850	PAR-CLIP	Functional MTI (Weak)	23592263
MIRT456196	hsa-miR-148a-3p	ZDHHC6	64429	PAR-CLIP	Functional MTI (Weak)	23592263
MIRT458103	hsa-miR-148a-3p	TTLL1	25809	PAR-CLIP	Functional MTI (Weak)	23592263
MIRT459958	hsa-miR-148a-3p	POC1A	25886	PAR-CLIP	Functional MTI (Weak)	23592263
MIRT461329	hsa-miR-148a-3p	MRPS27	23107	PAR-CLIP	Functional MTI (Weak)	23592263
MIRT462848	hsa-miR-148a-3p	B4GALT7	11285	PAR-CLIP	Functional MTI (Weak)	23592263
MIRT463247	hsa-miR-148a-3p	ZIC5	85416	PAR-CLIP	Functional MTI (Weak)	23592263
MIRT464041	hsa-miR-148a-3p	WASL	8976	PAR-CLIP	Functional MTI (Weak)	23592263
MIRT466869	hsa-miR-148a-3p	STX6	10228	PAR-CLIP	Functional MTI (Weak)	23592263
MIRT467697	hsa-miR-148a-3p	SLC38A2	54407	PAR-CLIP	Functional MTI (Weak)	23592263
MIRT468920	hsa-miR-148a-3p	RPS6KA4	8986	PAR-CLIP	Functional MTI (Weak)	23592263
MIRT469506	hsa-miR-148a-3p	RCC2	55920	PAR-CLIP	Functional MTI (Weak)	23592263
MIRT469815	hsa-miR-148a-3p	RAB14	51552	PAR-CLIP	Functional MTI (Weak)	23592263
MIRT470329	hsa-miR-148a-3p	PPP6R1	22870	PAR-CLIP	Functional MTI (Weak)	23592263
MIRT473770	hsa-miR-148a-3p	MAP3K9	4293	PAR-CLIP	Functional MTI (Weak)	23592263
MIRT477363	hsa-miR-148a-3p	EOGT	285203	PAR-CLIP	Functional MTI (Weak)	23592263
MIRT478215	hsa-miR-148a-3p	DDX6	1656	PAR-CLIP	Functional MTI (Weak)	23592263
MIRT479812	hsa-miR-148a-3p	CCNA2	890	PAR-CLIP	Functional MTI (Weak)	23592263
MIRT484939	hsa-miR-148a-3p	ZFYVE26	23503	PAR-CLIP	Functional MTI (Weak)	23592263
MIRT485432	hsa-miR-148a-3p	KLF6	1316	PAR-CLIP	Functional MTI (Weak)	23592263
MIRT485645	hsa-miR-148a-3p	DICER1	23405	PAR-CLIP	Functional MTI (Weak)	23592263
MIRT487938	hsa-miR-148a-3p	HLA-C	3107	PAR-CLIP	Functional MTI (Weak)	23592263
MIRT488937	hsa-miR-148a-3p	ETV7	51513	PAR-CLIP	Functional MTI (Weak)	23592263
MIRT492821	hsa-miR-148a-3p	PATL1	219988	PAR-CLIP	Functional MTI (Weak)	23592263
MIRT496749	hsa-miR-148a-3p	PDIK1L	149420	PAR-CLIP	Functional MTI (Weak)	22291592
MIRT497883	hsa-miR-148a-3p	SLC12A7	10723	PAR-CLIP	Functional MTI (Weak)	22291592
MIRT500918	hsa-miR-148a-3p	STARD13	90627	PAR-CLIP	Functional MTI (Weak)	24398324
MIRT503176	hsa-miR-148a-3p	AGO2	27161	PAR-CLIP	Functional MTI (Weak)	24398324
MIRT505102	hsa-miR-148a-3p	YWHAB	7529	PAR-CLIP	Functional MTI (Weak)	23446348
MIRT505256	hsa-miR-148a-3p	UBE2D3	7323	PAR-CLIP	Functional MTI (Weak)	23446348
MIRT511220	hsa-miR-148a-3p	LNPEP	4012	PAR-CLIP	Functional MTI (Weak)	23446348
MIRT513209	hsa-miR-148a-3p	RFT1	91869	PAR-CLIP	Functional MTI (Weak)	23446348
MIRT513617	hsa-miR-148a-3p	VPS37B	79720	PAR-CLIP	Functional MTI (Weak)	23446348
MIRT522804	hsa-miR-148a-3p	KPNA4	3840	PAR-CLIP	Functional MTI (Weak)	23446348
MIRT525443	hsa-miR-148a-3p	RBM23	55147	PAR-CLIP	Functional MTI (Weak)	22012620
MIRT530302	hsa-miR-148a-3p	AKAP17A	8227	PAR-CLIP	Functional MTI (Weak)	22012620
MIRT531689	hsa-miR-148a-3p	MYO3A	53904	PAR-CLIP	Functional MTI (Weak)	22012620
MIRT537300	hsa-miR-148a-3p	FZD5	7855	PAR-CLIP	Functional MTI (Weak)	22012620
MIRT541199	hsa-miR-148a-3p	HSP90AA1	3320	PAR-CLIP	Functional MTI (Weak)	21572407
MIRT544732	hsa-miR-148a-3p	NDRG1	10397	PAR-CLIP	Functional MTI (Weak)	21572407
MIRT549174	hsa-miR-148a-3p	BMP3	651	PAR-CLIP	Functional MTI (Weak)	21572407
MIRT549231	hsa-miR-148a-3p	BAZ2B	29994	PAR-CLIP	Functional MTI (Weak)	21572407
MIRT551864	hsa-miR-148a-3p	ASB6	140459	PAR-CLIP	Functional MTI (Weak)	21572407

MIRT559687	hsa-miR-148a-3p	AGO3	192669	PAR-CLIP	Functional MTI (Weak)	21572407
MIRT561658	hsa-miR-148a-3p	RNF219	79596	PAR-CLIP	Functional MTI (Weak)	20371350
MIRT561856	hsa-miR-148a-3p	NPTX1	4884	PAR-CLIP	Functional MTI (Weak)	20371350
MIRT565733	hsa-miR-148a-3p	SESN3	143686	PAR-CLIP	Functional MTI (Weak)	20371350
MIRT566568	hsa-miR-148a-3p	OTUD4	54726	PAR-CLIP	Functional MTI (Weak)	20371350
MIRT567186	hsa-miR-148a-3p	IGFBP5	3488	PAR-CLIP	Functional MTI (Weak)	20371350
MIRT567834	hsa-miR-148a-3p	DCUN1D3	123879	PAR-CLIP	Functional MTI (Weak)	20371350
MIRT574794	hsa-miR-148a-3p	FAM104A	84923	PAR-CLIP	Functional MTI (Weak)	20371350
MIRT610545	hsa-miR-148a-3p	WNT2B	7482	HITS-CLIP	Functional MTI (Weak)	23824327
MIRT613042	hsa-miR-148a-3p	FOXP1	27086	HITS-CLIP	Functional MTI (Weak)	23824327
MIRT615073	hsa-miR-148a-3p	COLEC12	81035	HITS-CLIP	Functional MTI (Weak)	23824327
MIRT617481	hsa-miR-148a-3p	AP5B1	91056	HITS-CLIP	Functional MTI (Weak)	23824327
MIRT622753	hsa-miR-148a-3p	PHACTR2	9749	HITS-CLIP	Functional MTI (Weak)	23824327
MIRT625516	hsa-miR-148a-3p	PLPP4	196051	HITS-CLIP	Functional MTI (Weak)	23824327
MIRT628641	hsa-miR-148a-3p	ABLIM1	3983	HITS-CLIP	Functional MTI (Weak)	23824327
MIRT630234	hsa-miR-148a-3p	SORD	6652	HITS-CLIP	Functional MTI (Weak)	23824327
MIRT635946	hsa-miR-148a-3p	PLA2G12A	81579	HITS-CLIP	Functional MTI (Weak)	23824327
MIRT646776	hsa-miR-148a-3p	IL23R	149233	HITS-CLIP	Functional MTI (Weak)	23824327
MIRT648163	hsa-miR-148a-3p	CHRFAM7A	89832	HITS-CLIP	Functional MTI (Weak)	23824327
MIRT653244	hsa-miR-148a-3p	SOS2	6655	HITS-CLIP	Functional MTI (Weak)	23824327
MIRT661182	hsa-miR-148a-3p	S1PR2	9294	HITS-CLIP	Functional MTI (Weak)	23824327
MIRT693234	hsa-miR-148a-3p	KIAA0907	22889	HITS-CLIP	Functional MTI (Weak)	23313552
MIRT693776	hsa-miR-148a-3p	VGLL2	245806	HITS-CLIP	Functional MTI (Weak)	23313552
MIRT702589	hsa-miR-148a-3p	JARID2	3720	HITS-CLIP	Functional MTI (Weak)	23313552
MIRT715175	hsa-miR-148a-3p	DTX4	23220	HITS-CLIP	Functional MTI (Weak)	19536157
MIRT722871	hsa-miR-148a-3p	FAM212B	55924	HITS-CLIP	Functional MTI (Weak)	19536157
MIRT723139	hsa-miR-148a-3p	YPEL1	29799	HITS-CLIP	Functional MTI (Weak)	19536157
MIRT723186	hsa-miR-148a-3p	OVOL1	5017	HITS-CLIP	Functional MTI (Weak)	19536157
MIRT732353	hsa-miR-148a-3p	WNT1	7471	Immunohistochemistry//In situ hybridization//Luciferase reporter assay//qRT	Functional MTI	28199399
MIRT732362	hsa-miR-148a-3p	STAT3	6774	Luciferase reporter assay//qRT-PCR//Western blot	Functional MTI	28280370
MIRT732662	hsa-miR-148a-3p	ALCAM	214	Luciferase reporter assay//qRT-PCR//Western blot	Functional MTI	28063929
MIRT732882	hsa-miR-148a-3p	INO80	54617	Luciferase reporter assay//Western blot	Functional MTI	27764804
MIRT733135	hsa-miR-148a-3p	TGFB2	7042	Flow//Immunofluorescence//Luciferase reporter assay//qRT-PCR//Western b	Functional MTI	26983401
MIRT733211	hsa-miR-148a-3p	IKBKB	3551	Luciferase reporter assay//Microarray//Next Generation Sequencing (NGS)//	Functional MTI	25630970
MIRT734355	hsa-miR-148a-3p	BAX	581	Luciferase reporter assay//qRT-PCR//Western blot	Functional MTI	27261578
MIRT735024	hsa-miR-148a-3p	QKI	9444	Luciferase reporter assay	Functional MTI	25971746
MIRT735317	hsa-miR-148a-3p	NRP1	8829	Immunohistochemistry//Luciferase reporter assay//qRT-PCR//Western blot	Functional MTI	26097868
MIRT737664	hsa-miR-148a-3p	APLP2	334	PAR-CLIP	Functional MTI (Weak)	26701625
MIRT737665	hsa-miR-148a-3p	CDKN1A	1026	PAR-CLIP	Functional MTI (Weak)	26701625
MIRT737666	hsa-miR-148a-3p	CSNK2A1	1457	PAR-CLIP	Functional MTI (Weak)	26701625
MIRT737667	hsa-miR-148a-3p	FURIN	5045	PAR-CLIP	Functional MTI (Weak)	26701625
MIRT737668	hsa-miR-148a-3p	FXR1	8087	PAR-CLIP	Functional MTI (Weak)	26701625
MIRT737669	hsa-miR-148a-3p	KIAA1456	57604	PAR-CLIP	Functional MTI (Weak)	26701625
MIRT737670	hsa-miR-148a-3p	PHLDB2	90102	PAR-CLIP	Functional MTI (Weak)	26701625
MIRT737671	hsa-miR-148a-3p	PPARD	5467	PAR-CLIP	Functional MTI (Weak)	26701625
MIRT737672	hsa-miR-148a-3p	RALY	22913	PAR-CLIP	Functional MTI (Weak)	26701625
MIRT762747	hsa-miR-148a-3p	ARL6IP1	23204	PAR-CLIP	Functional MTI (Weak)	27292025
MIRT762748	hsa-miR-148a-3p	KDM6B	23135	PAR-CLIP	Functional MTI (Weak)	27292025
MIRT762749	hsa-miR-148a-3p	LDLR	3949	PAR-CLIP	Functional MTI (Weak)	27292025
MIRT762750	hsa-miR-148a-3p	MCFD2	90411	PAR-CLIP	Functional MTI (Weak)	27292025
MIRT762751	hsa-miR-148a-3p	NEURL4	84461	PAR-CLIP	Functional MTI (Weak)	27292025
MIRT762752	hsa-miR-148a-3p	TMEM246	84302	PAR-CLIP	Functional MTI (Weak)	27292025
MIRT790121	hsa-miR-148a-3p	APLN	8862	HITS-CLIP	Functional MTI (Weak)	28735896
