# Supplementary material for: Integrated MicroRNA Expression Profile Reveals Dysregulated miR-20a-5p and miR-200a-3p in Liver Fibrosis
Source: Biomed Res Int. 2021 Jun 8;2021:9583932. doi: 10.1155/2021/9583932 (PMC8218919; doi:10.1155/2021/9583932)
Supplement: Supplementary 2 — Table S2: GO functions for target genes of the three upregulated miRNAs. [file 9583932.f2.pdf]

Category	Term	Count	%	PValue	Genes	List Total	Pop Hits	Pop Total	Fold Enrichmen	Bonferroni	Benjamini	FDR
GOTERM BP DIRECT	GO:0045944~positive regulation of transcription from RNA polymerase II promote	70	15.11879	8.36E-15	CCNT2, ZNF292, THRB, FOXA2, TBK1, M	427	981	16792	2.806102839	2.33E-11	2.33E-11	1.49E-11
GOTERM BP DIRECT	GO:0043066~negative regulation of apoptotic process	39	8.4233261	1.17E-10	MTDH, MCL1, XIAP, BTC, FOXO1, PTEN,	427	455	16792	3.370759451	3.27E-07	1.64E-07	2.09E-07
GOTERM BP DIRECT	GO:0000122~negative regulation of transcription from RNA polymerase II promote	50	10.799136	3.07E-10	BMI1, THRB, FGF9, MITF, DICER1, EZH2,	427	720	16792	2.73093937	8.58E-07	2.86E-07	5.48E-07
GOTERM BP DIRECT	GO:0045893~positive regulation of transcription, DNA-templated	41	8.8552916	3.32E-10	E2F3, FOXA2, CRLF3, MITF, FOXO1, FOX	427	515	16792	3.130770105	9.28E-07	2.32E-07	5.93E-07
GOTERM BP DIRECT	GO:0008284~positive regulation of cell proliferation	35	7.5593952	3.53E-08	E2F3, FGF9, BTC, PTEN, IL31RA, TGFB2,	427	466	16792	2.953633997	9.87E-05	1.97E-05	6.31E-05
GOTERM BP DIRECT	GO:0043525~positive regulation of neuron apoptotic process	10	2.1598272	1.05E-06	EPHA7, JUN, TP53, FOXO3, PMAIP1, CASI	427	43	16792	9.14547138	0.002930634	4.89E-04	0.001875309
GOTERM BP DIRECT	GO:0008285~negative regulation of cell proliferation	29	6.2634989	1.10E-06	DLC1, CYP1B1, BAP1, ZEB1, SRF, PTEN,	427	396	16792	2.8798997	0.003068831	4.39E-04	0.001963875
GOTERM BP DIRECT	GO:0007265~Ras protein signal transduction	12	2.5917927	1.39E-06	PLD1, CDKN1A, KRAS, CRKL, GRB2, MAI	427	70	16792	6.741518903	0.003869596	4.85E-04	0.002477307
GOTERM BP DIRECT	GO:0010628~positive regulation of gene expression	22	4.7516199	3.60E-06	WNT16, ROCK2, FGF9, MITF, TP53, HFE,	427	262	16792	3.302143483	0.010022888	0.001118647	0.006436392
GOTERM BP DIRECT	GO:0006366~transcription from RNA polymerase II promoter	32	6.9114471	7.75E-06	CCNT2, ZNF292, MITF, DEK, NR3C1, FOX	427	513	16792	2.453054312	0.021426216	0.002163565	0.013838473
GOTERM BP DIRECT	GO:0001666~response to hypoxia	17	3.6717063	8.16E-06	LDHA, CYP1A1, ACTN4, CREB1, SMAD4,	427	172	16792	3.886825336	0.022550738	0.002071388	0.014573055
GOTERM BP DIRECT	GO:0016032~viral process	23	4.9676026	8.41E-06	VAC14, GRB2, CREB1, RBM15B, TP53, AI	427	299	16792	3.025040533	0.023237681	0.001957409	0.015022206
GOTERM BP DIRECT	GO:0006357~regulation of transcription from RNA polymerase II promoter	29	6.2634989	8.68E-06	HMGB1, FOXA2, MITF, DEK, ZEB1, FOXC	427	441	16792	2.586032383	0.023969913	0.001864557	0.015501316
GOTERM BP DIRECT	GO:0007173~epidermal growth factor receptor signaling pathway	10	2.1598272	1.05E-05	EGFR, PDPK1, KRAS, PLCG1, GRB2, PIK3	427	56	16792	7.022415524	0.029024198	0.002101626	0.018818174
GOTERM BP DIRECT	GO:0007050~cell cycle arrest	15	3.2397408	1.43E-05	TP53, SMAD3, CDK6, TCF7L2, TGFB2, NC	427	141	16792	4.183566695	0.039090211	0.002654786	0.025475378
GOTERM BP DIRECT	GO:0071364~cellular response to epidermal growth factor stimulus	8	1.7278618	1.55E-05	ZFP36L1, EGFR, BAG4, PDPK1, PLCG1, SI	427	33	16792	9.533461074	0.042517588	0.002711812	0.027757912
GOTERM BP DIRECT	GO:0048015~phosphatidylinositol-mediated signaling	13	2.8077754	1.56E-05	EGFR, IGF1R, PDPK1, FGF9, GRB2, PIK3C	427	106	16792	4.822941982	0.042564915	0.002555397	0.027789488
GOTERM BP DIRECT	GO:0001837~epithelial to mesenchymal transition	8	1.7278618	1.91E-05	NOTCH1, GSK3B, FOXF2, HGF, SNAI2, C	427	34	16792	9.25306516	0.052119849	0.002969317	0.034196239
GOTERM BP DIRECT	GO:0001701~in utero embryonic development	17	3.6717063	2.33E-05	GNA13, SMAD4, GJA1, SMAD3, KEAP1, S	427	187	16792	3.575047903	0.063152698	0.003427532	0.041674271
GOTERM BP DIRECT	GO:0006974~cellular response to DNA damage stimulus	18	3.887689	2.36E-05	XIAP, CENPS, TP53BP1, ZMAT3, TP53, FC	427	208	16792	3.4031706	0.063931543	0.003297883	0.042205472
GOTERM BP DIRECT	GO:0030330~DNA damage response, signal transduction by p53 class mediator	6	1.2958963	2.50E-05	ATRX, TP53, NDRG1, FOXO3, MDM4, SES	427	15	16792	15.73021077	0.067467206	0.00332071	0.044622458
GOTERM BP DIRECT	GO:0071456~cellular response to hypoxia	12	2.5917927	3.13E-05	ZFP36L1, DNMT3A, ERO1A, GATA6, BCL	427	96	16792	4.915690867	0.083812469	0.003970923	0.055915779
GOTERM BP DIRECT	GO:0051571~positive regulation of histone H3-K4 methylation	6	1.2958963	4.94E-05	SMAD4, DNMT1, OGT, MYB, DNMT3B, C	427	17	16792	13.87959774	0.128917874	0.005982858	0.088150639
GOTERM BP DIRECT	GO:0008283~cell proliferation	24	5.1835853	6.46E-05	EGFR, MORF4L1, CDV3, CYP1A1, FSCN1,	427	366	16792	2.578723078	0.165314435	0.007500902	0.115394722
GOTERM BP DIRECT	GO:0016477~cell migration	15	3.2397408	1.28E-04	PDPK1, FMNL3, FLT1, PLCG1, TIAM1, PO	427	172	16792	3.429551767	0.300920348	0.014217585	0.22848254
GOTERM BP DIRECT	GO:0008286~insulin receptor signaling pathway	10	2.1598272	1.55E-04	IGF1R, EIF4EBP2, GRB2, PIK3C2A, ATP6V	427	78	16792	5.041734222	0.351864877	0.016540754	0.27670812
GOTERM BP DIRECT	GO:0010718~positive regulation of epithelial to mesenchymal transition	7	1.511879	1.59E-04	NOTCH1, EZH2, SMAD4, SMAD3, SMAD2	427	33	16792	8.34177844	0.359600749	0.016370576	0.284358913
GOTERM BP DIRECT	GO:0006977~DNA damage response, signal transduction by p53 class mediator res	9	1.9438445	1.64E-04	TNKS1BP1, CDKN1A, CDKN1B, EP300, NI	427	62	16792	5.708544232	0.367281782	0.016214609	0.292046871
GOTERM BP DIRECT	GO:0045766~positive regulation of angiogenesis	12	2.5917927	1.64E-04	MTDH, CYP1B1, FLT1, PLCG1, ETS1, GA	427	115	16792	4.103533245	0.368348103	0.015717136	0.293121477
GOTERM BP DIRECT	GO:0097192~extrinsic apoptotic signaling pathway in absence of ligand	7	1.511879	1.89E-04	MCL1, BCL2, GSK3B, MKNK2, FOXO3, C	427	34	16792	8.096432015	0.411218317	0.017501695	0.337888256
GOTERM BP DIRECT	GO:0035924~cellular response to vascular endothelial growth factor stimulus	6	1.2958963	2.37E-04	VCAM1, NOTCH1, FLT1, MAPK14, VEGF	427	23	16792	10.25883311	0.484161208	0.021127206	0.422077563
GOTERM BP DIRECT	GO:0030308~negative regulation of cell growth	12	2.5917927	2.57E-04	DCBLD2, ACVR1B, CDKN1A, CDKN1B, C	427	121	16792	3.900052258	0.513215189	0.022246719	0.458956634
GOTERM BP DIRECT	GO:0030335~positive regulation of cell migration	15	3.2397408	2.59E-04	EGFR, FLT1, ACTN4, PODXL, SMAD3, HC	427	184	16792	3.205885348	0.51578531	0.021736847	0.462323598
GOTERM BP DIRECT	GO:0006915~apoptotic process	30	6.4794816	2.87E-04	DLC1, PHLPP1, XIAP, CADM1, STK17B, F	427	567	16792	2.080715711	0.552200057	0.023352673	0.51203577
GOTERM BP DIRECT	GO:0038128~ERBB2 signaling pathway	7	1.511879	3.57E-04	EGFR, CUL5, KRAS, GRB2, BTC, ERBIN,	427	38	16792	7.244176014	0.631457058	0.028117089	0.635785287
GOTERM BP DIRECT	GO:0001934~positive regulation of protein phosphorylation	12	2.5917927	3.92E-04	EGFR, WNT1, EPHA7, KRAS, CCND2, RO	427	127	16792	3.71579782	0.665637952	0.02997304	0.697563088
GOTERM BP DIRECT	GO:0048146~positive regulation of fibroblast proliferation	8	1.7278618	4.07E-04	BMI1, EGFR, WNT1, CDKN1A, JUN, BTC,	427	54	16792	5.82600399	0.679955129	0.030322495	0.725327343
GOTERM BP DIRECT	GO:0030036~actin cytoskeleton organization	12	2.5917927	4.78E-04	DLC1, PFN1, RND3, MTSS1, FMNL3, PDPI	427	130	16792	3.63004864	0.737587076	0.03459368	0.851188932
GOTERM BP DIRECT	GO:0032355~response to estradiol	10	2.1598272	4.99E-04	DNMT3A, CDKN1B, ETS1, EZH2, MBD4,	427	91	16792	4.321486476	0.752373003	0.035157648	0.887924027
GOTERM BP DIRECT	GO:0001570~vasculogenesis	8	1.7278618	5.11E-04	ZFP36L1, VEGFA, QKI, ZFPM2, YAP1, EPI	427	56	16792	5.617932419	0.760347657	0.035083924	0.908652234
GOTERM BP DIRECT	GO:0001889~liver development	9	1.9438445	5.60E-04	NOTCH1, KRAS, CADM1, GATA6, JUN, PI	427	74	16792	4.782834357	0.791264702	0.037491047	0.996067271
GOTERM BP DIRECT	GO:0045740~positive regulation of DNA replication	7	1.511879	6.23E-04	EGFR, IGF1R, JUN, NPM1, SHC1, AREG, A	427	42	16792	6.554254489	0.824772447	0.040620281	1.106699038
GOTERM BP DIRECT	GO:0008584~male gonad development	10	2.1598272	6.34E-04	FGF9, GATA6, BCL2, GATA4, HOXA9, M	427	94	16792	4.183566695	0.830069058	0.040379848	1.126092079
GOTERM BP DIRECT	GO:0043065~positive regulation of apoptotic process	19	4.1036717	6.83E-04	HMGB1, LDHA, RARG, CYP1B1, CREB1,	427	300	16792	2.490616706	0.852159737	0.042515719	1.214033222
GOTERM BP DIRECT	GO:0030336~negative regulation of cell migration	10	2.1598272	6.85E-04	RECK, DLC1, CYP1B1, RAP2C, BCL2, PTE	427	95	16792	4.139529151	0.852646059	0.041661027	1.21611299
GOTERM BP DIRECT	GO:0050821~protein stabilization	12	2.5917927	7.00E-04	PFN1, CDKN1A, FBXW7, EP300, PHB2, CF	427	136	16792	3.469899435	0.858899623	0.041677948	1.24348159
GOTERM BP DIRECT	GO:2000811~negative regulation of anoikis	5	1.0799136	7.48E-04	NOTCH1, MCL1, BCL2, PDK4, SNAI2	427	17	16792	11.56633145	0.876466515	0.04351917	1.327346055
GOTERM BP DIRECT	GO:0001702~gastrulation with mouth forming second	5	1.0799136	7.48E-04	LHX1, RNF2, SMAD4, FRS2, CTNNB1	427	17	16792	11.56633145	0.876466515	0.04351917	1.327346055
GOTERM BP DIRECT	GO:2000177~regulation of neural precursor cell proliferation	4	0.8639309	8.26E-04	FOXO1, FOXO3, TRIM71, SHCBP1	427	8	16792	19.66276347	0.900688982	0.046975395	1.46485713
GOTERM BP DIRECT	GO:0000077~DNA damage checkpoint	6	1.2958963	8.66E-04	TP53BP1, MAPK14, BRIP1, CHEK2, RAD1	427	30	16792	7.865105386	0.911379005	0.048253791	1.536535495
GOTERM BP DIRECT	GO:0008360~regulation of cell shape	12	2.5917927	8.91E-04	GNA13, DLC1, FMNL3, BRWD1, WASF3, I	427	140	16792	3.370759451	0.91728028	0.048624023	1.579881058
GOTERM BP DIRECT	GO:0045892~negative regulation of transcription, DNA-templated	26	5.6155508	0.001002	RBM15B, EZH2, FOXO1, ZEB1, TCEAL7,	427	499	16792	2.049025451	0.939378126	0.053479581	1.77514178
GOTERM BP DIRECT	GO:0042771~intrinsic apoptotic signaling pathway in response to DNA damage by	6	1.2958963	0.0010117	CDKN1A, EP300, TP53, CHEK2, PMAIP1, I	427	31	16792	7.611392309	0.940995228	0.05297114	1.792109664
GOTERM BP DIRECT	GO:0071479~cellular response to ionizing radiation	6	1.2958963	0.0010117	CDKN1A, GRB2, MAPK14, TP53, SNAI2, C	427	31	16792	7.611392309	0.940995228	0.05297114	1.792109664
GOTERM BP DIRECT	GO:0042981~regulation of apoptotic process	15	3.2397408	0.0011135	PHLPP1, MCL1, ACTN4, MITF, TP53, MAI	427	213	16792	2.769403305	0.955630797	0.057083494	1.970834364
GOTERM BP DIRECT	GO:0071230~cellular response to amino acid stimulus	7	1.511879	0.0011458	EGFR, DNMT3A, IPO5, DNMT1, ZEB1, SE	427	47	16792	5.856993373	0.959462322	0.057634	2.027388173
GOTERM BP DIRECT	GO:0006919~activation of cysteine-type endopeptidase activity involved in apoptot	9	1.9438445	0.0012032	TNFRSF10A, DLC1, CDKN1B, TNFRSF10I	427	83	16792	4.264213764	0.965473662	0.059365468	2.1278175
GOTERM BP DIRECT	GO:0060070~canonical Wnt signaling pathway	9	1.9438445	0.0012032	WNT1, RARG, GSK3B, SMAD3, SNAI2, PT	427	83	16792	4.264213764	0.965473662	0.059365468	2.1278175
GOTERM BP DIRECT	GO:0048617~embryonic foregut morphogenesis	4	0.8639309	0.0012152	GATA4, SMAD3, SMAD2, CTNNB1	427	9	16792	17.47801197	0.966621988	0.058905528	2.148968427
GOTERM BP DIRECT	GO:1901796~regulation of signal transduction by p53 class mediator	11	2.3758099	0.0012385	EP300, MAPK14, TP53, BRIP1, PRKAA2, C	427	124	16792	3.488554808	0.968724223	0.058977208	2.189633681
GOTERM BP DIRECT	GO:0018105~peptidyl-serine phosphorylation	11	2.3758099	0.0013165	PDPK1, PAK2, TBK1, MAPK14, BCL2, GSI	427	125	16792	3.46064637	0.974858528	0.061529708	2.325986387
GOTERM BP DIRECT	GO:0006928~movement of cell or subcellular component	9	1.9438445	0.0015155	GNA13, MTSS1, TUBB, ARPC3, MAPK14,	427	86	16792	4.115462121	0.985601977	0.069353402	2.673260587
GOTERM BP DIRECT	GO:0009749~response to glucose	8	1.7278618	0.0016464	LDHA, CDKN1B, GJA1, SMAD2, THBS1, S	427	68	16792	4.62653258	0.99001858	0.073909977	2.900831416
GOTERM BP DIRECT	GO:0046902~regulation of mitochondrial membrane permeability	4	0.8639309	0.0017035	BCL2, TP53, PMAIP1, STAT3	427	10	16792	15.73021077	0.991494273	0.075172271	3.000040807
GOTERM BP DIRECT	GO:0072593~reactive oxygen species metabolic process	6	1.2958963	0.0017788	BCL2, PDK4, PMAIP1, SESN2, SESN1, DDI	427	35	16792	6.741518903	0.993112749	0.077152856	3.130771774
GOTERM BP DIRECT	GO:0090116~C-5 methylation of cytosine	3	0.6479482	0.0018939	DNMT3A, DNMT1, DNMT3B	427	3	16792	39.32552693	0.995010837	0.080692597	3.330121979
GOTERM BP DIRECT	GO:0042493~response to drug	18	3.887689	0.0020355	DNMT3A, LDHA, CYP1A1, CREB1, UQCR	427	304	16792	2.328485147	0.996644437	0.085170661	3.574816926
GOTERM BP DIRECT	GO:0060045~positive regulation of cardiac muscle cell proliferation	5	1.0799136	0.0020787	NOTCH1, FGF9, GATA6, MAPK14, ZFPM2	427	22	16792	8.937619757	0.99702655	0.085619225	3.649275557
GOTERM BP DIRECT	GO:0048010~vascular endothelial growth factor receptor signaling pathway	8	1.7278618	0.0022947	FLT1, PAK2, ROCK2, MAPK14, VEGFA, RI	427	72	16792	4.369502992	0.998376718	0.092736909	4.021191248
GOTERM BP DIRECT	GO:0046849~bone remodeling	4	0.8639309	0.0022984	NOTCH2, WNT16, RASSF2, EPHA2	427	11	16792	14.30019161	0.998393652	0.091560279	4.027622276
GOTERM BP DIRECT	GO:0048646~anatomical structure formation involved in morphogenesis	4	0.8639309	0.0022984	GRB2, LHX1, DLX5, GATA4	427	11	16792	14.30019161	0.998393652	0.091560279	4.027622276
GOTERM BP DIRECT	GO:0042770~signal transduction in response to DNA damage	4	0.8639309	0.0022984	WNT1, GRB2, MAPK14, CHEK2	427	11	16792	14.30019161	0.998393652	0.091560279	4.027622276
GOTERM BP DIRECT	GO:0007179~transforming growth factor beta receptor signaling pathway	9	1.9438445	0.0023316	JUN, CREB1, FERMT2, SMAD4, RHOA, SI	427	92	16792	3.847062417	0.99853607	0.091517692	4.084537168
GOTERM BP DIRECT	GO:0016569~covalent chromatin modification	10	2.1598272	0.0023354	BMI1, ATRX, SATB2, BRD3, FOXA2, SMA	427	113	16792	3.480135127	0.998551894	0.090396382	4.091197547
GOTERM BP DIRECT	GO:0030316~osteoclast differentiation	5	1.0799136	0.0024665	TFRC, MAPK14, OSTM1, EPHA2, CTNNB1	427	23	16792	8.549027594	0.998996974	0.093930309	4.315984519
GOTERM BP DIRECT	GO:0007507~heart development	13	2.8077754	0.002513	GJA1, SRF, PTEN, CITED2, TGFB2, SHOX	427	183	16792	2.793616667	0.99911954	0.094334666	4.395634694
GOTERM BP DIRECT	GO:0030182~neuron differentiation	9	1.9438445	0.0028516	DNMT3A, WNT16, PTPRD, ADNP2, NLGN	427	95	16792	3.725576236	0.999659238	0.104965851	4.973763195
GOTERM BP DIRECT	GO:0048041~focal adhesion assembly	5	1.0799136	0.0029012	DLC1, PDPK1, RCC2, BCL2, FERMT2	427	24	16792	8.192818111	0.999703509	0.105312451	5.058226448
GOTERM BP DIRECT	GO:0000165~MAPK cascade	16	3.4557235	0.0029303	EGFR, GRB2, FGF9, BTC, IRS1, IL31RA, Z	427	262	16792	2.401558897	0.999726776	0.104955031	5.107791202
GOTERM BP DIRECT	GO:0090399~replicative senescence	4	0.8639309	0.0030072	WNT16, CDKN1A, TP53, CHEK2	427	12	16792	13.10850898	0.999779786	0.106204962	5.23848155
GOTERM BP DIRECT	GO:0030324~lung development	8	1.7278618	0.0031241	EGFR, HMGB1, NOTCH1, EP300, VEGFA,	427	76	16792	4.139529151	0.999841318	0.108734827	5.436694407
GOTERM BP DIRECT	GO:0007568~aging	12	2.5917927	0.0032987	VCAM1, DNMT3A, CYP1A1, GRB2, JUN,	427	165	16792	2.860038322	0.999902773	0.113061895	5.732216595
GOTERM BP DIRECT	GO:2001235~positive regulation of apoptotic signaling pathway	5	1.0799136	0.0033855	TPD52L1, PTEN, CASP2, RPS3, TP53INP1	427	25	16792	7.865105386	0.999923784	0.114464951	5.878752211
GOTERM BP DIRECT	GO:0070372~regulation of ERK1 and ERK2 cascade	5	1.0799136	0.0033855	EPHA7, TIAM1, FRS2, EPHA2, FN1	427	25	16792	7.865105386	0.999923784	0.114464951	5.878752211
GOTERM BP DIRECT	GO:0007569~cell aging	5	1.0799136	0.0033855	BCL2, NPM1, TP53, PDCD4, CITED2	427	25	16792	7.865105386	0.999923784	0.114464951	5.878752211
GOTERM BP DIRECT	GO:0006306~DNA methylation	5	1.0799136	0.0033855	ATRX, DNMT3A, EZH2, DNMT1, DNMT3I	427	25	16792	7.865105386	0.999923784	0.114464951	5.878752211
GOTERM BP DIRECT	GO:0007049~cell cycle	14	3.0237581	0.0037116	CCNT2, PARD6B, E2F3, TP53, ERBIN, PIN	427	217	16792	2.53713077	0.999969478	0.123315849	6.427513422
GOTERM BP DIRECT	GO:0051098~regulation of binding	3	0.6479482	0.0037243	SMAD4, SMAD3, SMAD2	427	4	16792	29.4941452	0.999970539	0.122260583	6.448658843
GOTERM BP DIRECT	GO:0032909~regulation of transforming growth factor beta2 production	3	0.6479482	0.0037243	SMAD4, SMAD3, TGFB2	427	4	16792	29.4941452	0.999970539	0.122260583	6.448658843
GOTERM BP DIRECT	GO:0051301~cell division	19	4.1036717	0.0037451	CCNT2, PARD6B, CENPS, CDK6, NR3C1,	427	350	16792	2.134814319	0.999972216	0.121482327	6.483691792
GOTERM BP DIRECT	GO:0006351~transcription, DNA-templated	69	14.902808	0.0041821	BMI1, CCNT2, MORF4L1, TRAPPC2B, E2I	427	1955	16792	1.387959774	0.999991852	0.133158233	7.213793069
GOTERM BP DIRECT	GO:0007264~small GTPase mediated signal transduction	15	3.2397408	0.0041996	PLD1, RAB3B, RAB8B, RAP2C, RAB5B, RI	427	246	16792	2.397897984	0.999992243	0.132178657	7.242932614
GOTERM BP DIRECT	GO:0032092~positive regulation of protein binding	7	1.511879	0.0044031	EP300, TIAM1, GSK3B, FLOT1, HFE, PKD	427	61	16792	4.512765386	0.999995618	0.136604408	7.580888857
GOTERM BP DIRECT	GO:0048538~thymus development	6	1.2958963	0.0044701	CRKL, BCL2, SRF, BCL2L11, CTNNB1, CI	427	43	16792	5.487282828	0.99999637	0.137024696	7.692000042
GOTERM BP DIRECT	GO:0033077~T cell differentiation in thymus	5	1.0799136	0.0045133	ZFP36L1, WNT1, BCL2, CDK6, CTNNB1	427	27	16792	7.282504987	0.999996785	0.1367624	7.763478209
GOTERM BP DIRECT	GO:0046777~protein autophosphorylation	12	2.5917927	0.0045189	EGFR, ACVR1B, IGF1R, PDPK1, FLT1, PAI	427	172	16792	2.743641414	0.999996835	0.135458581	7.772766778
GOTERM BP DIRECT	GO:0060065~uterus development	4	0.8639309	0.0047916	LHX1, SMAD4, HOXA9, TGFB2	427	14	16792	11.23586484	0.999998529	0.141532828	8.223082632
GOTERM BP DIRECT	GO:0006470~protein dephosphorylation	10	2.1598272	0.004837	PHLPP1, PTPRD, CDC14B, PPM1H, BCL2,	427	126	16792	3.121073566	0.999998705	0.141293437	8.297992269
GOTERM BP DIRECT	GO:2000145~regulation of cell motility	5	1.0799136	0.0051616	EGFR, ROCK2, BTC, RHOA, CDK6	427	28	16792	7.022415524	0.99999948	0.148512431	8.830901863
GOTERM BP DIRECT	GO:0002223~stimulatory C-type lectin receptor signaling pathway	9	1.9438445	0.0052601	PDPK1, KRAS, EP300, PAK2, PSMD11, PSI	427	105	16792	3.370759451	0.999999606	0.149596456	8.99194585
GOTERM BP DIRECT	GO:0030307~positive regulation of cell growth	8	1.7278618	0.0054496	EGFR, ADNP2, BCL2, AVPR1A, EXOSC2,	427	84	16792	3.745288279	0.999999768	0.153014742	9.301128151
GOTERM BP DIRECT	GO:0038095~Fc-epsilon receptor signaling pathway	12	2.5917927	0.0058285	PDPK1, KRAS, PAK2, PLCG1, PSMD11, GI	427	178	16792	2.651159119	0.99999992	0.1611666	9.916520633
GOTERM BP DIRECT	GO:0071549~cellular response to dexamethasone stimulus	5	1.0799136	0.0058696	EGFR, FOXO1, DNMT3B, ERRFI1, DDIT4	427	29	16792	6.780263264	0.999999929	0.16062918	9.983000694
GOTERM BP DIRECT	GO:0060412~ventricular septum morphogenesis	5	1.0799136	0.0058696	NOTCH1, SMAD4, ZFPM2, TGFB2, CITED	427	29	16792	6.780263264	0.999999929	0.16062918	9.983000694
GOTERM BP DIRECT	GO:0051891~positive regulation of cardioblast differentiation	3	0.6479482	0.006103	GATA6, GATA4, TGFB2	427	5	16792	23.59531616	0.999999963	0.164873421	10.35992011
GOTERM BP DIRECT	GO:0032534~regulation of microvillus assembly	3	0.6479482	0.006103	PLD1, PODXL, FSCN1	427	5	16792	23.59531616	0.999999963	0.164873421	10.35992011
GOTERM BP DIRECT	GO:0051781~positive regulation of cell division	6	1.2958963	0.0065598	FGF9, VEGFA, BTC, IGF2, MACC1, TGFB	427	47	16792	5.020280034	0.99999999	0.174431223	11.09303097
GOTERM BP DIRECT	GO:0008630~intrinsic apoptotic signaling pathway in response to DNA damage	6	1.2958963	0.0065598	MCL1, BCL2, CHEK2, CASP2, BCL2L11, E	427	47	16792	5.020280034	0.99999999	0.174431223	11.09303097
GOTERM BP DIRECT	GO:0032956~regulation of actin cytoskeleton organization	6	1.2958963	0.0065598	DLC1, ROCK2, FSCN1, RHOA, SH3BGRL	427	47	16792	5.020280034	0.99999999	0.174431223	11.09303097
GOTERM BP DIRECT	GO:0021915~neural tube development	5	1.0799136	0.0066393	ZFP36L1, NOTCH1, PKD1, TRIM71, EPHA	427	30	16792	6.554254489	0.999999992	0.174704588	11.22008164
GOTERM BP DIRECT	GO:2000379~positive regulation of reactive oxygen species metabolic process	5	1.0799136	0.0066393	CDKN1A, GRB2, MAPK14, TP53, THBS1	427	30	16792	6.554254489	0.999999992	0.174704588	11.22008164
GOTERM BP DIRECT	GO:0051895~negative regulation of focal adhesion assembly	4	0.8639309	0.0070997	DLC1, RCC2, THBS1, PTEN	427	16	16792	9.831381733	0.999999998	0.183950208	11.9522803
GOTERM BP DIRECT	GO:0030513~positive regulation of BMP signaling pathway	5	1.0799136	0.007473	NOTCH1, GATA6, GATA4, SMAD4, SMAI	427	31	16792	6.342826925	0.999999999	0.1909126	12.54189676
GOTERM BP DIRECT	GO:0035690~cellular response to drug	7	1.511879	0.0080307	EGFR, TFRC, ACER2, TP53, PPP1R12A, PI	427	69	16792	3.989546211	1	0.201837869	13.41562736
GOTERM BP DIRECT	GO:0016055~Wnt signaling pathway	12	2.5917927	0.0083336	CYLD, WNT1, WNT16, XIAP, CPE, GSK3E	427	187	16792	2.523563226	1	0.206789849	13.88682424
GOTERM BP DIRECT	GO:0045814~negative regulation of gene expression, epigenetic	6	1.2958963	0.0085189	SUZ12, BMI1, DNMT3A, EZH2, DNMT1, I	427	50	16792	4.719063232	1	0.209049187	14.17390428
GOTERM BP DIRECT	GO:0038092~nodal signaling pathway	3	0.6479482	0.0090014	ACVR1B, SMAD3, SMAD2	427	6	16792	19.66276347	1	0.217651296	14.91695795
GOTERM BP DIRECT	GO:2001244~positive regulation of intrinsic apoptotic signaling pathway	5	1.0799136	0.0093407	BCL2, TP53, PMAIP1, BCL2L11, PLAGL2	427	33	16792	5.958413172	1	0.222989333	15.43590929
GOTERM BP DIRECT	GO:0070059~intrinsic apoptotic signaling pathway in response to endoplasmic retic	5	1.0799136	0.0093407	TNFRSF10B, ERO1A, BCL2, GSK3B, PMA	427	33	16792	5.958413172	1	0.222989333	15.43590929
GOTERM BP DIRECT	GO:0016925~protein sumoylation	9	1.9438445	0.009915	BMI1, TP53BP1, INCENP, RNF2, NUP50, M	427	117	16792	3.025040533	1	0.233054703	16.30746314
GOTERM BP DIRECT	GO:0033189~response to vitamin A	4	0.8639309	0.0099645	DNMT3A, CYP1A1, GATA4, DNMT3B	427	18	16792	8.739005985	1	0.232146286	16.38222296
GOTERM BP DIRECT	GO:0006417~regulation of translation	6	1.2958963	0.0100296	ZFP36L1, LSM14A, RGS2, MKNK2, CASC	427	52	16792	4.5375608	1	0.231569579	16.48037967
GOTERM BP DIRECT	GO:0007067~mitotic nuclear division	14	3.0237581	0.0109659	YEATS4, CENPS, NR3C1, TTC28, HGF, RI	427	248	16792	2.219989424	1	0.248333874	17.88039393
GOTERM BP DIRECT	GO:0090263~positive regulation of canonical Wnt signaling pathway	9	1.9438445	0.0114491	WNT1, XIAP, PSMD11, FGF9, DLX5, PSMI	427	120	16792	2.94941452	1	0.255751246	18.59424972
GOTERM BP DIRECT	GO:0043536~positive regulation of blood vessel endothelial cell migration	4	0.8639309	0.0116136	PLCG1, MAPK14, VEGFA, THBS1	427	19	16792	8.279058301	1	0.256898738	18.83582016
GOTERM BP DIRECT	GO:0001525~angiogenesis	13	2.8077754	0.0117468	FMNL3, CYP1B1, FLT1, FGF9, PTEN, UBP	427	223	16792	2.292519507	1	0.257434096	19.0310603
GOTERM BP DIRECT	GO:1900034~regulation of cellular response to heat	7	1.511879	0.0118923	BAG4, EP300, BAG1, GSK3B, NUP50, NUP	427	75	16792	3.670382514	1	0.258189557	19.24375176
GOTERM BP DIRECT	GO:1903140~regulation of establishment of endothelial barrier	3	0.6479482	0.0123916	ROCK2, PPP1R12A, IKBKB	427	7	16792	16.85379726	1	0.265470182	19.96968675
GOTERM BP DIRECT	GO:0010614~negative regulation of cardiac muscle hypertrophy	3	0.6479482	0.0123916	RGS2, GSK3B, SMAD4	427	7	16792	16.85379726	1	0.265470182	19.96968675
GOTERM BP DIRECT	GO:2000144~positive regulation of DNA-templated transcription, initiation	3	0.6479482	0.0123916	JUN, MITF, CTNNB1	427	7	16792	16.85379726	1	0.265470182	19.96968675
GOTERM BP DIRECT	GO:0060021~palate development	7	1.511879	0.0126438	SATB2, DLX5, FOXF2, SMAD4, SMAD2, S	427	76	16792	3.622088007	1	0.268079798	20.33410425
GOTERM BP DIRECT	GO:0002052~positive regulation of neuroblast proliferation	4	0.8639309	0.0134101	NOTCH1, SMARCD3, VEGFA, CTNNB1	427	20	16792	7.865105386	1	0.279814516	21.4314933
GOTERM BP DIRECT	GO:0017015~regulation of transforming growth factor beta receptor signaling pathv	4	0.8639309	0.0134101	SMAD4, SMAD3, SMAD2, ZEB1	427	20	16792	7.865105386	1	0.279814516	21.4314933
GOTERM BP DIRECT	GO:1902895~positive regulation of pri-miRNA transcription from RNA polymeras	4	0.8639309	0.0134101	JUN, SMAD3, SRF, STAT3	427	20	16792	7.865105386	1	0.279814516	21.4314933
GOTERM BP DIRECT	GO:0032869~cellular response to insulin stimulus	7	1.511879	0.0134281	ZFP36L1, PDPK1, GOT1, SP1, FOXO1, ERI	427	77	16792	3.575047903	1	0.278091146	21.4570902
GOTERM BP DIRECT	GO:0070301~cellular response to hydrogen peroxide	6	1.2958963	0.0145869	CYP1B1, ETS1, EZH2, FOXO1, MAPK7, RI	427	57	16792	4.139529151	1	0.296126913	23.08904507
GOTERM BP DIRECT	GO:0000082~G1/S transition of mitotic cell cycle	8	1.7278618	0.015122	CCNE2, ACVR1B, CDKN1A, CUL5, CDKN	427	102	16792	3.084355053	1	0.303056949	23.83177427
GOTERM BP DIRECT	GO:0030522~intracellular receptor signaling pathway	5	1.0799136	0.0152641	DCBLD2, NOTCH2, NCOA2, THRB, STAT	427	38	16792	5.174411438	1	0.303305811	24.02792538
GOTERM BP DIRECT	GO:0050772~positive regulation of axonogenesis	4	0.8639309	0.0153559	SHOX2, TIAM1, RHOA, ARHGDIA	427	21	16792	7.490576558	1	0.302720544	24.15434584
GOTERM BP DIRECT	GO:0007492~endoderm development	4	0.8639309	0.0153559	NOTCH1, GATA4, SMAD4, SMAD3	427	21	16792	7.490576558	1	0.302720544	24.15434584
GOTERM BP DIRECT	GO:0002576~platelet degranulation	8	1.7278618	0.015883	A1BG, ACTN4, VEGFA, IGF2, HGF, THBS	427	103	16792	3.054409859	1	0.309239541	24.87644381
GOTERM BP DIRECT	GO:0042060~wound healing	7	1.511879	0.0159839	DCBLD2, EGFR, SLC11A1, SMAD3, LOX,	427	80	16792	3.440983607	1	0.308767071	25.01386339
GOTERM BP DIRECT	GO:0007183~SMAD protein complex assembly	3	0.6479482	0.0162469	SMAD4, SMAD3, SMAD2	427	8	16792	14.7470726	1	0.310888553	25.37109779
GOTERM BP DIRECT	GO:0035148~tube formation	3	0.6479482	0.0162469	NOTCH1, VEGFA, BCL2L11	427	8	16792	14.7470726	1	0.310888553	25.37109779
GOTERM BP DIRECT	GO:0033629~negative regulation of cell adhesion mediated by integrin	3	0.6479482	0.0162469	CYP1B1, ACER2, SNAI2	427	8	16792	14.7470726	1	0.310888553	25.37109779
GOTERM BP DIRECT	GO:0000079~regulation of cyclin-dependent protein serine/threonine kinase activity	5	1.0799136	0.0166767	CCNT2, CCNE2, CDKN1A, CDKN1B, PTEI	427	39	16792	5.041734222	1	0.315594202	25.95154371
GOTERM BP DIRECT	GO:0043406~positive regulation of MAP kinase activity	6	1.2958963	0.0167435	EGFR, KRAS, FLT1, VEGFA, EZH2, TPD52	427	59	16792	3.999206129	1	0.31455632	26.04128688
GOTERM BP DIRECT	GO:0071407~cellular response to organic cyclic compound	6	1.2958963	0.0167435	CDKN1B, CYP1B1, PAK2, CYP1A1, ARHG	427	59	16792	3.999206129	1	0.31455632	26.04128688
GOTERM BP DIRECT	GO:0051443~positive regulation of ubiquitin-protein transferase activity	4	0.8639309	0.0174524	BMI1, FBXW7, ARRDC3, PIN1	427	22	16792	7.150095806	1	0.323413694	26.98810721
GOTERM BP DIRECT	GO:0051091~positive regulation of sequence-specific DNA binding transcription fa	8	1.7278618	0.0174859	WNT1, EP300, TRIM8, TP53BP1, PHB2, PT	427	105	16792	2.996230623	1	0.321839103	27.03263561
GOTERM BP DIRECT	GO:0008543~fibroblast growth factor receptor signaling pathway	7	1.511879	0.0178633	GRB2, FGF9, HNRNPF, SHOC2, TRIM71, I	427	82	16792	3.357057177	1	0.325463248	27.53177424
GOTERM BP DIRECT	GO:0043433~negative regulation of sequence-specific DNA binding transcription fa	6	1.2958963	0.017897	FOXA2, PHB2, RNF2, EZH2, KEAP1, TCF7	427	60	16792	3.932552693	1	0.323904	27.57615715
GOTERM BP DIRECT	GO:0010508~positive regulation of autophagy	5	1.0799136	0.0181685	MTDH, TRIM8, FOXO1, PRKAA2, TP53INI	427	40	16792	4.915690867	1	0.325885716	27.93300331
GOTERM BP DIRECT	GO:0001947~heart looping	6	1.2958963	0.0191018	NOTCH1, GATA4, SMAD3, GJA1, SRF, CI	427	61	16792	3.868084616	1	0.337439909	29.14714859
GOTERM BP DIRECT	GO:0009880~embryonic pattern specification	4	0.8639309	0.0197002	SATB2, LHX1, SMAD3, SMAD2	427	23	16792	6.839222075	1	0.343906466	29.91543288
GOTERM BP DIRECT	GO:0003148~outflow tract septum morphogenesis	4	0.8639309	0.0197002	GATA6, SMAD4, ZFPM2, TGFB2	427	23	16792	6.839222075	1	0.343906466	29.91543288
GOTERM BP DIRECT	GO:0001836~release of cytochrome c from mitochondria	4	0.8639309	0.0197002	MFF, JUN, BCL2, PMAIP1	427	23	16792	6.839222075	1	0.343906466	29.91543288
GOTERM BP DIRECT	GO:2000008~regulation of protein localization to cell surface	3	0.6479482	0.0205416	BDNF, HFE, CTNNB1	427	9	16792	13.10850898	1	0.353598606	30.98236955
GOTERM BP DIRECT	GO:0002544~chronic inflammatory response	3	0.6479482	0.0205416	VCAM1, GJA1, THBS1	427	9	16792	13.10850898	1	0.353598606	30.98236955
GOTERM BP DIRECT	GO:0034405~response to fluid shear stress	3	0.6479482	0.0205416	PKD1, GJA1, CITED2	427	9	16792	13.10850898	1	0.353598606	30.98236955
GOTERM BP DIRECT	GO:2000737~negative regulation of stem cell differentiation	3	0.6479482	0.0205416	NOTCH1, YAP1, STAT3	427	9	16792	13.10850898	1	0.353598606	30.98236955
GOTERM BP DIRECT	GO:0097105~presynaptic membrane assembly	3	0.6479482	0.0205416	PTPRD, NLGN4X, PTEN	427	9	16792	13.10850898	1	0.353598606	30.98236955
GOTERM BP DIRECT	GO:0009636~response to toxic substance	7	1.511879	0.020958	DNMT3A, CDKN1A, CYP1B1, BCL2, GAB	427	85	16792	3.238572806	1	0.357219048	31.50471694
GOTERM BP DIRECT	GO:0097191~extrinsic apoptotic signaling pathway	5	1.0799136	0.021394	TNFRSF10A, ACVR1B, PDPK1, SMAD3, T	427	42	16792	4.681610349	1	0.361033109	32.0476115

GOTERM BP DIRECT	GO:0001658~branching involved in ureteric bud morphogenesis	5	1.0799136	0.021394	WNT1, LHX1, BCL2, SMAD4, CTNNB1	427	42	16792	4.681610349	1	0.361033109	32.0476115
GOTERM BP DIRECT	GO:0043123~positive regulation of I-kappaB kinase/NF-kappaB signaling	10	2.1598272	0.0217992	MTDH, TNFRSF10B, MAP3K3, TRIM8, TB	427	161	16792	2.442579313	1	0.364360591	32.54854546
GOTERM BP DIRECT	GO:0007010~cytoskeleton organization	10	2.1598272	0.0217992	YEATS4, FMNL3, BRWD1, WASF3, TUBB	427	161	16792	2.442579313	1	0.364360591	32.54854546
GOTERM BP DIRECT	GO:0006338~chromatin remodeling	7	1.511879	0.0220654	MORF4L1, ATRX, SATB2, SMARCD3, DEI	427	86	16792	3.200914983	1	0.365786841	32.87567871
GOTERM BP DIRECT	GO:0045648~positive regulation of erythrocyte differentiation	4	0.8639309	0.0221001	ACVR1B, ETS1, MAPK14, FOXO3	427	24	16792	6.554254489	1	0.364147858	32.91821221
GOTERM BP DIRECT	GO:0048870~cell motility	4	0.8639309	0.0221001	PLD1, ETS1, FSCN1, EPHA2	427	24	16792	6.554254489	1	0.364147858	32.91821221
GOTERM BP DIRECT	GO:0090090~negative regulation of canonical Wnt signaling pathway	10	2.1598272	0.0233918	CYLD, NOTCH1, PSMD11, MAPK14, GSK3	427	163	16792	2.412609014	1	0.378810789	34.48374551
GOTERM BP DIRECT	GO:0010629~negative regulation of gene expression	9	1.9438445	0.0236072	ACVR1B, CDKN1A, TBK1, CREB1, GJA1,	427	137	16792	2.583428777	1	0.37943474	34.74145165
GOTERM BP DIRECT	GO:2000134~negative regulation of G1/S transition of mitotic cell cycle	4	0.8639309	0.0246521	CDKN1A, BCL2, EZH2, PTEN	427	25	16792	6.292084309	1	0.390412106	35.97787714
GOTERM BP DIRECT	GO:0023014~signal transduction by protein phosphorylation	5	1.0799136	0.0249488	ACVR1B, PAK2, CAB39, KDR, TGFB2	427	44	16792	4.468809879	1	0.391937023	36.32491197
GOTERM BP DIRECT	GO:0051272~positive regulation of cellular component movement	3	0.6479482	0.0252512	ACTN4, ETS1, VEGFA	427	10	16792	11.79765808	1	0.393507313	36.6767853
GOTERM BP DIRECT	GO:0006983~ER overload response	3	0.6479482	0.0252512	GSK3B, TP53, PPP1R15B	427	10	16792	11.79765808	1	0.393507313	36.6767853
GOTERM BP DIRECT	GO:0035265~organ growth	3	0.6479482	0.0252512	BCL2, NLGN4X, SMAD2	427	10	16792	11.79765808	1	0.393507313	36.6767853
GOTERM BP DIRECT	GO:0045616~regulation of keratinocyte differentiation	3	0.6479482	0.0252512	ZFP36L1, ROCK2, ERRFI1	427	10	16792	11.79765808	1	0.393507313	36.6767853
GOTERM BP DIRECT	GO:0090009~primitive streak formation	3	0.6479482	0.0252512	FOXA2, LHX1, SRF	427	10	16792	11.79765808	1	0.393507313	36.6767853
GOTERM BP DIRECT	GO:0003184~pulmonary valve morphogenesis	3	0.6479482	0.0252512	NOTCH2, NOTCH1, TGFB2	427	10	16792	11.79765808	1	0.393507313	36.6767853
GOTERM BP DIRECT	GO:0051146~striated muscle cell differentiation	3	0.6479482	0.0252512	SPAG9, KRAS, MAPK14	427	10	16792	11.79765808	1	0.393507313	36.6767853
GOTERM BP DIRECT	GO:0031643~positive regulation of myelination	3	0.6479482	0.0252512	WASF3, DICER1, HGF	427	10	16792	11.79765808	1	0.393507313	36.6767853
GOTERM BP DIRECT	GO:0071363~cellular response to growth factor stimulus	5	1.0799136	0.0268516	PFN1, SHC1, MAPK7, THBS1, CTNNB1	427	45	16792	4.369502992	1	0.410509791	38.50881676
GOTERM BP DIRECT	GO:0032147~activation of protein kinase activity	5	1.0799136	0.0268516	SLC11A1, HMGB1, VEGFA, CAB39, TGFB	427	45	16792	4.369502992	1	0.410509791	38.50881676
GOTERM BP DIRECT	GO:0007160~cell-matrix adhesion	7	1.511879	0.0268879	VCAM1, TIAM1, FERMT2, PKD1, SRF, BC	427	90	16792	3.058652095	1	0.408781896	38.54970464
GOTERM BP DIRECT	GO:0002053~positive regulation of mesenchymal cell proliferation	4	0.8639309	0.027356	SHOX2, FGF9, VEGFA, CTNNB1	427	26	16792	6.050081066	1	0.412092418	39.07570498
GOTERM BP DIRECT	GO:0000186~activation of MAPKK activity	5	1.0799136	0.0288391	EGFR, CRKL, PLCG1, MAP3K3, FRS2	427	46	16792	4.274513797	1	0.426843033	40.71414912
GOTERM BP DIRECT	GO:0045165~cell fate commitment	5	1.0799136	0.0288391	WNT1, WNT16, GATA6, GATA4, SMAD2	427	46	16792	4.274513797	1	0.426843033	40.71414912
GOTERM BP DIRECT	GO:0010595~positive regulation of endothelial cell migration	5	1.0799136	0.0288391	ROCK2, ETS1, VEGFA, THBS1, KDR	427	46	16792	4.274513797	1	0.426843033	40.71414912
GOTERM BP DIRECT	GO:0006355~regulation of transcription, DNA-templated	51	11.015119	0.0289728	MORF4L1, ZBTB34, THRB, TAF1D, RBM1	427	1504	16792	1.333511884	1	0.426178845	40.85988572
GOTERM BP DIRECT	GO:0032728~positive regulation of interferon-beta production	4	0.8639309	0.0302114	POLR3F, HMGB1, TBK1, FLOT1	427	27	16792	5.82600399	1	0.437666024	42.19310906
GOTERM BP DIRECT	GO:0031069~hair follicle morphogenesis	4	0.8639309	0.0302114	NOTCH1, BCL2, CTNNB1, TGFB2	427	27	16792	5.82600399	1	0.437666024	42.19310906
GOTERM BP DIRECT	GO:0032495~response to muramyl dipeptide	3	0.6479482	0.0303518	NOTCH1, MAPK14, ERBIN	427	11	16792	10.72514371	1	0.437025395	42.34246192
GOTERM BP DIRECT	GO:0050927~positive regulation of positive chemotaxis	3	0.6479482	0.0303518	VEGFA, SMAD3, KDR	427	11	16792	10.72514371	1	0.437025395	42.34246192
GOTERM BP DIRECT	GO:0043547~positive regulation of GTPase activity	23	4.9676026	0.0313884	DLC1, EGFR, FGF9, GRB2, EZH2, BTC, PS	427	565	16792	1.600862158	1	0.445962026	43.43381735
GOTERM BP DIRECT	GO:0046854~phosphatidylinositol phosphorylation	7	1.511879	0.0323619	EGFR, GRB2, PIK3C2A, FGF9, BTC, IRS1,	427	94	16792	2.928496686	1	0.453998989	44.44096767
GOTERM BP DIRECT	GO:0035176~social behavior	5	1.0799136	0.0330696	EIF4EBP2, KRAS, NLGN4X, AVPR1A, PTE	427	48	16792	4.096409055	1	0.459114859	45.16238785
GOTERM BP DIRECT	GO:0090200~positive regulation of release of cytochrome c from mitochondria	4	0.8639309	0.0332173	MFF, TP53, PMAIP1, BCL2L11	427	28	16792	5.617932419	1	0.458456843	45.3119115
GOTERM BP DIRECT	GO:0001569~patterning of blood vessels	4	0.8639309	0.0332173	GNA13, VEGFA, SRF, CTNNB1	427	28	16792	5.617932419	1	0.458456843	45.3119115
GOTERM BP DIRECT	GO:0071260~cellular response to mechanical stimulus	6	1.2958963	0.0341294	TNFRSF10A, EGFR, TNFRSF10B, GJA1, C.	427	71	16792	3.323283966	1	0.465488128	46.22634813
GOTERM BP DIRECT	GO:0050852~T cell receptor signaling pathway	9	1.9438445	0.035145	PDPK1, PAK2, PLCG1, PSMD11, PSMD2, N	427	148	16792	2.391417178	1	0.47336246	47.22765005
GOTERM BP DIRECT	GO:0036092~phosphatidylinositol-3-phosphate biosynthetic process	5	1.0799136	0.0353134	GRB2, PIK3C2A, FGF9, IRS1, FRS2	427	49	16792	4.012808871	1	0.472848065	47.39193521
GOTERM BP DIRECT	GO:0061158~3'-UTR-mediated mRNA destabilization	3	0.6479482	0.0358207	ZFP36L1, QKI, TRIM71	427	12	16792	9.831381733	1	0.475611196	47.8839659
GOTERM BP DIRECT	GO:0048596~embryonic camera-type eye morphogenesis	3	0.6479482	0.0358207	FOXF2, ZEB1, CITED2	427	12	16792	9.831381733	1	0.475611196	47.8839659
GOTERM BP DIRECT	GO:0035278~miRNA mediated inhibition of translation	3	0.6479482	0.0358207	TRIM71, TNRC6A, STAT3	427	12	16792	9.831381733	1	0.475611196	47.8839659
GOTERM BP DIRECT	GO:0042149~cellular response to glucose starvation	4	0.8639309	0.0363728	BCL2, TP53, PRKAA2, PMAIP1	427	29	16792	5.424210611	1	0.478755	48.41459323
GOTERM BP DIRECT	GO:0007409~axonogenesis	7	1.511879	0.0385136	PARD6B, NOTCH1, CREB1, BCL2, GSK3B	427	98	16792	2.808966209	1	0.496579277	50.42412714
GOTERM BP DIRECT	GO:0001654~eye development	4	0.8639309	0.0396766	HMGB1, RAB18, FGF9, TGFB2	427	30	16792	5.243403591	1	0.504943542	51.48451714
GOTERM BP DIRECT	GO:0097193~intrinsic apoptotic signaling pathway	4	0.8639309	0.0396766	CDKN1A, CUL5, TP53, PMAIP1	427	30	16792	5.243403591	1	0.504943542	51.48451714
GOTERM BP DIRECT	GO:1900740~positive regulation of protein insertion into mitochondrial membrane	4	0.8639309	0.0396766	BCL2, TP53, PMAIP1, BCL2L11	427	30	16792	5.243403591	1	0.504943542	51.48451714
GOTERM BP DIRECT	GO:0042542~response to hydrogen peroxide	5	1.0799136	0.0400596	LDHA, JUN, BCL2, AREG, PPP1R15B	427	51	16792	3.855443817	1	0.506201823	51.82904749
GOTERM BP DIRECT	GO:0006367~transcription initiation from RNA polymerase II promoter	9	1.9438445	0.040148	NOTCH2, NOTCH1, E2F3, NRBP1, RARG,	427	152	16792	2.328485147	1	0.50484302	51.90830472
GOTERM BP DIRECT	GO:0046685~response to arsenic-containing substance	3	0.6479482	0.041636	CDKN1A, CYP1A1, PTEN	427	13	16792	9.0751216	1	0.515696833	53.22294391
GOTERM BP DIRECT	GO:0061029~eyelid development in camera-type eye	3	0.6479482	0.041636	EGFR, JUN, SRF	427	13	16792	9.0751216	1	0.515696833	53.22294391
GOTERM BP DIRECT	GO:0072657~protein localization to membrane	3	0.6479482	0.041636	CPE, TIAM1, FERMT2	427	13	16792	9.0751216	1	0.515696833	53.22294391
GOTERM BP DIRECT	GO:0097296~activation of cysteine-type endopeptidase activity involved in apoptot	3	0.6479482	0.041636	TNFRSF10A, TNFRSF10B, SMAD3	427	13	16792	9.0751216	1	0.515696833	53.22294391
GOTERM BP DIRECT	GO:0060413~atrial septum morphogenesis	3	0.6479482	0.041636	NOTCH2, GATA4, TGFB2	427	13	16792	9.0751216	1	0.515696833	53.22294391
GOTERM BP DIRECT	GO:0003198~epithelial to mesenchymal transition involved in endocardial cushion	3	0.6479482	0.041636	NOTCH1, SMAD4, SNAI2	427	13	16792	9.0751216	1	0.515696833	53.22294391
GOTERM BP DIRECT	GO:2000273~positive regulation of receptor activity	3	0.6479482	0.041636	NCOA2, HFE, IGF2	427	13	16792	9.0751216	1	0.515696833	53.22294391
GOTERM BP DIRECT	GO:0060317~cardiac epithelial to mesenchymal transition	3	0.6479482	0.041636	WNT16, NOTCH1, TGFB2	427	13	16792	9.0751216	1	0.515696833	53.22294391
GOTERM BP DIRECT	GO:0007623~circadian rhythm	6	1.2958963	0.0417351	EGFR, EP300, JUN, CREB1, GSK3B, CLOC	427	75	16792	3.146042155	1	0.514415259	53.30924148
GOTERM BP DIRECT	GO:0010468~regulation of gene expression	7	1.511879	0.0418503	ZFP36L1, BMI1, DNMT3A, LHX1, BCL2, C	427	100	16792	2.752786885	1	0.513284614	53.40947486
GOTERM BP DIRECT	GO:0034097~response to cytokine	5	1.0799136	0.0425621	MCL1, JUN, BCL2, CD274, SRF	427	52	16792	3.781300667	1	0.517225369	54.02391889
GOTERM BP DIRECT	GO:0045765~regulation of angiogenesis	4	0.8639309	0.0431269	ETS1, MAPK7, EPHA2, CTNNB1	427	31	16792	5.07426154	1	0.519867287	54.50612126
GOTERM BP DIRECT	GO:0007165~signal transduction	40	8.6393089	0.0447897	GNA13, DLC1, FGF9, GABRB1, GJA1, DEI	427	1161	16792	1.354884649	1	0.531456096	55.89796354
GOTERM BP DIRECT	GO:0040008~regulation of growth	5	1.0799136	0.0451508	MORF4L1, YEATS4, PAK2, ZMAT3, SHC1	427	53	16792	3.709955371	1	0.532279866	56.19489622
GOTERM BP DIRECT	GO:0001843~neural tube closure	6	1.2958963	0.0458918	DLC1, PFN1, RARG, TRIM71, TGFB2, CIT	427	77	16792	3.064326774	1	0.536122257	56.79821948
GOTERM BP DIRECT	GO:0042177~negative regulation of protein catabolic process	4	0.8639309	0.0467222	EGFR, SMAD3, MDM4, PIN1	427	32	16792	4.915690867	1	0.54059497	57.46507977
GOTERM BP DIRECT	GO:0046677~response to antibiotic	4	0.8639309	0.0467222	CYP1A1, ETS1, TP53, UQCRFS1	427	32	16792	4.915690867	1	0.54059497	57.46507977
GOTERM BP DIRECT	GO:0070830~bicellular tight junction assembly	4	0.8639309	0.0467222	PARD6B, MTDH, ACTN4, SRF	427	32	16792	4.915690867	1	0.54059497	57.46507977
GOTERM BP DIRECT	GO:0035115~embryonic forelimb morphogenesis	4	0.8639309	0.0467222	RECK, SHOX2, HOXA9, CTNNB1	427	32	16792	4.915690867	1	0.54059497	57.46507977
GOTERM BP DIRECT	GO:0007062~sister chromatid cohesion	7	1.511879	0.0471873	FBXW7, RCC2, CENPS, PHB2, INCENP, NU	427	103	16792	2.672608626	1	0.542150341	57.83432464
GOTERM BP DIRECT	GO:0003407~neural retina development	3	0.6479482	0.0477768	SMARCD3, CASP2, TGFB2	427	14	16792	8.426898628	1	0.544640736	58.29792654
GOTERM BP DIRECT	GO:1901224~positive regulation of NIK/NF-kappaB signaling	3	0.6479482	0.0477768	HMGB1, ACTN4, RPS3	427	14	16792	8.426898628	1	0.544640736	58.29792654
GOTERM BP DIRECT	GO:2000353~positive regulation of endothelial cell apoptotic process	3	0.6479482	0.0477768	FOXO3, THBS1, ANO6	427	14	16792	8.426898628	1	0.544640736	58.29792654
GOTERM BP DIRECT	GO:0031103~axon regeneration	3	0.6479482	0.0477768	JUN, BCL2, CHL1	427	14	16792	8.426898628	1	0.544640736	58.29792654
GOTERM BP DIRECT	GO:0043923~positive regulation by host of viral transcription	3	0.6479482	0.0477768	EP300, SP1, JUN	427	14	16792	8.426898628	1	0.544640736	58.29792654
GOTERM BP DIRECT	GO:0035729~cellular response to hepatocyte growth factor stimulus	3	0.6479482	0.0477768	CREB1, GSK3B, HGF	427	14	16792	8.426898628	1	0.544640736	58.29792654
GOTERM BP DIRECT	GO:0014066~regulation of phosphatidylinositol 3-kinase signaling	6	1.2958963	0.0480593	EGFR, GRB2, FGF9, BTC, IRS1, FRS2	427	78	16792	3.025040533	1	0.544752893	58.51844578
GOTERM BP DIRECT	GO:0001649~osteoblast differentiation	7	1.511879	0.0490557	SHOX2, FGF9, DLX5, GJA1, SNAI2, TMEM	427	104	16792	2.646910467	1	0.550257527	59.28730541
GOTERM BP DIRECT	GO:0007411~axon guidance	9	1.9438445	0.0500021	BDNF, KRAS, GRB2, CREB1, DLX5, SMAI	427	159	16792	2.225973223	1	0.555273454	60.00519212
GOTERM BP DIRECT	GO:0032916~positive regulation of transforming growth factor beta3 production	2	0.4319654	0.0500963	CREB1, SMAD3	427	2	16792	39.32552693	1	0.553939585	60.07597371
GOTERM BP DIRECT	GO:0048340~paraxial mesoderm morphogenesis	2	0.4319654	0.0500963	SMAD3, SMAD2	427	2	16792	39.32552693	1	0.553939585	60.07597371
GOTERM BP DIRECT	GO:1904761~negative regulation of myofibroblast differentiation	2	0.4319654	0.0500963	PDCD4, TP53INP1	427	2	16792	39.32552693	1	0.553939585	60.07597371
GOTERM BP DIRECT	GO:0046499~S-adenosylmethioninamine metabolic process	2	0.4319654	0.0500963	DNMT3A, DNMT3B	427	2	16792	39.32552693	1	0.553939585	60.07597371
GOTERM BP DIRECT	GO:0003169~coronary vein morphogenesis	2	0.4319654	0.0500963	NOTCH1, VEGFA	427	2	16792	39.32552693	1	0.553939585	60.07597371
GOTERM BP DIRECT	GO:0048145~regulation of fibroblast proliferation	2	0.4319654	0.0500963	CREB1, CTNNB1	427	2	16792	39.32552693	1	0.553939585	60.07597371
GOTERM BP DIRECT	GO:0044334~canonical Wnt signaling pathway involved in positive regulation of ej	2	0.4319654	0.0500963	TCF7L2, CTNNB1	427	2	16792	39.32552693	1	0.553939585	60.07597371
GOTERM BP DIRECT	GO:0036303~lymph vessel morphogenesis	2	0.4319654	0.0500963	VEGFA, PKD1	427	2	16792	39.32552693	1	0.553939585	60.07597371
GOTERM BP DIRECT	GO:0043491~protein kinase B signaling	4	0.8639309	0.0504603	ZFP36L1, SESN2, PTEN, EPHA2	427	33	16792	4.766730537	1	0.554597961	60.34843072
GOTERM BP DIRECT	GO:0010634~positive regulation of epithelial cell migration	4	0.8639309	0.0504603	PFN1, PLCG1, JUN, TGFB2	427	33	16792	4.766730537	1	0.554597961	60.34843072
GOTERM BP DIRECT	GO:0019882~antigen processing and presentation	5	1.0799136	0.0505866	RAB3B, RAB8B, RAB5B, ULBP2, HFE	427	55	16792	3.575047903	1	0.553515242	60.44250568
GOTERM BP DIRECT	GO:0001764~neuron migration	7	1.511879	0.050969	SATB2, GJA1, SRF, CHL1, CTNNB1, DDIT	427	105	16792	2.621701795	1	0.554303976	60.7261856
GOTERM BP DIRECT	GO:0043524~negative regulation of neuron apoptotic process	8	1.7278618	0.0512089	PDPK1, BDNF, KRAS, JUN, BCL2, CHL1, F	427	132	16792	2.383365269	1	0.554056027	60.9031787
GOTERM BP DIRECT	GO:0009952~anterior/posterior pattern specification	6	1.2958963	0.0525733	CRKL, RARG, LHX1, HOXB5, HOXA9, SM	427	80	16792	2.94941452	1	0.561824731	61.8955415
GOTERM BP DIRECT	GO:0031116~positive regulation of microtubule polymerization	3	0.6479482	0.0542227	CDKN1B, RGS2, RPS3	427	15	16792	7.865105386	1	0.571356685	63.06347771
GOTERM BP DIRECT	GO:0002042~cell migration involved in sprouting angiogenesis	3	0.6479482	0.0542227	VEGFA, SRF, KDR	427	15	16792	7.865105386	1	0.571356685	63.06347771
GOTERM BP DIRECT	GO:0048821~erythrocyte development	3	0.6479482	0.0542227	TMOD3, SRF, CITED2	427	15	16792	7.865105386	1	0.571356685	63.06347771
GOTERM BP DIRECT	GO:0045787~positive regulation of cell cycle	4	0.8639309	0.0543392	CDKN1B, BCL2L11, TGFB2, CITED2	427	34	16792	4.62653258	1	0.570190273	63.14467017
GOTERM BP DIRECT	GO:0043410~positive regulation of MAPK cascade	6	1.2958963	0.0549199	HMGB1, FLT1, FGF9, IGF2, KDR, CTNNB	427	81	16792	2.913001995	1	0.57220247	63.54685666
GOTERM BP DIRECT	GO:0071902~positive regulation of protein serine/threonine kinase activity	4	0.8639309	0.0583565	EZH2, RHOA, IGF2, CAB39	427	35	16792	4.494345935	1	0.593037488	65.84381335
GOTERM BP DIRECT	GO:0040018~positive regulation of multicellular organism growth	4	0.8639309	0.0583565	CREB1, BCL2, ATP8A2, CELF1	427	35	16792	4.494345935	1	0.593037488	65.84381335
GOTERM BP DIRECT	GO:0006978~DNA damage response, signal transduction by p53 class mediator res	3	0.6479482	0.0609546	CDKN1A, TP53, CHEK2	427	16	16792	7.3735363	1	0.60754886	67.48886204
GOTERM BP DIRECT	GO:0048566~embryonic digestive tract development	3	0.6479482	0.0609546	FGF9, FOXF2, TGFB2	427	16	16792	7.3735363	1	0.60754886	67.48886204
GOTERM BP DIRECT	GO:0030949~positive regulation of vascular endothelial growth factor receptor sign	3	0.6479482	0.0609546	FLT1, FGF9, VEGFA	427	16	16792	7.3735363	1	0.60754886	67.48886204
GOTERM BP DIRECT	GO:0006349~regulation of gene expression by genetic imprinting	3	0.6479482	0.0609546	DNMT3A, GSK3B, IGF2	427	16	16792	7.3735363	1	0.60754886	67.48886204
GOTERM BP DIRECT	GO:0032924~activin receptor signaling pathway	3	0.6479482	0.0609546	ACVR1B, SMAD3, SMAD2	427	16	16792	7.3735363	1	0.60754886	67.48886204
GOTERM BP DIRECT	GO:0071356~cellular response to tumor necrosis factor	7	1.511879	0.0612141	ZFP36L1, VCAM1, BAG4, CCL22, ERBIN,	427	110	16792	2.502533532	1	0.607210848	67.64896911
GOTERM BP DIRECT	GO:0051897~positive regulation of protein kinase B signaling	6	1.2958963	0.0623176	EGFR, BAG4, MTDH, IGF2, THBS1, TCF7I	427	84	16792	2.808966209	1	0.612047333	68.32165219
GOTERM BP DIRECT	GO:0009612~response to mechanical stimulus	5	1.0799136	0.0624825	ETS1, JUN, GATA4, THBS1, CITED2	427	59	16792	3.332671774	1	0.611121693	68.42102224
GOTERM BP DIRECT	GO:0010507~negative regulation of autophagy	4	0.8639309	0.0625097	MCL1, BCL2, GATA4, HGF	427	36	16792	4.369502992	1	0.609369465	68.43742427
GOTERM BP DIRECT	GO:0007017~microtubule-based process	4	0.8639309	0.0625097	TUBB, TUBB2A, TUBD1, TUBA1B	427	36	16792	4.369502992	1	0.609369465	68.43742427
GOTERM BP DIRECT	GO:0030198~extracellular matrix organization	10	2.1598272	0.0626499	RECK, VCAM1, ERO1A, FOXF2, LOX, AT2	427	196	16792	2.006404435	1	0.60831192	68.52165077
GOTERM BP DIRECT	GO:0019083~viral transcription	7	1.511879	0.0656299	NUP50, RPL5, RPL12, RPS4X, NUP43, RPS	427	112	16792	2.457845433	1	0.624069444	70.26242047
GOTERM BP DIRECT	GO:0050679~positive regulation of epithelial cell proliferation	5	1.0799136	0.0656671	EGFR, NOTCH1, FGF9, DLX5, VEGFA	427	60	16792	3.277127244	1	0.622394115	70.28355503
GOTERM BP DIRECT	GO:0045732~positive regulation of protein catabolic process	5	1.0799136	0.0656671	CDKN1B, GSK3B, FOXO1, GJA1, BARD1	427	60	16792	3.277127244	1	0.622394115	70.28355503
GOTERM BP DIRECT	GO:2001240~negative regulation of extrinsic apoptotic signaling pathway in absenc	4	0.8639309	0.0667962	MCL1, BCL2, MAPK7, SNAI2	427	37	16792	4.251408317	1	0.62700284	70.91861582
GOTERM BP DIRECT	GO:0048013~ephrin receptor signaling pathway	6	1.2958963	0.0675468	EPHA7, ARPC3, TIAM1, ROCK2, RHOA, E	427	86	16792	2.743641414	1	0.629387688	71.33368295
GOTERM BP DIRECT	GO:0042325~regulation of phosphorylation	3	0.6479482	0.0679538	IKBKB, RAD17, BARD1	427	17	16792	6.93979887	1	0.629814242	71.55639142
GOTERM BP DIRECT	GO:0030224~monocyte differentiation	3	0.6479482	0.0679538	JUN, VEGFA, IL31RA	427	17	16792	6.93979887	1	0.629814242	71.55639142
GOTERM BP DIRECT	GO:0048514~blood vessel morphogenesis	3	0.6479482	0.0679538	CYP1B1, FLT1, GJA1	427	17	16792	6.93979887	1	0.629814242	71.55639142
GOTERM BP DIRECT	GO:0009948~anterior/posterior axis specification	3	0.6479482	0.0679538	LHX1, RNF2, CTNNB1	427	17	16792	6.93979887	1	0.629814242	71.55639142
GOTERM BP DIRECT	GO:0032331~negative regulation of chondrocyte differentiation	3	0.6479482	0.0679538	RARG, SNAI2, CTNNB1	427	17	16792	6.93979887	1	0.629814242	71.55639142
GOTERM BP DIRECT	GO:0060749~mammary gland alveolus development	3	0.6479482	0.0679538	PHB2, VEGFA, AREG	427	17	16792	6.93979887	1	0.629814242	71.55639142
GOTERM BP DIRECT	GO:0007178~transmembrane receptor protein serine/threonine kinase signaling patl	3	0.6479482	0.0679538	ACVR1B, MAPK14, PPM1L	427	17	16792	6.93979887	1	0.629814242	71.55639142
GOTERM BP DIRECT	GO:0006950~response to stress	5	1.0799136	0.0689349	EGFR, NPM1, PRKAA2, ERRFI1, TP53INP1	427	61	16792	3.223403847	1	0.633425628	72.08656334
GOTERM BP DIRECT	GO:0018107~peptidyl-threonine phosphorylation	4	0.8639309	0.0712132	ACVR1B, PDPK1, BCL2, GSK3B	427	38	16792	4.139529151	1	0.643986958	73.28202294
GOTERM BP DIRECT	GO:0001568~blood vessel development	4	0.8639309	0.0712132	CRKL, MAP3K3, FOXO1, TCF7L2	427	38	16792	4.139529151	1	0.643986958	73.28202294
GOTERM BP DIRECT	GO:0060395~SMAD protein signal transduction	5	1.0799136	0.0722849	JUN, SMAD4, SMAD3, SMAD2, TGFB2	427	62	16792	3.171413462	1	0.64785416	73.82747618
GOTERM BP DIRECT	GO:0007219~Notch signaling pathway	7	1.511879	0.0725944	NOTCH2, NOTCH1, GOT1, CDKN1B, EP30	427	115	16792	2.393727726	1	0.647661014	73.98303209
GOTERM BP DIRECT	GO:0006468~protein phosphorylation	18	3.887689	0.0731895	CCNT2, NRBP1, ROCK2, CREB1, PDK4, M	427	456	16792	1.552323432	1	0.648962834	74.27966088
GOTERM BP DIRECT	GO:2001027~negative regulation of endothelial cell chemotaxis	2	0.4319654	0.0741975	NOTCH1, THBS1	427	3	16792	26.21701795	1	0.652380315	74.7748675
GOTERM BP DIRECT	GO:0044336~canonical Wnt signaling pathway involved in negative regulation of a	2	0.4319654	0.0741975	WNT1, CTNNB1	427	3	16792	26.21701795	1	0.652380315	74.7748675
GOTERM BP DIRECT	GO:0060947~cardiac vascular smooth muscle cell differentiation	2	0.4319654	0.0741975	GATA6, SRF	427	3	16792	26.21701795	1	0.652380315	74.7748675
GOTERM BP DIRECT	GO:0010424~DNA methylation on cytosine within a CG sequence	2	0.4319654	0.0741975	DNMT3A, DNMT3B	427	3	16792	26.21701795	1	0.652380315	74.7748675
GOTERM BP DIRECT	GO:0060948~cardiac vascular smooth muscle cell development	2	0.4319654	0.0741975	NOTCH1, VEGFA	427	3	16792	26.21701795	1	0.652380315	74.7748675
GOTERM BP DIRECT	GO:0060066~oviduct development	2	0.4319654	0.0741975	LHX1, CTNNB1	427	3	16792	26.21701795	1	0.652380315	74.7748675
GOTERM BP DIRECT	GO:0044027~hypermethylation of CpG island	2	0.4319654	0.0741975	DNMT3A, GSK3B	427	3	16792	26.21701795	1	0.652380315	74.7748675
GOTERM BP DIRECT	GO:1904888~cranial skeletal system development	2	0.4319654	0.0741975	CTNNB1, TGFB2	427	3	16792	26.21701795	1	0.652380315	74.7748675
GOTERM BP DIRECT	GO:0090526~regulation of gluconeogenesis involved in cellular glucose homeostas	2	0.4319654	0.0741975	OGT, SESN2	427	3	16792	26.21701795	1	0.652380315	74.7748675
GOTERM BP DIRECT	GO:1902263~apoptotic process involved in embryonic digit morphogenesis	2	0.4319654	0.0741975	NOTCH1, BCL2L11	427	3	16792	26.21701795	1	0.652380315	74.7748675
GOTERM BP DIRECT	GO:1903378~positive regulation of oxidative stress-induced neuron intrinsic apopto	2	0.4319654	0.0741975	FBXW7, MCL1	427	3	16792	26.21701795	1	0.652380315	74.7748675
GOTERM BP DIRECT	GO:0010909~positive regulation of heparan sulfate proteoglycan biosynthetic proce	2	0.4319654	0.0741975	TCF7L2, CTNNB1	427	3	16792	26.21701795	1	0.652380315	74.7748675
GOTERM BP DIRECT	GO:0043161~proteasome-mediated ubiquitin-dependent protein catabolic process	10	2.1598272	0.0744439	C18ORF25, PSMD11, GSK3B, TP53, PSMD	427	203	16792	1.937218075	1	0.651850251	74.89454785
GOTERM BP DIRECT	GO:0008053~mitochondrial fusion	3	0.6479482	0.0752024	MFF, GDAP1, MIGA1	427	18	16792	6.554254489	1	0.65393493	75.2595935
GOTERM BP DIRECT	GO:0045216~cell-cell junction organization	3	0.6479482	0.0752024	NLGN4X, SMAD3, TGFB2	427	18	16792	6.554254489	1	0.65393493	75.2595935
GOTERM BP DIRECT	GO:0001708~cell fate specification	3	0.6479482	0.0752024	NOTCH1, FOXA2, CTNNB1	427	18	16792	6.554254489	1	0.65393493	75.2595935
GOTERM BP DIRECT	GO:0007250~activation of NF-kappaB-inducing kinase activity	3	0.6479482	0.0752024	TNFRSF10A, TNFRSF10B, MALT1	427	18	16792	6.554254489	1	0.65393493	75.2595935
GOTERM BP DIRECT	GO:0030705~cytoskeleton-dependent intracellular transport	3	0.6479482	0.0752024	KIF13A, TUBB, TUBA1B	427	18	16792	6.554254489	1	0.65393493	75.2595935
GOTERM BP DIRECT	GO:0009611~response to wounding	5	1.0799136	0.0757166	ZFP36L1, WNT1, CYP1A1, FN1, TGFB2	427	63	16792	3.121073566	1	0.654759292	75.50416566
GOTERM BP DIRECT	GO:0045668~negative regulation of osteoblast differentiation	4	0.8639309	0.0757579	NOTCH1, SMAD3, CDK6, AREG	427	39	16792	4.033387378	1	0.653198327	75.52375191
GOTERM BP DIRECT	GO:0030199~collagen fibril organization	4	0.8639309	0.0757579	CYP1B1, LOX, SERPINH1, TGFB2	427	39	16792	4.033387378	1	0.653198327	75.52375191
GOTERM BP DIRECT	GO:0070374~positive regulation of ERK1 and ERK2 cascade	9	1.9438445	0.0780131	EGFR, HMGB1, FBXW7, CCL22, PHB2, JU	427	175	16792	2.022455671	1	0.662644431	76.56905582
GOTERM BP DIRECT	GO:0030512~negative regulation of transforming growth factor beta receptor signal	5	1.0799136	0.0792289	WNT1, PDPK1, SMAD3, SMAD2, PIN1	427	64	16792	3.072306792	1	0.666799702	77.11494142
GOTERM BP DIRECT	GO:0034599~cellular response to oxidative stress	5	1.0799136	0.0792289	ADNP2, FOXO1, FOXO3, SESN2, ETV5	427	64	16792	3.072306792	1	0.666799702	77.11494142
GOTERM BP DIRECT	GO:0031398~positive regulation of protein ubiquitination	5	1.0799136	0.0792289	FBXW7, XIAP, CENPS, MALT1, UBE2D1	427	64	16792	3.072306792	1	0.666799702	77.11494142
GOTERM BP DIRECT	GO:0033209~tumor necrosis factor-mediated signaling pathway	7	1.511879	0.0799662	TNFRSF10A, BAG4, TNFRSF10B, PSMD11	427	118	16792	2.332870242	1	0.668596525	77.44014919
GOTERM BP DIRECT	GO:0000184~nuclear-transcribed mRNA catabolic process, nonsense-mediated deca	7	1.511879	0.0825135	RPL5, CASC3, RPL12, PABPC1, RPS4X, RF	427	119	16792	2.31326629	1	0.678826759	78.53035796
GOTERM BP DIRECT	GO:0046827~positive regulation of protein export from nucleus	3	0.6479482	0.0826835	GSK3B, TP53, TCF7L2	427	19	16792	6.209293726	1	0.677894121	78.60132238
GOTERM BP DIRECT	GO:0007520~myoblast fusion	3	0.6479482	0.0826835	WNT1, GSK3B, ADAM12	427	19	16792	6.209293726	1	0.677894121	78.60132238
GOTERM BP DIRECT	GO:0001659~temperature homeostasis	3	0.6479482	0.0826835	FOXO1, ARRDC3, STAT3	427	19	16792	6.209293726	1	0.677894121	78.60132238
GOTERM BP DIRECT	GO:0050771~negative regulation of axonogenesis	3	0.6479482	0.0826835	RHOA, PTEN, ARHGDIA	427	19	16792	6.209293726	1	0.677894121	78.60132238
GOTERM BP DIRECT	GO:0014068~positive regulation of phosphatidylinositol 3-kinase signaling	5	1.0799136	0.082821	WNT16, FLT1, HGF, KDR, TGFB2	427	65	16792	3.025040533	1	0.676817882	78.65854085
GOTERM BP DIRECT	GO:0051384~response to glucocorticoid	5	1.0799136	0.082821	HMGB1, GOT1, KRAS, BCL2, AREG	427	65	16792	3.025040533	1	0.676817882	78.65854085
GOTERM BP DIRECT	GO:0060071~Wnt signaling pathway, planar cell polarity pathway	6	1.2958963	0.0846442	PFN1, WNT1, TIAM1, PSMD11, RHOA, PS	427	92	16792	2.564708278	1	0.683414824	79.40389668
GOTERM BP DIRECT	GO:0009887~organ morphogenesis	6	1.2958963	0.0846442	NOTCH2, CRKL, EP300, THRB, LHX1, GS	427	92	16792	2.564708278	1	0.683414824	79.40389668
GOTERM BP DIRECT	GO:0000278~mitotic cell cycle	4	0.8639309	0.0852184	DNMT3A, PAK2, RNF2, MYB	427	41	16792	3.836636774	1	0.684299449	79.63351244
GOTERM BP DIRECT	GO:0045599~negative regulation of fat cell differentiation	4	0.8639309	0.090128	WNT1, SMAD3, FOXO1, ZFPM2	427	42	16792	3.745288279	1	0.703878658	81.50038971
GOTERM BP DIRECT	GO:0051496~positive regulation of stress fiber assembly	4	0.8639309	0.090128	PFN1, BAG4, RHOA, SMAD3	427	42	16792	3.745288279	1	0.703878658	81.50038971

GOTERM BP DIRECT	GO:0008361~regulation of cell size	3	0.6479482	0.0903805	RARG, CREB1, MSN	427	20	16792	5.89882904	1	0.703279228	81.59190734
GOTERM BP DIRECT	GO:0001892~embryonic placenta development	3	0.6479482	0.0903805	EGFR, PKD1, CITED2	427	20	16792	5.89882904	1	0.703279228	81.59190734
GOTERM BP DIRECT	GO:0050900~leukocyte migration	7	1.511879	0.0904229	KRAS, PLCG1, GRB2, PODXL, SHC1, MSN	427	122	16792	2.256382693	1	0.701805994	81.60722802
GOTERM BP DIRECT	GO:0018108~peptidyl-tyrosine phosphorylation	8	1.7278618	0.095068	EGFR, EPHA7, FLT1, FGF9, BTC, ABI2, EF	427	153	16792	2.056236702	1	0.719050631	83.21497142
GOTERM BP DIRECT	GO:1904837~beta-catenin-TCF complex assembly	4	0.8639309	0.0951529	EP300, TCF7L2, TCF7L1, CTNNB1	427	43	16792	3.658188552	1	0.717767028	83.24306628
GOTERM BP DIRECT	GO:0030155~regulation of cell adhesion	4	0.8639309	0.0951529	ROCK2, PPP1R12A, PKD1, SRF	427	43	16792	3.658188552	1	0.717767028	83.24306628
GOTERM BP DIRECT	GO:0051726~regulation of cell cycle	7	1.511879	0.0959169	CCNE2, EP300, JUN, BAP1, USP16, PTEN,	427	124	16792	2.219989424	1	0.719158105	83.49406179
GOTERM BP DIRECT	GO:0007169~transmembrane receptor protein tyrosine kinase signaling pathway	6	1.2958963	0.0971905	EGFR, IGF1R, MTSS1, FLT1, KDR, IL31RA	427	96	16792	2.457845433	1	0.722502511	83.90457031
GOTERM BP DIRECT	GO:0007621~negative regulation of female receptivity	2	0.4319654	0.0976886	THRB, AVPR1A	427	4	16792	19.66276347	1	0.72282582	84.06248713
GOTERM BP DIRECT	GO:0003219~cardiac right ventricle formation	2	0.4319654	0.0976886	NOTCH1, SMARCD3	427	4	16792	19.66276347	1	0.72282582	84.06248713
GOTERM BP DIRECT	GO:1901031~regulation of response to reactive oxygen species	2	0.4319654	0.0976886	SESN2, SESN1	427	4	16792	19.66276347	1	0.72282582	84.06248713
GOTERM BP DIRECT	GO:0046498~S-adenosylhomocysteine metabolic process	2	0.4319654	0.0976886	DNMT3A, DNMT3B	427	4	16792	19.66276347	1	0.72282582	84.06248713
GOTERM BP DIRECT	GO:0060534~trachea cartilage development	2	0.4319654	0.0976886	RARG, SRF	427	4	16792	19.66276347	1	0.72282582	84.06248713
GOTERM BP DIRECT	GO:0060429~epithelium development	2	0.4319654	0.0976886	LHX1, SNAI2	427	4	16792	19.66276347	1	0.72282582	84.06248713
GOTERM BP DIRECT	GO:0072177~mesonephric duct development	2	0.4319654	0.0976886	LHX1, PKD1	427	4	16792	19.66276347	1	0.72282582	84.06248713
GOTERM BP DIRECT	GO:0035522~monoubiquitinated histone H2A deubiquitination	2	0.4319654	0.0976886	BAP1, USP16	427	4	16792	19.66276347	1	0.72282582	84.06248713
GOTERM BP DIRECT	GO:0090403~oxidative stress-induced premature senescence	2	0.4319654	0.0976886	WNT16, TP53	427	4	16792	19.66276347	1	0.72282582	84.06248713
GOTERM BP DIRECT	GO:1904954~canonical Wnt signaling pathway involved in midbrain dopaminergic	2	0.4319654	0.0976886	WNT1, CTNNB1	427	4	16792	19.66276347	1	0.72282582	84.06248713
GOTERM BP DIRECT	GO:0061419~positive regulation of transcription from RNA polymerase II promote	2	0.4319654	0.0976886	NOTCH1, VEGFA	427	4	16792	19.66276347	1	0.72282582	84.06248713
GOTERM BP DIRECT	GO:1903347~negative regulation of bicellular tight junction assembly	2	0.4319654	0.0976886	ROCK2, IKBKB	427	4	16792	19.66276347	1	0.72282582	84.06248713
GOTERM BP DIRECT	GO:0007161~calcium-independent cell-matrix adhesion	2	0.4319654	0.0976886	PKD1, FN1	427	4	16792	19.66276347	1	0.72282582	84.06248713
GOTERM BP DIRECT	GO:0002520~immune system development	2	0.4319654	0.0976886	TROVE2, SMAD3	427	4	16792	19.66276347	1	0.72282582	84.06248713
GOTERM BP DIRECT	GO:0090400~stress-induced premature senescence	2	0.4319654	0.0976886	CDKN1A, MAPK14	427	4	16792	19.66276347	1	0.72282582	84.06248713
GOTERM BP DIRECT	GO:0003273~cell migration involved in endocardial cushion formation	2	0.4319654	0.0976886	NOTCH1, SNAI2	427	4	16792	19.66276347	1	0.72282582	84.06248713
GOTERM BP DIRECT	GO:0043154~negative regulation of cysteine-type endopeptidase activity involved i	5	1.0799136	0.0979668	XIAP, VEGFA, HGF, THBS1, DNAJB6	427	69	16792	2.849675865	1	0.722306814	84.15004748
GOTERM BP DIRECT	GO:0031954~positive regulation of protein autophosphorylation	3	0.6479482	0.0982779	RAP2C, VEGFA, RASSF2	427	21	16792	5.617932419	1	0.721917161	84.24741176
GOTERM BP DIRECT	GO:0097150~neuronal stem cell population maintenance	3	0.6479482	0.0982779	NOTCH1, FOXO1, FOXO3	427	21	16792	5.617932419	1	0.721917161	84.24741176
GOTERM BP DIRECT	GO:0031016~pancreas development	3	0.6479482	0.0982779	SMAD2, TCF7L2, CTNNB1	427	21	16792	5.617932419	1	0.721917161	84.24741176
GOTERM BP DIRECT	GO:0071480~cellular response to gamma radiation	3	0.6479482	0.0982779	CDKN1A, CHEK2, YAP1	427	21	16792	5.617932419	1	0.721917161	84.24741176
GOTERM BP DIRECT	GO:1900182~positive regulation of protein localization to nucleus	3	0.6479482	0.0982779	TRIM8, SESN2, TGFB2	427	21	16792	5.617932419	1	0.721917161	84.24741176
GOTERM BP DIRECT	GO:0051894~positive regulation of focal adhesion assembly	3	0.6479482	0.0982779	VEGFA, SMAD3, KDR	427	21	16792	5.617932419	1	0.721917161	84.24741176
GOTERM BP DIRECT	GO:0042127~regulation of cell proliferation	9	1.9438445	0.0994809	TNFRSF10A, CDKN1B, TNFRSF10B, XIAP	427	185	16792	1.913133743	1	0.724905829	84.61867705

Category	Term	Count	%	PValue	Genes	List Total	Pop Hits	Pop Total	Fold Enricl	Bonferroni	Benjamini	FDR
GOTERM CC DIRECT	GO:0005654~nucleoplasm	134	28.94168	1.16E-15	MORF4L1, NRBP1, THRB, RBM	443	2784	18224	1.980047	4.83E-13	4.83E-13	1.57E-12
GOTERM CC DIRECT	GO:0005634~nucleus	206	44.49244	5.80E-14	DLC1, LDHA, TRAPPC2B, TUB	443	5415	18224	1.56498	2.52E-11	1.26E-11	8.18E-11
GOTERM CC DIRECT	GO:0005829~cytosol	140	30.23758	6.72E-12	DLC1, LDHA, NRBP1, TBK1, FC	443	3315	18224	1.737339	2.92E-09	9.74E-10	9.48E-09
GOTERM CC DIRECT	GO:0005737~cytoplasm	192	41.46868	2.84E-11	GNA13, DLC1, MTSS1, LDHA,	443	5222	18224	1.512531	1.23E-08	3.08E-09	4.00E-08
GOTERM CC DIRECT	GO:0030529~intracellular ribonucleoprotein complex	17	3.671706	1.99E-07	SNRPA1, ACTN4, G3BP2, CASC	443	136	18224	5.142212	8.67E-05	1.73E-05	2.81E-04
GOTERM CC DIRECT	GO:0005925~focal adhesion	29	6.263499	3.59E-07	GNA13, DLC1, FERMT2, GJA1,	443	391	18224	3.051134	1.56E-04	2.60E-05	5.07E-04
GOTERM CC DIRECT	GO:0005768~endosome	21	4.535637	6.35E-07	EGFR, HMGB1, PLD1, FLT1, RA	443	225	18224	3.839518	2.76E-04	3.94E-05	8.96E-04
GOTERM CC DIRECT	GO:0000790~nuclear chromatin	18	3.887689	4.97E-06	RARG, THRB, CREB1, EZH2, TI	443	193	18224	3.836676	0.002161	2.70E-04	0.007021
GOTERM CC DIRECT	GO:0005667~transcription factor complex	18	3.887689	4.97E-06	SATB2, E2F3, RARG, SMAD4, S	443	193	18224	3.836676	0.002161	2.70E-04	0.007021
GOTERM CC DIRECT	GO:0030027~lamellipodium	15	3.239741	3.65E-05	NRBP1, ARPC3, PLCG1, WASF3	443	160	18224	3.856659	0.015737	0.001761	0.051468
GOTERM CC DIRECT	GO:0048471~perinuclear region of cytoplasm	31	6.695464	2.85E-04	CCNT2, TRAPPC2B, RAB3B, NI	443	621	18224	2.053573	0.116669	0.012329	0.401819
GOTERM CC DIRECT	GO:0031093~platelet alpha granule lumen	8	1.727862	3.50E-04	A1BG, ACTN4, VEGFA, IGF2, H	443	55	18224	5.983665	0.14112	0.013734	0.492518
GOTERM CC DIRECT	GO:0016605~PML body	10	2.159827	6.26E-04	ATRX, TRIM8, MKNK2, TP53, M	443	98	18224	4.197724	0.238422	0.022441	0.880077
GOTERM CC DIRECT	GO:0005730~nucleolus	37	7.991361	9.99E-04	CDV3, MTDH, CDC14B, GRB2,	443	857	18224	1.776073	0.352678	0.032901	1.401619
GOTERM CC DIRECT	GO:0030496~midbody	11	2.37581	0.0012	KIF13A, CYLD, RCC2, INCENP,	443	129	18224	3.507866	0.406948	0.036632	1.681429
GOTERM CC DIRECT	GO:0016363~nuclear matrix	9	1.943844	0.002472	XPOT, YEATS4, DNMT3A, SAT	443	97	18224	3.8169	0.659298	0.069267	3.434298
GOTERM CC DIRECT	GO:0043234~protein complex	21	4.535637	0.002688	PARD6B, ACTN4, MITF, TP53,	443	412	18224	2.096824	0.689944	0.070574	3.729257
GOTERM CC DIRECT	GO:0032993~protein-DNA complex	5	1.079914	0.002882	SUZ12, SP1, KDM5A, TCF7L2, C	443	25	18224	8.22754	0.71511	0.0712	3.993383
GOTERM CC DIRECT	GO:0005741~mitochondrial outer membrane	11	2.37581	0.003481	MFF, CISD2, MCL1, PHB2, BCL	443	149	18224	3.037011	0.78058	0.080812	4.803569
GOTERM CC DIRECT	GO:0016020~membrane	72	15.55076	0.007142	GNA13, LDHA, NRBP1, RAB5B	443	2200	18224	1.346325	0.955752	0.151344	9.624306
GOTERM CC DIRECT	GO:0043292~contractile fiber	3	0.647948	0.011369	PPP1R12A, GJA1, DEK	443	7	18224	17.63044	0.993083	0.220179	14.90726
GOTERM CC DIRECT	GO:0016324~apical plasma membrane	15	3.239741	0.01186	EGFR, PARD6B, PLD1, MTDH,	443	291	18224	2.1205	0.994428	0.218971	15.50227
GOTERM CC DIRECT	GO:0009986~cell surface	23	4.967603	0.013339	EGFR, DCBLD2, HMGB1, FERM	443	542	18224	1.745696	0.997096	0.233196	17.27029
GOTERM CC DIRECT	GO:0071141~SMAD protein complex	3	0.647948	0.014917	SMAD4, SMAD3, SMAD2	443	8	18224	15.42664	0.998552	0.247423	19.11877
GOTERM CC DIRECT	GO:0015629~actin cytoskeleton	12	2.591793	0.017612	IPP, MTSS1, ACTN4, ARPC3, ZN	443	218	18224	2.26446	0.99956	0.275351	22.18766
GOTERM CC DIRECT	GO:0009898~cytoplasmic side of plasma membrane	5	1.079914	0.01848	RGS2, PTPN4, IKBKB, PTEN, R	443	42	18224	4.897345	0.999701	0.277154	23.15231
GOTERM CC DIRECT	GO:0036464~cytoplasmic ribonucleoprotein granule	4	0.863931	0.021938	TUBB, ROCK2, PABPC1, RPS4X	443	25	18224	6.582032	0.999936	0.310047	26.88807
GOTERM CC DIRECT	GO:0005739~mitochondrion	45	9.719222	0.022422	APOOL, CYP1B1, MCL1, CLPB,	443	1331	18224	1.390831	0.999948	0.306053	27.39715
GOTERM CC DIRECT	GO:0070062~extracellular exosome	85	18.35853	0.022537	GNA13, LDHA, CADM1, WASF	443	2811	18224	1.243936	0.999951	0.298224	27.5178
GOTERM CC DIRECT	GO:0005916~fascia adherens	3	0.647948	0.023218	GJA1, CTNNA3, CTNNB1	443	10	18224	12.34131	0.999964	0.296986	28.22717
GOTERM CC DIRECT	GO:0031234~extrinsic component of cytoplasmic side of plasma me	6	1.295896	0.024551	CYLD, KRAS, TIAM1, FERMT2,	443	68	18224	3.629797	0.99998	0.30263	29.59803
GOTERM CC DIRECT	GO:0005793~endoplasmic reticulum-Golgi intermediate compartme	6	1.295896	0.024551	HMGB1, TRAPPC2B, DICER1, S	443	68	18224	3.629797	0.99998	0.30263	29.59803
GOTERM CC DIRECT	GO:0005911~cell-cell junction	10	2.159827	0.024683	CADM1, ACTN4, PLCG1, TIAM	443	172	18224	2.391727	0.999981	0.295809	29.73208
GOTERM CC DIRECT	GO:0005719~nuclear euchromatin	4	0.863931	0.026928	JUN, CREB1, H2AFZ, CTNNB1	443	27	18224	6.094474	0.999993	0.310008	31.98163
GOTERM CC DIRECT	GO:0005819~spindle	8	1.727862	0.0281	CYLD, EVI5, INCENP, KLHL42,	443	121	18224	2.719848	0.999996	0.313203	33.12914
GOTERM CC DIRECT	GO:0045177~apical part of cell	6	1.295896	0.0355	VCAM1, PARD6B, HFE, MSN, C	443	75	18224	3.291016	1	0.370263	39.96928
GOTERM CC DIRECT	GO:0005856~cytoskeleton	16	3.455724	0.037701	PPP1R18, WASF3, FERMT2, PT	443	371	18224	1.774133	1	0.379748	41.87434
GOTERM CC DIRECT	GO:0000164~protein phosphatase type 1 complex	3	0.647948	0.038367	PPP1R11, SHOC2, PPP1R15B	443	13	18224	9.493315	1	0.376699	42.43958
GOTERM CC DIRECT	GO:0005721~pericentric heterochromatin	3	0.647948	0.038367	ATRX, INCENP, DNMT1	443	13	18224	9.493315	1	0.376699	42.43958
GOTERM CC DIRECT	GO:0001725~stress fiber	5	1.079914	0.041722	ACTN4, FSCN1, FERMT2, SEPT	443	54	18224	3.809046	1	0.394101	45.21107
GOTERM CC DIRECT	GO:0090575~RNA polymerase II transcription factor complex	4	0.863931	0.045189	GATA4, SMAD4, STAT3, HNRN	443	33	18224	4.986388	1	0.411008	47.94389
GOTERM CC DIRECT	GO:0070435~Shc-EGFR complex	2	0.431965	0.047921	EGFR, SHC1	443	2	18224	41.1377	1	0.421739	50.00768
GOTERM CC DIRECT	GO:0071144~SMAD2-SMAD3 protein complex	2	0.431965	0.047921	SMAD3, SMAD2	443	2	18224	41.1377	1	0.421739	50.00768
GOTERM CC DIRECT	GO:0070369~beta-catenin-TCF7L2 complex	2	0.431965	0.047921	TCF7L2, CTNNB1	443	2	18224	41.1377	1	0.421739	50.00768
GOTERM CC DIRECT	GO:0005802~trans-Golgi network	8	1.727862	0.048026	RAB30, PLEKHA8, STX16, ATP	443	136	18224	2.419865	1	0.414471	50.08571
GOTERM CC DIRECT	GO:0000307~cyclin-dependent protein kinase holoenzyme complex	3	0.647948	0.050036	CDKN1A, CCND2, CDK6	443	15	18224	8.22754	1	0.419936	51.55363
GOTERM CC DIRECT	GO:0005913~cell-cell adherens junction	14	3.023758	0.051936	EGFR, LDHA, CADM1, FSCN1,	443	323	18224	1.783058	1	0.424425	52.90386
GOTERM CC DIRECT	GO:0031965~nuclear membrane	11	2.37581	0.052921	EGFR, MTDH, LMNB1, CCND2,	443	229	18224	1.976047	1	0.423078	53.58953
GOTERM CC DIRECT	GO:0000777~condensed chromosome kinetochore	6	1.295896	0.060354	CENPS, TP53BP1, INCENP, RAS	443	87	18224	2.837083	1	0.4596	58.47561
GOTERM CC DIRECT	GO:0044291~cell-cell contact zone	3	0.647948	0.062796	RAP2C, TIAM1, FLOT1	443	17	18224	7.259594	1	0.465768	59.97356
GOTERM CC DIRECT	GO:0045121~membrane raft	10	2.159827	0.063827	EGFR, KRAS, PODXL, FLOT1, C	443	206	18224	1.996976	1	0.464047	60.59096
GOTERM CC DIRECT	GO:0071944~cell periphery	4	0.863931	0.068157	PHB2, RHOA, MSN, CTNNB1	443	39	18224	4.219251	1	0.479695	63.08737
GOTERM CC DIRECT	GO:0032444~activin responsive factor complex	2	0.431965	0.071015	SMAD4, SMAD2	443	3	18224	27.42513	1	0.487043	64.65383
GOTERM CC DIRECT	GO:0031528~microvillus membrane	3	0.647948	0.076515	PODXL, MSN, CTNNB1	443	19	18224	6.495426	1	0.50671	67.49642
GOTERM CC DIRECT	GO:0043209~myelin sheath	8	1.727862	0.077302	BCL2, PGAM4, FSCN1, NDRG1,	443	152	18224	2.165142	1	0.503389	67.88557
GOTERM CC DIRECT	GO:0005938~cell cortex	7	1.511879	0.07908	PFN1, PARD6B, NRBP1, FERM	443	123	18224	2.34117	1	0.50474	68.74815
GOTERM CC DIRECT	GO:0031463~Cul3-RING ubiquitin ligase complex	5	1.079914	0.079546	IPP, KLHL28, KLHL42, KEAP1,	443	67	18224	3.069977	1	0.500125	68.97085
GOTERM CC DIRECT	GO:0005637~nuclear inner membrane	4	0.863931	0.085865	LMNB1, SMAD3, DPY19L1, MA	443	43	18224	3.826763	1	0.52138	71.8463
GOTERM CC DIRECT	GO:0043235~receptor complex	7	1.511879	0.088996	EGFR, ACVR1B, NOTCH2, IGF1	443	127	18224	2.267432	1	0.52803	73.17746
GOTERM CC DIRECT	GO:0030175~filopodium	5	1.079914	0.093723	VCAM1, PODXL, FSCN1, ABI2,	443	71	18224	2.897021	1	0.54083	75.077
GOTERM CC DIRECT	GO:0005622~intracellular	41	8.855292	0.094001	RAB5B, ADCY6, BTC, BAP1, IL	443	1332	18224	1.26625	1	0.535511	75.18461
GOTERM CC DIRECT	GO:0005720~nuclear heterochromatin	3	0.647948	0.09863	ATRX, DNMT3A, H2AFZ	443	22	18224	5.609686	1	0.547269	76.91594

Category	Term	Count	%	PValue	Genes	List Total	Pop Hits	Pop Total	Fold Enricl	Bonferroni	Benjamini	FDR
GOTERM_MF_DIRECT	GO:0005515~protein binding	303	65.44276	7.69E-16	DLC1, LD	427	8785	16881	1.36355	5.21E-13	5.21E-13	1.17E-12
GOTERM_MF_DIRECT	GO:0003682~chromatin binding	35	7.559395	3.55E-10	CCNT2, M	427	391	16881	3.538845	2.38E-07	1.19E-07	5.32E-07
GOTERM_MF_DIRECT	GO:0031490~chromatin DNA binding	13	2.807775	1.78E-08	SUZ12, NC	427	58	16881	8.86106	1.20E-05	3.98E-06	2.67E-05
GOTERM_MF_DIRECT	GO:0019901~protein kinase binding	30	6.479482	1.02E-07	CCNT2, G	427	376	16881	3.154305	6.85E-05	1.71E-05	1.53E-04
GOTERM_MF_DIRECT	GO:0043565~sequence-specific DNA binding	35	7.559395	3.84E-07	FOXA2, T	427	518	16881	2.671213	2.57E-04	5.15E-05	5.76E-04
GOTERM_MF_DIRECT	GO:0008134~transcription factor binding	24	5.183585	9.51E-07	HMGB1, F	427	284	16881	3.340898	6.37E-04	1.06E-04	0.001425
GOTERM_MF_DIRECT	GO:0044212~transcription regulatory region DNA binding	19	4.103672	8.05E-06	ZNF831, F	427	213	16881	3.526503	0.005379	7.70E-04	0.012067
GOTERM_MF_DIRECT	GO:0003700~transcription factor activity, sequence-specific DNA binding	48	10.36717	1.06E-05	HMGB1, E	427	961	16881	1.974641	0.007096	8.90E-04	0.015933
GOTERM_MF_DIRECT	GO:0044822~poly(A) RNA binding	53	11.44708	1.85E-05	ZMAT3, R	427	1129	16881	1.85589	0.012311	0.001375	0.027715
GOTERM_MF_DIRECT	GO:0003725~double-stranded RNA binding	9	1.943844	1.41E-04	LSM14A, I	427	61	16881	5.832879	0.089903	0.009376	0.210574
GOTERM_MF_DIRECT	GO:0001228~transcriptional activator activity, RNA polymerase II transcription r	11	2.37581	1.41E-04	EP300, GA	427	95	16881	4.577616	0.089959	0.008533	0.21071
GOTERM_MF_DIRECT	GO:0001085~RNA polymerase II transcription factor binding	8	1.727862	1.63E-04	MTDH, GA	427	47	16881	6.729184	0.103534	0.009067	0.244265
GOTERM_MF_DIRECT	GO:0035257~nuclear hormone receptor binding	6	1.295896	1.85E-04	NCOA2, E	427	22	16881	10.78199	0.116344	0.009469	0.276386
GOTERM_MF_DIRECT	GO:0008013~beta-catenin binding	10	2.159827	2.19E-04	EP300, GS	427	82	16881	4.821214	0.136642	0.01044	0.328228
GOTERM_MF_DIRECT	GO:0005168~neurotrophin TRKA receptor binding	4	0.863931	3.02E-04	PLCG1, GI	427	6	16881	26.35597	0.182971	0.013382	0.451163
GOTERM_MF_DIRECT	GO:0003713~transcription coactivator activity	17	3.671706	5.85E-04	MTDH, RA	427	248	16881	2.709989	0.324463	0.024217	0.873868
GOTERM_MF_DIRECT	GO:0003677~DNA binding	64	13.82289	8.15E-04	E2F3, ZNF	427	1674	16881	1.511454	0.42097	0.03163	1.215204
GOTERM_MF_DIRECT	GO:0003924~GTPase activity	16	3.455724	9.17E-04	GNA13, R	427	234	16881	2.703177	0.459197	0.033574	1.366062
GOTERM_MF_DIRECT	GO:0005158~insulin receptor binding	6	1.295896	9.88E-04	IGF1R, PD	427	31	16881	7.651734	0.484381	0.034262	1.471252
GOTERM_MF_DIRECT	GO:0003690~double-stranded DNA binding	9	1.943844	9.91E-04	LSM14A, I	427	81	16881	4.392662	0.485395	0.032672	1.475591
GOTERM_MF_DIRECT	GO:0019903~protein phosphatase binding	8	1.727862	0.001015	EGFR, CD	427	63	16881	5.020185	0.493736	0.031894	1.511614
GOTERM_MF_DIRECT	GO:0000978~RNA polymerase II core promoter proximal region sequence-specif	20	4.319654	0.001776	FOXA2, Z	427	355	16881	2.227265	0.696009	0.052687	2.629299
GOTERM_MF_DIRECT	GO:0019003~GDP binding	7	1.511879	0.002314	RAB3B, K	427	54	16881	5.124772	0.788146	0.065246	3.412895
GOTERM_MF_DIRECT	GO:0001102~RNA polymerase II activating transcription factor binding	6	1.295896	0.002526	EP300, TP	427	38	16881	6.242204	0.81637	0.068182	3.721413
GOTERM_MF_DIRECT	GO:0042802~identical protein binding	33	7.12743	0.002648	MTSS1, LI	427	749	16881	1.741817	0.83076	0.068592	3.897062
GOTERM_MF_DIRECT	GO:0031625~ubiquitin protein ligase binding	17	3.671706	0.002667	EGFR, TP:	427	287	16881	2.341733	0.832886	0.066497	3.924238
GOTERM_MF_DIRECT	GO:0003723~RNA binding	26	5.615551	0.003264	RBM15B,	427	547	16881	1.879128	0.888154	0.07793	4.78365
GOTERM_MF_DIRECT	GO:0003886~DNA (cytosine-5-)-methyltransferase activity	3	0.647948	0.003686	DNMT3A,	427	4	16881	29.65047	0.915754	0.084567	5.385501
GOTERM_MF_DIRECT	GO:0005525~GTP binding	20	4.319654	0.00422	GNA13, R	427	384	16881	2.05906	0.941169	0.093072	6.142653
GOTERM_MF_DIRECT	GO:0001076~transcription factor activity, RNA polymerase II transcription factor	4	0.863931	0.004721	GATA4, SI	427	14	16881	11.29542	0.95803	0.1003	6.849273
GOTERM_MF_DIRECT	GO:0019899~enzyme binding	18	3.887689	0.004914	EGFR, CY	427	333	16881	2.136971	0.963137	0.100998	7.119318
GOTERM_MF_DIRECT	GO:0035035~histone acetyltransferase binding	5	1.079914	0.005067	SP1, ETS1	427	28	16881	7.059635	0.966733	0.10089	7.332398
GOTERM_MF_DIRECT	GO:0009008~DNA-methyltransferase activity	3	0.647948	0.00604	DNMT3A,	427	5	16881	23.72037	0.98274	0.115747	8.683107
GOTERM_MF_DIRECT	GO:0003705~transcription factor activity, RNA polymerase II distal enhancer seq	7	1.511879	0.006324	GATA6, JU	427	66	16881	4.192996	0.985739	0.117509	9.072244
GOTERM_MF_DIRECT	GO:0000977~RNA polymerase II regulatory region sequence-specific DNA bindi	13	2.807775	0.006694	RARG, KL	427	208	16881	2.470872	0.988889	0.120646	9.578718
GOTERM_MF_DIRECT	GO:0005200~structural constituent of cytoskeleton	9	1.943844	0.006725	YEATS4,	427	110	16881	3.234597	0.989123	0.118021	9.621759
GOTERM_MF_DIRECT	GO:0001190~transcriptional activator activity, RNA polymerase II transcription f	4	0.863931	0.006997	NOTCH1,	427	16	16881	9.883489	0.990945	0.119397	9.991808
GOTERM_MF_DIRECT	GO:0001077~transcriptional activator activity, RNA polymerase II core promoter	14	3.023758	0.007112	TFAM, ZN	427	236	16881	2.345235	0.99162	0.118243	10.14748
GOTERM_MF_DIRECT	GO:0046982~protein heterodimerization activity	22	4.75162	0.00757	EGFR, CE	427	465	16881	1.870424	0.993849	0.122379	10.76705
GOTERM_MF_DIRECT	GO:0004842~ubiquitin-protein transferase activity	17	3.671706	0.009638	IPP, XIAP,	427	329	16881	2.042788	0.998479	0.149742	13.51435
GOTERM_MF_DIRECT	GO:0070064~proline-rich region binding	4	0.863931	0.011449	PFN1, CYI	427	19	16881	8.322938	0.999554	0.171529	15.85565
GOTERM_MF_DIRECT	GO:0019900~kinase binding	7	1.511879	0.01161	LDHA, TL	427	75	16881	3.689836	0.9996	0.16997	16.06098
GOTERM_MF_DIRECT	GO:0000979~RNA polymerase II core promoter sequence-specific DNA binding	6	1.295896	0.014287	SUZ12, EP	427	57	16881	4.161469	0.999935	0.200859	19.4054
GOTERM_MF_DIRECT	GO:0004714~transmembrane receptor protein tyrosine kinase activity	5	1.079914	0.014998	EGFR, IGI	427	38	16881	5.201837	0.99996	0.205555	20.2728
GOTERM_MF_DIRECT	GO:0070412~R-SMAD binding	4	0.863931	0.015141	JUN, SMA	427	21	16881	7.530278	0.999964	0.203209	20.44632
GOTERM_MF_DIRECT	GO:0001227~transcriptional repressor activity, RNA polymerase II transcription	6	1.295896	0.016403	KLF12, MI	427	59	16881	4.020402	0.999985	0.214072	21.96019
GOTERM_MF_DIRECT	GO:0042803~protein homodimerization activity	29	6.263499	0.01863	NRBP1, C	427	730	16881	1.570527	0.999997	0.23515	24.56757
GOTERM_MF_DIRECT	GO:0000982~transcription factor activity, RNA polymerase II core promoter prox	4	0.863931	0.019429	SP1, ETS1	427	23	16881	6.875471	0.999998	0.239563	25.48319
GOTERM_MF_DIRECT	GO:0050839~cell adhesion molecule binding	6	1.295896	0.019951	VCAM1, F	427	62	16881	3.825867	0.999999	0.240847	26.07555
GOTERM_MF_DIRECT	GO:0046934~phosphatidylinositol-4,5-bisphosphate 3-kinase activity	6	1.295896	0.019951	EGFR, GR	427	62	16881	3.825867	0.999999	0.240847	26.07555
GOTERM_MF_DIRECT	GO:0001047~core promoter binding	6	1.295896	0.022575	NOTCH1,	427	64	16881	3.706309	1	0.263591	28.98826
GOTERM_MF_DIRECT	GO:0030971~receptor tyrosine kinase binding	5	1.079914	0.022738	PITPNM3,	427	43	16881	4.596972	1	0.260786	29.16613
GOTERM_MF_DIRECT	GO:0016303~1-phosphatidylinositol-3-kinase activity	5	1.079914	0.022738	GRB2, PIK	427	43	16881	4.596972	1	0.260786	29.16613
GOTERM_MF_DIRECT	GO:0000774~adenyl-nucleotide exchange factor activity	3	0.647948	0.025003	PFN1, BA	427	10	16881	11.86019	1	0.278379	31.58795
GOTERM_MF_DIRECT	GO:0005068~transmembrane receptor protein tyrosine kinase adaptor activity	3	0.647948	0.025003	SHC1, IRS	427	10	16881	11.86019	1	0.278379	31.58795
GOTERM_MF_DIRECT	GO:0001223~transcription coactivator binding	3	0.647948	0.025003	CCNT2, T	427	10	16881	11.86019	1	0.278379	31.58795
GOTERM_MF_DIRECT	GO:0000981~RNA polymerase II transcription factor activity, sequence-specific I	10	2.159827	0.029724	FOXA2, E	427	171	16881	2.311927	1	0.317136	36.38947
GOTERM_MF_DIRECT	GO:0001103~RNA polymerase II repressing transcription factor binding	4	0.863931	0.029806	SP1, GAT	427	27	16881	5.856883	1	0.313015	36.46996
GOTERM_MF_DIRECT	GO:0070411~I-SMAD binding	3	0.647948	0.030057	SMAD4, S	427	11	16881	10.78199	1	0.31048	36.71514
GOTERM_MF_DIRECT	GO:0017124~SH3 domain binding	8	1.727862	0.031222	GRB2, AB	427	119	16881	2.657745	1	0.315796	37.84524
GOTERM_MF_DIRECT	GO:0070410~co-SMAD binding	3	0.647948	0.035475	GATA4, SI	427	12	16881	9.883489	1	0.345947	41.81345
GOTERM_MF_DIRECT	GO:0004672~protein kinase activity	16	3.455724	0.039573	EGFR, NR	427	359	16881	1.761959	1	0.372761	45.41133
GOTERM_MF_DIRECT	GO:0005154~epidermal growth factor receptor binding	4	0.863931	0.042565	GRB2, BT	427	31	16881	5.101156	1	0.38979	47.90597
GOTERM_MF_DIRECT	GO:0002020~protease binding	7	1.511879	0.042667	TNFRSF1(	427	101	16881	2.739977	1	0.385481	47.98948
GOTERM_MF_DIRECT	GO:0032794~GTPase activating protein binding	3	0.647948	0.047324	TUBB, FM	427	14	16881	8.471562	1	0.412861	51.65619
GOTERM_MF_DIRECT	GO:0005524~ATP binding	49	10.58315	0.049492	NRBP1, PI	427	1495	16881	1.295762	1	0.422193	53.27897
GOTERM_MF_DIRECT	GO:0051718~DNA (cytosine-5-)-methyltransferase activity, acting on CpG subst	2	0.431965	0.049836	DNMT3A,	427	2	16881	39.53396	1	0.41938	53.53188
GOTERM_MF_DIRECT	GO:0005178~integrin binding	7	1.511879	0.049917	VCAM1, E	427	105	16881	2.635597	1	0.41495	53.59121
GOTERM_MF_DIRECT	GO:0004713~protein tyrosine kinase activity	8	1.727862	0.051733	EGFR, IGI	427	133	16881	2.377982	1	0.421626	54.90367
GOTERM_MF_DIRECT	GO:0008083~growth factor activity	9	1.943844	0.053323	BDNF, FG	427	162	16881	2.196331	1	0.42666	56.02425
GOTERM_MF_DIRECT	GO:0005159~insulin-like growth factor receptor binding	3	0.647948	0.053714	IGF2, SHC	427	15	16881	7.906792	1	0.424262	56.29564
GOTERM_MF_DIRECT	GO:0004674~protein serine/threonine kinase activity	16	3.455724	0.054851	CCNT2, N	427	376	16881	1.682296	1	0.426403	57.07624
GOTERM_MF_DIRECT	GO:0004722~protein serine/threonine phosphatase activity	5	1.079914	0.058463	CDC14B, I	427	58	16881	3.4081	1	0.442869	59.47098
GOTERM_MF_DIRECT	GO:0005518~collagen binding	5	1.079914	0.064664	SMAD4, S	427	60	16881	3.294496	1	0.472625	63.29347
GOTERM_MF_DIRECT	GO:0008270~zinc ion binding	39	8.423326	0.065234	BMI1, ZF	427	1169	16881	1.318926	1	0.470902	63.62734
GOTERM_MF_DIRECT	GO:0005088~Ras guanyl-nucleotide exchange factor activity	7	1.511879	0.071168	EGFR, GR	427	115	16881	2.406415	1	0.496917	66.93936
GOTERM_MF_DIRECT	GO:0050816~phosphothreonine binding	2	0.431965	0.073816	FBXW7, P	427	3	16881	26.35597	1	0.505298	68.32492
GOTERM_MF_DIRECT	GO:0071837~HMG box domain binding	3	0.647948	0.074516	SP1, JUN,	427	18	16881	6.588993	1	0.503974	68.6816
GOTERM_MF_DIRECT	GO:0005057~receptor signaling protein activity	4	0.863931	0.079445	BAG4, BA	427	40	16881	3.953396	1	0.522643	71.09153
GOTERM_MF_DIRECT	GO:0002039~p53 binding	5	1.079914	0.088921	EP300, TP	427	67	16881	2.950295	1	0.559998	75.24537
GOTERM_MF_DIRECT	GO:1990841~promoter-specific chromatin binding	3	0.647948	0.08957	BMI1, EZI	427	20	16881	5.930094	1	0.558031	75.50842
GOTERM_MF_DIRECT	GO:0030332~cyclin binding	3	0.647948	0.08957	CDKN1A,	427	20	16881	5.930094	1	0.558031	75.50842
GOTERM_MF_DIRECT	GO:0003727~single-stranded RNA binding	4	0.863931	0.094017	LSM14A, I	427	43	16881	3.677577	1	0.571773	77.24173
GOTERM_MF_DIRECT	GO:0003729~mRNA binding	7	1.511879	0.096885	ZFP36L1,	427	125	16881	2.213902	1	0.578638	78.29847
GOTERM_MF_DIRECT	GO:0045322~unmethylated CpG binding	2	0.431965	0.097193	DNMT3A,	427	4	16881	19.76698	1	0.575276	78.40912
GOTERM_MF_DIRECT	GO:0051434~BH3 domain binding	2	0.431965	0.097193	MCL1, BC	427	4	16881	19.76698	1	0.575276	78.40912
GOTERM_MF_DIRECT	GO:0045569~TRAIL binding	2	0.431965	0.097193	TNFRSF1(	427	4	16881	19.76698	1	0.575276	78.40912
GOTERM_MF_DIRECT	GO:0097100~supercoiled DNA binding	2	0.431965	0.097193	HMGB1, F	427	4	16881	19.76698	1	0.575276	78.40912
GOTERM_MF_DIRECT	GO:0048408~epidermal growth factor binding	2	0.431965	0.097193	EGFR, SH	427	4	16881	19.76698	1	0.575276	78.40912
GOTERM_MF_DIRECT	GO:0004872~receptor activity	10	2.159827	0.098959	TNFRSF1(	427	217	16881	1.821841	1	0.577656	79.03354
