# Supplementary material for: Integrated MicroRNA Expression Profile Reveals Dysregulated miR-20a-5p and miR-200a-3p in Liver Fibrosis
Source: Biomed Res Int. 2021 Jun 8;2021:9583932. doi: 10.1155/2021/9583932 (PMC8218919; doi:10.1155/2021/9583932)
Supplement: Supplementary 3 — Table S3: GO functions for target genes of the three downregulated miRNAs. [file 9583932.f3.pdf]

Category	Term	Count	%	PValue	Genes	List Total	Pop Hits	Pop Total	Fold Enricl	Bonferroni	Benjamini	FDR
GOTERM_BP_DIRECT	GO:0006351~transcription, DNA-templated	205	15.29851	1.77E-08	BBX, ROF	1214	1955	16792	1.45041	7.34E-05	7.34E-05	3.30E-05
GOTERM_BP_DIRECT	GO:0006977~DNA damage response, signal transduction by p53 class mediator	20	1.492537	3.87E-08	E2F1, RBI	1214	62	16792	4.461923	1.60E-04	8.02E-05	7.21E-05
GOTERM_BP_DIRECT	GO:0007179~transforming growth factor beta receptor signaling pathway	23	1.716418	4.47E-07	PARD3, PI	1214	92	16792	3.45799	0.001851	6.17E-04	8.34E-04
GOTERM_BP_DIRECT	GO:0045893~positive regulation of transcription, DNA-templated	68	5.074627	1.80E-06	E2F1, CHU	1214	515	16792	1.826356	0.007423	0.001861	0.003353
GOTERM_BP_DIRECT	GO:0000086~G2/M transition of mitotic cell cycle	27	2.014925	4.74E-06	CEP72, PP	1214	137	16792	2.726007	0.019473	0.003925	0.008848
GOTERM_BP_DIRECT	GO:0006468~protein phosphorylation	60	4.477612	8.78E-06	CDK19, N	1214	456	16792	1.819995	0.035762	0.006051	0.016385
GOTERM_BP_DIRECT	GO:1900740~positive regulation of protein insertion into mitochondrial membra	11	0.820896	2.94E-05	E2F1, YW	1214	30	16792	5.071719	0.114742	0.01726	0.054824
GOTERM_BP_DIRECT	GO:0035194~posttranscriptional gene silencing by RNA	6	0.447761	3.62E-05	AGO1, AG	1214	7	16792	11.85597	0.13946	0.018599	0.067559
GOTERM_BP_DIRECT	GO:0035280~miRNA loading onto RISC involved in gene silencing by miRNA	6	0.447761	3.62E-05	DICER1, A	1214	7	16792	11.85597	0.13946	0.018599	0.067559
GOTERM_BP_DIRECT	GO:0000122~negative regulation of transcription from RNA polymerase II prom	82	6.119403	4.16E-05	BACH1, E	1214	720	16792	1.575307	0.158416	0.018981	0.077574
GOTERM_BP_DIRECT	GO:0035278~miRNA mediated inhibition of translation	7	0.522388	8.87E-05	AGO1, AG	1214	12	16792	8.068644	0.30779	0.036118	0.165388
GOTERM_BP_DIRECT	GO:0071456~cellular response to hypoxia	19	1.41791	1.56E-04	E2F1, PPA	1214	96	16792	2.737576	0.476229	0.057096	0.290566
GOTERM_BP_DIRECT	GO:0051726~regulation of cell cycle	22	1.641791	2.07E-04	CDK19, E2	1214	124	16792	2.454058	0.57677	0.069146	0.386145
GOTERM_BP_DIRECT	GO:0075733~intracellular transport of virus	13	0.970149	2.17E-04	NUP98, TS	1214	51	16792	3.525794	0.592633	0.066748	0.403266
GOTERM_BP_DIRECT	GO:0045892~negative regulation of transcription, DNA-templated	59	4.402985	2.17E-04	E2F1, PPA	1214	499	16792	1.635442	0.592959	0.062185	0.403625
GOTERM_BP_DIRECT	GO:0045944~positive regulation of transcription from RNA polymerase II prom	101	7.537313	2.54E-04	MORF4L2	1214	981	16792	1.424086	0.651896	0.067933	0.473696
GOTERM_BP_DIRECT	GO:0001701~in utero embryonic development	28	2.089552	4.30E-04	STIL, GJA	1214	187	16792	2.071096	0.831884	0.105458	0.799111
GOTERM_BP_DIRECT	GO:0016032~viral process	39	2.910448	4.80E-04	CCL1, CR	1214	299	16792	1.804169	0.863455	0.110525	0.891911
GOTERM_BP_DIRECT	GO:0006974~cellular response to DNA damage stimulus	30	2.238806	4.86E-04	ARMT1, X	1214	208	16792	1.994994	0.866494	0.105837	0.901948
GOTERM_BP_DIRECT	GO:0030512~negative regulation of transforming growth factor beta receptor sig	14	1.044776	5.61E-04	GLG1, WN	1214	64	16792	3.025741	0.902268	0.115202	1.040936
GOTERM_BP_DIRECT	GO:0061418~regulation of transcription from RNA polymerase II promoter in re	9	0.671642	6.10E-04	BACH1, U	1214	28	16792	4.445987	0.920304	0.118805	1.131733
GOTERM_BP_DIRECT	GO:0003181~atrioventricular valve morphogenesis	6	0.447761	6.23E-04	BMP2, TG	1214	11	16792	7.544706	0.924613	0.115825	1.156458
GOTERM_BP_DIRECT	GO:0003148~outflow tract septum morphogenesis	8	0.597015	8.84E-04	GATA6, S	1214	23	16792	4.811117	0.97441	0.153476	1.635828
GOTERM_BP_DIRECT	GO:0016236~macroautophagy	15	1.119403	9.77E-04	LAMTOR1	1214	76	16792	2.729992	0.982599	0.161499	1.806369
GOTERM_BP_DIRECT	GO:0030308~negative regulation of cell growth	20	1.492537	0.001062	DCBLD2,	1214	121	16792	2.286274	0.987776	0.167661	1.962273
GOTERM_BP_DIRECT	GO:0007050~cell cycle arrest	22	1.641791	0.001198	WNT10B,	1214	141	16792	2.158178	0.99307	0.180349	2.212324
GOTERM_BP_DIRECT	GO:0018107~peptidyl-threonine phosphorylation	10	0.746269	0.001251	ACVR1B,	1214	38	16792	3.63999	0.994419	0.180904	2.307534
GOTERM_BP_DIRECT	GO:0048147~negative regulation of fibroblast proliferation	9	0.671642	0.001275	BAX, TRII	1214	31	16792	4.01573	0.994958	0.177927	2.35218
GOTERM_BP_DIRECT	GO:0035556~intracellular signal transduction	47	3.507463	0.001362	ZBTB33, N	1214	403	16792	1.613157	0.996492	0.182806	2.511372
GOTERM_BP_DIRECT	GO:0046777~protein autophosphorylation	25	1.865672	0.001415	MAPKAPI	1214	172	16792	2.010459	0.997173	0.183206	2.60611
GOTERM_BP_DIRECT	GO:0090625~mRNA cleavage involved in gene silencing by siRNA	4	0.298507	0.001423	AGO1, AG	1214	4	16792	13.83196	0.997271	0.178644	2.621562
GOTERM_BP_DIRECT	GO:0035196~production of miRNAs involved in gene silencing by miRNA	5	0.373134	0.001498	DICER1, A	1214	8	16792	8.644975	0.998	0.181654	2.757564
GOTERM_BP_DIRECT	GO:0048015~phosphatidylinositol-mediated signaling	18	1.343284	0.001503	NRG4, FG	1214	106	16792	2.348823	0.998046	0.177108	2.76772
GOTERM_BP_DIRECT	GO:0046686~response to cadmium ion	8	0.597015	0.001526	CDKN1B,	1214	25	16792	4.426227	0.998217	0.174535	2.807925
GOTERM_BP_DIRECT	GO:0060389~pathway-restricted SMAD protein phosphorylation	6	0.447761	0.001537	BMP2, SM	1214	13	16792	6.383982	0.998298	0.171001	2.828271
GOTERM_BP_DIRECT	GO:0031054~pre-miRNA processing	6	0.447761	0.001537	DICER1, A	1214	13	16792	6.383982	0.998298	0.171001	2.828271
GOTERM_BP_DIRECT	GO:0007507~heart development	26	1.940299	0.00154	PPARD, B	1214	183	16792	1.965197	0.998324	0.166905	2.834845
GOTERM_BP_DIRECT	GO:0006357~regulation of transcription from RNA polymerase II promoter	50	3.731343	0.001716	PPARD, F(	1214	441	16792	1.568249	0.999192	0.179456	3.153088
GOTERM_BP_DIRECT	GO:0098609~cell-cell adhesion	34	2.537313	0.002215	HSP90AB	1214	271	16792	1.735375	0.999898	0.219994	4.051989
GOTERM_BP_DIRECT	GO:0014911~positive regulation of smooth muscle cell migration	7	0.522388	0.002253	NRP1, PD(	1214	20	16792	4.841186	0.999913	0.218148	4.120453
GOTERM_BP_DIRECT	GO:0016567~protein ubiquitination	42	3.134328	0.002389	BACH1, X	1214	359	16792	1.618224	0.999951	0.224507	4.363719
GOTERM_BP_DIRECT	GO:0002480~antigen processing and presentation of exogenous peptide antigen	5	0.373134	0.002542	LNPEP, HI	1214	9	16792	7.684422	0.999974	0.231908	4.637802
GOTERM_BP_DIRECT	GO:0034629~cellular protein complex localization	5	0.373134	0.002542	NACC2, S	1214	9	16792	7.684422	0.999974	0.231908	4.637802
GOTERM_BP_DIRECT	GO:0000082~G1/S transition of mitotic cell cycle	17	1.268657	0.002577	PPP6C, PK	1214	102	16792	2.305327	0.999977	0.229683	4.700103
GOTERM_BP_DIRECT	GO:0009749~response to glucose	13	0.970149	0.003192	TXNIP, PF	1214	68	16792	2.644345	0.999998	0.270632	5.789513
GOTERM_BP_DIRECT	GO:0035279~mRNA cleavage involved in gene silencing by miRNA	4	0.298507	0.003366	AGO1, AG	1214	5	16792	11.06557	0.999999	0.277534	6.096101
GOTERM_BP_DIRECT	GO:1905007~positive regulation of epithelial to mesenchymal transition involve	4	0.298507	0.003366	TGFBR1,	1214	5	16792	11.06557	0.999999	0.277534	6.096101
GOTERM_BP_DIRECT	GO:0008283~cell proliferation	42	3.134328	0.00342	PTGES3, N	1214	366	16792	1.587274	0.999999	0.275914	6.191761
GOTERM_BP_DIRECT	GO:0000209~protein polyubiquitination	25	1.865672	0.003499	MKRN1, U	1214	184	16792	1.879342	1	0.27597	6.329406
GOTERM_BP_DIRECT	GO:0042147~retrograde transport, endosome to Golgi	13	0.970149	0.003615	STX6, ARI	1214	69	16792	2.606022	1	0.278524	6.533808
GOTERM_BP_DIRECT	GO:0032880~regulation of protein localization	11	0.820896	0.003619	STX6, SUI	1214	52	16792	2.925992	1	0.273718	6.539858
GOTERM_BP_DIRECT	GO:0034644~cellular response to UV	10	0.746269	0.003681	USP28, N4	1214	44	16792	3.143627	1	0.272777	6.648148
GOTERM_BP_DIRECT	GO:0060412~ventricular septum morphogenesis	8	0.597015	0.003843	SMAD7, T	1214	29	16792	3.815713	1	0.278044	6.931132
GOTERM_BP_DIRECT	GO:0032092~positive regulation of protein binding	12	0.895522	0.003927	HSP90AB	1214	61	16792	2.721041	1	0.278366	7.076771
GOTERM_BP_DIRECT	GO:0006355~regulation of transcription, DNA-templated	136	10.14925	0.004261	RALY, MC	1214	1504	16792	1.250762	1	0.293312	7.657639
GOTERM_BP_DIRECT	GO:0006915~apoptotic process	59	4.402985	0.004496	DLC1, IER	1214	567	16792	1.439305	1	0.30184	8.063319
GOTERM_BP_DIRECT	GO:0032480~negative regulation of type I interferon production	8	0.597015	0.004705	MAVS, CY	1214	30	16792	3.688523	1	0.308548	8.422918
GOTERM_BP_DIRECT	GO:0045165~cell fate commitment	10	0.746269	0.005025	SPRY2, W	1214	46	16792	3.006948	1	0.320749	8.969453
GOTERM_BP_DIRECT	GO:0000186~activation of MAPKK activity	10	0.746269	0.005025	MAP3K5,	1214	46	16792	3.006948	1	0.320749	8.969453
GOTERM_BP_DIRECT	GO:0090263~positive regulation of canonical Wnt signaling pathway	18	1.343284	0.005657	FZD9, CA	1214	120	16792	2.074794	1	0.347943	10.042
GOTERM_BP_DIRECT	GO:0050927~positive regulation of positive chemotaxis	5	0.373134	0.005924	S1PR1, F3	1214	11	16792	6.287255	1	0.355873	10.49129
GOTERM_BP_DIRECT	GO:0007223~Wnt signaling pathway, calcium modulating pathway	9	0.671642	0.005947	PPP3R1, A	1214	39	16792	3.191991	1	0.352008	10.5311
GOTERM_BP_DIRECT	GO:0006919~activation of cysteine-type endopeptidase activity involved in apo	14	1.044776	0.006333	DLC1, AC	1214	83	16792	2.333102	1	0.364986	11.17585
GOTERM_BP_DIRECT	GO:0016477~cell migration	23	1.716418	0.006345	FMNL3, A	1214	172	16792	1.849623	1	0.360646	11.19671
GOTERM_BP_DIRECT	GO:0048681~negative regulation of axon regeneration	4	0.298507	0.006371	EPHA4, CI	1214	6	16792	9.221307	1	0.35701	11.23947
GOTERM_BP_DIRECT	GO:0010862~positive regulation of pathway-restricted SMAD protein phosphor	10	0.746269	0.006721	ACVR1B,	1214	48	16792	2.881658	1	0.367667	11.8211
GOTERM_BP_DIRECT	GO:0007032~endosome organization	8	0.597015	0.006857	STX6, TM	1214	32	16792	3.45799	1	0.368804	12.04694
GOTERM_BP_DIRECT	GO:0030335~positive regulation of cell migration	24	1.791045	0.007044	BMP2, ST	1214	184	16792	1.804169	1	0.371987	12.35455
GOTERM_BP_DIRECT	GO:0042787~protein ubiquitination involved in ubiquitin-dependent protein cat	21	1.567164	0.007093	MYLIP, A	1214	153	16792	1.898504	1	0.369423	12.43515
GOTERM_BP_DIRECT	GO:0034976~response to endoplasmic reticulum stress	13	0.970149	0.007201	PDIA3, TN	1214	75	16792	2.39754	1	0.369321	12.61252
GOTERM_BP_DIRECT	GO:1900034~regulation of cellular response to heat	13	0.970149	0.007201	PTGES3, I	1214	75	16792	2.39754	1	0.369321	12.61252
GOTERM_BP_DIRECT	GO:0006914~autophagy	19	1.41791	0.007249	HMGB1, F	1214	133	16792	1.975994	1	0.366818	12.69095
GOTERM_BP_DIRECT	GO:0051301~cell division	39	2.910448	0.007771	CDK19, M	1214	350	16792	1.541276	1	0.382931	13.54475
GOTERM_BP_DIRECT	GO:0030509~BMP signaling pathway	13	0.970149	0.008004	BMP3, BN	1214	76	16792	2.365993	1	0.387364	13.92245
GOTERM_BP_DIRECT	GO:0006606~protein import into nucleus	11	0.820896	0.008067	TXNIP, NI	1214	58	16792	2.623303	1	0.385351	14.02448
GOTERM_BP_DIRECT	GO:0008284~positive regulation of cell proliferation	49	3.656716	0.00811	E2F3, FGF	1214	466	16792	1.454434	1	0.382632	14.09323
GOTERM_BP_DIRECT	GO:2001244~positive regulation of intrinsic apoptotic signaling pathway	8	0.597015	0.008172	CAV1, DN	1214	33	16792	3.353203	1	0.380714	14.19444
GOTERM_BP_DIRECT	GO:0018105~peptidyl-serine phosphorylation	18	1.343284	0.00849	CSNK1A1	1214	125	16792	1.991802	1	0.387965	14.70534
GOTERM_BP_DIRECT	GO:0030097~hemopoiesis	11	0.820896	0.009102	SGPL1, GI	1214	59	16792	2.57884	1	0.405054	15.68155
GOTERM_BP_DIRECT	GO:0001541~ovarian follicle development	9	0.671642	0.009421	BAX, BCL	1214	42	16792	2.963992	1	0.411581	16.18676
GOTERM_BP_DIRECT	GO:0007595~lactation	9	0.671642	0.009421	CAV1, GP	1214	42	16792	2.963992	1	0.411581	16.18676
GOTERM_BP_DIRECT	GO:0006986~response to unfolded protein	9	0.671642	0.009421	HSP90AB	1214	42	16792	2.963992	1	0.411581	16.18676
GOTERM_BP_DIRECT	GO:0045787~positive regulation of cell cycle	8	0.597015	0.009664	CCND1, C	1214	34	16792	3.254579	1	0.415404	16.57021
GOTERM_BP_DIRECT	GO:0014066~regulation of phosphatidylinositol 3-kinase signaling	13	0.970149	0.00982	MAPK1, II	1214	78	16792	2.305327	1	0.416301	16.8152
GOTERM_BP_DIRECT	GO:0030433~ER-associated ubiquitin-dependent protein catabolic process	11	0.820896	0.010234	TMEM67,	1214	60	16792	2.535859	1	0.425293	17.46163
GOTERM_BP_DIRECT	GO:0001783~B cell apoptotic process	4	0.298507	0.010553	TRAF3IP2	1214	7	16792	7.903977	1	0.431023	17.95614
GOTERM_BP_DIRECT	GO:0003183~mitral valve morphogenesis	4	0.298507	0.010553	SMAD6, B	1214	7	16792	7.903977	1	0.431023	17.95614
GOTERM_BP_DIRECT	GO:0072332~intrinsic apoptotic signaling pathway by p53 class mediator	7	0.522388	0.011149	E2F1, E2F	1214	27	16792	3.586064	1	0.444791	18.87352
GOTERM_BP_DIRECT	GO:0032728~positive regulation of interferon-beta production	7	0.522388	0.011149	POLR3G, I	1214	27	16792	3.586064	1	0.444791	18.87352
GOTERM_BP_DIRECT	GO:0048565~digestive tract development	8	0.597015	0.011345	CCNB1, C	1214	35	16792	3.161591	1	0.446406	19.17282
GOTERM_BP_DIRECT	GO:0050918~positive chemotaxis	8	0.597015	0.011345	HMGB1, F	1214	35	16792	3.161591	1	0.446406	19.17282
GOTERM_BP_DIRECT	GO:0035335~peptidyl-tyrosine dephosphorylation	15	1.119403	0.01156	SSH2, PTF	1214	99	16792	2.095752	1	0.448521	19.5
GOTERM_BP_DIRECT	GO:0043161~proteasome-mediated ubiquitin-dependent protein catabolic proces	25	1.865672	0.011691	RNF216, F	1214	203	16792	1.703443	1	0.448222	19.6992
GOTERM_BP_DIRECT	GO:0030036~actin cytoskeleton organization	18	1.343284	0.01236	DLC1, FM	1214	130	16792	1.915195	1	0.462741	20.70736
GOTERM_BP_DIRECT	GO:0042493~response to drug	34	2.537313	0.012454	HMGB2, S	1214	304	16792	1.546996	1	0.461283	20.84767
GOTERM_BP_DIRECT	GO:0097190~apoptotic signaling pathway	12	0.895522	0.012471	TNFRSF21	1214	71	16792	2.337796	1	0.4578	20.87289
GOTERM_BP_DIRECT	GO:2000145~regulation of cell motility	7	0.522388	0.013343	NRG4, RO	1214	28	16792	3.45799	1	0.47669	22.16593
GOTERM_BP_DIRECT	GO:0007049~cell cycle	26	1.940299	0.013886	TXNIP, E2	1214	217	16792	1.657286	1	0.486431	22.96118
GOTERM_BP_DIRECT	GO:0043988~histone H3-S28 phosphorylation	3	0.223881	0.01489	RPS6KA5,	1214	3	16792	13.83196	1	0.506786	24.41198
GOTERM_BP_DIRECT	GO:0003274~endocardial cushion fusion	3	0.223881	0.01489	TGFBR2, A	1214	3	16792	13.83196	1	0.506786	24.41198
GOTERM_BP_DIRECT	GO:0045214~sarcomere organization	7	0.522388	0.015821	MYLK3, C	1214	29	16792	3.338749	1	0.524271	25.73327
GOTERM_BP_DIRECT	GO:0001937~negative regulation of endothelial cell proliferation	7	0.522388	0.015821	CAV1, TG	1214	29	16792	3.338749	1	0.524271	25.73327
GOTERM_BP_DIRECT	GO:0060155~platelet dense granule organization	4	0.298507	0.015986	F2RL3, BI	1214	8	16792	6.91598	1	0.52402	25.96531
GOTERM_BP_DIRECT	GO:0030900~forebrain development	9	0.671642	0.016151	E2F1, DL(	1214	46	16792	2.706253	1	0.523766	26.19607
GOTERM_BP_DIRECT	GO:0007030~Golgi organization	12	0.895522	0.016736	CSNK1A1	1214	74	16792	2.243021	1	0.53261	27.01061
GOTERM_BP_DIRECT	GO:0043065~positive regulation of apoptotic process	33	2.462687	0.017275	CDK19, H	1214	300	16792	1.521516	1	0.540161	27.75406
GOTERM_BP_DIRECT	GO:0001657~ureteric bud development	8	0.597015	0.017647	SMAD7, S	1214	38	16792	2.911992	1	0.544015	28.262
GOTERM_BP_DIRECT	GO:0009790~embryo development	8	0.597015	0.017647	BMP2, FU	1214	38	16792	2.911992	1	0.544015	28.262
GOTERM_BP_DIRECT	GO:0021766~hippocampus development	10	0.746269	0.018146	DLX2, PH	1214	56	16792	2.469993	1	0.550305	28.93822
GOTERM_BP_DIRECT	GO:0008630~intrinsic apoptotic signaling pathway in response to DNA damage	9	0.671642	0.018269	E2F1, MC	1214	47	16792	2.648673	1	0.548998	29.10448
GOTERM_BP_DIRECT	GO:0070936~protein K48-linked ubiquitination	9	0.671642	0.018269	UBE2D3, I	1214	47	16792	2.648673	1	0.548998	29.10448
GOTERM_BP_DIRECT	GO:0002474~antigen processing and presentation of peptide antigen via MHC c	7	0.522388	0.0186	SEC23A, I	1214	30	16792	3.227457	1	0.55179	29.54926
GOTERM_BP_DIRECT	GO:0006397~mRNA processing	22	1.641791	0.018954	RBM23, P	1214	179	16792	1.700017	1	0.554957	30.02237
GOTERM_BP_DIRECT	GO:0015031~protein transport	41	3.059701	0.018995	RAB5B, T	1214	395	16792	1.435722	1	0.552078	30.07626
GOTERM_BP_DIRECT	GO:0048490~anterograde synaptic vesicle transport	5	0.373134	0.019431	BLOC1S4,	1214	15	16792	4.610653	1	0.556709	30.65347
GOTERM_BP_DIRECT	GO:0007264~small GTPase mediated signal transduction	28	2.089552	0.019544	RAB5B, R	1214	246	16792	1.574369	1	0.555235	30.80261
GOTERM_BP_DIRECT	GO:0002062~chondrocyte differentiation	8	0.597015	0.020206	MEF2D, B	1214	39	16792	2.837325	1	0.563837	31.66961
GOTERM_BP_DIRECT	GO:0008333~endosome to lysosome transport	8	0.597015	0.020206	AKTIP, TS	1214	39	16792	2.837325	1	0.563837	31.66961
GOTERM_BP_DIRECT	GO:0043401~steroid hormone mediated signaling pathway	10	0.746269	0.020212	NR1H2, PI	1214	57	16792	2.42666	1	0.560408	31.67675
GOTERM_BP_DIRECT	GO:0032091~negative regulation of protein binding	10	0.746269	0.020212	CAV1, RO	1214	57	16792	2.42666	1	0.560408	31.67675
GOTERM_BP_DIRECT	GO:0007067~mitotic nuclear division	28	2.089552	0.021467	TRIOBP, S	1214	248	16792	1.561673	1	0.578999	33.29158
GOTERM_BP_DIRECT	GO:0042771~intrinsic apoptotic signaling pathway in response to DNA damage	7	0.522388	0.021695	USP28, CI	1214	31	16792	3.123346	1	0.579392	33.58011
GOTERM_BP_DIRECT	GO:0019058~viral life cycle	7	0.522388	0.021695	TSG101, U	1214	31	16792	3.123346	1	0.579392	33.58011

GOTERM_BP_DIRECT	GO:0030032~lamellipodium assembly	7	0.522388	0.021695	ABLIM1,	1214	31	16792	3.123346	1	0.579392	33.58011
GOTERM_BP_DIRECT	GO:0001836~release of cytochrome c from mitochondria	6	0.447761	0.021982	FZD9, DN	1214	23	16792	3.608338	1	0.580787	33.94318
GOTERM_BP_DIRECT	GO:0016239~positive regulation of macroautophagy	6	0.447761	0.021982	HIF1A, UL	1214	23	16792	3.608338	1	0.580787	33.94318
GOTERM_BP_DIRECT	GO:0006511~ubiquitin-dependent protein catabolic process	22	1.641791	0.02239	NPLOC4,	1214	182	16792	1.671995	1	0.584139	34.45493
GOTERM_BP_DIRECT	GO:0070102~interleukin-6-mediated signaling pathway	4	0.298507	0.022708	GAB1, SM	1214	9	16792	6.147538	1	0.585959	34.85213
GOTERM_BP_DIRECT	GO:0008354~germ cell migration	4	0.298507	0.022708	TGFBR1,	1214	9	16792	6.147538	1	0.585959	34.85213
GOTERM_BP_DIRECT	GO:2000188~regulation of cholesterol homeostasis	4	0.298507	0.022708	RALY, NR	1214	9	16792	6.147538	1	0.585959	34.85213
GOTERM_BP_DIRECT	GO:0045184~establishment of protein localization	8	0.597015	0.023013	RCC2, NP	1214	40	16792	2.766392	1	0.587524	35.23054
GOTERM_BP_DIRECT	GO:0043123~positive regulation of I-kappaB kinase/NF-kappaB signaling	20	1.492537	0.023418	MAVS, F2	1214	161	16792	1.718256	1	0.590633	35.72924
GOTERM_BP_DIRECT	GO:0001666~response to hypoxia	21	1.567164	0.02358	BMP2, CA	1214	172	16792	1.688786	1	0.589881	35.92831
GOTERM_BP_DIRECT	GO:0000165~MAPK cascade	29	2.164179	0.024202	NRG4, CA	1214	262	16792	1.531019	1	0.596244	36.68566
GOTERM_BP_DIRECT	GO:0010628~positive regulation of gene expression	29	2.164179	0.024202	E2F1, PPA	1214	262	16792	1.531019	1	0.596244	36.68566
GOTERM_BP_DIRECT	GO:0051895~negative regulation of focal adhesion assembly	5	0.373134	0.024459	DLC1, RC	1214	16	16792	4.322488	1	0.596895	36.99523
GOTERM_BP_DIRECT	GO:1904355~positive regulation of telomere capping	5	0.373134	0.024459	MAPK1, M	1214	16	16792	4.322488	1	0.596895	36.99523
GOTERM_BP_DIRECT	GO:1900745~positive regulation of p38MAPK cascade	5	0.373134	0.024459	MAP3K5,	1214	16	16792	4.322488	1	0.596895	36.99523
GOTERM_BP_DIRECT	GO:0032924~activin receptor signaling pathway	5	0.373134	0.024459	ACVR1B,	1214	16	16792	4.322488	1	0.596895	36.99523
GOTERM_BP_DIRECT	GO:0032212~positive regulation of telomere maintenance via telomerase	7	0.522388	0.025119	MAPK1, M	1214	32	16792	3.025741	1	0.603544	37.78536
GOTERM_BP_DIRECT	GO:0010506~regulation of autophagy	9	0.671642	0.025805	HMGB1, U	1214	50	16792	2.489753	1	0.610359	38.59717
GOTERM_BP_DIRECT	GO:0046907~intracellular transport	6	0.447761	0.026161	BLOC1S4,	1214	24	16792	3.45799	1	0.612283	39.01479
GOTERM_BP_DIRECT	GO:0048041~focal adhesion assembly	6	0.447761	0.026161	DLC1, PD	1214	24	16792	3.45799	1	0.612283	39.01479
GOTERM_BP_DIRECT	GO:0006366~transcription from RNA polymerase II promoter	50	3.731343	0.027181	BACH1, F	1214	513	16792	1.348144	1	0.623377	40.19528
GOTERM_BP_DIRECT	GO:0048661~positive regulation of smooth muscle cell proliferation	10	0.746269	0.027422	IRAK4, S1	1214	60	16792	2.305327	1	0.62354	40.4712
GOTERM_BP_DIRECT	GO:0031647~regulation of protein stability	11	0.820896	0.028205	MAPK1, U	1214	70	16792	2.173594	1	0.630943	41.35921
GOTERM_BP_DIRECT	GO:0007265~Ras protein signal transduction	11	0.820896	0.028205	NRAS, US	1214	70	16792	2.173594	1	0.630943	41.35921
GOTERM_BP_DIRECT	GO:0003104~positive regulation of glomerular filtration	3	0.223881	0.028358	PDGFB, F	1214	4	16792	10.37397	1	0.629876	41.53058
GOTERM_BP_DIRECT	GO:0008356~asymmetric cell division	3	0.223881	0.028358	ACTR2, P	1214	4	16792	10.37397	1	0.629876	41.53058
GOTERM_BP_DIRECT	GO:1990086~lens fiber cell apoptotic process	3	0.223881	0.028358	E2F1, E2F	1214	4	16792	10.37397	1	0.629876	41.53058
GOTERM_BP_DIRECT	GO:0033631~cell-cell adhesion mediated by integrin	3	0.223881	0.028358	ITGA5, NI	1214	4	16792	10.37397	1	0.629876	41.53058
GOTERM_BP_DIRECT	GO:1901031~regulation of response to reactive oxygen species	3	0.223881	0.028358	SESN2, SE	1214	4	16792	10.37397	1	0.629876	41.53058
GOTERM_BP_DIRECT	GO:0032392~DNA geometric change	3	0.223881	0.028358	HMGB1, H	1214	4	16792	10.37397	1	0.629876	41.53058
GOTERM_BP_DIRECT	GO:0032909~regulation of transforming growth factor beta2 production	3	0.223881	0.028358	HIF1A, SM	1214	4	16792	10.37397	1	0.629876	41.53058
GOTERM_BP_DIRECT	GO:0010718~positive regulation of epithelial to mesenchymal transition	7	0.522388	0.028884	BMP2, TG	1214	33	16792	2.934052	1	0.633692	42.11903
GOTERM_BP_DIRECT	GO:0070059~intrinsic apoptotic signaling pathway in response to endoplasmic r	7	0.522388	0.028884	MAP3K5,	1214	33	16792	2.934052	1	0.633692	42.11903
GOTERM_BP_DIRECT	GO:0016055~Wnt signaling pathway	22	1.641791	0.029156	CSNK1A1	1214	187	16792	1.627289	1	0.634153	42.41982
GOTERM_BP_DIRECT	GO:0008285~negative regulation of cell proliferation	40	2.985075	0.029726	DLC1, TS	1214	396	16792	1.397168	1	0.63839	43.04806
GOTERM_BP_DIRECT	GO:0048208~COPII vesicle coating	10	0.746269	0.030183	SEC23A, I	1214	61	16792	2.267535	1	0.641102	43.54576
GOTERM_BP_DIRECT	GO:0031589~cell-substrate adhesion	5	0.373134	0.030201	PPARD, IT	1214	17	16792	4.068224	1	0.638378	43.56595
GOTERM_BP_DIRECT	GO:2000811~negative regulation of anoikis	5	0.373134	0.030201	CAV1, MC	1214	17	16792	4.068224	1	0.638378	43.56595
GOTERM_BP_DIRECT	GO:0033160~positive regulation of protein import into nucleus, translocation	4	0.298507	0.030728	HSP90AB	1214	10	16792	5.532784	1	0.641899	44.13446
GOTERM_BP_DIRECT	GO:0046902~regulation of mitochondrial membrane permeability	4	0.298507	0.030728	BCL2, TP	1214	10	16792	5.532784	1	0.641899	44.13446
GOTERM_BP_DIRECT	GO:0042118~endothelial cell activation	4	0.298507	0.030728	TGFBR1,	1214	10	16792	5.532784	1	0.641899	44.13446
GOTERM_BP_DIRECT	GO:0097192~extrinsic apoptotic signaling pathway in absence of ligand	7	0.522388	0.033003	MCL1, BA	1214	34	16792	2.847757	1	0.665656	46.53104
GOTERM_BP_DIRECT	GO:0001837~epithelial to mesenchymal transition	7	0.522388	0.033003	FAM83D,	1214	34	16792	2.847757	1	0.665656	46.53104
GOTERM_BP_DIRECT	GO:0060395~SMAD protein signal transduction	10	0.746269	0.033131	LNPEP, BI	1214	62	16792	2.230961	1	0.66423	46.66347
GOTERM_BP_DIRECT	GO:0042981~regulation of apoptotic process	24	1.791045	0.034587	TNFRSF21	1214	213	16792	1.558531	1	0.67738	48.14136
GOTERM_BP_DIRECT	GO:0045727~positive regulation of translation	9	0.671642	0.035272	MAPK1, E	1214	53	16792	2.348823	1	0.681846	48.82362
GOTERM_BP_DIRECT	GO:0040008~regulation of growth	9	0.671642	0.035272	MORF4L1	1214	53	16792	2.348823	1	0.681846	48.82362
GOTERM_BP_DIRECT	GO:0045930~negative regulation of mitotic cell cycle	6	0.447761	0.035955	CDKN1B,	1214	26	16792	3.191991	1	0.686171	49.4958
GOTERM_BP_DIRECT	GO:0006457~protein folding	21	1.567164	0.035989	HSP90AB	1214	180	16792	1.613729	1	0.68375	49.52865
GOTERM_BP_DIRECT	GO:0060070~canonical Wnt signaling pathway	12	0.895522	0.036027	FZD9, TBI	1214	83	16792	1.999802	1	0.681388	49.56531
GOTERM_BP_DIRECT	GO:0010039~response to iron ion	5	0.373134	0.036672	CCND1, B	1214	18	16792	3.842211	1	0.685245	50.19134
GOTERM_BP_DIRECT	GO:0045089~positive regulation of innate immune response	5	0.373134	0.036672	POLR3G,	1214	18	16792	3.842211	1	0.685245	50.19134
GOTERM_BP_DIRECT	GO:0036120~cellular response to platelet-derived growth factor stimulus	5	0.373134	0.036672	CREB1, H	1214	18	16792	3.842211	1	0.685245	50.19134
GOTERM_BP_DIRECT	GO:0010501~RNA secondary structure unwinding	8	0.597015	0.036906	DDX19A,	1214	44	16792	2.514902	1	0.6849	50.41669
GOTERM_BP_DIRECT	GO:0023014~signal transduction by protein phosphorylation	8	0.597015	0.036906	PAK6, AC	1214	44	16792	2.514902	1	0.6849	50.41669
GOTERM_BP_DIRECT	GO:0010629~negative regulation of gene expression	17	1.268657	0.038454	LDLR, PD	1214	137	16792	1.716375	1	0.697421	51.88256
GOTERM_BP_DIRECT	GO:0006413~translational initiation	17	1.268657	0.038454	RPL17, RI	1214	137	16792	1.716375	1	0.697421	51.88256
GOTERM_BP_DIRECT	GO:0048146~positive regulation of fibroblast proliferation	9	0.671642	0.038888	CCNB1, E	1214	54	16792	2.305327	1	0.69891	52.28606
GOTERM_BP_DIRECT	GO:0090161~Golgi ribbon formation	4	0.298507	0.040029	STX6, ST	1214	11	16792	5.029804	1	0.706929	53.33192
GOTERM_BP_DIRECT	GO:0031571~mitotic G1 DNA damage checkpoint	4	0.298507	0.040029	RPA2, CC	1214	11	16792	5.029804	1	0.706929	53.33192
GOTERM_BP_DIRECT	GO:0060213~positive regulation of nuclear-transcribed mRNA poly(A) tail shor	4	0.298507	0.040029	BTG2, AG	1214	11	16792	5.029804	1	0.706929	53.33192
GOTERM_BP_DIRECT	GO:0030278~regulation of ossification	4	0.298507	0.040029	MAPK1, E	1214	11	16792	5.029804	1	0.706929	53.33192
GOTERM_BP_DIRECT	GO:0030514~negative regulation of BMP signaling pathway	8	0.597015	0.041086	NBL1, WN	1214	45	16792	2.459015	1	0.713885	54.28091
GOTERM_BP_DIRECT	GO:0008542~visual learning	8	0.597015	0.041086	APP, HIF1	1214	45	16792	2.459015	1	0.713885	54.28091
GOTERM_BP_DIRECT	GO:0007029~endoplasmic reticulum organization	6	0.447761	0.041593	DNM1L, A	1214	27	16792	3.073769	1	0.715806	54.73021
GOTERM_BP_DIRECT	GO:0055007~cardiac muscle cell differentiation	6	0.447761	0.041593	BMP2, GA	1214	27	16792	3.073769	1	0.715806	54.73021
GOTERM_BP_DIRECT	GO:0007611~learning or memory	7	0.522388	0.042335	FZD9, EG	1214	36	16792	2.689548	1	0.719715	55.37957
GOTERM_BP_DIRECT	GO:0042059~negative regulation of epidermal growth factor receptor signaling p	7	0.522388	0.042335	SPRY2, R	1214	36	16792	2.689548	1	0.719715	55.37957
GOTERM_BP_DIRECT	GO:0016925~protein sumoylation	15	1.119403	0.042527	NUP98, E	1214	117	16792	1.773328	1	0.718842	55.54595
GOTERM_BP_DIRECT	GO:0006897~endocytosis	17	1.268657	0.043047	DNM1L, L	1214	139	16792	1.691679	1	0.720773	55.99458
GOTERM_BP_DIRECT	GO:0042276~error-prone translesion synthesis	5	0.373134	0.043877	RPA2, RF	1214	19	16792	3.63999	1	0.725233	56.70061
GOTERM_BP_DIRECT	GO:0045672~positive regulation of osteoclast differentiation	5	0.373134	0.043877	IL23R, CR	1214	19	16792	3.63999	1	0.725233	56.70061
GOTERM_BP_DIRECT	GO:0070987~error-free translesion synthesis	5	0.373134	0.043877	RPA2, NP	1214	19	16792	3.63999	1	0.725233	56.70061
GOTERM_BP_DIRECT	GO:0006895~Golgi to endosome transport	5	0.373134	0.043877	TGOLN2,	1214	19	16792	3.63999	1	0.725233	56.70061
GOTERM_BP_DIRECT	GO:0006089~lactate metabolic process	3	0.223881	0.045019	LDHB, HI	1214	5	16792	8.299176	1	0.732089	57.65547
GOTERM_BP_DIRECT	GO:0043550~regulation of lipid kinase activity	3	0.223881	0.045019	RBL2, RB	1214	5	16792	8.299176	1	0.732089	57.65547
GOTERM_BP_DIRECT	GO:1990314~cellular response to insulin-like growth factor stimulus	3	0.223881	0.045019	SLC25A33	1214	5	16792	8.299176	1	0.732089	57.65547
GOTERM_BP_DIRECT	GO:0006686~sphingomyelin biosynthetic process	3	0.223881	0.045019	SAMD8, S	1214	5	16792	8.299176	1	0.732089	57.65547
GOTERM_BP_DIRECT	GO:0003289~atrial septum primum morphogenesis	3	0.223881	0.045019	SOX4, AC	1214	5	16792	8.299176	1	0.732089	57.65547
GOTERM_BP_DIRECT	GO:0016575~histone deacetylation	8	0.597015	0.045561	MORF4L1	1214	46	16792	2.405558	1	0.733987	58.10176
GOTERM_BP_DIRECT	GO:0016197~endosomal transport	10	0.746269	0.046905	AP5B1, TS	1214	66	16792	2.095752	1	0.742036	59.18855
GOTERM_BP_DIRECT	GO:0006367~transcription initiation from RNA polymerase II promoter	18	1.343284	0.047702	E2F2, PPA	1214	152	16792	1.637995	1	0.745693	59.82047
GOTERM_BP_DIRECT	GO:0045931~positive regulation of mitotic cell cycle	6	0.447761	0.047734	CCNB1, E	1214	28	16792	2.963992	1	0.743591	59.84631
GOTERM_BP_DIRECT	GO:0043388~positive regulation of DNA binding	6	0.447761	0.047734	HMGB1, H	1214	28	16792	2.963992	1	0.743591	59.84631
GOTERM_BP_DIRECT	GO:0018108~peptidyl-tyrosine phosphorylation	18	1.343284	0.050214	NRG4, HS	1214	153	16792	1.627289	1	0.759247	61.75289
GOTERM_BP_DIRECT	GO:0031532~actin cytoskeleton reorganization	8	0.597015	0.050337	S1PR2, S1	1214	47	16792	2.354376	1	0.757827	61.84503
GOTERM_BP_DIRECT	GO:0031629~synaptic vesicle fusion to presynaptic active zone membrane	4	0.298507	0.050578	STX4, SN	1214	12	16792	4.610653	1	0.757241	62.02493
GOTERM_BP_DIRECT	GO:0050921~positive regulation of chemotaxis	4	0.298507	0.050578	STX4, PD	1214	12	16792	4.610653	1	0.757241	62.02493
GOTERM_BP_DIRECT	GO:0015908~fatty acid transport	4	0.298507	0.050578	PPARD, M	1214	12	16792	4.610653	1	0.757241	62.02493
GOTERM_BP_DIRECT	GO:1902895~positive regulation of pri-miRNA transcription from RNA polyme	5	0.373134	0.051816	BMP2, HI	1214	20	16792	3.45799	1	0.763496	62.93812
GOTERM_BP_DIRECT	GO:0048545~response to steroid hormone	5	0.373134	0.051816	HMGB2, T	1214	20	16792	3.45799	1	0.763496	62.93812
GOTERM_BP_DIRECT	GO:0072583~clathrin-mediated endocytosis	5	0.373134	0.051816	FNBP1L, I	1214	20	16792	3.45799	1	0.763496	62.93812
GOTERM_BP_DIRECT	GO:0032438~melanosome organization	5	0.373134	0.051816	BLOC1S4,	1214	20	16792	3.45799	1	0.763496	62.93812
GOTERM_BP_DIRECT	GO:0051607~defense response to virus	19	1.41791	0.052099	POLR3G,	1214	165	16792	1.592771	1	0.763184	63.14421
GOTERM_BP_DIRECT	GO:0038128~ERBB2 signaling pathway	7	0.522388	0.053172	NRAS, NR	1214	38	16792	2.547993	1	0.768107	63.91482
GOTERM_BP_DIRECT	GO:0030522~intracellular receptor signaling pathway	7	0.522388	0.053172	DCBLD2,	1214	38	16792	2.547993	1	0.768107	63.91482
GOTERM_BP_DIRECT	GO:2001237~negative regulation of extrinsic apoptotic signaling pathway	7	0.522388	0.053172	NRP1, TG	1214	38	16792	2.547993	1	0.768107	63.91482
GOTERM_BP_DIRECT	GO:0045666~positive regulation of neuron differentiation	11	0.820896	0.053595	NBL1, BM	1214	78	16792	1.950661	1	0.76869	64.21461
GOTERM_BP_DIRECT	GO:0042113~B cell activation	6	0.447761	0.054383	LAT2, AK	1214	29	16792	2.861785	1	0.7716	64.76612
GOTERM_BP_DIRECT	GO:0051973~positive regulation of telomerase activity	6	0.447761	0.054383	MAPK1, M	1214	29	16792	2.861785	1	0.7716	64.76612
GOTERM_BP_DIRECT	GO:0000289~nuclear-transcribed mRNA poly(A) tail shortening	6	0.447761	0.054383	PAN3, CN	1214	29	16792	2.861785	1	0.7716	64.76612
GOTERM_BP_DIRECT	GO:0000281~mitotic cytokinesis	6	0.447761	0.054383	CKAP2, K	1214	29	16792	2.861785	1	0.7716	64.76612
GOTERM_BP_DIRECT	GO:0045909~positive regulation of vasodilation	6	0.447761	0.054383	PPARD, F	1214	29	16792	2.861785	1	0.7716	64.76612
GOTERM_BP_DIRECT	GO:0042149~cellular response to glucose starvation	6	0.447761	0.054383	MTMR3,	1214	29	16792	2.861785	1	0.7716	64.76612
GOTERM_BP_DIRECT	GO:0048008~platelet-derived growth factor receptor signaling pathway	6	0.447761	0.054383	TXNIP, SC	1214	29	16792	2.861785	1	0.7716	64.76612
GOTERM_BP_DIRECT	GO:0000910~cytokinesis	8	0.597015	0.055418	KIF23, RA	1214	48	16792	2.305327	1	0.775985	65.47854
GOTERM_BP_DIRECT	GO:0031047~gene silencing by RNA	14	1.044776	0.056902	NUP98, R	1214	111	16792	1.744572	1	0.782952	66.47653
GOTERM_BP_DIRECT	GO:0006605~protein targeting	7	0.522388	0.059166	YWHAZ,	1214	39	16792	2.48266	1	0.7941	67.94633
GOTERM_BP_DIRECT	GO:0051056~regulation of small GTPase mediated signal transduction	16	1.19403	0.059804	DLC1, GD	1214	134	16792	1.651577	1	0.79567	68.34945
GOTERM_BP_DIRECT	GO:0019083~viral transcription	14	1.044776	0.060319	RPL17, NU	1214	112	16792	1.728995	1	0.79653	68.67126
GOTERM_BP_DIRECT	GO:0048844~artery morphogenesis	5	0.373134	0.060483	NRP1, SM	1214	21	16792	3.293324	1	0.795441	68.77288
GOTERM_BP_DIRECT	GO:0051216~cartilage development	9	0.671642	0.060645	BMP3, DL	1214	59	16792	2.10996	1	0.794352	68.87338
GOTERM_BP_DIRECT	GO:0007254~JNK cascade	8	0.597015	0.060806	IRAK4, M	1214	49	16792	2.258279	1	0.793266	68.97301
GOTERM_BP_DIRECT	GO:0071773~cellular response to BMP stimulus	6	0.447761	0.06154	BMP2, GA	1214	30	16792	2.766392	1	0.795326	69.42189
GOTERM_BP_DIRECT	GO:0034975~protein folding in endoplasmic reticulum	4	0.298507	0.062323	HSP90B1,	1214	13	16792	4.255988	1	0.797614	69.8948
GOTERM_BP_DIRECT	GO:0033962~cytoplasmic mRNA processing body assembly	4	0.298507	0.062323	PATL1, LS	1214	13	16792	4.255988	1	0.797614	69.8948
GOTERM_BP_DIRECT	GO:0032479~regulation of type I interferon production	4	0.298507	0.062323	DTX4, TR	1214	13	16792	4.255988	1	0.797614	69.8948
GOTERM_BP_DIRECT	GO:0090160~Golgi to lysosome transport	3	0.223881	0.064343	AP1G1, A	1214	6	16792	6.91598	1	0.806268	71.0816
GOTERM_BP_DIRECT	GO:0009299~mRNA transcription	3	0.223881	0.064343	PPARD, E	1214	6	16792	6.91598	1	0.806268	71.0816
GOTERM_BP_DIRECT	GO:0060430~lung saccule development	3	0.223881	0.064343	PTGES3,	1214	6	16792	6.91598	1	0.806268	71.0816
GOTERM_BP_DIRECT	GO:0010887~negative regulation of cholesterol storage	3	0.223881	0.064343	NR1H2, P	1214	6	16792	6.91598	1	0.806268	71.0816
GOTERM_BP_DIRECT	GO:0061635~regulation of protein complex stability	3	0.223881	0.064343	SQSTM1,	1214	6	16792	6.91598	1	0.806268	71.0816
GOTERM_BP_DIRECT	GO:0038092~nodal signaling pathway	3	0.223881	0.064343	ACVR1B,	1214	6	16792	6.91598	1	0.806268	71.0816
GOTERM_BP_DIRECT	GO:0051438~regulation of ubiquitin-protein transferase activity	3	0.223881	0.064343	ZYG11A,	1214	6	16792	6.91598	1	0.806268	71.0816

GOTERM BP DIRECT	GO:0006207~'de novo' pyrimidine nucleobase biosynthetic process	3	0.223881	0.064343	DHODH, (	1214	6	16792	6.91598	1	0.806268	71.0816
GOTERM BP DIRECT	GO:1903672~positive regulation of sprouting angiogenesis	3	0.223881	0.064343	HMGB1, I	1214	6	16792	6.91598	1	0.806268	71.0816
GOTERM BP DIRECT	GO:0007346~regulation of mitotic cell cycle	7	0.522388	0.065546	CYLD, CD	1214	40	16792	2.420593	1	0.810455	71.7674
GOTERM BP DIRECT	GO:0043280~positive regulation of cysteine-type endopeptidase activity involve	7	0.522388	0.065546	HMGB1, N	1214	40	16792	2.420593	1	0.810455	71.7674
GOTERM BP DIRECT	GO:0045669~positive regulation of osteoblast differentiation	9	0.671642	0.065756	BMP2, WN	1214	60	16792	2.074794	1	0.809639	71.88568
GOTERM BP DIRECT	GO:0007411~axon guidance	18	1.343284	0.067261	ZNF280B,	1214	159	16792	1.565882	1	0.815149	72.71838
GOTERM BP DIRECT	GO:0002576~platelet degranulation	13	0.970149	0.06765	PHACTR2	1214	103	16792	1.745781	1	0.815194	72.9299
GOTERM BP DIRECT	GO:0071260~cellular response to mechanical stimulus	10	0.746269	0.068855	SLC38A2,	1214	71	16792	1.948163	1	0.819079	73.57543
GOTERM BP DIRECT	GO:1990090~cellular response to nerve growth factor stimulus	6	0.447761	0.069202	E2F1, APP	1214	31	16792	2.677154	1	0.818908	73.75859
GOTERM BP DIRECT	GO:0010332~response to gamma radiation	6	0.447761	0.069202	BAX, BCL	1214	31	16792	2.677154	1	0.818908	73.75859
GOTERM BP DIRECT	GO:0043552~positive regulation of phosphatidylinositol 3-kinase activity	6	0.447761	0.069202	PDGFB, S	1214	31	16792	2.677154	1	0.818908	73.75859
GOTERM BP DIRECT	GO:1904886~beta-catenin destruction complex disassembly	5	0.373134	0.069865	CSNK1A1	1214	22	16792	3.143627	1	0.820192	74.10496
GOTERM BP DIRECT	GO:0006470~protein dephosphorylation	15	1.119403	0.070697	PPP2R1A,	1214	126	16792	1.646662	1	0.822219	74.53389
GOTERM BP DIRECT	GO:0006950~response to stress	9	0.671642	0.071126	HSP90AB	1214	61	16792	2.040781	1	0.822404	74.75196
GOTERM BP DIRECT	GO:0007219~Notch signaling pathway	14	1.044776	0.071383	DTX4, AP	1214	115	16792	1.683891	1	0.821825	74.88208
GOTERM BP DIRECT	GO:0001649~osteoblast differentiation	13	0.970149	0.071699	BMP3, BM	1214	104	16792	1.728995	1	0.821511	75.0409
GOTERM BP DIRECT	GO:0031397~negative regulation of protein ubiquitination	7	0.522388	0.072312	CAV1, N4	1214	41	16792	2.361554	1	0.822515	75.34668
GOTERM BP DIRECT	GO:0044255~cellular lipid metabolic process	7	0.522388	0.072312	NR1H2, TI	1214	41	16792	2.361554	1	0.822515	75.34668
GOTERM BP DIRECT	GO:1902043~positive regulation of extrinsic apoptotic signaling pathway via de	4	0.298507	0.075204	PEA15, SK	1214	14	16792	3.951989	1	0.83318	76.74161
GOTERM BP DIRECT	GO:0042176~regulation of protein catabolic process	4	0.298507	0.075204	FAM83D,	1214	14	16792	3.951989	1	0.83318	76.74161
GOTERM BP DIRECT	GO:0010801~negative regulation of peptidyl-threonine phosphorylation	4	0.298507	0.075204	SPRY2, PA	1214	14	16792	3.951989	1	0.83318	76.74161
GOTERM BP DIRECT	GO:0003407~neural retina development	4	0.298507	0.075204	ATP2B1, F	1214	14	16792	3.951989	1	0.83318	76.74161
GOTERM BP DIRECT	GO:2001241~positive regulation of extrinsic apoptotic signaling pathway in abs	4	0.298507	0.075204	PPP2R1A,	1214	14	16792	3.951989	1	0.83318	76.74161
GOTERM BP DIRECT	GO:0051016~barbed-end actin filament capping	4	0.298507	0.075204	TRIOBP, T	1214	14	16792	3.951989	1	0.83318	76.74161
GOTERM BP DIRECT	GO:0030194~positive regulation of blood coagulation	4	0.298507	0.075204	SERPINE1	1214	14	16792	3.951989	1	0.83318	76.74161
GOTERM BP DIRECT	GO:0032461~positive regulation of protein oligomerization	4	0.298507	0.075204	SH3GLB1,	1214	14	16792	3.951989	1	0.83318	76.74161
GOTERM BP DIRECT	GO:0071425~hematopoietic stem cell proliferation	4	0.298507	0.075204	WNT1, WI	1214	14	16792	3.951989	1	0.83318	76.74161
GOTERM BP DIRECT	GO:0006461~protein complex assembly	14	1.044776	0.075346	PPP2R1A,	1214	116	16792	1.669375	1	0.83212	76.80834
GOTERM BP DIRECT	GO:0046854~phosphatidylinositol phosphorylation	12	0.895522	0.075976	NRG4, FG	1214	94	16792	1.765782	1	0.833073	77.10143
GOTERM BP DIRECT	GO:1900026~positive regulation of substrate adhesion-dependent cell spreading	6	0.447761	0.077366	TRIOBP, A	1214	32	16792	2.593493	1	0.837061	77.73537
GOTERM BP DIRECT	GO:0061024~membrane organization	6	0.447761	0.077366	LNPEP, Y	1214	32	16792	2.593493	1	0.837061	77.73537
GOTERM BP DIRECT	GO:0042220~response to cocaine	6	0.447761	0.077366	HSP90AB	1214	32	16792	2.593493	1	0.837061	77.73537
GOTERM BP DIRECT	GO:0045595~regulation of cell differentiation	6	0.447761	0.077366	SPRY2, PI	1214	32	16792	2.593493	1	0.837061	77.73537
GOTERM BP DIRECT	GO:0001974~blood vessel remodeling	6	0.447761	0.077366	EPAS1, BA	1214	32	16792	2.593493	1	0.837061	77.73537
GOTERM BP DIRECT	GO:0007259~JAK-STAT cascade	6	0.447761	0.077366	IFNAR2, F	1214	32	16792	2.593493	1	0.837061	77.73537
GOTERM BP DIRECT	GO:0043547~positive regulation of GTPase activity	51	3.80597	0.077758	DLC1, NR	1214	565	16792	1.248549	1	0.837014	77.91105
GOTERM BP DIRECT	GO:0009791~post-embryonic development	10	0.746269	0.079171	SGPL1, TC	1214	73	16792	1.894789	1	0.840949	78.53421
GOTERM BP DIRECT	GO:0050729~positive regulation of inflammatory response	10	0.746269	0.079171	CDK19, C	1214	73	16792	1.894789	1	0.840949	78.53421
GOTERM BP DIRECT	GO:0001658~branching involved in ureteric bud morphogenesis	7	0.522388	0.079462	WNT1, BM	1214	42	16792	2.305327	1	0.840499	78.66042
GOTERM BP DIRECT	GO:0032967~positive regulation of collagen biosynthetic process	5	0.373134	0.079946	AMELX, I	1214	23	16792	3.006948	1	0.840789	78.86849
GOTERM BP DIRECT	GO:0006479~protein methylation	5	0.373134	0.079946	ARMT1, B	1214	23	16792	3.006948	1	0.840789	78.86849
GOTERM BP DIRECT	GO:0000083~regulation of transcription involved in G1/S transition of mitotic c	5	0.373134	0.079946	BACH1, R	1214	23	16792	3.006948	1	0.840789	78.86849
GOTERM BP DIRECT	GO:0071398~cellular response to fatty acid	5	0.373134	0.079946	CCNB1, E	1214	23	16792	3.006948	1	0.840789	78.86849
GOTERM BP DIRECT	GO:0030307~positive regulation of cell growth	11	0.820896	0.080332	EIF4G2, C	1214	84	16792	1.811328	1	0.840708	79.03342
GOTERM BP DIRECT	GO:0030336~negative regulation of cell migration	12	0.895522	0.08061	DLC1, AD	1214	95	16792	1.747195	1	0.840217	79.15118
GOTERM BP DIRECT	GO:0009611~response to wounding	9	0.671642	0.082643	F2RL3, W	1214	63	16792	1.975994	1	0.846244	79.99465
GOTERM BP DIRECT	GO:0043066~negative regulation of apoptotic process	42	3.134328	0.083642	ARL6IP1,	1214	455	16792	1.276796	1	0.848349	80.39732
GOTERM BP DIRECT	GO:0001525~angiogenesis	23	1.716418	0.084488	SLC12A6,	1214	223	16792	1.426615	1	0.849868	80.73218
GOTERM BP DIRECT	GO:0000187~activation of MAPK activity	13	0.970149	0.084767	BMP2, MA	1214	107	16792	1.680519	1	0.849378	80.84145
GOTERM BP DIRECT	GO:0009636~response to toxic substance	11	0.820896	0.085463	MAPK1, W	1214	85	16792	1.790018	1	0.85035	81.11156
GOTERM BP DIRECT	GO:0010586~miRNA metabolic process	3	0.223881	0.085859	AGO1, AG	1214	7	16792	5.927983	1	0.850269	81.26337
GOTERM BP DIRECT	GO:0008064~regulation of actin polymerization or depolymerization	3	0.223881	0.085859	SSH2, ARI	1214	7	16792	5.927983	1	0.850269	81.26337
GOTERM BP DIRECT	GO:0072643~interferon-gamma secretion	3	0.223881	0.085859	BTN3A1, I	1214	7	16792	5.927983	1	0.850269	81.26337
GOTERM BP DIRECT	GO:0000578~embryonic axis specification	3	0.223881	0.085859	STIL, WN	1214	7	16792	5.927983	1	0.850269	81.26337
GOTERM BP DIRECT	GO:0071679~commissural neuron axon guidance	3	0.223881	0.085859	NRP1, VE	1214	7	16792	5.927983	1	0.850269	81.26337
GOTERM BP DIRECT	GO:0019885~antigen processing and presentation of endogenous peptide antiger	3	0.223881	0.085859	HLA-A, EI	1214	7	16792	5.927983	1	0.850269	81.26337
GOTERM BP DIRECT	GO:0048842~positive regulation of axon extension involved in axon guidance	3	0.223881	0.085859	NRP1, VE	1214	7	16792	5.927983	1	0.850269	81.26337
GOTERM BP DIRECT	GO:0001844~protein insertion into mitochondrial membrane involved in apopto	3	0.223881	0.085859	BAX, PMA	1214	7	16792	5.927983	1	0.850269	81.26337
GOTERM BP DIRECT	GO:0006029~proteoglycan metabolic process	3	0.223881	0.085859	BMP2, PP	1214	7	16792	5.927983	1	0.850269	81.26337
GOTERM BP DIRECT	GO:0048102~autophagic cell death	3	0.223881	0.085859	CDKN1B,	1214	7	16792	5.927983	1	0.850269	81.26337
GOTERM BP DIRECT	GO:0032287~peripheral nervous system myelin maintenance	3	0.223881	0.085859	MPP5, ND	1214	7	16792	5.927983	1	0.850269	81.26337
GOTERM BP DIRECT	GO:0060982~coronary artery morphogenesis	3	0.223881	0.085859	NRP1, TG	1214	7	16792	5.927983	1	0.850269	81.26337
GOTERM BP DIRECT	GO:0034616~response to laminar fluid shear stress	3	0.223881	0.085859	SMAD7, S	1214	7	16792	5.927983	1	0.850269	81.26337
GOTERM BP DIRECT	GO:0071455~cellular response to hyperoxia	3	0.223881	0.085859	CAV1, PP	1214	7	16792	5.927983	1	0.850269	81.26337
GOTERM BP DIRECT	GO:0051297~centrosome organization	6	0.447761	0.086023	CEP120, B	1214	33	16792	2.514902	1	0.84939	81.32616
GOTERM BP DIRECT	GO:1902042~negative regulation of extrinsic apoptotic signaling pathway via de	6	0.447761	0.086023	PEA15, AI	1214	33	16792	2.514902	1	0.84939	81.32616
GOTERM BP DIRECT	GO:0048538~thymus development	7	0.522388	0.086994	MAPK1, M	1214	43	16792	2.251714	1	0.85129	81.69273
GOTERM BP DIRECT	GO:0000184~nuclear-transcribed mRNA catabolic process, nonsense-mediated (	14	1.044776	0.088073	RPL17, PP	1214	119	16792	1.627289	1	0.853514	82.09234
GOTERM BP DIRECT	GO:0032436~positive regulation of proteasomal ubiquitin-dependent protein cat	9	0.671642	0.08879	CSNK1A1	1214	64	16792	1.945119	1	0.854489	82.35286
GOTERM BP DIRECT	GO:0042632~cholesterol homeostasis	9	0.671642	0.08879	NR1H2, A	1214	64	16792	1.945119	1	0.854489	82.35286
GOTERM BP DIRECT	GO:0008631~intrinsic apoptotic signaling pathway in response to oxidative stre	4	0.298507	0.089147	MAP3K5,	1214	15	16792	3.688523	1	0.854271	82.48152
GOTERM BP DIRECT	GO:0030330~DNA damage response, signal transduction by p53 class mediator	4	0.298507	0.089147	TP53, NDI	1214	15	16792	3.688523	1	0.854271	82.48152
GOTERM BP DIRECT	GO:0032793~positive regulation of CREB transcription factor activity	4	0.298507	0.089147	RPS6KA5,	1214	15	16792	3.688523	1	0.854271	82.48152
GOTERM BP DIRECT	GO:0035024~negative regulation of Rho protein signal transduction	4	0.298507	0.089147	DLC1, AR	1214	15	16792	3.688523	1	0.854271	82.48152
GOTERM BP DIRECT	GO:0006122~mitochondrial electron transport, ubiquinol to cytochrome c	4	0.298507	0.089147	UQCRC1,	1214	15	16792	3.688523	1	0.854271	82.48152
GOTERM BP DIRECT	GO:0007623~circadian rhythm	10	0.746269	0.090376	NONO, CI	1214	75	16792	1.844261	1	0.856897	82.91736
GOTERM BP DIRECT	GO:0045737~positive regulation of cyclin-dependent protein serine/threonine ki	5	0.373134	0.090702	CCND1, C	1214	24	16792	2.881658	1	0.856575	83.03131
GOTERM BP DIRECT	GO:0030010~establishment of cell polarity	5	0.373134	0.090702	UST, WW	1214	24	16792	2.881658	1	0.856575	83.03131
GOTERM BP DIRECT	GO:0043524~negative regulation of neuron apoptotic process	15	1.119403	0.095072	HSP90AB	1214	132	16792	1.571814	1	0.868707	84.48974
GOTERM BP DIRECT	GO:0001932~regulation of protein phosphorylation	6	0.447761	0.095165	CCDC88C	1214	34	16792	2.440934	1	0.867674	84.51925
GOTERM BP DIRECT	GO:0035019~somatic stem cell population maintenance	9	0.671642	0.095194	EPAS1, KI	1214	65	16792	1.915195	1	0.866456	84.52871
GOTERM BP DIRECT	GO:0060021~palate development	10	0.746269	0.096311	SGPL1, SU	1214	76	16792	1.819995	1	0.868447	84.88118
GOTERM BP DIRECT	GO:0019882~antigen processing and presentation	8	0.597015	0.099638	MICB, RA	1214	55	16792	2.011922	1	0.876573	85.88638

Category	Term	Count	%	PValue	Genes	List Total	Pop Hits	Pop Total	Fold Enricl	Bonferroni	Benjamini	FDR
GOTERM_CC_DIREC	GO:0005829~cytosol	357	26.64179	9.06E-20	TGOLN2,	1269	3315	18224	1.54656	6.35E-17	6.35E-17	1.37E-16
GOTERM_CC_DIREC	GO:0005654~nucleoplasm	306	22.83582	6.43E-18	TGOLN2,	1269	2784	18224	1.578463	4.50E-15	2.25E-15	9.70E-15
GOTERM_CC_DIREC	GO:0016020~membrane	228	17.01493	2.35E-10	LDHB, DY	1269	2200	18224	1.488313	1.65E-07	5.49E-08	3.55E-07
GOTERM_CC_DIREC	GO:0005634~nucleus	475	35.44776	7.46E-10	DLC1, RPl	1269	5415	18224	1.259729	5.22E-07	1.31E-07	1.13E-06
GOTERM_CC_DIREC	GO:0005737~cytoplasm	450	33.58209	3.67E-08	DLC1, LD	1269	5222	18224	1.237536	2.57E-05	5.14E-06	5.54E-05
GOTERM_CC_DIREC	GO:0005925~focal adhesion	53	3.955224	4.97E-06	PVR, KIF2	1269	391	18224	1.94662	0.003473	5.80E-04	0.007495
GOTERM_CC_DIREC	GO:0048471~perinuclear region of cytoplasm	74	5.522388	6.91E-06	SEPT2, NI	1269	621	18224	1.711284	0.004822	6.90E-04	0.010413
GOTERM_CC_DIREC	GO:0043231~intracellular membrane-bounded orga	67	5	1.59E-05	ACOX1, A	1269	558	18224	1.724339	0.011053	0.001388	0.023942
GOTERM_CC_DIREC	GO:0005794~Golgi apparatus	91	6.791045	6.91E-05	TGOLN2,	1269	863	18224	1.514303	0.0472	0.005358	0.10411
GOTERM_CC_DIREC	GO:0016605~PML body	19	1.41791	1.29E-04	MKNK2, T	1269	98	18224	2.784259	0.086107	0.008964	0.193797
GOTERM_CC_DIREC	GO:0005913~cell-cell adherens junction	42	3.134328	1.37E-04	PVR, HSP	1269	323	18224	1.867363	0.091701	0.008706	0.206996
GOTERM_CC_DIREC	GO:0005768~endosome	32	2.38806	2.05E-04	TGOLN2,	1269	225	18224	2.042441	0.133728	0.011892	0.308797
GOTERM_CC_DIREC	GO:0005769~early endosome	32	2.38806	2.81E-04	HMGB1, N	1269	229	18224	2.006765	0.17886	0.015044	0.423645
GOTERM_CC_DIREC	GO:0035068~micro-ribonucleoprotein complex	5	0.373134	3.12E-04	AGO1, AC	1269	6	18224	11.96743	0.196382	0.015495	0.469905
GOTERM_CC_DIREC	GO:0034045~pre-autophagosomal structure membr	7	0.522388	4.89E-04	ATG2B, U	1269	16	18224	6.2829	0.289956	0.02257	0.735004
GOTERM_CC_DIREC	GO:0016442~RISC complex	6	0.447761	5.24E-04	DICER1, A	1269	11	18224	7.833226	0.307197	0.022677	0.787557
GOTERM_CC_DIREC	GO:0015629~actin cytoskeleton	30	2.238806	5.82E-04	ABLIM1,	1269	218	18224	1.976273	0.334604	0.023678	0.873795
GOTERM_CC_DIREC	GO:0005741~mitochondrial outer membrane	23	1.716418	6.39E-04	MAVS, DN	1269	149	18224	2.216785	0.360846	0.024561	0.959684
GOTERM_CC_DIREC	GO:0070578~RISC-loading complex	5	0.373134	6.88E-04	DICER1, A	1269	7	18224	10.2578	0.382486	0.025052	1.03315
GOTERM_CC_DIREC	GO:0000932~cytoplasmic mRNA processing body	15	1.119403	8.87E-04	PAN3, CN	1269	78	18224	2.761714	0.462825	0.030594	1.329873
GOTERM_CC_DIREC	GO:0017053~transcriptional repressor complex	12	0.895522	0.001237	TBL1XR1,	1269	55	18224	3.13329	0.579459	0.040409	1.848844
GOTERM_CC_DIREC	GO:0000139~Golgi membrane	62	4.626866	0.001269	CDIPT, PI	1269	591	18224	1.50656	0.588937	0.039604	1.89703
GOTERM_CC_DIREC	GO:0005730~nucleolus	82	6.119403	0.002929	ACOX1, Z	1269	857	18224	1.37409	0.871699	0.085408	4.327316
GOTERM_CC_DIREC	GO:0031083~BLOC-1 complex	6	0.447761	0.003697	BLOC1S4,	1269	16	18224	5.385343	0.925156	0.102386	5.431802
GOTERM_CC_DIREC	GO:0005905~clathrin-coated pit	11	0.820896	0.004229	APP, AP1S	1269	55	18224	2.872183	0.948529	0.111899	6.191501
GOTERM_CC_DIREC	GO:0005776~autophagosome	12	0.895522	0.004336	ORAI1, TI	1269	64	18224	2.692671	0.952243	0.110402	6.342737
GOTERM_CC_DIREC	GO:0043209~myelin sheath	21	1.567164	0.004399	LDHB, GD	1269	152	18224	1.984074	0.954315	0.108006	6.432158
GOTERM_CC_DIREC	GO:0032154~cleavage furrow	10	0.746269	0.004567	RAB11FIP	1269	47	18224	3.055514	0.959398	0.108122	6.669642
GOTERM_CC_DIREC	GO:0032588~trans-Golgi network membrane	14	1.044776	0.004648	TGOLN2,	1269	83	18224	2.422323	0.961647	0.106354	6.784151
GOTERM_CC_DIREC	GO:0005813~centrosome	45	3.358209	0.005606	CEP97, KI	1269	426	18224	1.516998	0.980456	0.122931	8.12823
GOTERM_CC_DIREC	GO:0016604~nuclear body	8	0.597015	0.006691	SUMO1, N	1269	33	18224	3.481434	0.990902	0.14067	9.629148
GOTERM_CC_DIREC	GO:0015630~microtubule cytoskeleton	19	1.41791	0.006774	HYPK, CK	1269	137	18224	1.99166	0.991414	0.138151	9.741855
GOTERM_CC_DIREC	GO:0030496~midbody	18	1.343284	0.008106	KIF23, TR	1269	129	18224	2.003848	0.996645	0.158563	11.55065
GOTERM_CC_DIREC	GO:0043234~protein complex	43	3.208955	0.008338	CEP97, OI	1269	412	18224	1.498833	0.997151	0.158337	11.86172
GOTERM_CC_DIREC	GO:1904115~axon cytoplasm	8	0.597015	0.009331	BLOC1S4,	1269	35	18224	3.282495	0.998588	0.170976	13.18452
GOTERM_CC_DIREC	GO:0010008~endosome membrane	23	1.716418	0.009633	ATP6V0E	1269	185	18224	1.785411	0.998859	0.171562	13.58194
GOTERM_CC_DIREC	GO:0032587~ruffle membrane	13	0.970149	0.010944	DLC1, SPI	1269	82	18224	2.27673	0.999548	0.18794	15.29071
GOTERM_CC_DIREC	GO:0005802~trans-Golgi network	18	1.343284	0.013414	TGOLN2,	1269	136	18224	1.900709	0.999922	0.220244	18.42623
GOTERM_CC_DIREC	GO:0031252~cell leading edge	8	0.597015	0.016771	GBF1, SLI	1269	39	18224	2.945829	0.999993	0.261817	22.5127
GOTERM_CC_DIREC	GO:0005662~DNA replication factor A complex	5	0.373134	0.017134	RPA2, PLI	1269	15	18224	4.786971	0.999994	0.260984	22.94293
GOTERM_CC_DIREC	GO:0000151~ubiquitin ligase complex	15	1.119403	0.017549	RNF216, T	1269	108	18224	1.994571	0.999996	0.260862	23.43217
GOTERM_CC_DIREC	GO:0030670~phagocytic vesicle membrane	10	0.746269	0.020023	ATP6V0E	1269	59	18224	2.434053	0.999999	0.286166	26.28952
GOTERM_CC_DIREC	GO:0005819~spindle	16	1.19403	0.020774	KIF23, MA	1269	121	18224	1.898964	1	0.289473	27.13703
GOTERM_CC_DIREC	GO:0030424~axon	25	1.865672	0.021108	TNFRSF21	1269	222	18224	1.61722	1	0.2878	27.51002
GOTERM_CC_DIREC	GO:0042470~melanosome	14	1.044776	0.022765	HSP90AB	1269	101	18224	1.990622	1	0.30108	29.33943
GOTERM_CC_DIREC	GO:0005801~cis-Golgi network	8	0.597015	0.024572	MPPE1, R	1269	42	18224	2.735412	1	0.315179	31.28447
GOTERM_CC_DIREC	GO:0005643~nuclear pore	11	0.820896	0.026821	MAD1L1,	1269	72	18224	2.194029	1	0.332963	33.63451
GOTERM_CC_DIREC	GO:0000790~nuclear chromatin	22	1.641791	0.027766	POLR3G,	1269	193	18224	1.636995	1	0.336784	34.60063
GOTERM_CC_DIREC	GO:0000813~ESCRT I complex	4	0.298507	0.027857	TSG101, V	1269	10	18224	5.744366	1	0.332094	34.69266
GOTERM_CC_DIREC	GO:0031901~early endosome membrane	15	1.119403	0.030553	CAV1, RA	1269	116	18224	1.857015	1	0.352358	37.3714
GOTERM_CC_DIREC	GO:0005901~caveola	10	0.746269	0.035254	DLC1, MA	1269	65	18224	2.209371	1	0.388976	41.79828
GOTERM_CC_DIREC	GO:0009986~cell surface	50	3.731343	0.03531	PVR, HSP	1269	542	18224	1.324808	1	0.38364	41.84905
GOTERM_CC_DIREC	GO:0031985~Golgi cisterna	4	0.298507	0.036362	RAB30, R	1269	11	18224	5.222151	1	0.386881	42.79749
GOTERM_CC_DIREC	GO:0042612~MHC class I protein complex	4	0.298507	0.036362	HLA-A, H	1269	11	18224	5.222151	1	0.386881	42.79749
GOTERM_CC_DIREC	GO:1990316~ATG1/ULK1 kinase complex	3	0.223881	0.041998	ULK1, RB	1269	5	18224	8.616548	1	0.426604	47.64048
GOTERM_CC_DIREC	GO:0005874~microtubule	31	2.313433	0.043626	KIF23, DY	1269	311	18224	1.431474	1	0.433181	48.96661
GOTERM_CC_DIREC	GO:0005844~polysome	7	0.522388	0.045697	EIF4H, EI	1269	38	18224	2.645432	1	0.442713	50.60796
GOTERM_CC_DIREC	GO:0005667~transcription factor complex	21	1.567164	0.048124	E2F2, E2F	1269	193	18224	1.562587	1	0.454302	52.4687
GOTERM_CC_DIREC	GO:0000792~heterochromatin	5	0.373134	0.053997	SUMO1, M	1269	21	18224	3.419265	1	0.488261	56.70423
GOTERM_CC_DIREC	GO:0005721~pericentric heterochromatin	4	0.298507	0.056834	DNMT1, C	1269	13	18224	4.418743	1	0.500539	58.62188
GOTERM_CC_DIREC	GO:0005739~mitochondrion	108	8.059701	0.059609	HCCS, LD	1269	1331	18224	1.165273	1	0.511802	60.42036
GOTERM_CC_DIREC	GO:0001940~male pronucleus	3	0.223881	0.060132	TET3, CB	1269	6	18224	7.180457	1	0.50917	60.75077
GOTERM_CC_DIREC	GO:0035749~myelin sheath adaxonal region	3	0.223881	0.060132	STX4, MP	1269	6	18224	7.180457	1	0.50917	60.75077
GOTERM_CC_DIREC	GO:0070062~extracellular exosome	216	16.1194	0.062468	PVR, LDH	1269	2811	18224	1.103507	1	0.517262	62.19666
GOTERM_CC_DIREC	GO:0005720~nuclear heterochromatin	5	0.373134	0.062499	CBX3, FO	1269	22	18224	3.263844	1	0.511824	62.21512
GOTERM_CC_DIREC	GO:0072686~mitotic spindle	7	0.522388	0.062541	KIF23, MA	1269	41	18224	2.451863	1	0.506568	62.24091
GOTERM_CC_DIREC	GO:0005765~lysosomal membrane	27	2.014925	0.066776	HSP90AB	1269	274	18224	1.415127	1	0.524917	64.7332
GOTERM_CC_DIREC	GO:0000974~Prp19 complex	4	0.298507	0.068712	PLRG1, IS	1269	14	18224	4.103118	1	0.529994	65.82035
GOTERM_CC_DIREC	GO:0090575~RNA polymerase II transcription fact	6	0.447761	0.075873	NONO, HI	1269	33	18224	2.611075	1	0.561495	69.57608
GOTERM_CC_DIREC	GO:0055037~recycling endosome	13	0.970149	0.080372	AP1G1, R	1269	110	18224	1.697199	1	0.577894	71.73482
GOTERM_CC_DIREC	GO:0000779~condensed chromosome, centromeric	3	0.223881	0.080381	CBX3, AU	1269	7	18224	6.154677	1	0.572629	71.73917
GOTERM_CC_DIREC	GO:0045121~membrane raft	21	1.567164	0.082077	LDHB, CA	1269	206	18224	1.463977	1	0.575322	72.51525
GOTERM_CC_DIREC	GO:0045171~intercellular bridge	7	0.522388	0.082593	KIF23, SP	1269	44	18224	2.284691	1	0.572543	72.74725
GOTERM_CC_DIREC	GO:0055038~recycling endosome membrane	7	0.522388	0.082593	RAB11FIP	1269	44	18224	2.284691	1	0.572543	72.74725
GOTERM_CC_DIREC	GO:0031463~Cul3-RING ubiquitin ligase complex	9	0.671642	0.092503	BACH1, II	1269	67	18224	1.929078	1	0.610808	76.8643
GOTERM_CC_DIREC	GO:0005814~centriole	13	0.970149	0.093818	CEP104, S	1269	113	18224	1.652141	1	0.611192	77.36502
GOTERM_CC_DIREC	GO:0030014~CCR4-NOT complex	4	0.298507	0.095451	PATL1, CI	1269	16	18224	3.590229	1	0.612859	77.97245
GOTERM_CC_DIREC	GO:0035098~ESC/E(Z) complex	4	0.298507	0.095451	TRIM37, J	1269	16	18224	3.590229	1	0.612859	77.97245
GOTERM_CC_DIREC	GO:0031410~cytoplasmic vesicle	23	1.716418	0.095672	ORAI1, C	1269	235	18224	1.405536	1	0.608821	78.05335
GOTERM_CC_DIREC	GO:0012505~endomembrane system	13	0.970149	0.098587	PARD3, T	1269	114	18224	1.637648	1	0.615565	79.09643
GOTERM_CC_DIREC	GO:0005793~endoplasmic reticulum-Golgi interme	9	0.671642	0.098802	HMGB1, T	1269	68	18224	1.900709	1	0.611603	79.17128
GOTERM_CC_DIREC	GO:0030659~cytoplasmic vesicle membrane	14	1.044776	0.099337	YWHAZ,	1269	126	18224	1.595657	1	0.608955	79.35697

Category	Term	Count	%	PValue	Genes	List Total	Pop Hits	Pop Total	Fold Enric	Bonferroni	Benjamini	FDR
GOTERM	GO:0005515~protein binding	804	60	1.79E-24	LDHB, CI	1221	8785	16881	1.26531	2.22E-21	2.22E-21	2.90E-21
GOTERM	GO:0031625~ubiquitin protein ligase binding	48	3.58209	1.01E-07	TRIOBP, '	1221	287	16881	2.312288	1.26E-04	6.29E-05	1.64E-04
GOTERM	GO:0019901~protein kinase binding	54	4.029851	2.09E-06	E2F1, HSI	1221	376	16881	1.985585	0.002598	8.67E-04	0.003393
GOTERM	GO:0004672~protein kinase activity	52	3.880597	2.58E-06	CDK19, N	1221	359	16881	2.002587	0.003212	8.04E-04	0.004197
GOTERM	GO:0044822~poly(A) RNA binding	119	8.880597	2.50E-05	MKRN1, I	1221	1129	16881	1.457255	0.030688	0.006214	0.040651
GOTERM	GO:0004674~protein serine/threonine kinase activi	50	3.731343	4.18E-05	CDK19, N	1221	376	16881	1.838504	0.050744	0.008642	0.06791
GOTERM	GO:0008134~transcription factor binding	39	2.910448	1.69E-04	E2F1, E2F	1221	284	16881	1.898579	0.189492	0.029568	0.273688
GOTERM	GO:0003700~transcription factor activity, sequence	100	7.462687	1.95E-04	REST, RO	1221	961	16881	1.438663	0.215942	0.029951	0.316841
GOTERM	GO:0003677~DNA binding	158	11.79104	3.16E-04	BBX, INO	1221	1674	16881	1.304921	0.325188	0.042761	0.511764
GOTERM	GO:0042826~histone deacetylase binding	19	1.41791	3.44E-04	HSP90AB	1221	102	16881	2.575348	0.348678	0.041969	0.557734
GOTERM	GO:0046872~metal ion binding	189	14.10448	4.07E-04	HCCS, MI	1221	2069	16881	1.262943	0.397438	0.045007	0.658622
GOTERM	GO:0004842~ubiquitin-protein transferase activity	42	3.134328	4.43E-04	MKRN1, I	1221	329	16881	1.764964	0.423709	0.04489	0.716375
GOTERM	GO:0019903~protein phosphatase binding	14	1.044776	4.80E-04	PARD3, H	1221	63	16881	3.072345	0.450066	0.044955	0.776987
GOTERM	GO:0046332~SMAD binding	11	0.820896	7.95E-04	ACVR1B,	1221	43	16881	3.536769	0.62867	0.068316	1.283985
GOTERM	GO:0005524~ATP binding	140	10.44776	0.001029	DYNC1LI	1221	1495	16881	1.294701	0.722319	0.081872	1.65751
GOTERM	GO:0004702~receptor signaling protein serine/thre	12	0.895522	0.00122	PAK6, AC	1221	53	16881	3.130314	0.781205	0.090605	1.962789
GOTERM	GO:0042802~identical protein binding	77	5.746269	0.001606	CEP72, LI	1221	749	16881	1.421319	0.864743	0.111021	2.575953
GOTERM	GO:0001047~core promoter binding	13	0.970149	0.001886	E2F1, NOI	1221	64	16881	2.808315	0.904651	0.122402	3.019274
GOTERM	GO:0098641~cadherin binding involved in cell-cell	36	2.686567	0.001948	HSP90AB	1221	290	16881	1.716276	0.911785	0.119961	3.117614
GOTERM	GO:0003707~steroid hormone receptor activity	12	0.895522	0.001953	NR1H2, P.	1221	56	16881	2.962618	0.912324	0.114591	3.125361
GOTERM	GO:0003682~chromatin binding	45	3.358209	0.002277	MORF4L1	1221	391	16881	1.591176	0.941466	0.126415	3.634591
GOTERM	GO:0000981~RNA polymerase II transcription fact	24	1.791045	0.002862	ZMYM1, I	1221	171	16881	1.940428	0.971802	0.149732	4.548332
GOTERM	GO:0003727~single-stranded RNA binding	10	0.746269	0.003134	HMGB1, I	1221	43	16881	3.215245	0.979929	0.15628	4.970748
GOTERM	GO:0004709~MAP kinase kinase kinase activity	7	0.522388	0.003838	MAP3K5,	1221	22	16881	4.39904	0.991671	0.18086	6.054782
GOTERM	GO:0070411~I-SMAD binding	5	0.373134	0.005934	SMAD7, S	1221	11	16881	6.284342	0.999394	0.25649	9.21291
GOTERM	GO:0043130~ubiquitin binding	13	0.970149	0.009856	ADRM1, I	1221	78	16881	2.304259	0.999996	0.377667	14.85877
GOTERM	GO:0001046~core promoter sequence-specific DN.	9	0.671642	0.010889	USP3, KLI	1221	43	16881	2.89372	0.999999	0.396422	16.29094
GOTERM	GO:0004725~protein tyrosine phosphatase activity	15	1.119403	0.012626	SSH2, PTI	1221	100	16881	2.073833	1	0.431622	18.64571
GOTERM	GO:0042803~protein homodimerization activity	70	5.223881	0.012794	PVR, GRP	1221	730	16881	1.325738	1	0.424667	18.87069
GOTERM	GO:0004879~RNA polymerase II transcription fact	8	0.597015	0.01326	NR1H2, P.	1221	36	16881	3.072345	1	0.425342	19.49063
GOTERM	GO:0017048~Rho GTPase binding	7	0.522388	0.013372	FMNL2, F	1221	28	16881	3.456388	1	0.417639	19.63874
GOTERM	GO:0035035~histone acetyltransferase binding	7	0.522388	0.013372	ZBTB7A,	1221	28	16881	3.456388	1	0.417639	19.63874
GOTERM	GO:0002134~UTP binding	3	0.223881	0.014904	HSP90AB	1221	3	16881	13.82555	1	0.442461	21.64133
GOTERM	GO:0017098~sulfonylurea receptor binding	3	0.223881	0.014904	HSP90AB	1221	3	16881	13.82555	1	0.442461	21.64133
GOTERM	GO:0004713~protein tyrosine kinase activity	18	1.343284	0.015343	TWF1, NF	1221	133	16881	1.871127	1	0.441971	22.20684
GOTERM	GO:0019904~protein domain specific binding	25	1.865672	0.01551	HMGB2, '	1221	208	16881	1.661725	1	0.435816	22.42001
GOTERM	GO:0035197~siRNA binding	4	0.298507	0.016007	DICER1, I	1221	8	16881	6.912776	1	0.436725	23.05378
GOTERM	GO:0019899~enzyme binding	36	2.686567	0.016436	CDK19, A	1221	333	16881	1.494654	1	0.43625	23.59753
GOTERM	GO:0001228~transcriptional activator activity, RN.	14	1.044776	0.018935	MAFB, TI	1221	95	16881	2.03745	1	0.474423	26.68995
GOTERM	GO:0001077~transcriptional activator activity, RN.	27	2.014925	0.020845	CAMTA1,	1221	236	16881	1.581737	1	0.49851	28.97359
GOTERM	GO:0004675~transmembrane receptor protein serin	4	0.298507	0.022737	ACVR1B,	1221	9	16881	6.14469	1	0.520112	31.16946
GOTERM	GO:0034713~type I transforming growth factor bet	4	0.298507	0.022737	SMAD7, S	1221	9	16881	6.14469	1	0.520112	31.16946
GOTERM	GO:0032403~protein complex binding	24	1.791045	0.024821	TWF1, UC	1221	206	16881	1.610744	1	0.542651	33.51548
GOTERM	GO:0003723~RNA binding	53	3.955224	0.02564	RALY, RF	1221	547	16881	1.339587	1	0.545581	34.41634
GOTERM	GO:0051082~unfolded protein binding	15	1.119403	0.027043	PTGES3, I	1221	110	16881	1.885303	1	0.556325	35.9331
GOTERM	GO:0051434~BH3 domain binding	3	0.223881	0.028383	MCL1, BA	1221	4	16881	10.36916	1	0.56555	37.35139
GOTERM	GO:0004843~thiol-dependent ubiquitin-specific pro	12	0.895522	0.028485	OTUD4, C	1221	80	16881	2.073833	1	0.558559	37.4586
GOTERM	GO:0051015~actin filament binding	17	1.268657	0.028644	TRIOBP, I	1221	132	16881	1.780564	1	0.552489	37.6241
GOTERM	GO:0003725~double-stranded RNA binding	10	0.746269	0.030263	HSP90AB	1221	61	16881	2.266484	1	0.564703	39.29145
GOTERM	GO:0003743~translation initiation factor activity	10	0.746269	0.030263	EIF4G2, R	1221	61	16881	2.266484	1	0.564703	39.29145
GOTERM	GO:0005086~ARF guanyl-nucleotide exchange fac	6	0.447761	0.03087	GBF1, FN	1221	25	16881	3.318133	1	0.564215	39.90524
GOTERM	GO:0017137~Rab GTPase binding	17	1.268657	0.032366	TBC1D2B	1221	134	16881	1.753988	1	0.574022	41.39413
GOTERM	GO:0003684~damaged DNA binding	10	0.746269	0.036368	HMGB1, I	1221	63	16881	2.194532	1	0.609866	45.20931
GOTERM	GO:0008301~DNA binding, bending	5	0.373134	0.036727	TFAM, HI	1221	18	16881	3.840431	1	0.606122	45.53974
GOTERM	GO:0000978~RNA polymerase II core promoter pr	36	2.686567	0.037871	ZNF805, F	1221	355	16881	1.402028	1	0.610336	46.5813
GOTERM	GO:0042056~chemoattractant activity	6	0.447761	0.041665	HMGB1, I	1221	27	16881	3.072345	1	0.639022	49.90126
GOTERM	GO:0051721~protein phosphatase 2A binding	6	0.447761	0.041665	EIF4EBP1	1221	27	16881	3.072345	1	0.639022	49.90126
GOTERM	GO:0005525~GTP binding	38	2.835821	0.04488	HSP90AB	1221	384	16881	1.368154	1	0.65994	52.56162
GOTERM	GO:0015057~thrombin receptor activity	3	0.223881	0.045058	F2RL3, F2	1221	5	16881	8.295332	1	0.654571	52.70536
GOTERM	GO:0043621~protein self-association	8	0.597015	0.045659	ATXN1, C	1221	46	16881	2.404444	1	0.652812	53.18638
GOTERM	GO:0031267~small GTPase binding	5	0.373134	0.051891	GDI2, RC	1221	20	16881	3.456388	1	0.694147	57.91149
GOTERM	GO:0005102~receptor binding	35	2.61194	0.052863	PVR, GLC	1221	353	16881	1.370806	1	0.694644	58.60702
GOTERM	GO:0042169~SH2 domain binding	6	0.447761	0.054474	DLC1, LA	1221	29	16881	2.860459	1	0.69952	59.73598
GOTERM	GO:0003713~transcription coactivator activity	26	1.940299	0.057217	TDRD3, P	1221	248	16881	1.449453	1	0.711564	61.59115
GOTERM	GO:0044212~transcription regulatory region DNA	23	1.716418	0.057441	TBL1XR1	1221	213	16881	1.4929	1	0.70698	61.73972
GOTERM	GO:0001227~transcriptional repressor activity, RN	9	0.671642	0.060781	ZNF280B,	1221	59	16881	2.108983	1	0.721916	63.88281
GOTERM	GO:1990226~histone methyltransferase binding	3	0.223881	0.064397	HSP90AB	1221	6	16881	6.912776	1	0.737277	66.07624
GOTERM	GO:0032947~protein complex scaffold	7	0.522388	0.065667	ISCU, CA'	1221	40	16881	2.419472	1	0.738748	66.81606
GOTERM	GO:0003697~single-stranded DNA binding	12	0.895522	0.071705	PRIM1, HI	1221	93	16881	1.783942	1	0.764824	70.1326
GOTERM	GO:0046934~phosphatidylinositol-4,5-bisphosphat	9	0.671642	0.07692	NRG4, FG	1221	62	16881	2.006935	1	0.784127	72.7441
GOTERM	GO:0046982~protein heterodimerization activity	43	3.208955	0.079132	CAV1, PP	1221	465	16881	1.278492	1	0.788828	73.78545
GOTERM	GO:0008270~zinc ion binding	98	7.313433	0.083019	MKRN1, I	1221	1169	16881	1.159028	1	0.800208	75.52594
GOTERM	GO:0043565~sequence-specific DNA binding	47	3.507463	0.085697	E2F1, BA(	1221	518	16881	1.254442	1	0.806089	76.66158
GOTERM	GO:0004703~G-protein coupled receptor kinase ac	3	0.223881	0.085929	GRK7, GF	1221	7	16881	5.925237	1	0.802331	76.75757
GOTERM	GO:0001222~transcription corepressor binding	3	0.223881	0.085929	HDGF, RC	1221	7	16881	5.925237	1	0.802331	76.75757
GOTERM	GO:0005114~type II transforming growth factor be	3	0.223881	0.085929	TGFBR1,	1221	7	16881	5.925237	1	0.802331	76.75757
GOTERM	GO:0005161~platelet-derived growth factor recept	4	0.298507	0.089245	PDGFB, I'	1221	15	16881	3.686814	1	0.810362	78.08968
GOTERM	GO:0047485~protein N-terminus binding	12	0.895522	0.090623	MORF4L1	1221	97	16881	1.710378	1	0.810955	78.62179
GOTERM	GO:0030742~GTP-dependent protein binding	5	0.373134	0.090824	DNM1L, F	1221	24	16881	2.880324	1	0.807269	78.69847
GOTERM	GO:0019003~GDP binding	8	0.597015	0.092574	RAP2C, R	1221	54	16881	2.04823	1	0.809245	79.35471
GOTERM	GO:0001948~glycoprotein binding	9	0.671642	0.095392	HSP90AB	1221	65	16881	1.914307	1	0.814869	80.37148
