# Supplementary material for: Integrated MicroRNA Expression Profile Reveals Dysregulated miR-20a-5p and miR-200a-3p in Liver Fibrosis
Source: Biomed Res Int. 2021 Jun 8;2021:9583932. doi: 10.1155/2021/9583932 (PMC8218919; doi:10.1155/2021/9583932)
Supplement: Supplementary 4 — Table S4: KEGG pathway enrichment analysis for selected DEMs. [file 9583932.f4.pdf]

Category	Term	Count	%	PValue	Genes	List Total	Pop Hits	Pop Total	Fold Enricl	Bonferroni	Benjamini	FDR
KEGG_PATHWAY	hsa05206:MicroRNAs in cancer	43	9.287257	3.45E-17	BMI1, E2F3, CYP1B1, MCL1, GRB2, EZH2, DICER1, ZE	225	286	6879	4.59669	7.49E-15	7.49E-15	4.38E-14
KEGG_PATHWAY	hsa05215:Prostate cancer	21	4.535637	3.34E-12	EGFR, E2F3, GRB2, CREB1, TP53, FOXO1, CREB5, PTE	225	88	6879	7.295909	7.25E-10	3.63E-10	4.25E-09
KEGG_PATHWAY	hsa04068:FoxO signaling pathway	24	5.183585	3.64E-11	EGFR, ATG12, GRB2, SMAD4, SMAD3, FOXO1, SMAD	225	134	6879	5.475821	7.90E-09	2.63E-09	4.62E-08
KEGG_PATHWAY	hsa05200:Pathways in cancer	41	8.855292	5.62E-11	GNA13, E2F3, WNT16, XIAP, GRB2, FGF9, ADCY6, MI	225	393	6879	3.189584	1.22E-08	3.05E-09	7.14E-08
KEGG_PATHWAY	hsa05205:Proteoglycans in cancer	27	5.831533	1.07E-09	WNT16, GRB2, PDCD4, CTNNB1, TGFB2, WNT1, IGF1	225	200	6879	4.1274	2.32E-07	4.65E-08	1.36E-06
KEGG_PATHWAY	hsa04722:Neurotrophin signaling pathway	20	4.319654	8.14E-09	GRB2, TP53, FOXO3, IRS1, PDPK1, BDNF, CRKL, KRA	225	120	6879	5.095556	1.77E-06	2.94E-07	1.03E-05
KEGG_PATHWAY	hsa05166:HTLV-I infection	28	6.047516	4.20E-08	WNT16, E2F3, XIAP, ADCY6, CHEK2, SRF, ATF1, CTN	225	254	6879	3.370289	9.12E-06	1.30E-06	5.34E-05
KEGG_PATHWAY	hsa04151:PI3K-Akt signaling pathway	33	7.12743	5.75E-08	PHLPP1, MCL1, FGF9, GRB2, FOXO3, PTEN, CCNE2, I	225	345	6879	2.924406	1.25E-05	1.56E-06	7.31E-05
KEGG_PATHWAY	hsa04510:Focal adhesion	24	5.183585	1.87E-07	EGFR, FLT1, XIAP, ACTN4, ROCK2, GRB2, HGF, PTEN	225	206	6879	3.561942	4.06E-05	4.51E-06	2.38E-04
KEGG_PATHWAY	hsa04520:Adherens junction	14	3.023758	3.56E-07	EGFR, WASF3, ACTN4, SMAD4, SMAD3, SMAD2, SNA	225	71	6879	6.028545	7.73E-05	7.73E-06	4.52E-04
KEGG_PATHWAY	hsa05210:Colorectal cancer	13	2.807775	5.42E-07	KRAS, JUN, BCL2, GSK3B, TP53, SMAD4, RHOA, SMA	225	62	6879	6.410538	1.18E-04	1.07E-05	6.89E-04
KEGG_PATHWAY	hsa05213:Endometrial cancer	12	2.591793	6.36E-07	EGFR, PDPK1, KRAS, GRB2, GSK3B, TP53, FOXO3, PT	225	52	6879	7.055385	1.38E-04	1.15E-05	8.08E-04
KEGG_PATHWAY	hsa05161:Hepatitis B	19	4.103672	9.08E-07	E2F3, GRB2, TBK1, CREB1, TP53, SMAD4, CDK6, CRE	225	145	6879	4.006161	1.97E-04	1.52E-05	0.001153
KEGG_PATHWAY	hsa04115:p53 signaling pathway	13	2.807775	1.30E-06	ZMAT3, TP53, CDK6, CHEK2, PMAIP1, SESN2, PTEN,	225	67	6879	5.932139	2.83E-04	2.02E-05	0.001655
KEGG_PATHWAY	hsa05212:Pancreatic cancer	12	2.591793	6.49E-06	EGFR, E2F3, KRAS, VEGFA, TP53, SMAD4, SMAD3, SI	225	65	6879	5.644308	0.001407	9.39E-05	0.008241
KEGG_PATHWAY	hsa04110:Cell cycle	16	3.455724	1.05E-05	E2F3, CDC14B, TP53, SMAD4, SMAD3, CDK6, SMAD2	225	124	6879	3.944946	0.002269	1.42E-04	0.013295
KEGG_PATHWAY	hsa05220:Chronic myeloid leukemia	12	2.591793	1.79E-05	CDKN1A, E2F3, CDKN1B, KRAS, CRKL, GRB2, TP53, S	225	72	6879	5.095556	0.003882	2.29E-04	0.022764
KEGG_PATHWAY	hsa04012:ErbB signaling pathway	13	2.807775	2.17E-05	EGFR, GRB2, BTC, CDKN1A, CRKL, KRAS, CDKN1B, I	225	87	6879	4.568429	0.004692	2.61E-04	0.027522
KEGG_PATHWAY	hsa05214:Glioma	11	2.37581	4.08E-05	EGFR, IGF1R, CDKN1A, E2F3, KRAS, PLCG1, GRB2, TI	225	65	6879	5.173949	0.008814	4.66E-04	0.051806
KEGG_PATHWAY	hsa04014:Ras signaling pathway	21	4.535637	4.18E-05	EGFR, PLD1, FLT1, RAB5B, FGF9, GRB2, TBK1, HGF, E	225	226	6879	2.840885	0.009028	4.53E-04	0.053069
KEGG_PATHWAY	hsa04066:HIF-1 signaling pathway	13	2.807775	5.89E-05	EGFR, FLT1, PFKFB3, MKNK2, STAT3, IGF1R, CDKN1	225	96	6879	4.140139	0.012706	6.09E-04	0.074819
KEGG_PATHWAY	hsa04919:Thyroid hormone signaling pathwa	14	3.023758	8.42E-05	NOTCH2, PDPK1, NOTCH1, KRAS, NCOA2, EP300, TH	225	115	6879	3.721971	0.018098	8.30E-04	0.106843
KEGG_PATHWAY	hsa05218:Melanoma	11	2.37581	8.87E-05	EGFR, IGF1R, CDKN1A, E2F3, KRAS, FGF9, MITF, TP5	225	71	6879	4.736714	0.019064	8.37E-04	0.1126
KEGG_PATHWAY	hsa04390:Hippo signaling pathway	16	3.455724	1.08E-04	PARD6B, WNT1, WNT16, CCND2, GSK3B, SMAD4, SM	225	151	6879	3.239558	0.023236	9.79E-04	0.137516
KEGG_PATHWAY	hsa05203:Viral carcinogenesis	19	4.103672	1.13E-04	VAC14, ACTN4, GRB2, CREB1, TP53, CDK6, CREB5, P	225	205	6879	2.833626	0.024307	9.84E-04	0.143925
KEGG_PATHWAY	hsa04015:Rap1 signaling pathway	19	4.103672	1.54E-04	EGFR, PARD6B, FLT1, FGF9, ADCY6, HGF, EPHA2, KD	225	210	6879	2.766159	0.032943	0.001288	0.195874
KEGG_PATHWAY	hsa04550:Signaling pathways regulating plu	15	3.239741	1.71E-04	BMI1, WNT16, GRB2, SMAD4, SMAD3, SMAD2, STAT	225	140	6879	3.275714	0.036524	0.001377	0.217545
KEGG_PATHWAY	hsa05211:Renal cell carcinoma	10	2.159827	2.58E-04	KRAS, CRKL, EP300, PAK2, GRB2, ETS1, JUN, VEGFA	225	66	6879	4.632323	0.054379	0.001995	0.326732
KEGG_PATHWAY	hsa05223:Non-small cell lung cancer	9	1.943844	4.11E-04	EGFR, PDPK1, E2F3, KRAS, PLCG1, GRB2, TP53, CDK	225	56	6879	4.913571	0.085359	0.003072	0.520873
KEGG_PATHWAY	hsa04916:Melanogenesis	11	2.37581	0.00146	WNT1, WNT16, KRAS, EP300, CREB1, GSK3B, ADCY6	225	100	6879	3.363067	0.271694	0.010512	1.838524
KEGG_PATHWAY	hsa04660:T cell receptor signaling pathway	11	2.37581	0.00146	PDPK1, KRAS, PAK2, PLCG1, GRB2, MAPK14, JUN, GS	225	100	6879	3.363067	0.271694	0.010512	1.838524
KEGG_PATHWAY	hsa04320:Dorso-ventral axis formation	6	1.295896	0.001572	EGFR, NOTCH2, NOTCH1, KRAS, GRB2, ETS1	225	27	6879	6.794074	0.289273	0.010955	1.978809
KEGG_PATHWAY	hsa05222:Small cell lung cancer	10	2.159827	0.001678	CCNE2, E2F3, CDKN1B, XIAP, BCL2, TP53, CDK6, IKB	225	85	6879	3.596863	0.305456	0.011326	2.110865
KEGG_PATHWAY	hsa04310:Wnt signaling pathway	13	2.807775	0.001724	WNT1, WNT16, EP300, CCND2, ROCK2, JUN, GSK3B,	225	138	6879	2.880097	0.312343	0.011283	2.167945
KEGG_PATHWAY	hsa05219:Bladder cancer	7	1.511879	0.001929	EGFR, CDKN1A, E2F3, KRAS, VEGFA, TP53, THBS1	225	41	6879	5.219837	0.342348	0.01225	2.423085
KEGG_PATHWAY	hsa04931:Insulin resistance	11	2.37581	0.002605	PDPK1, CREB1, GSK3B, FOXO1, CREB5, PRKAA2, OG	225	108	6879	3.113951	0.43221	0.016041	3.258615
KEGG_PATHWAY	hsa04810:Regulation of actin cytoskeleton	16	3.455724	0.003434	EGFR, GNA13, ACTN4, ROCK2, FGF9, ABI2, PFN1, CR	225	210	6879	2.329397	0.526006	0.020524	4.275605
KEGG_PATHWAY	hsa04010:MAPK signaling pathway	18	3.887689	0.003526	EGFR, GRB2, CACNG8, FGF9, MKNK2, TP53, SRF, TGF	225	253	6879	2.175178	0.535396	0.020505	4.387657
KEGG_PATHWAY	hsa05100:Bacterial invasion of epithelial cell	9	1.943844	0.003677	CRKL, ARPC3, RHOA, SHC1, ELMO2, CTNNA3, SEPT8	225	78	6879	3.527692	0.550344	0.020814	4.570497
KEGG_PATHWAY	hsa04915:Estrogen signaling pathway	10	2.159827	0.004766	EGFR, KRAS, SP1, FKBP5, GRB2, JUN, CREB1, ADCY6	225	99	6879	3.088215	0.645401	0.026234	5.887866
KEGG_PATHWAY	hsa04910:Insulin signaling pathway	12	2.591793	0.005199	PDPK1, KRAS, CRKL, GRB2, GSK3B, FLOT1, MKNK2,	225	138	6879	2.658551	0.677313	0.027881	6.405906
KEGG_PATHWAY	hsa04071:Sphingolipid signaling pathway	11	2.37581	0.005567	GNA13, PLD1, PDPK1, KRAS, ROCK2, MAPK14, ACER	225	120	6879	2.802556	0.702234	0.029115	6.845182
KEGG_PATHWAY	hsa04350:TGF-beta signaling pathway	9	1.943844	0.005789	ACVR1B, EP300, SP1, SMAD4, RHOA, SMAD3, SMAD	225	84	6879	3.275714	0.7163	0.029551	7.108655
KEGG_PATHWAY	hsa05130:Pathogenic Escherichia coli infecti	7	1.511879	0.0059	TUBB, ARPC3, TUBB2A, ROCK2, RHOA, TUBA1B, CT	225	51	6879	4.19634	0.723109	0.029422	7.240654
KEGG_PATHWAY	hsa05169:Epstein-Barr virus infection	11	2.37581	0.006249	CDKN1A, CDKN1B, PSMD11, TBK1, MAPK14, JUN, B	225	122	6879	2.756612	0.743405	0.030442	7.653032
KEGG_PATHWAY	hsa04152:AMPK signaling pathway	11	2.37581	0.006613	IGF1R, PDPK1, PFKFB3, CREB1, FOXO1, CREB5, CAB	225	123	6879	2.734201	0.763034	0.03149	8.082188
KEGG_PATHWAY	hsa04540:Gap junction	9	1.943844	0.007642	EGFR, TUBB, KRAS, TUBB2A, GRB2, ADCY6, GJA1, M	225	88	6879	3.126818	0.810731	0.03554	9.283418
KEGG_PATHWAY	hsa05217:Basal cell carcinoma	7	1.511879	0.007806	WNT1, WNT16, GSK3B, TP53, TCF7L2, TCF7L1, CTNN	225	54	6879	3.96321	0.817417	0.035535	9.474197
KEGG_PATHWAY	hsa04917:Prolactin signaling pathway	8	1.727862	0.008011	KRAS, CCND2, GRB2, MAPK14, GSK3B, SHC1, FOXO3	225	71	6879	3.444883	0.825432	0.03571	9.711725
KEGG_PATHWAY	hsa05202:Transcriptional misregulation in ca	13	2.807775	0.008127	CCNT2, BMI1, IGF1R, WNT16, CDKN1A, FLT1, CDKN1	225	167	6879	2.37996	0.829797	0.035493	9.845462
KEGG_PATHWAY	hsa04912:GnRH signaling pathway	9	1.943844	0.009301	EGFR, PLD1, KRAS, MAP3K3, GRB2, MAPK14, JUN, A	225	91	6879	3.023736	0.868358	0.039742	11.19096
KEGG_PATHWAY	hsa04921:Oxytocin signaling pathway	12	2.591793	0.009582	EGFR, CDKN1A, KRAS, RGS2, CACNG8, ROCK2, JUN,	225	150	6879	2.445867	0.876215	0.040137	11.5103
KEGG_PATHWAY	hsa04150:mTOR signaling pathway	7	1.511879	0.010989	PDPK1, CAB39, PRKAA2, IKBKB, PTEN, IRS1, DDIT4	225	58	6879	3.689885	0.909089	0.045066	13.09461
KEGG_PATHWAY	hsa05162:Measles	11	2.37581	0.011234	TNFRSF10A, CCNE2, CDKN1B, TNFRSF10B, CCND2,	225	133	6879	2.528622	0.913836	0.045201	13.367
KEGG_PATHWAY	hsa05160:Hepatitis C	11	2.37581	0.011234	EGFR, CDKN1A, PDPK1, KRAS, TBK1, GRB2, MAPK14	225	133	6879	2.528622	0.913836	0.045201	13.367
KEGG_PATHWAY	hsa05216:Thyroid cancer	5	1.079914	0.013715	KRAS, TP53, TCF7L2, TCF7L1, CTNNB1	225	29	6879	5.271264	0.950054	0.053985	16.08846
KEGG_PATHWAY	hsa05231:Choline metabolism in cancer	9	1.943844	0.016794	EGFR, PLD1, PDPK1, KRAS, WASF3, SP1, PLCG1, GRB	225	101	6879	2.724356	0.974659	0.064641	19.3557
KEGG_PATHWAY	hsa05131:Shigellosis	7	1.511879	0.017341	PFN1, CRKL, ARPC3, ROCK2, MAPK14, IKBKB, ELMO	225	64	6879	3.343958	0.977538	0.065538	19.92294
KEGG_PATHWAY	hsa04062:Chemokine signaling pathway	13	2.807775	0.017901	ROCK2, GRB2, ADCY6, FOXO3, STAT3, CCL22, CRKL	225	186	6879	2.136846	0.980154	0.066456	20.50111
KEGG_PATHWAY	hsa04022:cGMP-PKG signaling pathway	11	2.37581	0.033099	GNA13, RGS2, ROCK2, CREB1, ADCY6, GATA4, RHO	225	158	6879	2.128523	0.999327	0.118323	34.78681
KEGG_PATHWAY	hsa04670:Leukocyte transendothelial migrati	9	1.943844	0.033507	VCAM1, ACTN4, PLCG1, ROCK2, MAPK14, RHOA, MS	225	115	6879	2.392696	0.999386	0.117812	35.13598
KEGG_PATHWAY	hsa00270:Cysteine and methionine metabolis	5	1.079914	0.034001	DNMT3A, LDHA, GOT1, DNMT1, DNMT3B	225	38	6879	4.022807	0.999451	0.117601	35.556
KEGG_PATHWAY	hsa05221:Acute myeloid leukemia	6	1.295896	0.034826	KRAS, GRB2, IKBKB, TCF7L2, TCF7L1, STAT3	225	56	6879	3.275714	0.999544	0.118471	36.25106
KEGG_PATHWAY	hsa04210:Apoptosis	6	1.295896	0.050549	TNFRSF10A, TNFRSF10B, XIAP, BCL2, TP53, IKBKB	225	62	6879	2.95871	0.999987	0.166024	48.25452
KEGG_PATHWAY	hsa04962:Vasopressin-regulated water reabs	5	1.079914	0.05386	RAB5B, CREB1, ADCY6, CREB5, ARHGDIA	225	44	6879	3.474242	0.999994	0.17362	50.50011
KEGG_PATHWAY	hsa05120:Epithelial cell signaling in Helicob	6	1.295896	0.066373	EGFR, PLCG1, MAPK14, JUN, ATP6V1E1, IKBKB	225	67	6879	2.73791	1	0.207738	58.2012
KEGG_PATHWAY	hsa05412:Arrhythmogenic right ventricular c	6	1.295896	0.066373	CACNG8, GJA1, TCF7L2, TCF7L1, CTNNA3, CTNNB1	225	67	6879	2.73791	1	0.207738	58.2012
KEGG_PATHWAY	hsa04662:B cell receptor signaling pathway	6	1.295896	0.073394	KRAS, GRB2, JUN, GSK3B, MALT1, IKBKB	225	69	6879	2.658551	1	0.224681	62.02267
KEGG_PATHWAY	hsa04622:RIG-I-like receptor signaling path	6	1.295896	0.077051	CYLD, ATG12, TBK1, MAPK14, IKBKB, PIN1	225	70	6879	2.620571	1	0.231741	63.88311
KEGG_PATHWAY	hsa05016:Huntington's disease	11	2.37581	0.096221	TFAM, BDNF, EP300, SP1, CREB1, TP53, COX6B1, AT	225	192	6879	1.751597	1	0.2794	72.33466
KEGG_PATHWAY	hsa04141:Protein processing in endoplasmic	10	2.159827	0.099734	SEC23A, UBE2D4, SEC24A, ERO1A, BAG1, BCL2, MA	225	169	6879	1.809073	1	0.284863	73.66987

Category	Term	Count	%	PValue	Genes	List Total	Pop Hits	Pop Total	Fold Enricl	Bonferroni	Benjamini	FDR
KEGG PA	hsa05200:Pathways in cancer	66	4.925373	1.03E-09	HSP90AB1, E2F1, F2RL3, E2F2, PPARD, E2F3,	527	393	6879	2.192129	2.72E-07	2.72E-07	1.35E-06
KEGG PA	hsa04350:TGF-beta signaling pathway	25	1.865672	7.89E-09	E2F5, BMPR2, TGFB2, ACVR1B, ZFYVE9, TH	527	84	6879	3.88486	2.07E-06	1.04E-06	1.03E-05
KEGG PA	hsa04110:Cell cycle	28	2.089552	4.65E-07	E2F1, MAD1L1, E2F2, YWHAZ, E2F3, E2F5, PI	527	124	6879	2.947481	1.22E-04	4.08E-05	6.09E-04
KEGG PA	hsa04144:Endocytosis	42	3.134328	7.25E-07	CAV1, PARD3, RAB5B, LDLR, TSG101, CAPZ	527	241	6879	2.274819	1.91E-04	4.76E-05	9.49E-04
KEGG PA	hsa05219:Bladder cancer	15	1.119403	1.00E-06	E2F1, RPS6KA5, E2F2, MAPK1, NRAS, E2F3, C	527	41	6879	4.775536	2.64E-04	5.28E-05	0.001315
KEGG PA	hsa05220:Chronic myeloid leukemia	20	1.492537	1.13E-06	E2F1, E2F2, E2F3, TGFBR1, TGFBR2, TP53, SM	527	72	6879	3.62587	2.97E-04	4.95E-05	0.001478
KEGG PA	hsa05161:Hepatitis B	30	2.238806	1.15E-06	E2F1, MAVS, E2F2, YWHAZ, E2F3, PTEN, TGI	527	145	6879	2.700648	3.03E-04	4.33E-05	0.001509
KEGG PA	hsa05215:Prostate cancer	22	1.641791	1.87E-06	E2F1, HSP90AB1, E2F2, E2F3, HSP90AA1, PDC	527	88	6879	3.263283	4.90E-04	6.13E-05	0.002442
KEGG PA	hsa04390:Hippo signaling pathway	30	2.238806	2.74E-06	YWHAZ, PARD3, BMPR2, WWC1, TCF7L2, TC	527	151	6879	2.593337	7.21E-04	8.01E-05	0.003589
KEGG PA	hsa05166:HTLV-I infection	42	3.134328	2.94E-06	E2F1, CRTC3, E2F2, E2F3, NRP1, XIAP, PDGFI	527	254	6879	2.158392	7.74E-04	7.74E-05	0.003854
KEGG PA	hsa05212:Pancreatic cancer	17	1.268657	2.08E-05	E2F1, E2F2, E2F3, TGFBR1, TGFBR2, TP53, SM	527	65	6879	3.413896	0.005451	4.97E-04	0.027212
KEGG PA	hsa04115:p53 signaling pathway	17	1.268657	3.12E-05	ZMAT3, CYCS, TP53, PMAIP1, SESN2, SESN1	527	67	6879	3.311988	0.008177	6.84E-04	0.040872
KEGG PA	hsa05205:Proteoglycans in cancer	33	2.462687	4.41E-05	CAV1, PPP1R12B, ITGB1, TGFB2, WNT1, PDP	527	200	6879	2.153767	0.011527	8.91E-04	0.057708
KEGG PA	hsa05210:Colorectal cancer	16	1.19403	4.68E-05	MSH3, TGFBR1, TGFBR2, CYCS, TP53, SMAD	527	62	6879	3.36855	0.012227	8.78E-04	0.061235
KEGG PA	hsa04068:FoxO signaling pathway	25	1.865672	6.54E-05	PTEN, TGFB2, PDPK1, S1PR1, SOS2, RBL2, TC	527	134	6879	2.435286	0.017046	0.001146	0.085567
KEGG PA	hsa04151:PI3K-Akt signaling pathway	47	3.507463	1.18E-04	HSP90AB1, YWHAZ, FGF7, PHLPP2, MCL1, PI	527	345	6879	1.778253	0.030528	0.001936	0.154243
KEGG PA	hsa05203:Viral carcinogenesis	31	2.313433	3.86E-04	MAD1L1, YWHAZ, PMAIP1, GTF2H2C, HIST1	527	205	6879	1.973888	0.096623	0.00596	0.504655
KEGG PA	hsa05206:MicroRNAs in cancer	39	2.910448	4.95E-04	E2F1, KIF23, E2F2, E2F3, MCL1, PDGFB, DICE	527	286	6879	1.779972	0.122175	0.007213	0.646693
KEGG PA	hsa04071:Sphingolipid signaling pathway	21	1.567164	6.94E-04	PPP2R1A, SGPL1, ROCK1, SPTLC2, ACER2, T	527	120	6879	2.284298	0.166816	0.009559	0.904539
KEGG PA	hsa04310:Wnt signaling pathway	23	1.716418	7.15E-04	CSNK1A1, FZD9, TBL1XR1, PPARD, WNT10B	527	138	6879	2.175522	0.171431	0.009359	0.931936
KEGG PA	hsa04010:MAPK signaling pathway	35	2.61194	7.99E-04	FGF7, PDGFB, MAPKAPK5, PPP3R1, MKNK2,	527	253	6879	1.805769	0.189616	0.009962	1.041338
KEGG PA	hsa05169:Epstein-Barr virus infection	21	1.567164	8.61E-04	NFKBIB, MAP2K3, TP53, HLA-A, HLA-C, RB1	527	122	6879	2.24685	0.202655	0.010241	1.121226
KEGG PA	hsa05221:Acute myeloid leukemia	13	0.970149	8.73E-04	PPARD, KIT, TCF7L2, STAT3, NRAS, MAPK1,	527	56	6879	3.030191	0.205244	0.009938	1.137241
KEGG PA	hsa05214:Glioma	14	1.044776	0.001072	E2F1, E2F2, E2F3, PDGFB, TP53, RB1, PTEN,	527	65	6879	2.811444	0.245753	0.011683	1.394414
KEGG PA	hsa05222:Small cell lung cancer	16	1.19403	0.001755	E2F1, E2F2, E2F3, XIAP, CYCS, TP53, ITGA2,	527	85	6879	2.45706	0.369943	0.018308	2.273731
KEGG PA	hsa05218:Melanoma	14	1.044776	0.002485	E2F1, E2F2, E2F3, FGF7, PDGFB, MET, TP53,	527	71	6879	2.573857	0.480298	0.024859	3.206166
KEGG PA	hsa04810:Regulation of actin cytoskeleton	29	2.164179	0.002545	FGF7, PDGFB, DIAPH2, PPP1R12B, SSH2, ABI	527	210	6879	1.802575	0.488417	0.024518	3.282018
KEGG PA	hsa05211:Renal cell carcinoma	13	0.970149	0.003821	EPAS1, PDGFB, MET, EGLN3, TGFB2, PAK6,	527	66	6879	2.571071	0.634602	0.035317	4.889047
KEGG PA	hsa04510:Focal adhesion	28	2.089552	0.00384	CAV1, XIAP, PDGFB, PPP1R12B, ARHGAP35,	527	206	6879	1.774212	0.636474	0.034292	4.913368
KEGG PA	hsa04722:Neurotrophin signaling pathway	19	1.41791	0.004189	NFKBIB, TP53, YWHAE, IRS1, IRAK4, RPS6K	527	120	6879	2.066746	0.668498	0.036135	5.348939
KEGG PA	hsa04612:Antigen processing and presentat	14	1.044776	0.004599	HSP90AB1, HSP90AA1, PDIA3, CREB1, HLA-	527	76	6879	2.404524	0.702486	0.038351	5.857348
KEGG PA	hsa04550:Signaling pathways regulating pl	21	1.567164	0.004632	FZD9, BMP2, WNT10B, JARID2, SMAD5, BMF	527	140	6879	1.95797	0.705064	0.037437	5.898138
KEGG PA	hsa05216:Thyroid cancer	8	0.597015	0.0052	MAPK1, NRAS, CCDC6, CCND1, PPARG, TP5	527	29	6879	3.600864	0.746214	0.040702	6.599547
KEGG PA	hsa04012:ErbB signaling pathway	15	1.119403	0.005865	NRG4, PRKCB, PAK6, NRAS, MAPK1, CDKN1	527	87	6879	2.25054	0.787149	0.044485	7.413964
KEGG PA	hsa05230:Central carbon metabolism in car	12	0.895522	0.008502	MAPK1, SLC1A5, NRAS, HIF1A, MET, TP53, P	527	64	6879	2.447462	0.894139	0.062146	10.57839
KEGG PA	hsa05145:Toxoplasmosis	17	1.268657	0.009046	XIAP, LDLR, NFKBIB, MAP2K3, CYCS, ITGB1	527	110	6879	2.017302	0.908353	0.064228	11.21799
KEGG PA	hsa05223:Non-small cell lung cancer	11	0.820896	0.009076	E2F1, MAPK1, NRAS, E2F2, PDPK1, E2F3, CCI	527	56	6879	2.564008	0.909089	0.062752	11.25364
KEGG PA	hsa03018:RNA degradation	13	0.970149	0.01327	PAN3, EXOSC2, PFKP, PAPD5, DIS3L, CNOT7	527	77	6879	2.203775	0.970204	0.088312	16.04827
KEGG PA	hsa05213:Endometrial cancer	10	0.746269	0.015865	MAPK1, NRAS, PDPK1, CCND1, SOS2, TP53, I	527	52	6879	2.510217	0.985092	0.102231	18.89338
KEGG PA	hsa04962:Vasopressin-regulated water reab	9	0.671642	0.016906	STX4, DYNC1LI2, RAB5B, DYNLL2, CREB1, I	527	44	6879	2.669959	0.988715	0.106052	20.01013
KEGG PA	hsa04520:Adherens junction	12	0.895522	0.018077	MAPK1, PARD3, CSNK2A1, TGFBR1, TGFBR2	527	71	6879	2.206163	0.991752	0.110431	21.24875
KEGG PA	hsa05164:Influenza A	22	1.641791	0.024009	MAVS, NUP98, NFKBIB, MAP2K3, CYCS, CCI	527	174	6879	1.650396	0.998324	0.141161	27.25557
KEGG PA	hsa05162:Measles	18	1.343284	0.024378	MAVS, NFKBIB, TP53, TLR7, STAT3, IFNAR1	527	133	6879	1.766589	0.998483	0.140112	27.61565
KEGG PA	hsa04360:Axon guidance	17	1.268657	0.032001	ABLIM1, NRP1, ROCK1, PLXNA1, LIMK1, ME	527	127	6879	1.747269	0.999807	0.176676	34.68107
KEGG PA	hsa05160:Hepatitis C	17	1.268657	0.046124	MAVS, PPP2R1A, LDLR, TP53, STAT3, IFNAR	527	133	6879	1.668445	0.999996	0.241176	46.11653
KEGG PA	hsa05217:Basal cell carcinoma	9	0.671642	0.051093	FZD9, WNT1, BMP2, WNT10B, TP53, FZD5, T	527	54	6879	2.175522	0.999999	0.259069	49.67861
KEGG PA	hsa05131:Shigellosis	10	0.746269	0.053051	MAPK1, ROCK1, ITGA5, ARPC2, NFKBIB, MA	527	64	6879	2.039552	0.999999	0.262893	51.02104
KEGG PA	hsa04919:Thyroid hormone signaling pathv	15	1.119403	0.054818	ATP1B3, PFKFB2, TP53, PRKCB, NRAS, MAPI	527	115	6879	1.702582	1	0.265747	52.20438
KEGG PA	hsa04066:HIF-1 signaling pathway	13	0.970149	0.061103	EGLN3, MKNK2, PDHB, STAT3, PRKCB, MAP	527	96	6879	1.767611	1	0.287097	56.20307
KEGG PA	hsa05416:Viral myocarditis	9	0.671642	0.066615	EIF4G2, CAV1, CCND1, CYCS, HLA-A, HLA-C	527	57	6879	2.061021	1	0.304144	59.45295
KEGG PA	hsa04150:mTOR signaling pathway	9	0.671642	0.07237	MAPK1, EIF4EBP1, PDPK1, ULK1, RRAGD, IK	527	58	6879	2.025486	1	0.321179	62.6074
KEGG PA	hsa04380:Osteoclast differentiation	16	1.19403	0.074124	CREB1, TGFBR1, PPARG, TGFBR2, PPP3R1, S	527	131	6879	1.594276	1	0.322616	63.52237
KEGG PA	hsa04916:Melanogenesis	13	0.970149	0.078274	FZD9, WNT10B, CREB1, KIT, FZD5, TCF7L2,	527	100	6879	1.696907	1	0.332663	65.60635
KEGG PA	hsa04152:AMPK signaling pathway	15	1.119403	0.085701	PPP2R1A, CREB1, PFKFB2, SCD, PPARG, PFK	527	123	6879	1.591845	1	0.353625	69.06373
KEGG PA	hsa04917:Prolactin signaling pathway	10	0.746269	0.090177	MAPK1, NRAS, CCND1, CCND2, SOS2, MAPK	527	71	6879	1.838469	1	0.363586	70.98915
KEGG PA	hsa04141:Protein processing in endoplasmi	19	1.41791	0.093931	SEC23A, HSP90AB1, NPLOC4, HSP90AA1, PD	527	169	6879	1.467512	1	0.370769	72.51817
KEGG PA	hsa05142:Chagas disease (American trypar	13	0.970149	0.098236	PPP2R1A, TGFBR1, TGFBR2, SMAD2, CCL5,	527	104	6879	1.631641	1	0.379421	74.17959
